# The opportunistic pathogen *Stenotrophomonas maltophilia* utilizes a type IV secretion system for interbacterial killing

**DOI:** 10.1371/journal.ppat.1007651

**Published:** 2019-09-12

**Authors:** Ethel Bayer-Santos, William Cenens, Bruno Yasui Matsuyama, Gabriel Umaji Oka, Giancarlo Di Sessa, Izabel Del Valle Mininel, Tiago Lubiana Alves, Chuck Shaker Farah

**Affiliations:** 1 Departamento de Bioquímica, Instituto de Química, Universidade de São Paulo, São Paulo, São Paulo, Brazil; 2 Departamento de Microbiologia, Instituto de Ciências Biomédicas, Universidade de São Paulo, São Paulo, São Paulo, Brazil; University of North Carolina at Chapel Hil, UNITED STATES

## Abstract

Bacterial type IV secretion systems (T4SS) are a highly diversified but evolutionarily related family of macromolecule transporters that can secrete proteins and DNA into the extracellular medium or into target cells. It was recently shown that a subtype of T4SS harboured by the plant pathogen *Xanthomonas citri* transfers toxins into target cells. Here, we show that a similar T4SS from the multi-drug-resistant opportunistic pathogen *Stenotrophomonas maltophilia* is proficient in killing competitor bacterial species. T4SS-dependent duelling between *S*. *maltophilia* and *X*. *citri* was observed by time-lapse fluorescence microscopy. A bioinformatic search of the *S*. *maltophilia* K279a genome for proteins containing a C-terminal domain conserved in *X*. *citri* T4SS effectors (XVIPCD) identified twelve putative effectors and their cognate immunity proteins. We selected a putative *S*. *maltophilia* effector with unknown function (Smlt3024) for further characterization and confirmed that it is indeed secreted in a T4SS-dependent manner. Expression of Smlt3024 in the periplasm of *E*. *coli* or its contact-dependent delivery via T4SS into *E*. *coli* by *X*. *citri* resulted in reduced growth rates, which could be counteracted by expression of its cognate inhibitor Smlt3025 in the target cell. Furthermore, expression of the VirD4 coupling protein of *X*. *citri* can restore the function of *S*. *maltophilia* Δ*virD4*, demonstrating that effectors from one species can be recognized for transfer by T4SSs from another species. Interestingly, Smlt3024 is homologous to the N-terminal domain of large Ca^2+^-binding RTX proteins and the crystal structure of Smlt3025 revealed a topology similar to the iron-regulated protein FrpD from *Neisseria meningitidis* which has been shown to interact with the RTX protein FrpC. This work expands our current knowledge about the function of bacteria-killing T4SSs and increases the panel of effectors known to be involved in T4SS-mediated interbacterial competition, which possibly contribute to the establishment of *S*. *maltophilia* in clinical and environmental settings.

## Author summary

Competition between microorganisms determines which species will dominate or be eradicated from a specific habitat. Bacteria use a series of mechanisms to kill or prevent multiplication of competitors. We show that an opportunistic pathogen, *Stenotrophomonas maltophilia*, harbours a type IV secretion system (T4SS) that works as a weapon to kill competitor bacterial species. We identified a series of new putative toxic T4SS effectors secreted by *S*. *maltophilia* and their cognate immunity proteins. Characterization of one *S*. *maltophilia* effector with unknown function (Smlt3024) shows that it reduces growth rate of *E*. *coli* cells. Its cognate immunity protein, Smlt3025, presents a structure similar to the FrpD lipoprotein from *Neisseria meningitidis*. Smlt3024 expressed in the plant pathogen *Xanthomonas citri* can be translocated into *E*. *coli* cells, highlighting the interchangeable characteristic of T4SSs toxins and the conservation of secretion system function. We show that *X*. *citri* and *S*. *maltophilia* can kill each other in a T4SS-dependent manner, most likely due to differences in their cohorts of effector-immunity protein pairs. This work expands our current knowledge about the function of bacteria-killing T4SSs and the bacterial arsenal fired by these systems during encounters with other species.

## Introduction

The ecological interactions between bacterial species range from cooperative to competitive and can be mediated by diffusible soluble factors secreted into the extracellular medium or by factors transferred directly into target cells in a contact-dependent manner [1]. Several types of contact-dependent antagonistic interactions between bacteria have been described [1]. Contact-dependent growth inhibition (CDI) is mediated by the CdiA/CdiB family of two-partner secretion proteins in which the outer membrane protein CdiB is required for secretion of the CdiA toxin [2, 3]. The type VI secretion system (T6SS) is a dynamic contractile organelle evolutionarily related to bacteriophage tails, enabling the injection of proteinaceous effectors into target prokaryotic or eukaryotic cells [4, 5]. A specialized secretion system widely distributed among Gram-positive bacteria called Esx pathway or type VII secretion system (T7SS) induces contact-dependent cell death [6, 7]. An atypical bacteriocin system in *Caulobacter crescentus* called contact-dependent inhibition by glycine zipper proteins (Cdz) was also reported [8]. Another distinct contact-dependent toxin delivery mechanism is that of outer membrane exchange (OME) described in the social bacterium *Myxococcus xanthus* [9]. Contact-dependent antagonism has also been shown to be mediated via a specialized type IV secretion system (T4SS) that transports toxic effectors into target prokaryotic cells [10, 11].

T4SSs are a highly diverse superfamily of secretion systems found in many species of Gram-negative and Gram-positive bacteria. These systems mediate a wide range of events from transfer of DNA during bacterial conjugation to transfer of effector proteins into eukaryotic host cells [12] and into competitor bacteria [10]. T4SSs have been classified based on their physiological functions as (i) conjugation systems, (ii) effector translocators, or (iii) contact-independent DNA/protein exchange systems [13]. Another common classification scheme divides T4SSs into two phylogenetic families called types A and B [14, 15]; while more finely discriminating phylogenetic analyses based on two highly conserved T4SS ATPases (VirB4 and VirD4) identified eight distinct clades [16, 17].

The model type A VirB/D4 T4SS from *Agrobacterium tumefaciens*, which is used to transfer tumour-inducing effectors into some plant species [18], is composed of a core set of 12 proteins designated VirB1-VirB11 and VirD4. Electron microscopy studies on homologous systems from the conjugative plasmids R388 and pKM101 [19–21] have revealed an architecture that can be divided into two large subcomplexes: i) a periplasmatic core complex made up of 14 repeats of VirB7, VirB9 and VirB10 subunits that forms a pore in the outer membrane and which is also linked, via VirB10, to the inner membrane and ii) an inner membrane complex composed of VirB3, VirB6 and VirB8 and three ATPases (VirB4, VirB11 and VirD4) that energize the system during pilus formation and substrate transfer. Finally, VirB2 and VirB5 form the extracellular pilus and VirB1 is a periplasmic transglycosidase [22–24]. The *X*. *citri* T4SS involved in bacterial killing, and its homologues in other bacterial species (together called X-T4SSs for Xanthomonadales-like T4SSs), share many features with the type A T4SSs from *A*. *tumefaciens* and the conjugative T4SSs pKM101 and R388, with one distinctive feature being an uncharacteristically large VirB7 lipoprotein subunit [25] whose C-terminal N0 domain decorates the periphery of the outer membrane layer of the core complex [11, 26].

VirD4 and its orthologs play a key role by recognizing substrates on the cytoplasmic face of the inner membrane and directing them for secretion through the T4SS channel [14, 27–29]. A yeast two-hybrid screen using *X*. *citri* VirD4 as bait identified several prey proteins (initially termed XVIPs for *X**anthomonas* VirD4 interacting proteins) containing a conserved C-terminal domain named XVIPCD (XVIP conserved domain) [30]. These proteins were later shown to be putative antibacterial effectors secreted via the *X*. *citri* T4SS into target cells, often carrying N-terminal domains with enzymatic activities predicted to target structures in the cell envelope, including peptidoglycan-targeting glycohydrolases and proteases, phospholipases, as well as nucleases [10]. Furthermore, each T4SS effector is co-expressed with a cognate immunity protein, which is predicted to prevent self-intoxication [10], a feature also observed for effector-immunity pairs associated with T6SSs [31]. Bioinformatic analysis identified potential XVIPCD-containing proteins in many other bacterial species of the Xanthomonadales order, including *Stenotrophomonas* spp., *Lysobacter* spp., *Luteimonas* spp., *Luteibacter* spp. and *Dyella* spp. Therefore, these effectors and their cognate immunity proteins were generally designated X-Tfes and X-Tfis (Xanthomonadales T4SS effectors and immunity proteins, respectively) [10, 11].

*Stenotrophomonas maltophilia* is an emerging multi-drug-resistant global opportunistic pathogen. *S*. *maltophilia* strains are frequently isolated from water, soil and in association with plants [32], but in the last decades an increased number of hospital-acquired infections, particularly of immunocompromised patients, has called attention to this opportunistic pathogen [33, 34]. Infections associated with virulent strains of *S*. *maltophilia* are very diverse, ranging from respiratory and urinary tract infections to bacteremia and infections associated with intravenous cannulas and prosthetic devices [33]. The ability of *Stenotrophomonas* spp. to form biofilms on different biotic and abiotic surfaces [35, 36] and its capacity to secrete several hydrolytic enzymes (proteases, lipases, esterases) that promote cytotoxicity both contribute to pathogenesis [37, 38]. In addition, *S*. *maltophilia* is naturally competent to acquire foreign DNA, which probably contributes to the multi-drug-resistant phenotype of several strains [32, 39].

**Fig 1:**
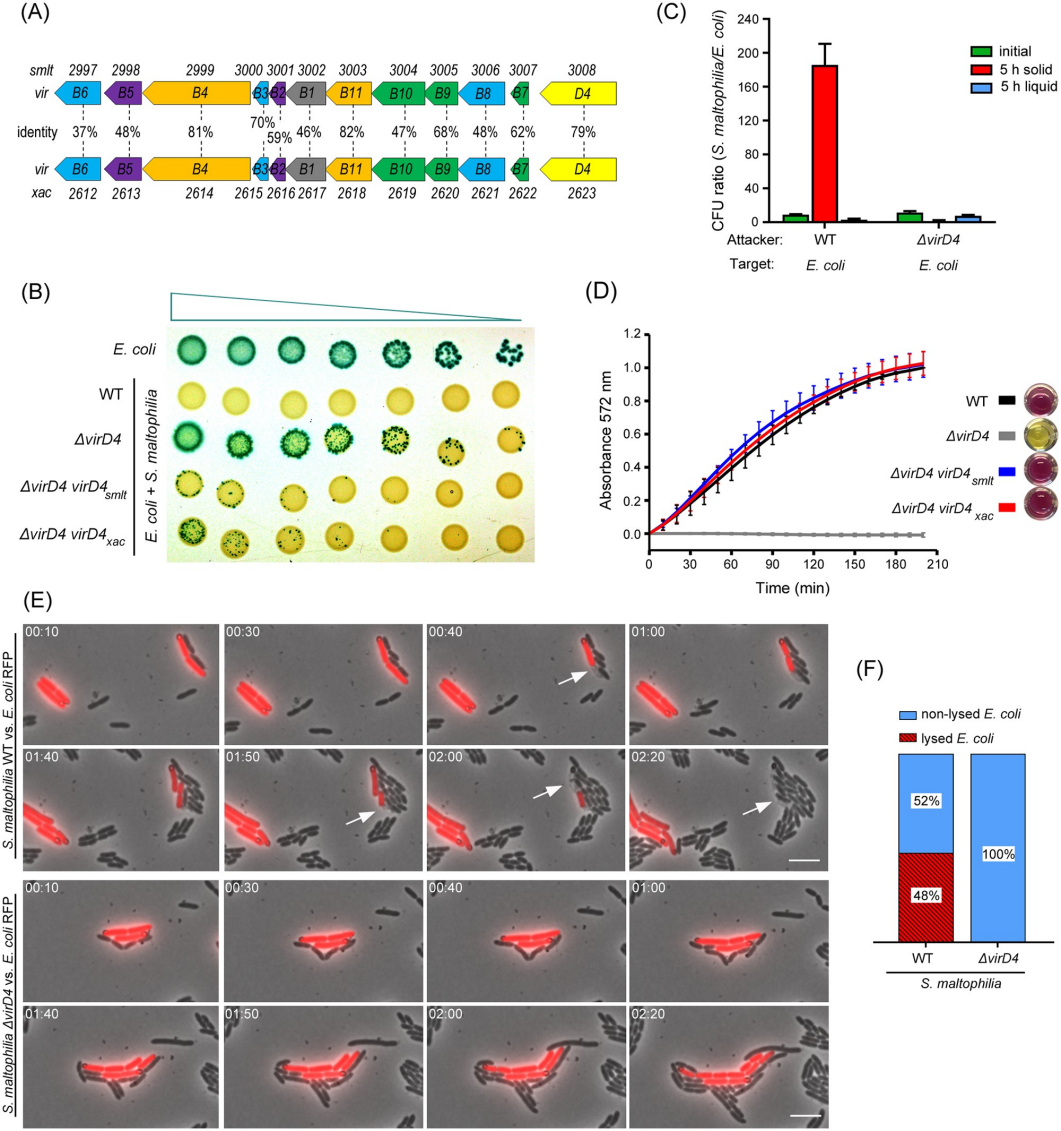
*S*. *maltophilia* uses the X-T4SS to induce *E*. *coli* cell death in a contact-dependent manner. (A) Schematic representation of the organization of the chromosomal *virB1-11* and *virD4* genes encoding the X-T4SSs of *S*. *maltophilia* K279a and *X*. *citri* 306. The amino acid identities (%) between homologues are shown. (B) Bacterial competition assay using *S*. *maltophilia* strains (wild-type, Δ*virD4* and complemented strains Δ*virD4 virD4*_*smlt*_ and Δ*virD4 virD4*_*xac*_ and *E*. *coli* (naturally expressing β-galactosidase). A serial dilution of *E*. *coli* (1:4) was mixed with constant amounts of *S*. *maltophilia*, spotted onto LB-agar containing IPTG and X-gal and incubated for 24 h at 30°C. Representative image of three independent experiments. (C) CFUs ratio of either wild-type or Δ*virD4 S*. *maltophilia* (attacker) to *E*. *coli* (target) recovered after 5 h of co-culture in solid or liquid media. CFUs ratios of mixed cultures at the initial time-point was included as a control. (D) Quantification of *E*. *coli* target cell lysis using the cell-impermeable compound CPRG. The same bacterial strains described in (B) were used. Graph represents the means and standard deviation (SD) of three independent experiments performed in triplicate. The slopes in the linear part of the curves (between 50 and 100 min) is proportional to the amount of β-galactosidase released by the lysed *E*. *coli* cells. (E) Representative images of time-lapse microscopy showing wild-type *S*. *maltophilia* interacting with *E*. *coli*-RFP (upper panel) at the single cell level. Images were acquired every 10 min. Dead/lysed *E*. *coli*-RFP cells are indicated by white arrows. Interaction between *S*. *maltophilia* Δ*virD4* and *E*. *coli*-RFP strains (lower panel) did not induce target cell lysis. Timestamps in hours:minutes. Scale bar 5 μm. (F) Percentage of dead/lysed *E*. *coli* cells after cell-to-cell contact with *Stenotrophomonas* strains over a 100 min timeframe.

*S*. *maltophilia* strain K279a contains a cluster of genes (*virB1-virB11* and *virD4*) on its chromosome coding for a T4SS homologous to the X-T4SS of the plant pathogen *Xanthomonas citri* involved in interbacterial antagonism [10], and their cytoplasmic ATPases VirD4 share 79% amino acid identity (Fig 1A). In this study, we show that *S*. *maltophilia* K279a is proficient in inducing the death of several other Gram-negative bacterial species in a T4SS-dependent manner. Interestingly, *S*. *maltophilia* and *X*. *citri* can duel using their T4SSs and kill each other. A bioinformatic search of the *S*. *maltophilia* K279a genome for proteins containing a C-terminal domain conserved in *X*. *citri* T4SS effectors (XVIPCD) identified twelve putative effectors. We selected a putative *S*. *maltophilia* effector with unknown function (Smlt3024) for further characterization and confirmed that it is indeed secreted in a contact- and T4SS-dependent manner. Heterologous expression of Smlt3024 in the periplasm of *E*. *coli* reduced growth rate, which could be counteracted by co-expression with its cognate immunity protein, Smlt3025. Using an *X*. *citri* strain that is deficient in target cell lysis due to the lack of nine X-Tfes but proficient in substrate delivery, we show that Smlt3024 can be translocated via the T4SS into target *E*. *coli* cells. Furthermore, heterologous expression of the *X*. *citri* VirD4 coupling protein in the *S*. *maltophilia* Δ*virD4* strain can restore T4SS function. These results highlight the conservation of X-T4SS function and the interchangeable usage of T4SSs effectors by different species. Interestingly, the crystal structure of Smlt3025 revealed a topology similar to the iron-regulated protein FrpD, the cognate binding partner of FrpC, an RTX protein of unknown function secreted by the type I secretion system (T1SS) of *Neisseria meningitidis*. This work expands our current knowledge about the mechanism of bacteria-killing T4SSs and the bacterial arsenal fired by these systems in encounters with other species.

## Results

### The *Stenotrophomonas maltophilia* X-T4SS induces target bacteria cell death

The genome of *S*. *maltophilia* K279a [40] harbours two clusters of genes encoding distinct T4SSs: *smlt2997-smlt3008* (annotated as *virB*) and *smlt1283-smlt1293* (annotated as *trb*) [41]. Comparative sequence analysis showed that the *S*. *maltophilia virB1-11 and virD4* genes are most closely related with their counterparts in the *X*. *citri* T4SS involved in bacteria killing (X-T4SS) (37% − 82% identity at the amino acid level), with the three ATPases that energize the system presenting the greatest levels of identity: VirB4 (81%), VirB11 (82%) and VirD4 (79%) (Fig 1A). Phylogenetic analysis based on the amino acid sequences of *S*. *maltophilia* VirD4/Smlt3008 grouped the *S*. *maltophilia* VirB/T4SS together with the *X*. *citri* X-T4SS involved in bacterial killing, while *Stenotrophomonas* Trb/T4SS, for which no functional information is available, belongs to another group of T4SSs (S1 Fig). The second T4SS from *X*. *citri* (encoded by plasmid pXAC64), which was proposed to be involved in conjugation due to neighbouring relaxosome genes and *oriT* site [30], is located in another branch of the phylogenetic tree, distinct from the two systems described above (S1 Fig).

To investigate the involvement of the *S*. *maltophilia* X-T4SS in bacterial antagonism, we created a mutant strain lacking the ATPase coupling protein VirD4 (Δ*virD4*) and analysed its ability to restrict growth of other species such as *E*. *coli*. Different dilutions of an *E*. *coli* culture were mixed with a fixed number of *S*. *maltophilia* cells and the co-cultures were spotted onto LB-agar plates containing the chromogenic substrate X-gal and incubated for 24 h at 30°C (Fig 1B). As only *E*. *coli* cells naturally express β-galactosidase, they turn blue while *S*. *maltophilia* cells are yellow. Growth of *E*. *coli* was inhibited by *S*. *maltophilia* wild-type, but not by the Δ*virD4* strain (Fig 1B). The phenotype of *S*. *maltophilia* Δ*virD4* could be restored by complementing the strain with a plasmid encoding VirD4 (*smlt3008*) under the control of the P_BAD_ promoter (Δ*virD4 virD4*_*smlt*_) (Fig 1B). This plasmid promotes low expression levels sufficient for complementation under non-inducing conditions (no L-arabinose) in *Stenotrophomonas*. Interestingly, transformation of *S*. *maltophilia* Δ*virD4* strain with a plasmid encoding VirD4 from *X*. *citri* (*xac2623*) (Δ*virD4 virD4*_*xac*_) also restored the phenotype (Fig 1B), indicating that the *X*. *citri* protein is able to couple substrates to the *S*. *maltophilia* translocation apparatus. The *S*. *maltophilia* T4SS-dependent antibacterial effect is only detected in co-cultures incubated on solid LB-agar surfaces where cell-cell contact is frequent and long-lasting; no effect on target cell growth is observed when *S*. *maltophilia* and *E*. *coli* are co-cultured in liquid media (Fig 1C).

To analyse whether the antagonism mediated by the *S*. *maltophilia* T4SS is due to target cell lysis, *E*. *coli* cells were mixed with different *S*. *maltophilia* strains (wild-type, Δ*virD4*, Δ*virD4 virD4*_*smlt*_ and Δ*virD4 virD4*_*xac*_*)* and spotted onto 96 well plates containing LB-agar with CPRG. CPRG is a cell-impermeable chromogenic substrate hydrolysed by β-galactosidase released from lysed *E*. *coli*, thus producing chlorophenol red with an absorbance maximum at 572 nm [26, 42]. Fig 1D shows that *S*. *maltophilia* wild-type and complemented strains (Δ*virD4 virD4*_*smlt*_ and Δ*virD4 virD4*_*xac*_) induce lysis of *E*. *coli* with very similar efficiencies (based on the slopes of the curves) while the Δ*virD4* strain does not induce target cell lysis.

**Fig 2:**
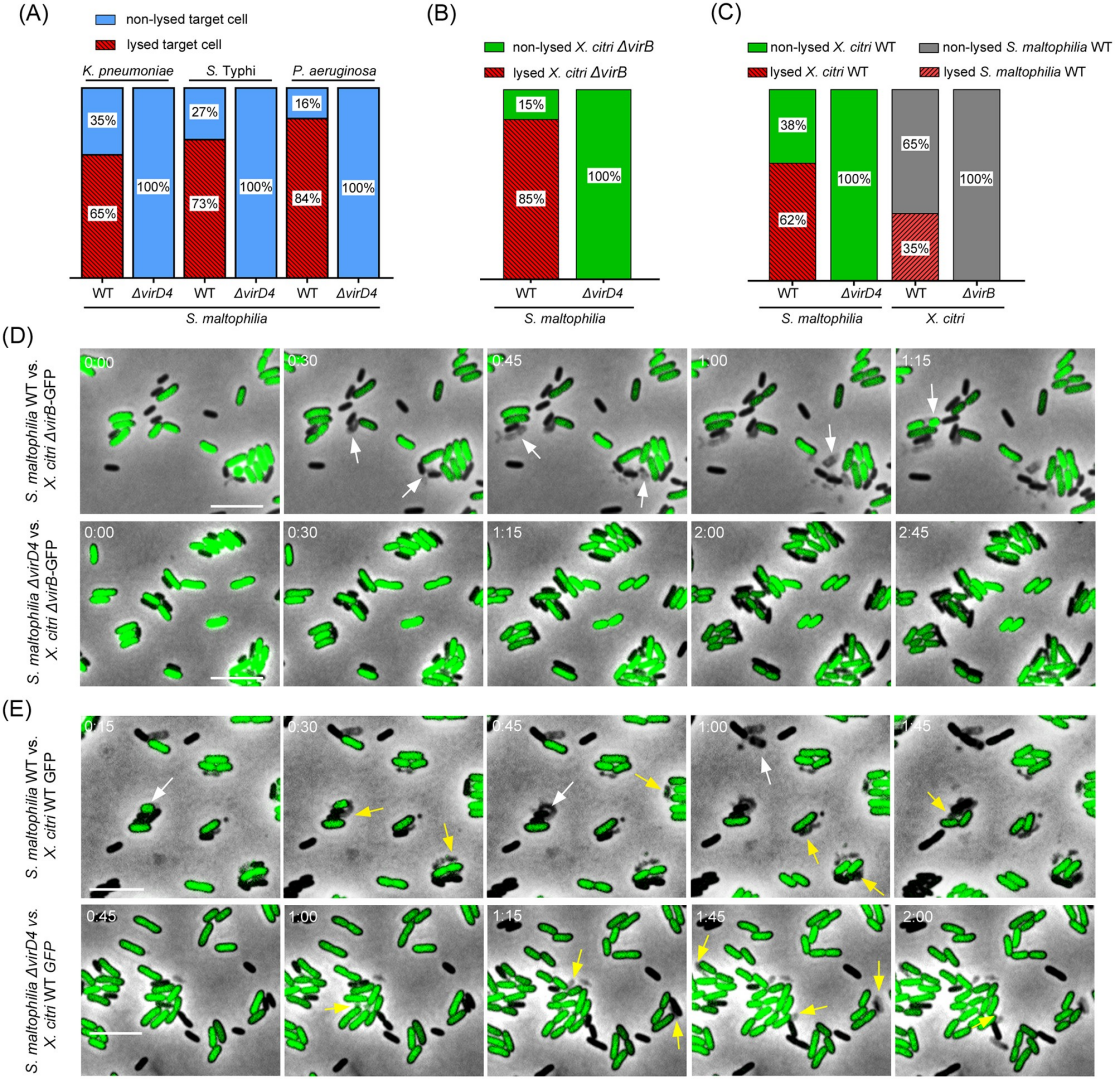
*S*. *maltophilia* kills Gram-negative species and duels with *X*. *citri* in a T4SS-dependent manner. (A) Percentage of dead/lysed *Klebsiella pneumoniae*, *Salmonella* Typhi and *Pseudomonas aeruginosa* after cell-to-cell contact with *Stenotrophomonas* strains over a 100 min time frame. (B) Percentage of T4SS-deficient *X*. *citri* cells that lysed after cell-to-cell contact with *S*. *maltophilia* cells. (C) Percentage of *X*. *citri* cells that lysed after cell-to-cell contact with *S*. *maltophilia* cells (*left*) and % of *S*. *maltophilia* cells that lysed after cell-to-cell contact with *X*. *citri* cells (*right*). Cells were counted per interaction over a 300 min time frame. (D) Representative images of time-lapse microscopy showing *S*. *maltophilia* wild-type and Δ*virD4* strains interacting with the T4SS-deficient *X*. *citri* Δ*virB*-GFP strain at the single cell level. Dead/lysed *X*. *citri* Δ*virB*-GFP cells are indicated by white arrows. (E) *S*. *maltophilia* wild-type and Δ*virD4* strains interacting with the *X*. *citri*-GFP strain (functional T4SS) at the single cell level. Dead/lysed *X*. *citri* cells are indicated by white arrows and dead/lysed *S*. *maltophilia* cells are indicated by yellow arrows. Timestamps in hours:minutes. Scale bar 5 μm. Images were acquired every 15 min.

Single cell analysis by fluorescence microscopy of *S*. *maltophilia* co-incubated with *E*. *coli* expressing red fluorescent protein (*E*. *coli-*RFP) further confirms that *Stenotrophomonas* induces target cell lysis in a contact-dependent manner (Fig 1E and S1 Movie). No cell lysis was detected when *E*. *coli* was co-incubated with *S*. *maltophilia* Δ*virD4* (Fig 1E and S2 Movie). Quantification of *E*. *coli* cell lysis over a timeframe of 100 min shows that approximately 50% of *E*. *coli* cells in contact with wild-type *Stenotrophomonas* lysed during this period, while no *E*. *coli* cell lysis was detected when mixed with *S*. *maltophilia* Δ*virD4* (Fig 1F). It is important to note that during the time frame of these experiments some *E*. *coli* cells may be intoxicated without cellular lysis since the time of target-cell lysis may vary after the initial physical contact. Therefore, the quantification presented in Fig 1F most likely sub-estimates the efficiency of the T4SS mediated antagonistic effect. In addition to *E*. *coli*, we observed that *S*. *maltophilia* is able to kill other Gram-negative bacterial species such as *Klebsiella pneumoniae*, *Salmonella* Typhi and *Pseudomonas aeruginosa* in a T4SS-dependent manner (Fig 2A, S3, S4 and S5 Movies) while no killing was observed using the *S*. *maltophilia* Δ*virD4* strain (S15, S16 and S17 Movies). These results are consistent with our previous work in which we demonstrated that *X*. *citri* displays an antagonistic effect towards not only *E*. *coli*, but also *Chromobacterium violaceum* (Betaproteobacteria) in a T4SS-dependent manner [10].

As *X*. *citri* is, to date, the only other bacterial species experimentally shown to use a T4SS for interbacterial killing, we decided to analyse whether *S*. *maltophilia* and *X*. *citri* could use their T4SSs to compete with and kill each other. First, we co-incubated *S*. *maltophilia* (either wild-type or Δ*virD4)* with an *X*. *citri* T4SS mutant in which all of the chromosomal *virB* genes were substituted with the gene for green fluorescent protein (GFP) under the control of the endogenous *virB7* promoter (Δ*virB-*GFP) [43] and confirmed that *S*. *maltophilia* induces lysis of *X*. *citri* Δ*virB*-GFP in a T4SS-dependent manner (Fig 2B and 2D; S6 and S7 Movies). Next, we co-incubated *X*. *citri*-GFP (carrying a functional T4SS) with *S*. *maltophilia* wild-type or Δ*virD4* strains. Besides showing that *X*. *citri* can induce lysis of *S*. *maltophilia* Δ*virD4* (S8 Movie), we observed that when both wild-type species are mixed, they duel and kill each other in a T4SS-dependent manner (Fig 2C and 2E; S9 Movie). *S*. *maltophilia* seems to be slightly more effective in killing *X*. *citri* via its T4SS, which could be due to differences in the efficiencies of the systems, differences in their repertoires of effectors (see below) and/or the shorter doubling time of *S*. *maltophilia* compared to *X*. *citri* under the conditions tested.

### Identification of twelve putative effectors secreted via the *S*. *maltophilia* X-T4SS

**Fig 3:**
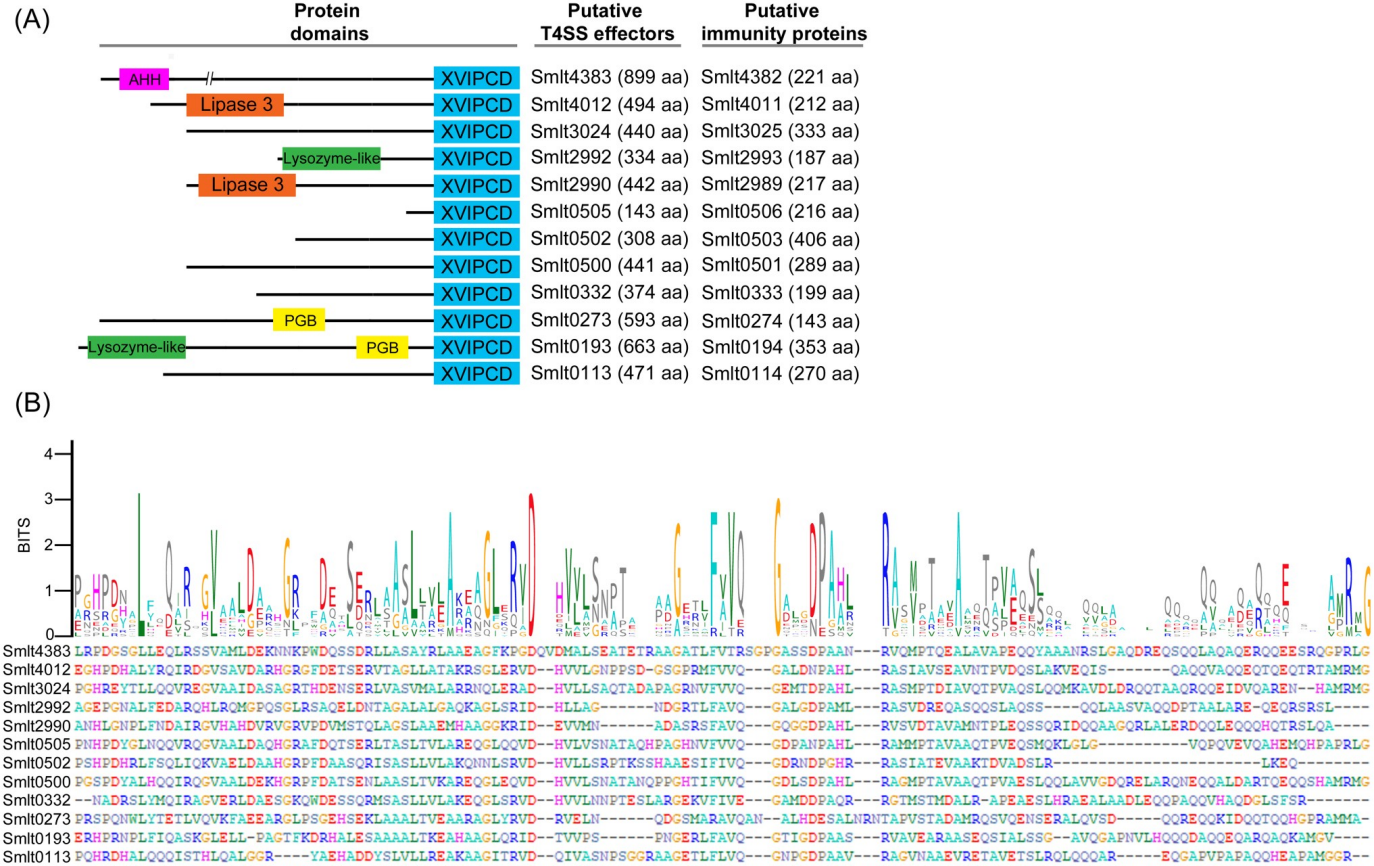
Putative X-T4SS effectors (X-Tfes) and immunity proteins (X-Tfis) of *S*. *maltophilia*. (A) Schematic representation of size and domain architectures of *S*. *maltophilia* X-T4SS effectors identified via BLASTp search using XVIPCD (*Xanthomonas* VirD4-interacting protein conserved domain) of *X*. *citri* T4SS effectors. Gene entries are shown for both effectors and their cognate immunity proteins with sizes shown in parenthesis. AHH: putative nuclease domain; PGB: peptidoglycan-binding domain. (B) Alignment of the XVIPCDs of the identified *S*. *maltophilia* effectors using Clustal Omega [85] and the consensus sequence logo generated by WebLogo [86] showing several highly conserved amino acids that match conserved residues of the *X*. *citri* XVIPCDs [30].

After confirming that the *S*. *maltophilia* X-T4SS is functional and induces target cell death, we decided to search for the effector proteins translocated by this system that were mediating the phenotype. As the VirD4 coupling protein of *X*. *citri* complements the Δ*virD4* strain of *S*. *maltophilia* (Fig 1B and 1D), we hypothesized that potential substrates secreted via the T4SS of *S*. *maltophilia* could be identified by applying a bioinformatic approach using the conserved C-terminal domains of *X*. *citri* X-Tfes (XVIPCD) that interact with VirD4 to search the genome of *S*. *maltophilia* K279a. Using this approach, we identified twelve *S*. *maltophilia* proteins as potential T4SS substrates (X-Tfes) (Fig 3A, S1 Table). Amino acid sequence alignment of C-terminal XVIPCDs from *Stenotrophomonas* X-Tfes revealed a series of conserved amino acid motifs that are also present in *X*. *citri* X-Tfes (Fig 3B) [30], highlighting putative key residues required for VirD4 recognition and secretion by these X-T4SSs.

All identified *S*. *maltophilia* effectors are organized in small operons together with an upstream gene encoding a conserved hypothetical protein, reminiscent of the organization of effectors with their immunity proteins [10, 44]. Six of the identified *S*. *maltophilia* T4SS substrates harbour domains already described in other bacterial toxins such as lipases, nucleases, lysozyme-like hydrolases and proteins with peptidoglycan binding domains (Fig 3A). Three of these effectors (*smlt2990*, *smlt2992* and *smlt3024*) are encoded by genes close to the *S*. *maltophilia virB* structural locus (genes *smlt2997* to *smlt3008*), further illustrating the link of these effectors with the T4SS. It is interesting to note that six of the identified putative *Stenotrophomonas* T4SS effectors do not display any known protein domain that could indicate the mechanism mediating antibacterial activity (*smlt0113*, *smlt0332*, *smlt0500*, *smlt0502*, *smlt0505*, *smlt3024*) (Fig 3A). To validate our bioinformatic results and obtain further insight regarding the function of the effectors with unknown function, we selected the products of the *smlt3024* gene and its upstream putatively co-transcribed cognate immunity protein (*smlt3025)* for further characterization.

### Smlt3024 reduces growth rate of target cells when directed to the periplasm and is neutralized by Smlt3025

**Fig 4:**
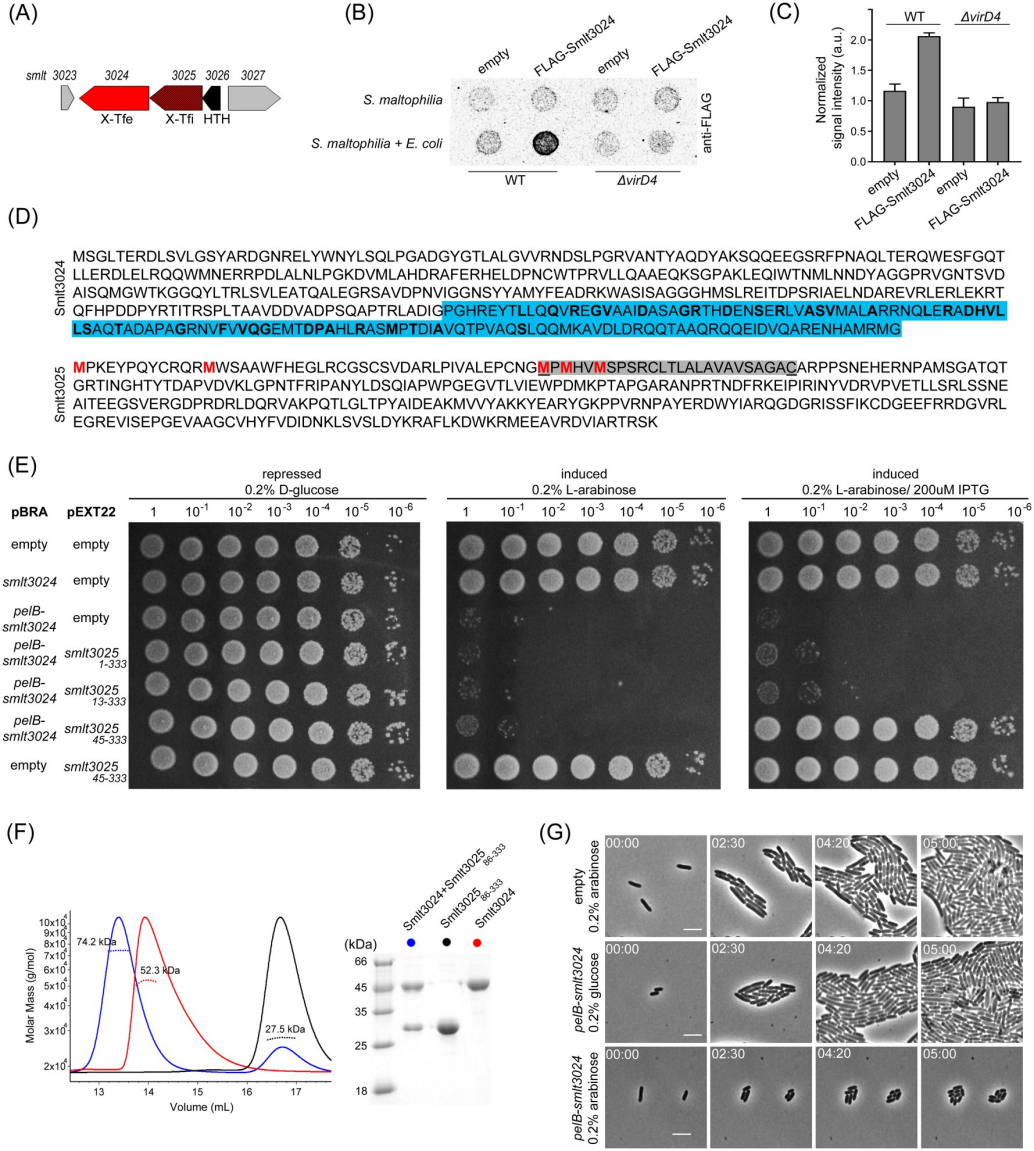
*S*. *maltophilia* Smlt3024 induces target cell stasis and Smlt3025 is its inhibitor. (A) Schematic representation of *smlt3024* and *smlt3025* genomic organization. (B) Immunoblot showing T4SS-dependent and *E*. *coli* contact-dependent secretion/translocation of FLAG-Smlt3024. Representative image of three independent experiments. (C) Densitometry of quantitative dot blot analysis signals shown in (B). Signal intensity detected for *S*. *maltophilia* mixed with *E*. *coli* were normalized by the background signal detected for *S*. *maltophilia* alone. (D) Amino acid sequence of Smlt3024 and Smlt3025 as annotated in *S*. *maltophilia* str. K279a genome (GenBank AM743169). Coloured in blue is the Smlt3024 XVIPCD with conserved amino acids in bold. Methionine (M) residues at positions 1, 13, 45, 47 and 50 of Smlt3025 are shown in red. The predicted periplasmic localization signal of Smlt3025 beginning at Met_45_ is shaded in grey with cleavage and lipidation predicted at the underlined cysteine. (E) Serial dilution (10-fold) of *E*. *coli* strains containing pBRA and pEXT22 constructs as indicated, spotted on LB-agar plates. Growth inhibition is observed upon expression of the *pelB-smlt3024* construct (periplasmic) and can be reverted by the concomitant expression of Smlt3025_45-333_ but not Smlt3025_1-333_ or Smlt3025_13-333_. (F) Left panel: SEC-MALS analysis showing the formation of a stable complex between Smlt3024 and Smlt3025_86-333_. The continuous line corresponds to the normalized differential refractive index, and the spotted lines indicate the calculated molecular mass. Right panel: SDS-PAGE showing the apparent molecular mass of proteins eluted from different SEC peaks. (G) Time-lapse imaging of single cells expressing pBRA-*pelB*-*smlt3024* showing reduced growth rates and smaller cell-sizes (L-arabinose) compared to the non-induced (D-glucose) and empty plasmid controls. Images were acquired every 10 min. Timestamps in hours:minutes. Scale bar 5 μm.

In its genomic context, *smlt3024* seems to be organized in an operon downstream of two genes encoding for its putative cognate immunity protein (*smlt3025*) and another small protein containing a helix-turn-helix (HTH) domain annotated as a putative transcriptional regulator (*smlt3026*) (Fig 4A). This operon, along with the putative operons coding for the effector/immunity pairs *smlt2990/smlt2989* and *smlt2992/smlt2993*, is in close proximity to the locus coding the X-T4SS structural genes (*smlt2997-smlt3008*, Fig 1A).

To determine whether Smlt3024 is indeed an effector secreted via the *S*. *maltophilia* T4SS, we cloned an N-terminal FLAG-tagged version of *smlt3024* (FLAG-Smlt3024) into the pBRA plasmid under the control of the P_BAD_ promoter and used it to transform both *S*. *maltophilia* wild-type and Δ*virD4* strains. These strains were co-incubated with *E*. *coli* and spotted onto nitrocellulose membranes placed over LB-agar plates containing 0.1% L-arabinose and incubated for 6 h at 30°C. The membranes were later processed for immunodetection with an anti-FLAG antibody. Results show an increase in signal intensity for FLAG-Smlt3024 when *S*. *maltophilia* was co-incubated with *E*. *coli* (Fig 4B and 4C), while no increase was detected when *S*. *maltophilia* Δ*virD4* was co-incubated with *E*. *coli* (Fig 4B and 4C). In addition, no increase in signal intensity could be detected when *S*. *maltophilia* FLAG-Smlt3024 was incubated without target *E*. *coli* cells (Fig 4B). SDS-PAGE of total protein extracts followed by western blot with anti-FLAG antibody showed that both *S*. *maltophilia* wild-type and Δ*virD4* strains were expressing similar levels of FLAG-Smlt3024 (S2 Fig). These results indicate that translocation of Smlt3024 is dependent on a functional T4SS and on contact with a target cell from another species. We interpret the anti-FLAG signal detected by western blot as due to *E*. *coli* cell lysis caused by the delivery of FLAG-Smlt3024 along with the full cocktail of *S*. *maltophilia* X-Tfes via the T4SS into the target *E*. *coli* cells. After target cell lysis, the released FLAG-Smlt3024 binds to the nitrocellulose membrane; hence the assay is an indirect measurement of protein translocation. Although we do not have direct experimental visualization of X-Tfe delivery into target cells, we note that all except for a few T4SSs described to date transfer macromolecules across the bacterial cell envelope directly into the target cell [45–47], so we hypothesize that X-T4SS toxic effectors are translocated directly into the target cell.

If Smlt3024 is indeed a toxic effector translocated by the *S*. *maltophilia* T4SS, then we would expect that its expression in the appropriate compartment within *E*. *coli* would cause an impairment of bacterial growth. To evaluate the toxicity of Smlt3024 upon expression in *E*. *coli* and to establish in which cellular compartment Smlt3024 exerts its effect, we cloned the full-length *smlt3024* gene into the pBRA vector placing it under control of the P_BAD_ promoter (inducible by L-arabinose and repressed by D-glucose) both with and without an N-terminal PelB periplasmic localization signal sequence. We also cloned the sequence of the putative Smlt3025 immunity protein into the pEXT22 vector placing it under the control of the P_TAC_ promoter, which can be induced by IPTG. We noted that the published annotated sequence for Smlt3025 [40] has a non-canonical GTG start codon with 4 possible in frame ATG start codons at positions 13, 45, 47 and 50 and that initiation at positions 45, 47 or 50 is predicted to produce proteins with an N-terminal signal sequence lipobox for periplasmic localization as a lipoprotein (Fig 4D) [48]. Therefore, three versions of Smlt3025 were cloned into pEXT22, leading to the production of Smlt3025_1-333_, Smlt3025_13-333_ and Smlt3025_45-333_. *E*. *coli* strains carrying the different combinations of pBRA-Smlt3024 and each one of the pEXT22-Smlt3025 plasmids were serial diluted and incubated on LB-agar plates containing either D-glucose, L-arabinose or L-arabinose plus IPTG (D-glucose inhibits and L-arabinose induces expression of Smlt3024; IPTG induces expression of Smlt3025). Results showed that Smlt3024 is toxic when directed to the periplasm of *E*. *coli* cells (pBRA-*pelB-smlt3024*) but not in the cytoplasm (pBRA-*smlt3024*), and that only Smlt3025_45-333_, which is predicted by the SignalP 5.0 algorithm to be directed to the periplasm [49], could neutralize Smlt3024 toxicity (Fig 4E). These results support the hypothesis that Smlt3025 was mistakenly annotated and that the correct start codon is Met_45_, Met_47_ or Met_50_. Bioinformatic analysis of the closest 100 homologues of Smlt3025 in the non-redundant protein database, shows that most proteins are annotated with initiation codons that align with Met_47_ of Smlt3025 (S3A Fig). Similar results are obtained when more distantly related Smlt3025 homologues from the KEGG database [50] are aligned (S3B Fig).

To gain some information about the inhibitory mechanism of Smlt3025, we decided to analyse whether this protein could interact directly with Smlt3024 by expressing and purifying full-length Smlt3024 and a soluble version of Smlt3025 (amino acid residues 86–333) lacking its predicted N-terminal signal peptide. Complex formation was analysed using size exclusion chromatography coupled to multiple-angle light scattering (SEC-MALS) (Fig 4F). The MALS analysis calculated average masses for Smlt3024 and Smlt3025_86-333_ of 52.3 kDa and 27.5 kDa, respectively, which are very close to the theoretical values of their monomer molecular masses of 49 kDa and 28 kDa, respectively (Fig 4F). When a mixture of these proteins was analysed by SEC-MALS followed by SDS-PAGE, a new peak was observed containing both Smlt3024 and Smlt3025_86-333_ with an estimated molecular mass calculated by MALS of 74.2 kDa, showing that a stable 1:1 complex (theoretical mass of 77 kDa) was formed between Smlt3024 and Smlt3025_86-333_ (Fig 4F).

To gather further insight on the mechanism by which Smlt3024 could induce toxicity, we decided to perform time-lapse microscopy to evaluate growth and morphology of individual *E*. *coli* cells carrying the empty pBRA or pBRA-*pelB-smlt3024* plasmids. *E*. *coli* carrying the empty plasmid incubated on LB-agar with 0.2% L-arabinose (Fig 4G and S10 Movie) as well as the repressed pBRA-*pelB-smlt3024* (0.2% D-glucose) grew normally (Fig 4G and S11 Movie). Upon induction with L-arabinose, cells carrying pBRA-*pelB-smlt3024* quickly experienced a strong reduction in growth rate and single cells were smaller (average length of 2.1 ± 0.7 μm after 300 min) compared to the controls incubated in glucose (average length of 3.6 ± 1.2 μm after 300 min) (Fig 4G and S12 Movie). Despite the severe delay in growth rate, *E*. *coli* cells expressing PelB-Smlt3024 remained viable and continued growing and dividing for up to 8 h (S12 Movie).

**Fig 5:**
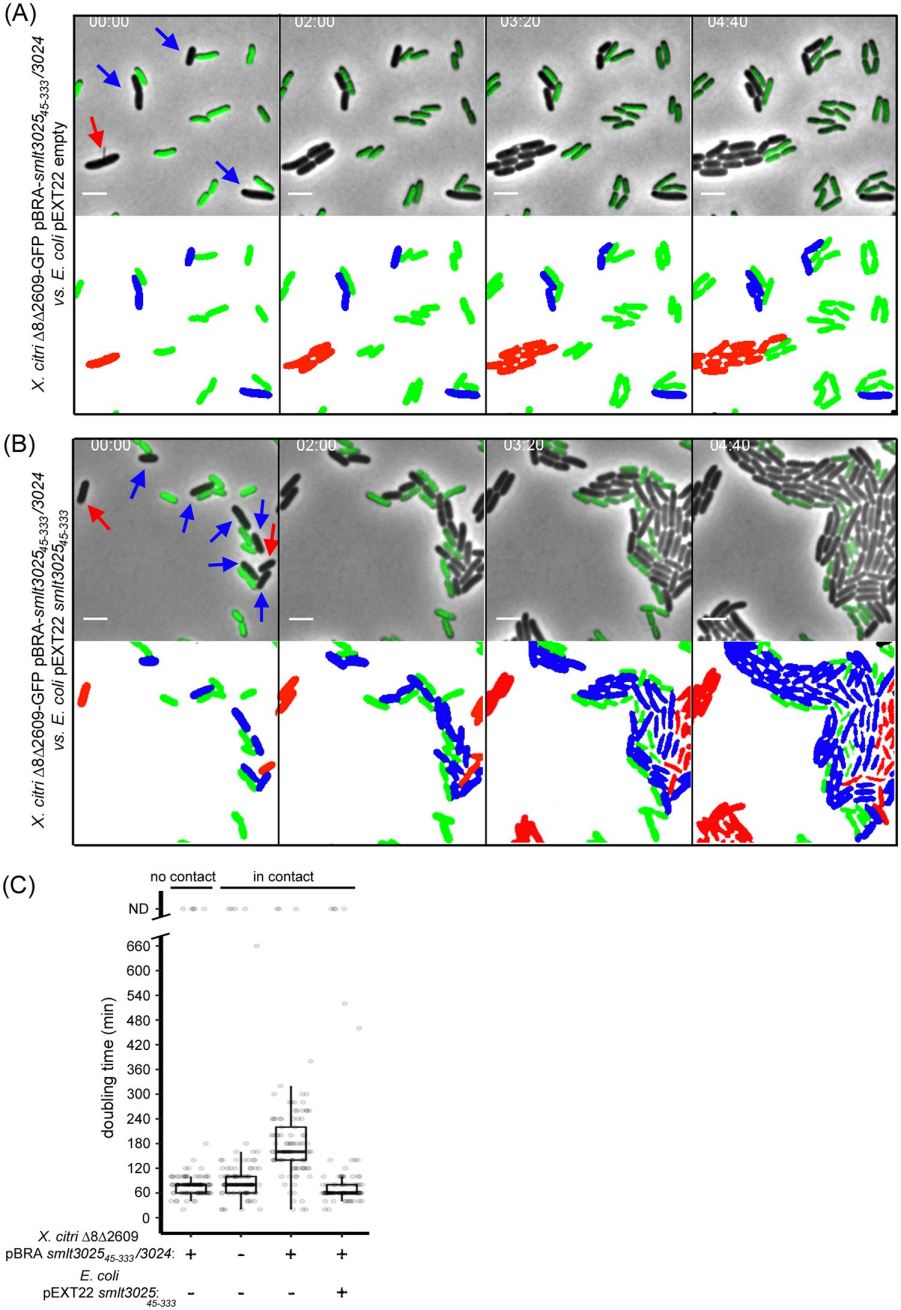
Smlt3024 delivery by *X*. *citri* reduces target *E*. *coli* cells growth speed. (A) Upper panel: Representative images of time-lapse microscopy showing *X*. *citri* Δ8Δ2609-GFP expressing pBRA-*smlt3025*_*45-333*_*/3024* in contact with *E*. *coli* cells carrying empty pEXT22 plasmid. Blue arrows indicate *E*. *coli* cells that are in contact with *X*. *citri* at time zero, while red arrows show *E*. *coli* cells that were not in contact. Lower panel: Schematic representation showing the growth of *E*. *coli* cells that were either in contact (blue) or not (red) with *X*. *citri* at time zero. (B) Upper panel: Representative images of time-lapse microscopy showing *X*. *citri* strain described in (A) in contact with *E*. *coli* cells expressing Smlt3025_45-333_. Red and blue arrows indicate *E*. *coli* cells as in (A). Lower panel: Schematic representation as in (A). Timestamps in hours:minutes. Scale bar 2 μm. Images were recorded every 20 min. (C) Quantitative analysis of the doubling time of *E*. *coli* cells either carrying empty plasmid or expressing Smlt3025_45-333_ when in contact or not in contact with *X*. *citri* Δ8Δ2609-GFP with or without the plasmid expressing the Smlt3024/Smlt3025 X-Tfe/X-Tfi pair. Strains were grown at 28°C. Boxplots represent means ± SD. ND represents cells tagged at time zero that did not divide and were not used to calculate the average doubling times.

In order to confirm that Smlt3024 produces the same phenotype when delivered by a bona fide X-T4SS into a target cell, we employed an *X*. *citri* strain (Δ8Δ2609-GFP) that has an intact and functional X-T4SS but is deficient in inducing target cell lysis due to the sequential deletion of nine X-Tfes genes (see Materials and methods). This strain allows phenotypic analysis of individual effectors without the interference of other lytic toxins. As the structural genes of *X*. *citri* and *S*. *maltophilia* T4SSs are very similar (Fig 1A) and expression of the VirD4 coupling protein of *X*. *citri* can restore the function of *S*. *maltophilia* Δ*virD4* (Fig 1B and 1D), we reasoned that *X*. *citri* Δ8Δ2609-GFP could be used to deliver *S*. *maltophilia* effectors. *X*. *citri* Δ8Δ2609-GFP was transformed with pBRA plasmid carrying the operon coding for Smlt3025 (starting from Met_45_) and Smlt3024. Time-lapse microscopy analysis of *X*. *citri* Δ8Δ2609-GFP and *E*. *coli* co-cultures grown on agar pads allowed us to measure the doubling times of *E*. *coli* cells (Fig 5, S13 and S14 Movies). The average doubling time of *E*. *coli* cells that were not in contact with *X*. *citri* Δ8Δ2609-GFP or were in contact with *X*. *citri* Δ8Δ2609-GFP carrying empty plasmid was 77 ± 23 and 92 ± 66 min, respectively (Fig 5C). However, the *E*. *coli* doubling time increased to 173 ± 71 min when in contact with *X*. *citri* Δ8Δ2609-GFP expressing Smlt3024 (Fig 5A and 5C). This growth inhibition effect could be reverted by expressing the immunity protein Smlt3025_45-333_ in target *E*. *coli* cells in which doubling times were restored to 78 ± 65 min (Fig 5B and 5C). These results confirm the inhibitory effect of Smlt3024 on cell growth upon translocation via a bona fide X-T4SS into target cells. Furthermore, these results demonstrate that X-Tfes from one species can be recognized for transfer by T4SSs from another species, thus highlighting the conservation of X-Tfe secretion signal recognition and X-T4SS function in *Stenotrophomonas* and *Xanthomonas* species.

### Smlt3024 is similar to the N-terminal domain of unknown function often found in proteins containing Ca^2+^-binding RTX motifs

To obtain some insight into the possible contribution of Smlt3024 to T4SS-dependent antagonism, we searched for homologues similar to its amino acid sequence (residues 1–308, excluding the C-terminal XVIPCD) using the PSI-BLAST algorithm [51] against the non-redundant protein sequence database. Three iterations of PSI-BLAST retrieved 815 hits (cutoff e-values < e^-6^). The first 402 hits with the highest scores are from shorter proteins of unknown function (less than 600 amino acids), which are about the same size of Smlt3024 (440 residues). The PSI-BLAST search also returned 221 hits with lower scores (e-values between e^-43^ and e^-7^) from larger proteins (greater than 750 amino acids in length) derived from a wide variety of bacterial genera including *Yersinia*, *Ralstonia*, *Pseudomonas*, *Cupriavidus*, *Snodgrassella*, *Xanthomonas*, *Pseudoxanthomonas*, *Leisingera*, *Thalassospira*, *Nitrosomonas*, *Halocynthiibacter*, *Vibrio*, *Neisseria*, *Thioalkalivibrio*, *Stenotrophomonas*, *Rhizobium*, *Robbsia*, *Devosia*, *Sphingomonas*, *Paraburkholderia*, *Sphingomonas* and *Acinetobacter*. This group of 221 proteins (S4 Fig) share the following characteristics: i) all except for one align with Smlt3024 via their N-terminal regions (within the first 300 amino acids) and ii) all but six have multiple Repeat in ToXin (RTX) calcium-binding nonapeptide motifs (Pfam: PF00353) [52] or carry hemolysin-type calcium binding protein related domains (Pfam: PF06594). Some also have additional C-terminal domains such as peptidase S8, subtilisin-like, pro-protein convertase P, cadherin-like and IgG-like domains. An analogous search using the JACKHMMER algorithm [53] against the rp75 database produced similar results (S2 Table). Thus, both PSI-BLAST and JACKHMMER searches indicate that Smlt3024 is similar to the N-terminal domain of unknown function often found in larger proteins with downstream Ca^2+^-binding RTX motifs. One notable exception to the above pattern is the alignment of Smlt3024 with the C-terminal domain of a type VI secretion system tip protein VgrG from *Sphingomonas jatrophae* strain S5-249 (accession number WP_093316205.1), whose possible significance will be considered in the Discussion.

### The Smlt3025 crystal structure presents a topology similar to the iron-regulated protein FrpD of *Neisseria meningitidis*

**Fig 6:**
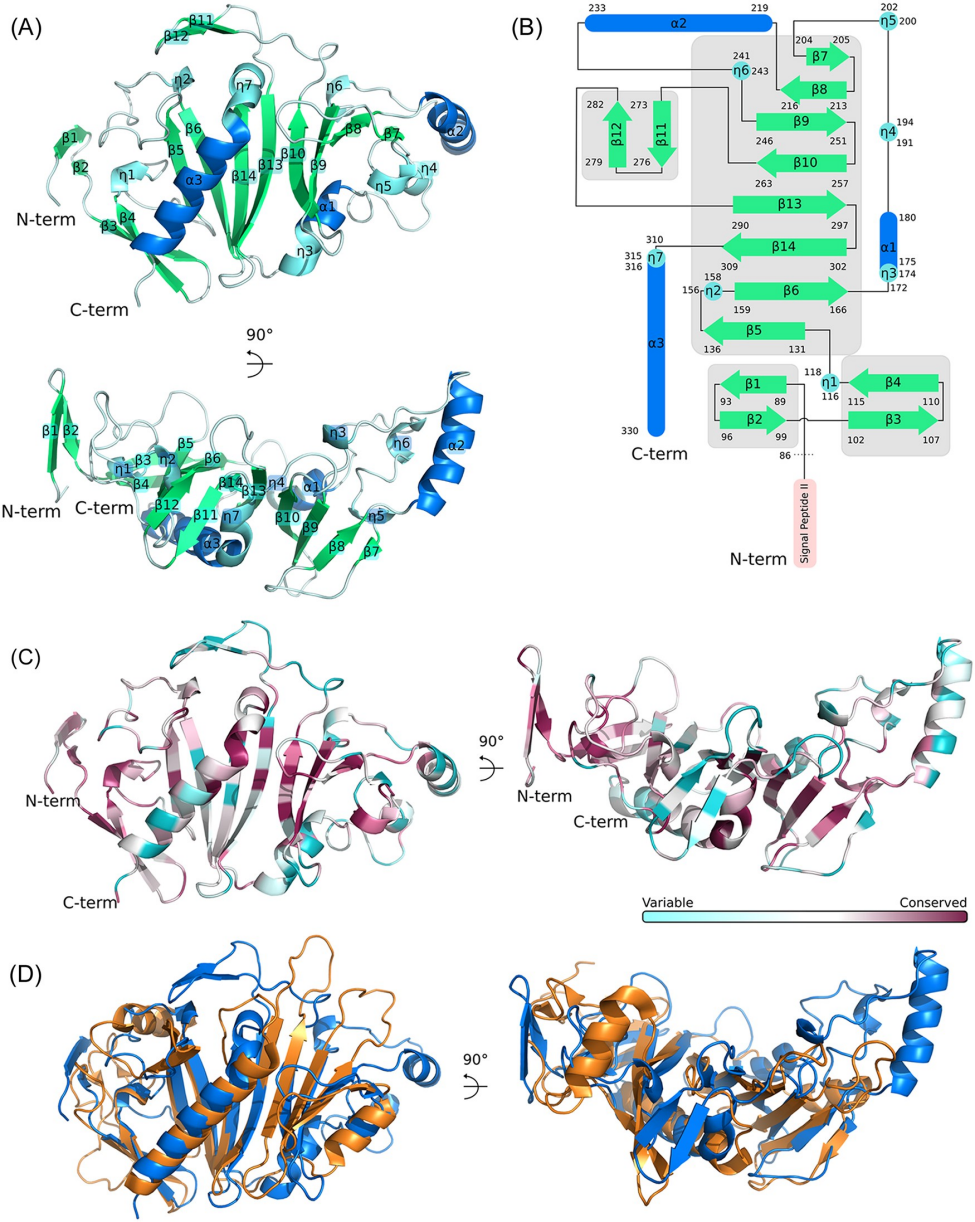
Smlt3025 crystal structure presents a topology similar to the iron-regulated protein FrpD of *Neisseria meningitidis*. (A) Ribbon representation of the Smlt3025_86-333_ structure (PDB 6PDK). The protein has two N-terminal beta hairpins followed by an 8-stranded antiparallel central beta sheet and a C-terminal alpha helix. Some of the loops between beta strands contain additional secondary structure elements. (B) Scheme illustrating Smlt3025 topology. In both (A) and (B), beta strands, 3_10_ helix and alpha helices are colored green, light blue and marine, respectively. (C) Sequence conservation of Smlt3025 homologues mapped onto the Smlt3025 structure. Coloring generated by Consurf [87]. (D) Structural alignment between Smlt3025_86-333_ (blue) and FrpD (orange, PDB 5EDF). Molecular orientations in (A), (C) and (D) are the same.

In order to obtain more information regarding the mechanism of the effector/immunity pair Smlt3024/Smlt3025 we tried to crystalize these proteins to solve their structures by X-ray crystallography. We successfully crystallized a soluble fragment of Smlt3025, corresponding to residues 86–333. Crystals belonged to space group R3, some of which diffracted to around 2 Å resolution. Initial phases were estimated by single wavelength anomalous dispersion using a crystal soaked in sodium iodide and the final model was obtained using data collected from a native crystal (Table 1). The Smlt3025 structure (PDB 6PDK) is organized around a central 8-stranded anti-parallel β sheet (β5-β6-β14-β13-β10-β9-β8-β7). The intervening loops between these β-strands contain α-helices (α1 and α2), 3_10_ helices (η2, η3, η4, η5 and η6) and a small beta-hairpin (β11-β12). The central β-sheet is preceded by two β-hairpins (β1-β2, β3-β4) and a 3_10_ helix (η1) and is followed by a C-terminal helix (η7 and α3; Fig 6A and 6B). An analysis of Smlt3025 homologues using the Consurf algorithm (S5 Fig) identified conserved positions which, once mapped onto the Smlt3025 structure, cluster into the hydrophobic core of the central β-sheet and to the N-terminal β-hairpins (Fig 6C).

**Table 1:** Data collection and refinement statistics for Smlt3025_86-333_.

Data Collection	Native Smlt3025	NaI-derivative Smlt3025
Wavelength (A)	1.5419	1.5419
Space group	R3	R3
Unit cell (A)	67.5, 67.5, 149.4 90.0°, 90.0°, 120.0°	67.0, 67.0, 149.6, 90.0°, 90.0°, 120.0°
Resolution range (A)	46.05–1.90 (1.91–1.90)*	45.81–2.09 (2.11–2.09)*
Total reflections	95110 (11518)*	147582 (23137)*
Unique reflections	19897 (3140)*	29281 (4705)*
Completeness (%)	99.4 (96.4)*	99.8 (99.1)*
Redundancy	4.8 (3.7)*	5.0 (4.9)*
I/sigma (I)	14.3 (2.6)*	15.6 (2.0)*
R meas (%)	6.7 (43.2)*	6.7 (74.9)*
**Refinement**		
R_work_ / R_free_	0.195 / 0.248	
Number of atoms		
Protein	1999	
Water	447	
RMSD		
Bonds (A)	0.007	
Angles (°)	0.933	
Ramachandran (%)		
Favorable	98.78	
Allowed	1.22	
Outliers	0.00	

* Data shown in parentheses refers to the highest-resolution shell.

Smlt3025 has no significant amino acid sequence similarity with proteins with known 3D structure. Structure-based similarity searches using the DALI algorithm [54] identified a single protein with a Z-score of 9.3, named iron-regulated protein D (FrpD) from *Neisseria meningitidis* (PDB 5EDF). FrpD is a lipoprotein associated with the *N*. *meningitidis* outer membrane that strongly interacts with the N-terminal domain of iron-regulated protein C (FrpC), a 1829 residue protein secreted into the extracellular milieu via a T1SS [55, 56]. FrpC belongs to the RTX protein family, with 43 C-terminal RTX motifs [57], an architecture very similar to most of the 221 proteins identified as Smlt3024 homologues in S4 Fig.

Fig 6D presents a structural alignment between Smlt3025_86-333_ and FrpD. The topologies of the central β-sheets of the two proteins are identical. However, the loops connecting the β-strands have significant differences, for example α2 and the β11-β12 hairpin in Smlt3025 are absent in FrpD. Previous NMR chemical shift perturbation studies identified the surface-exposed portions of the N-terminal β strands (preceding the central β sheet), the C-terminal portion of the last α helix and the unstructured C-terminal tail of FrpD as the probable binding site for FrpC [55, 56]. Although the corresponding surface of Smlt3025 has significantly different structural features at the N-terminus, due to a different relative orientation of its β1-β2 hairpin, the C-terminal α helices and the β3-β4 hairpins of the two proteins superpose well (Fig 6D) and the N-terminal β hairpins are amongst the most well conserved sequences in Smlt3025 homologues (Fig 6C and S5 Fig). These observations raise the hypothesis that Smlt3025 could interact with Smlt3024 in a manner analogous to the FrpD-FrpC interaction.

## Discussion

Competition between microorganisms for nutrients and space often determines which species will thrive and dominate or be eradicated from a specific habitat. *S*. *maltophilia* is often found as a member of microbial communities in water, soil and in association with plants. Some *Stenotrophomonas* species like *S*. *rhizophila* can participate in beneficial interactions with plants, but no species were reported to be phytopathogenic, which distinguishes *Stenotrophomonas* from the phylogenetically related genera *Xanthomonas* and *Xylella* [32]. More importantly, an increasing number of hospital-acquired *S*. *maltophilia* infections over the last decades has led to the classification of this bacterium as an emerging opportunistic pathogen [33, 34]. Key to the opportunistic behaviour of *S*. *maltophilia* strains are their ability to form biofilms and their resistance to multiple antibiotics. In this manuscript, we show that the X-T4SS of *S*. *maltophilia* is involved in interbacterial competition, allowing *S*. *maltophilia* to induce lysis of several Gram-negative species. The antibacterial property conferred by the X-T4SS probably provides a competitive advantage to *S*. *maltophilia* in polymicrobial communities, contributing to increased fitness. *S*. *maltophilia* is frequently associated with cystic fibrosis patients [58, 59] and may need to compete with oral and nasal microbiota during infection of susceptible organisms [60, 61]. Our competition experiments showed that *S*. *maltophilia* can kill two pathogens that colonize the respiratory tract of susceptible hosts, *K*. *pneumoniae* and *P*. *aeruginosa*; hence the contribution of *S*. *maltophilia* T4SS to colonization and maintenance during polymicrobial infections within mammalian hosts merits further investigation.

The most worrying aspect of pathogenic *S*. *maltophilia* strains is their multi-drug resistance phenotype [62]. As *S*. *maltophilia* is naturally competent to acquire foreign DNA [32, 39], the T4SS described here could, by inducing target cell lysis and increasing the availability of foreign DNA, be a positive factor in promoting *Stenotrophomonas* transformation, thus leading to the acquisition of antibiotic resistance genes by horizontal gene transfer. A similar mechanism has already been reported in *Vibrio cholerae*, which uses a bacterial killing T6SS as a predatory device to induce target cell lysis concomitantly with the uptake of target-cell DNA [63].

The *S*. *maltophilia* X-T4SS is homologous to the *X*. *citri* X-T4SS and complementation of *S*. *maltophilia* Δ*virD4* with the *X*. *citri* VirD4 coupling protein restored its full capacity to lyse *E*. *coli* target cells. The VirD4 coupling protein interacts with the conserved C-terminal domain (XVIPCD) of X-Tfes described in *X*. *citri* [10, 30]; thus it was reasonable to use these conserved regions to search the genome of *S*. *maltophilia* for new T4SS effectors. The search rationale proved to be efficient and we identified 12 new putative *S*. *maltophilia* T4SS effectors and provided experimental evidence that at least one of them (Smlt3024) is secreted in a T4SS-dependent manner. Due to the conservation of the amino acid sequence of XVIPCD of *S*. *maltophilia* T4SS effectors, it is likely that the other 11 putative effectors are also secreted via the T4SS. Translocation of Smlt3024 from the killing-deficient strain *X*. *citri* Δ8Δ2609-GFP into target *E*. *coli* cells also illustrates the conserved function of both T4SSs systems and confirms the ability of *X*. *citri* VirD4 coupling protein to recognize and translocate *S*. *maltophilia* effectors by means of their conserved XVIPCDs. Furthermore, recognition and translocation of *S*. *maltophilia* effectors by the *X*. *citri* T4SS machinery suggests that toxic effectors containing an XVIPCD could be easily exchanged between species in the environment by horizontal gene transfer of effector/immunity protein pairs.

Among the twelve *S*. *maltophilia* effector/immunity protein (X-Tfe/X-Tfi) pairs, we believe that special attention should be given to effectors with no recognizable domain annotated in Pfam database—six effectors including Smlt3024. Detailed biochemical and structural characterization of these new effectors could identify new toxic domains and might reveal interesting mechanisms impairing bacteria proliferation, contributing to the design of novel and effective antibacterial drugs. Most of the characterized T4SS and T6SS antibacterial toxins are enzymes that degrade structural cellular components such as peptidoglycan and phospholipids, thus promoting target cell lysis [64]. Recent studies have identified effectors that change cell metabolism, promoting altered cell growth rather than lysis, but these effectors act in the target cell cytoplasm [65, 66]. In this context, the mechanism underlying the apparent periplasmic toxicity induced by Smlt3024, which reduces target *E*. *coli* cell growth rate either by ectopic expression or after translocation by *X*. *citri* T4SS, is likely to be a mechanism not yet described.

According to our bioinformatic analyses, Smlt3024 presents homology with the N-terminal region of proteins that contain multiple RTX motifs (annotated as RTX toxins or hemolysin-type calcium binding proteins). However, no functional information is available for these N-terminal regions. The crystal structure of Smlt3025 revealed a topology similar to FrpD from *N*. *meningitidis*, which is a lipoprotein [55] that is known to bind the N-terminal region of FrpC, an 1829 residue protein that contains 43 RTX repeats between residues 879 and 1705 [57]. Upon secretion by the T1SS, FrpC undergoes Ca^2+^-dependent trans-splicing via autocatalytic cleavage between Asp_414_ and Pro_415_ to form an Asp_414_-Lys isopeptide bond, which results in covalent linkage of the FrpC_1-414_ fragment to plasma membrane proteins of epithelial cells *in vitro* [56]. FrpC was originally proposed to play a role during infection of mammalian hosts; however, subsequent studies analyzing FrpC cytotoxicity towards macrophages *in vitro* and infection of mammalian hosts with mutant strains failed to detect any cytotoxic effect or virulence attenuation [67]. Considering these findings, we hypothesize that FrpC may in fact be an *N*. *meningitidis* T1SS antibacterial effector and FrpD its cognate immunity protein.

The mechanism by which Smlt3024 causes reduction of growth speed after heterologous expression or T4SS-mediated translocation into the periplasm of the target cell is still unknown. Based on the similarity with the N-terminus of RTX proteins, we speculate that Smlt3024 could bind to and inhibit the function of one or more key metabolic or signal transduction components in the periplasm, thus promoting target cell stasis. Inducing target cell stasis could be sufficient in natural scenarios to provide the attacker with a competitive advantage, allowing it to outnumber the target species and establish itself in the environment. It is worth mentioning that Smlt3024 is homologous to Smlt0500, another *S*. *maltophilia* X-Tfe (48% identity over the first 308 residues), as are their cognate X-Tfis, Smlt3025 and Smlt0501 (41% identity; both predicted to be lipoproteins). Therefore, Smlt3024 and Smlt0500 could exert their functions via similar mechanisms and it is possible that their combined action could be more detrimental.

In natural settings, many species are likely to have acquired resistance mechanisms against some effectors by means of immunity proteins. Thus, by employing a cocktail of diversified effectors, species deploying an X-T4SS can gain an advantage over competitors. The importance of employing diversified effector-immunity pairs is illustrated by the duelling observed between *S*. *maltophilia* and *X*. *citri*: these species can kill one another in a T4SS-dependent manner, indicating that each lack immunity proteins against at least a subset of the rival´s set of T4SS effectors. Both *S*. *maltophilia* K279a and *X*. *citri* 306 carry twelve putative X-Tfe/X-Tfi pairs, but only six of the X-Tfis have homologues with 26–58% identity over segments that vary in size from 99 residues to 265 residues (S3 Table). Hence, these two bacteria could potentially be protected against some homologous cognate X-Tfes from the rival species. However, *S*. *maltophilia* is probably susceptible to the action of *X*. *citri* X-Tfes XAC4264 (unknown function), XAC2885 (putative fosfolipase), XAC2609 (peptidoglycan hydrolase), XAC1918 (putative peptidoglycan hydrolase), XAC0096 (putative HExxH metallopeptidase) and XAC0151 (unknown function) [10]. Likewise, *X*. *citri* can be expected to be susceptible to the action of the *S*. *maltophilia* X-Tfes for which it apparently has no immunity proteins: Smlt3024, Smlt0505, Smlt0502, Smlt0500, Smlt0332 and Smlt0273, all of unknown function (Fig 3A).

The above considerations stress the importance of our observations showing that X-Tfes from one organism can be employed by the X-T4SS from another. Therefore, the acquisition by horizontal gene transfer of genes encoding X-Tfe/X-Tfi pairs could be relevant in determining the outcome of encounters between environmental bacteria from the Xanthomonadales order. In addition to Smlt3024 similarity to the N-terminus of a large number of RTX proteins that are often secreted via a type I secretion system [52], one interesting exception is its similarity with the C-terminal region of VgrG from *Sphingomonas jatrophae* (S2 Table and S4 Fig). VgrG is a secreted component of T6SSs that either interacts with toxic effectors to promote their secretion or itself carries a toxic domain at its C-terminal region [68]. An analogous observation has been made for the *S*. *maltophilia* X-Tfe Smlt0332, which is homologous to the C-terminal domains of several VgrG proteins [11]. These observations illustrate the dynamic exchange of effector/toxin domains, not just between bacteria employing similar secretion systems but also their recombination with diverse recognition motifs employed by evolutionarily distinct secretion systems.

This work expands our current knowledge about the function of bacteria-killing T4SSs by increasing the panel of effectors known to be involved in X-T4SS-mediated interbacterial competition and by highlighting the possibility of interspecies exchangeability of X-Tfes, which is dependent on XVIPCD recognition by the VirD4 coupling protein. In addition, the study adds information about the mechanisms *S*. *maltophilia* has at its disposal to compete with other species, possibly contributing to its establishment in both clinical and environmental settings.

## Materials and methods

### Bacterial strains and culture conditions

*S*. *maltophilia* K279a [40] and *X*. *citri pv*. *citri* 306 [69] were grown in 2x YT media (16 g/L tryptone, 10 g/L yeast extract, 5 g/L NaCl). *E*. *coli* strain K-12 subsp. MG1655 [70] was used in competition assays because of its endogenous expression of β-galactosidase. *K*. *pneumoniae*, *S*. Typhi (ATCC 19430) and *P*. *aeruginosa* (PA14) were used for competition experiments. *E*. *coli* DH5α and *E*. *coli* HST08 were used for cloning purposes and *E*. *coli* S17 was used for conjugation with *S*. *maltophilia*. The *X*. *citri* Δ*virB*-GFP strain lacks all chromosomal *virB* genes and has the *msfGFP* gene under the control of the endogenous *virB7* promoter, while the *X*. *citri-*GFP strain has a functional T4SS and expresses GFP as a transcriptional fusion under the control of the *virB7* promoter [43]. For time-lapse imaging of *S*. *maltophilia* and *X*. *citri* strains, AB defined media was used (0.2% (NH_4_)_2_SO_4_, 0.6% Na_2_HPO_4_, 0.3% KH_2_PO_4_, 0.3% NaCl, 0.1 mM CaCl_2_, 1 mM MgCl_2_, 3 μM FeCl_3_) supplemented with 0.2% sucrose, 0.2% casamino acids, 10 μg/mL thiamine and 25 μg/mL uracil. Cultures of *E*. *coli* and *S*. *maltophilia* were grown at 37°C with agitation (200 rpm) and *X*. *citri* cultures were grown at 28°C with agitation (200 rpm). Antibiotics were used at the following concentrations to select *S*. *maltophilia* strains: tetracycline 40 μg/mL and streptomycin 150 μg/mL. For selection of *E*. *coli* strains, kanamycin 50 μg/ml and spectinomycin 100 μg/ml were used when appropriate. For induction from the P_BAD_ promoter, 0.2% L-arabinose was added. For P_TAC_ induction, 200 μM IPTG was used. Expression from both promoters was repressed using 0.2% D-glucose.

### Cloning and mutagenesis

All primers and plasmids used for cloning are listed in S4 Table. To produce in-frame deletions of *virD4* (*smlt3008*) in *S*. *maltophilia*, we used a two-step integration/excision exchange process and the pEX18Tc vector [71]. Fragments of ~1000-bp homologous to the upstream and downstream regions of *smlt3008* were amplified by PCR and cloned into pEX18Tc using standard restriction digestion and ligation. The pEX18Tc-Δ*virD4* was transformed in *E*. *coli* S17 donor cells by electroporation and transferred to *S*. *maltophilia* recipients via conjugation following the protocol described by Welker et al. [72]. Tetracycline-resistant colonies were first selected. Colonies were then grown in 2x YT without antibiotic and plated on 2x YT agar containing 10% sucrose without antibiotic. Mutant clones were confirmed by PCR. To complement the Δ*virD4* strain, the gene encoding full-length *smlt3008* was PCR amplified from genomic DNA and cloned into the pBRA vector, which is a pBAD24-derived vector that promotes low constitutive expression in *Stenotrophomonas* and *Xanthomonas* under non-inducing conditions. The pBRA construct encoding full-length *X*. *citri virD4*/*XAC2623* was reported previously [10]. For indirect secretion/translocation assays, the full-length sequence of *smlt3024* was cloned into pBRA vector, including a FLAG tag at its N-terminus and transformed into *S*. *maltophilia* wild-type and Δ*virD4*. Plasmids were transformed into *S*. *maltophilia* by electroporation (2.5 kV, 200 Ω, 25 μF, 0.2 cm cuvettes), followed by streptomycin selection. For cloning *smlt3024* and *smlt3025* into pSUMO–a modified version of pET28a (Novagen), with a SUMO tag between the hexahistidine and the cloning site–we used the soluble portion of Smlt3025 (residues between 86–333) that lacks the N-terminal signal peptide and the full-length Smlt3024. Smlt3025_86-333_ was also cloned into pET28a in order to express the protein with an N-terminal 6xHis tag that was subsequently crystallized (see below). To produce *smlt3024* with the *pelB* periplasmic localization sequence, PCR products were first cloned in pET22b (Novagen; containing the N-terminal *pelB* sequence). The *pelB-smlt3024* construct was subsequently transferred to pBRA using Gibson assembly. For the immunity protein *smlt3025*, three different constructs were cloned in pEXT22 [73]: one starting at the annotated GTG start-codon and two starting at two downstream ATG codons (Met_13_ and Met_45_). The sequences of all constructs containing effectors in pBRA and immunity proteins in pEXT22 were confirmed by DNA sequencing to assure absence of point mutations in the cloned genes and upstream promoter sites using the Macrogen standard sequencing service (https://dna.macrogen.com/). The *X*. *citri* Δ8Δ2609-GFP strain was constructed by sequentially deleting the genes coding for X-Tfe/X-Tfi pairs (except for the XAC2610 X-Tfi) from the *X*. *citri* genome [10, 11, 30] using the two-step allelic exchange procedure described above (Oka et al., in preparation). This strain has a total of nine deletions which were introduced in the following order: 1) Δ*XAC2885/XAC2884*; 2) Δ*XAC0574/XAC0573*; 3) Δ*XAC0097/XAC0096;* 4) Δ*XAC3364/XAC3363;* 5) Δ*XAC1918/XAC1917*; 6) Δ*XAC0467/XAC0466*; 7) Δ*XAC4264/XAC4263/XAC0462;* 8) Δ*XAC2609*::*msfGFP*; 9) Δ*XAC3266/XAC3267*. For the 8th deletion, the *xac2609* gene was replaced with the msfGFP gene, which allows the strain to be distinguished from target cells during time-lapse fluorescence microscopy.

### Bacterial competition assays

Bacterial competition was assessed either by analysing target cell growth or target cell lysis. To analyse *E*. *coli* growth during co-incubation with *S*. *maltophilia* we used a protocol adapted from Hachani et al. [74]. Briefly, strains were subcultured (1:100 dilution) and grown to exponential phase for 2 h at 37°C (200 rpm). Cells were washed with 2x YT, the optical density measured at 600 nm (OD_600nm_) and adjusted to 1. Serial dilutions (1:4) of *E*. *coli* culture was performed in 96 well plates. Equal volumes of *E*. *coli* and *S*. *maltophilia* cultures at OD_600nm_ 1.0 were mixed into each well. After mixing, 5 μl were spotted onto LB-agar plates containing 100 μM IPTG (isopropyl β-D-1-thiogalactopyranoside) and 40 μg/mL X-gal (5-bromo-4-chloro-3-indolyl-β-D-galactopyranoside) using multichannel pipettes. Plates were incubated for 24 h at 30°C. Competitions in solid and liquid media were performed as described previously [75]. Analysis of target cell death was performed using CPRG (chlorophenol red-β-D-galactopyranoside) as described previously with minor modifications [26, 42]. Briefly, *S*. *maltophilia* and *E*. *coli* overnight cultures were subcultured by 1:100 dilution and grown at 37°C (200 rpm) to reach OD_600nm_ of approximately 1 (*E*. *coli* cultures contained 200 μM IPTG). Cells were washed with LB media, OD_600nm_ adjusted to 1.0 for *S*. *maltophilia* strains and OD_600nm_ adjusted to 8.0 for *E*. *coli*. The adjusted cultures were mixed 1:1 and 10 μL spotted in triplicate onto 96 well plates containing 100 μL of semi-solid 1.5% 2x YT agar and 40 μg/mL CPRG. Plates were let dry completely, covered with adhesive seals and analysed on a SpectraMax Microplate Reader (Molecular Devices) at 572 nm every 10 min for 3.5 h. *E*. *coli* cultures were also spotted onto the same plate as a control for spontaneous cell death. The obtained A_572_ data was processed using RStudio (www.rstudio.com) and plotted using the ggplot2 package [76]. Background intensities obtained from the mean A_572_ values containing only *E*. *coli* cells were subtracted from all data series. The initial A_572_ value at time-point 0 min was subtracted from all subsequent time-points to correct for small differences in initial measurements. Finally, the *E*. *coli* lysis curves of *S*. *maltophilia* Δ*virD4* and complementation strains were normalized with respect to those obtained for the *S*. *maltophilia* wild-type strain.

### Time-lapse microscopy

For time-lapse imaging of bacterial killing at the single-cell level, agar slabs containing either 2x YT or supplemented AB media were created by cutting a rectangular frame out of a double-sided adhesive tape (3M VHB transparent, 24 mm wide, 1 mm thick), which was taped onto a first microscopy slide. Into the resulting tray, agar was poured and covered by a second microscopy glass slide to create a smooth surface. After solidification, the second microscopy slide was removed, exposing the agar’s surface onto which 2 μl of cell suspensions were spotted. After cell suspensions were left to dry completely, a #1.5 cover glass (Corning) was laid on top of the agar slab and closed at the sides by the second adhesive layer of the tape, leaving the cell mixtures closely and stably pressed between cover glass and the agar slab. Soon after, phase contrast images together with GFP or RFP excitation images were obtained with a Leica DMI-8+ epi-fluorescent microscope equipped with a Leica DFC365 FX camera, a HC PL APO 100x/1.4 Oil ph3 objective (Leica), a GFP excitation-emission band-pass filter cube (Ex: 470/40, DC: 495, EM: 525/50; Leica) and a Cy3/Rhodamine excitation-emission band-pass filter cube (Ex: 541/51, DC: 560, EM: 565/605; Leica). An incubation cage around the microscope kept temperatures constant at 37°C for *E*. *coli* and *S*. *maltophilia* experiments and at 28°C for experiments with *X*. *citri*. Several separate positions of each cell mixture were imaged every 10–15 min after auto-focusing using the LASX software package (Leica). Images were further processed with the FIJI software using the Bio-Formats plugin [77]. Time-lapse images were visually scored for cell lysis events. Small groups of cells (approximately 2 to 8 cells per colony) containing a mixture of bacterial species in close contact with each other were tagged at time-point zero and followed during 100 min (*E*. *coli* vs *S*. *maltophilia* competitions) or 300 min (*X*. *citri* vs *S*. *maltophilia* competitions) and cell lysis events were manually registered. Approximately 100 cells were scored for each assay. Quantification of *K*. *pneumoniae*, *S*. Typhi and *P*. *aeruginosa* killing by *S*. *maltophilia* was performed as described for *E*. *coli*.

For time-lapse imaging of the effect of Smlt3024 delivery into *E*. *coli* cells, the *X*. *citri* Δ8Δ2609-GFP strain expressing Smlt3025/3024 and *E*. *coli* containing the pEXT22-derived constructs expressing Smlt3025 were grown overnight in AB media supplemented with antibiotics. *E*. *coli* cells were diluted 100-fold in the same media with 200μM IPTG and grown for an additional 6 h to induce Smlt3025 production from the P_TAC_ promoter. No induction of Smlt3025/3024 expression in *X*. *citri* is required due to leaky expression from the P_BAD_ promoter. Before imaging, cells were pelleted and resuspended in AB medium with 0.2% sucrose and 0.2% casamino acids to remove antibiotics, diluted and mixed. To quantify *E*. *coli* doubling times, single cells in close contact with *X*. *citri* cells at time 0 were marked and followed through time. When mother and daughter cells showed clear separation of the division septa, the time of division was recorded. If either mother or daughter cell were still in contact with *X*. *citri* cells after division, subsequent division events of these cells were also counted. Cells that did not divide during the recorded time-lapse movie were not included in the calculations (ND in Fig 5). Doubling times of *E*. *coli* cells in the vicinity of but not in contact with *X*. *citri* expressing Smlt3024 were also recorded. For each condition, on average 100 cells were tracked. Since measurements started at time 0, independently of the cell-cycle of each marked cell at this time, and each frame of the time-lapse was taken every 20 min, the recorded values provide only a rough estimate of the true doubling times.

### BLASTp searches

To identify putative effectors secreted by the *S*. *maltophilia* T4SS, we used the XVIPCDs of known and putative *X*. *citri* T4SS substrates (residues in parenthesis): *XAC4264*(140–279), *XAC3634*(189–306), *XAC3266*(735–861), *XAC2885*(271–395), *XAC2609*(315–431), *XAC1918*(477–606), *XAC1165*(1–112), *XAC0574*(317–440), *XAC0466*(488–584), *XAC0323*(16–136), *XAC0151*(120–254), *XAC0096*(506–646) [10, 30] to BLAST search the genome of *S*. *maltophilia* K279a (https://www.genome.jp/tools/blast/). A list of *S*. *maltophilia* proteins identified by each *X*. *citri* XVIPCD with their respective E-values is shown in S1 Table.

### Recombinant protein expression, purification and SEC-MALS analysis

Smlt3025_86-333_ and full-length Smlt3024 cloned into pSUMO or pET28a, were transformed into *E*. *coli* BL21(DE3) and SHuffle T7 competent *E*. *coli* cells (New England BioLabs), respectively, and subcultured into 2x YT medium supplemented with 50 μg/mL kanamycin at 37°C until OD_600nm_ of 0.6 and then shifted to 18°C. After 30 min, protein production was induced with 0.1 mM IPTG. After overnight expression, cells were harvested by centrifugation and resuspended in 20 mM Tris-HCl (pH 8.0), 200 mM NaCl, 5 mM imidazole and lysed by 10 passages in a French Press system. The lysate soluble fraction was loaded onto a 5 mL HiTrap chelating HP column (GE Healthcare) immobilized with 100 mM cobalt chloride and equilibrated with the lysis buffer. After the removal of unbound proteins, the protein was eluted with lysis buffer supplemented with 100 mM imidazole. For the proteins expressed with the SUMO tag, there was an intermediate purification step that began with the removal of the 6xHisSUMO-tag, with the addition of Ulp1 protease to the eluted protein, followed by dialysis at 4°C for 12 h for removal of imidazole. The cleaved target proteins were purified after a second passage through the HiTrap chelating HP column immobilized with cobalt, being eluted in the unbound fraction. Molecular masses of the isolated proteins and the effector-immunity complex were determined by SEC-MALS (size-exclusion chromatography coupled to multi-angle light scattering), using a Superdex 200 10/300 GL (GE Healthcare) coupled to a Wyatt MALS detector. Graphs and the average molecular masses were generated using the ASTRA software (Wyatt), assuming a refractive index increment dn/dc = 0.185 mL/g.

### Immunoblot

Translocation assays were performed essentially as previously described [10]. Briefly, *S*. *maltophilia* wild-type and Δ*virD4* strains carrying pBRA-FLAG*-smlt3024* were grown overnight with antibiotics (150 μg/mL streptomycin), subcultured on the next day (1:25 dilution) and grown for an additional 2 h at 37°C (200 rpm). *E*. *coli* cells were subcultured (1:100 dilution) in a similar manner. *S*. *maltophilia* and *E*. *coli* cells were washed with 2x YT, OD_600nm_ adjusted to 1.0, mixed 1:1 volume and 5–10 μL were spotted onto dry nitrocellulose membranes, which were quickly placed onto LB-agar plates containing 0.1% L-arabinose to induce the expression of FLAG-Smlt3024. Plates were incubated at 30°C for 6 h, sufficient to allow detection of secreted proteins and before spontaneous cell death, which would produce background in the dot blot. After 6 h, membranes were washed with 5% low-fat milk diluted in PBS containing 0.02% sodium azide and processed for quantitative dot blot analysis with anti-FLAG rabbit polyclonal antibody, followed by IRDye 800CW anti-rabbit IgG (LI-COR Biosciences) and scanned using an Odyssey CLx infrared imaging system (LI-COR Biosciences). To obtain good signal to noise ratios, the membranes were washed in PBS/Tween (0.05%) at least four times for 1 h each. Quantification of signal intensity was performed using FIJI software [77].

### Crystallization, data collection and model building

6xHis-Smlt3025_86-333_ at a concentration of 8 mg/ml, was submitted to initial crystallization assays using the sitting drop vapour diffusion method with several commercial crystallization screening kits. 6xHis-Smlt3025_86-333_ successfully crystallized at 18°C, in the Morpheus conditions B4 and D8 (Molecular Dimensions). X-ray diffraction data of the crystals were collected in the MX-2 beamline of the National Laboratory of Synchrotron Light (Campinas, Brazil). Two datasets were acquired, a native dataset at 1.9 Å resolution and an iodine derivative dataset at 2 Å obtained after soaking the crystals for 40 s in the crystallization condition supplemented with 1M NaI. Space group determination and reflection intensity integration was calculated by the XDS program package [78]. Heavy atoms positions were found by SHELX [79], and the automated phasing and model building was performed with CRANK2 [80] within the CCP4i2 package [81]. The preliminary model was used for molecular replacement conducted with Phenix AutoSol [82] and applied to the native dataset to extend the structure resolution to a 1.9 Å resolution. Structural refinement of the model was performed using Phenix [82] and Coot [83]. Secondary structure was assigned by STRIDE [84].

## Supporting information

S1 Table List of putative *S*. *maltophilia* T4SS effector/immunity pairs identified by BLASTp search using *X*. *citri* XVIPCDs. (XLSX)

S2 Table List of proteins homologous to Smlt3024 identified by JACKHMMER analysis (rp75 database) after 3 iterations. (XLSX)

S3 Table Homologous X-Tfis in *Stentotrophomons maltophilia* K279a and *Xanthomonas citri* pv. citri 306. (DOCX)

S4 Table List of strains, primers and plasmids used in this study. (XLSX)

S1 Fig Phylogenetic distribution of *S*. *maltophilia* K279a T4SSs. Maximum-likelihood tree with 1000 bootstrap replicates built with amino acid sequence of VirD4 (Smlt3008) homologues using MEGA 7.0 [88]. VirB/T4SSs from *S*. *maltophilia* and *X*. *citri* [10] involved in interbacterial competition are highlighted in orange. Trb/T4SS from *S*. *maltophilia* is in green and the VirB/T4SS involved in conjugation [89] encoded by the pXAC64 plasmid from *X*. *citri* strain 306 is in blue [30].
(TIF)

S2 Fig Loading control for immunoblot assay. SDS-PAGE of total protein extracts followed by western blot of *S*. *maltophilia* strains carrying pBRA-FLAG-*smlt3024* or empty pBRA. RnhA (Ribonuclease HI) was used as loading control.
(TIF)

S3 Fig Bioinformatic analysis of Smlt3025 homologues. (A) Top 100 homologues of Smlt3025 in protein databases identified using the BLAST algorithm. The first 60 amino acids of the Clustal Omega alignment shows that almost all homologues have an annotated start codon that aligns with Smlt3025 Met_47_. (B) Alignment of the top 26 homologues of Smlt3025 in the KEGG database using the BLAST algorithm.
(DOCX)

S4 Fig List of Smlt3024 homologues greater than 750 residues in length identified by three iterations of PSI-BLAST. (DOCX)

S5 Fig WebLogo [86] representation of conserved positions in Smlt3025 homologues identified using the Consurf algorithm [87]. The conservation pattern was generated from the alignment of 48 sequences from the UniRef90 database. Color scheme corresponds to amino acids chemical properties (polar—green, neutral—purple, blue—basic, red—acidic, hydrophobic—black).
(PNG)

S1 Movie Time-lapse microscopy showing *S*. *maltophilia* wild-type interacting with *E*. *coli-*RFP. Dead/lysed *E*. *coli* cells are indicated by white arrows. Images were acquired every 10 min. Timestamps in hours:minutes. Scale bar 5 μm.
(AVI)

S2 Movie Time-lapse microscopy showing *S*. *maltophilia* Δ*virD4* interacting with *E*. *coli-*RFP. Images were acquired every 10 min. Timestamps in hours:minutes. Scale bar 5 μm.
(AVI)

S3 Movie Time-lapse microscopy showing *S*. *maltophilia* wild-type interacting with *Klebsiella pneumoniae-*RFP. Several *K*. *pneumoniae*-RFP lysis events can be observed. Images were acquired every 10 min. Timestamps in hours:minutes. Scale bar 5 μm. No *K*. *pneumoniae*-RFP lysis is observed in co-cultures grown using the T4SS-deficient *S*. *maltophilia* Δ*virD4* strain (S15 Movie).
(AVI)

S4 Movie Time-lapse microscopy showing *S*. *maltophilia* wild-type interacting with *Salmonella* Typhi*-*RFP. Several *S*. Typhi-RFP lysis events can be observed. Images were acquired every 10 min. Timestamps in hours:minutes. Scale bar 5 μm. No *S*. Typhi-RFP lysis is observed in co-cultures grown using the T4SS-deficient *S*. *maltophilia* Δ*virD4* strain (S16 Movie).
(AVI)

S5 Movie Time-lapse microscopy showing *S*. *maltophilia* wild-type interacting with *Pseudomonas aeruginosa-*GFP. Several *P*. *aeruginosa*-GFP lysis events can be observed. Images were acquired every 10 min. Timestamps in hours:minutes. Scale bar 5 μm. No *P*. *aeruginosa*-GFP lysis is observed in co-cultures grown using the T4SS-deficient *S*. *maltophilia* Δ*virD4* strain (S17 Movie).
(AVI)

S6 Movie Time-lapse microscopy showing *S*. *maltophilia* wild-type interacting with *X*. *citri* Δ*virB*-GFP. Dead/lysed *X*. *citri* cells are indicated by white arrows. Images were acquired every 15 min. Timestamps in hours:minutes. Scale bar 5 μm.
(AVI)

S7 Movie Time-lapse microscopy showing *S*. *maltophilia* Δ*virD4* interacting with *X*. *citri* Δ*virB*-GFP. Images were acquired every 15 min. Timestamps in hours:minutes. Scale bar 5 μm.
(AVI)

S8 Movie Time-lapse microscopy showing *S*. *maltophilia* Δ*virD4* interacting with *X*. *citri*-GFP. Dead/lysed *S*. *maltophilia* cells are indicated by yellow arrows. Images were acquired every 15 min. Timestamps in hours:minutes. Scale bar 5 μm.
(AVI)

S9 Movie Time-lapse microscopy showing wild-type *S*. *maltophilia* interacting with *X*. *citri*-GFP. Dead/lysed *X*. *citri* cells are indicated by white arrows and dead/lysed *S*. *maltophilia* cells are indicated by yellow arrows. Images were acquired every 15 min. Timestamps in hours:minutes. Scale bar 5 μm.
(AVI)

S10 Movie Time-lapse microscopy showing *E*. *coli* cells containing the empty pBRA plasmid grown with 0.2% L-arabinose. Images were acquired every 10 min. Scale bar 5 μm.
(AVI)

S11 Movie Time-lapse microscopy showing *E*. *coli* cells containing pBRA*-pelB-smlt3024* grown with 0.2% D-glucose. Images were acquired every 10 min. Scale bar 5 μm.
(AVI)

S12 Movie Time-lapse microscopy showing *E*. *coli* cells containing pBRA*-pelB-smlt3024* grown with 0.2% L-arabinose. Images were acquired every 10 min. Scale bar 5 μm.
(AVI)

S13 Movie Time-lapse microscopy showing *X*. *citri* Δ8Δ2609-GFP expressing pBRA-*smlt3025*_*45-333*_*/3024* interacting with *E*. *coli* cells carrying empty pEXT22 plasmid. Images were acquired every 20 min. Timestamps in hours:minutes. Scale bar 2 μm.
(AVI)

S14 Movie Time-lapse microscopy showing *X*. *citri* Δ8Δ2609-GFP expressing pBRA-*smlt3025*_*45-333*_*/3024* interacting with *E*. *coli* cells expressing Smlt3025_45-333_. Images were acquired every 20 min. Timestamps in hours:minutes. Scale bar 2 μm.
(AVI)

S15 Movie Time-lapse microscopy showing *S*. *maltophilia* Δ*virD4* strain interacting with *Klebsiella pneumoniae*-RFP. No *K*. *pneumoniae*-RFP lysis events are observed. Images were acquired every 10 min. Timestamps in hours:minutes. Scale bar 5 μm.
(AVI)

S16 Movie Time-lapse microscopy showing *S*. *maltophilia* Δ*virD4* strain interacting with *Salmonella* Typhi-RFP. No *S*. Typhi-RFP lysis are observed. Images were acquired every 10 min. Timestamps in hours:minutes. Scale bar 5 μm.
(AVI)

S17 Movie Time-lapse microscopy showing *S*. *maltophilia* Δ*virD4* strain wild-type interacting with Pseudomonas aeruginosa-GFP. No *P*. *aeruginosa*-GFP lysis events are observed. Images were acquired every 10 min. Timestamps in hours:minutes. Scale bar 5 μm.
(AVI)
