# Supplementary material for: The opportunistic pathogen Stenotrophomonas maltophilia utilizes a type IV secretion system for interbacterial killing
Source: PLoS Pathog. 2019 Sep 12;15(9):e1007651. doi: 10.1371/journal.ppat.1007651 (PMC6759196; doi:10.1371/journal.ppat.1007651)
Supplement: S3 Table — (DOCX) [file ppat.1007651.s003.docx]

**S3 Table. Homologous X-Tfis in *Stentotrophomons maltophilia* K279a and *Xanthomonas citri* pv citri 306.**

The sequences of the *S. maltophilia* X-Tfis listed in the leftmost column were used to query the *X. citri* genome using the Blast algorithm. Hits with *X . citri* X. Tfis are listed showing the number of identical residues over the total number of residues in the alignment, expressed as a percentage in parenthesis.

| ***Xanthomonas citri* 306 X-Tfis** | | | | | | | | | | | | | |
| --- | --- | --- | --- | --- | --- | --- | --- | --- | --- | --- | --- | --- | --- |
| ***Stenotrophomonas maltophilia* K279a X-Tfis** |  | **XAC**  **4264** | **XAC**  **3663** | **XAC**  **3267** | **XAC**  **3174*** | **XAC**  **2884** | **XAC**  **2610** | **XAC**  **1917** | **XAC**  **1163** | **XAC**  **0573** | **XAC**  **0467** | **XAC**  **0097** | **XAC**  **0150** |
|  | **Smlt4382** |  |  | **84/190 (44%)** |  |  |  |  |  |  |  |  |  |
|  | **Smlt4011** |  |  |  | **41/136 (30%)** |  |  |  |  | **26/99 (26%)** |  |  |  |
|  | **Smlt3025** |  |  |  |  |  |  |  |  |  |  |  |  |
|  | **Smlt2993** |  |  |  |  |  |  |  |  |  | **69/164**  **(42%)** |  |  |
|  | **Smlt2989** |  |  |  | **70/194 (36%)** |  |  |  |  | **118/204 (58%)** |  |  |  |
|  | **Smlt0506** |  |  |  |  |  |  |  |  |  |  |  |  |
|  | **Smlt0503** |  |  |  |  |  |  |  |  |  |  |  |  |
|  | **Smlt0501** |  |  |  |  |  |  |  |  |  |  |  |  |
|  | **Smlt0333** |  |  |  |  |  |  |  |  |  |  |  |  |
|  | **Smlt0274** |  |  |  |  |  |  |  |  |  |  |  |  |
|  | **Smlt0194** |  | **110/265 (42%)** |  |  |  |  |  |  |  |  |  |  |
|  | **Smlt0114** |  |  |  |  |  |  |  | **67/219**  **(31%)** |  |  |  |  |

* XAC3174 is the cognate X-Tfi of XAC3173, a putative *X. citri* X-Tfe not originally identified in Souza et al (2015) due to the relatively low similarity of its C-terminal region with other XVIPCD sequences.
