# Supplementary figures and images for: The opportunistic pathogen Stenotrophomonas maltophilia utilizes a type IV secretion system for interbacterial killing
Source: PLoS Pathog. 2019 Sep 12;15(9):e1007651. doi: 10.1371/journal.ppat.1007651 (PMC6759196; doi:10.1371/journal.ppat.1007651)

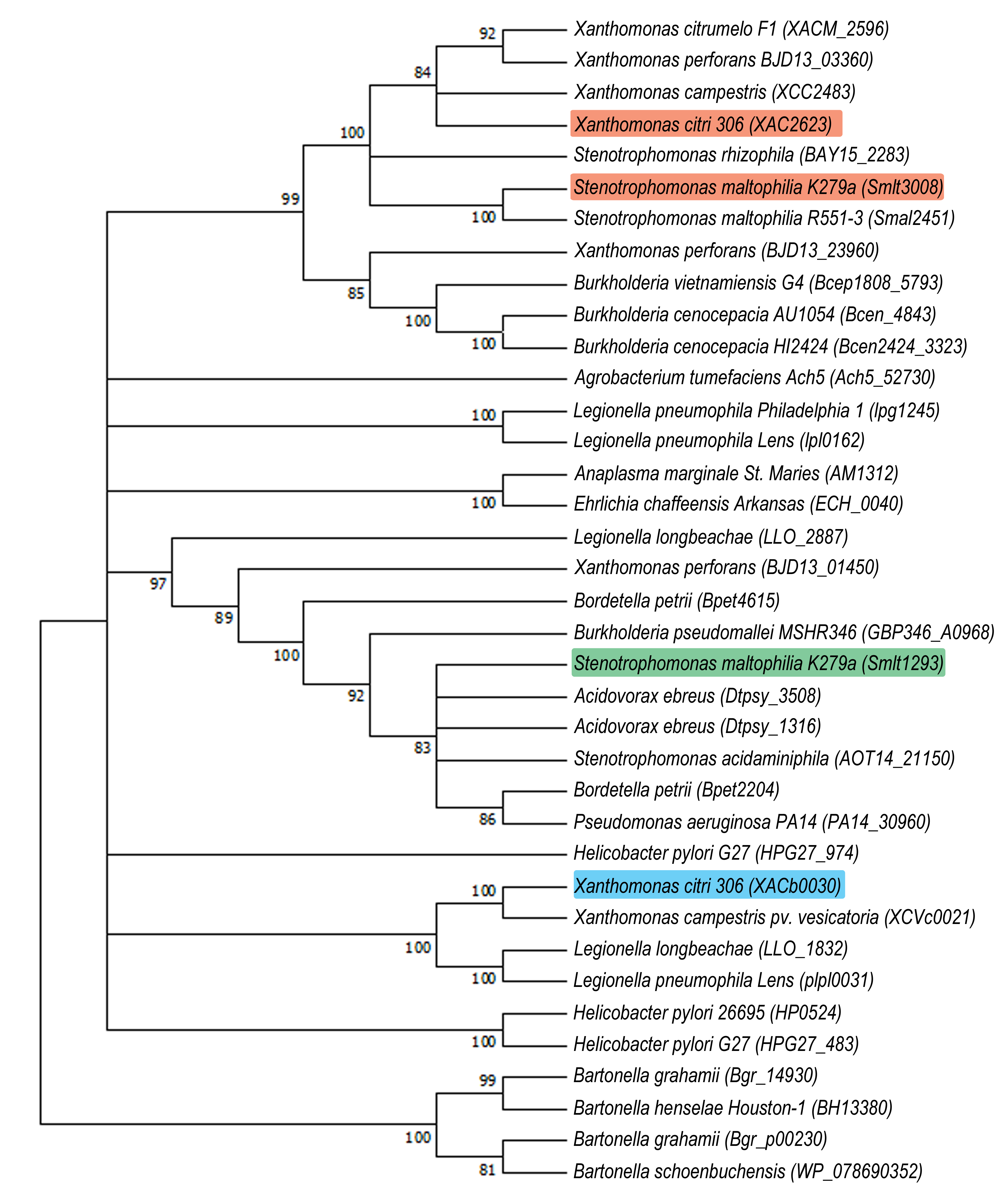

Supplement: S1 Fig — Maximum-likelihood tree with 1000 bootstrap replicates built with amino acid sequence of VirD4 (Smlt3008) homologues using MEGA 7.0 [88]. VirB/T4SSs from S. maltophilia and X. citri [10] involved in interbacterial competition are highlighted in orange. Trb/T4SS from S. maltophilia is in green and the VirB/T4SS involved in conjugation [89] encoded by the pXAC64 plasmid from X. citri strain 306 is in blue [30]. (TIF) [file ppat.1007651.s005.tif]

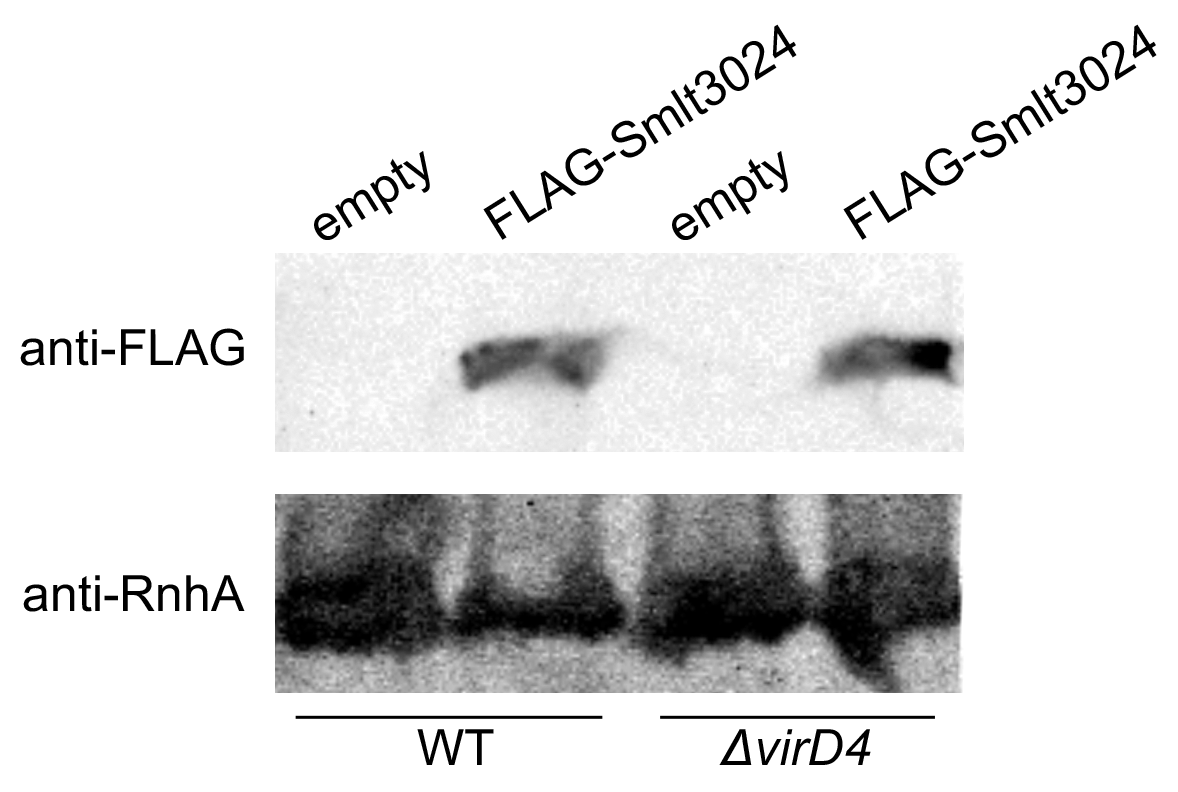

Supplement: S2 Fig — SDS-PAGE of total protein extracts followed by western blot of S. maltophilia strains carrying pBRA-FLAG-smlt3024 or empty pBRA. RnhA (Ribonuclease HI) was used as loading control. (TIF) [file ppat.1007651.s006.tif]

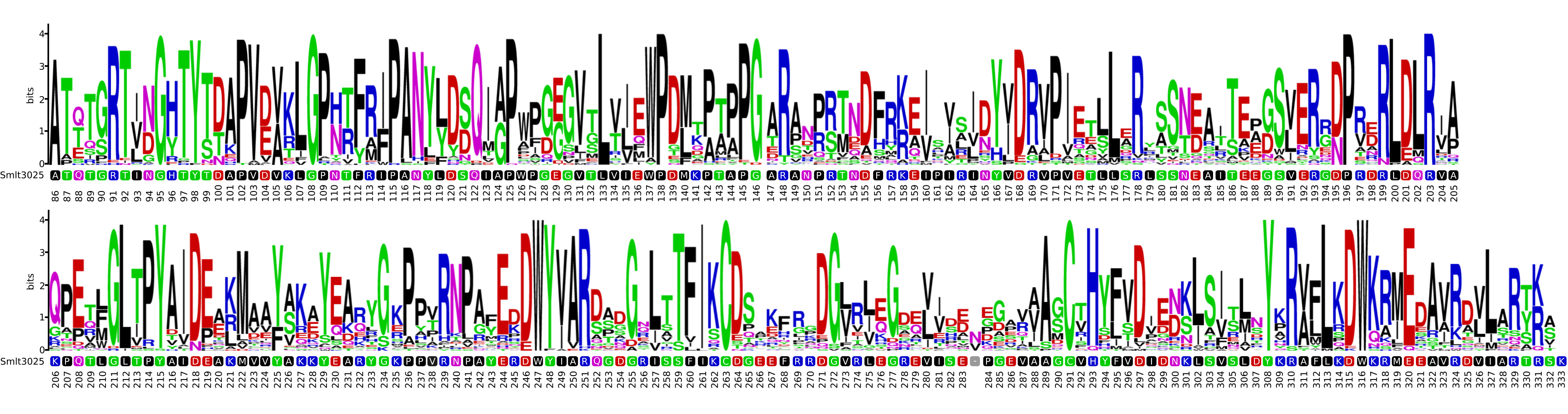

Supplement: S5 Fig — The conservation pattern was generated from the alignment of 48 sequences from the UniRef90 database. Color scheme corresponds to amino acids chemical properties (polar—green, neutral—purple, blue—basic, red—acidic, hydrophobic—black). (PNG) [file ppat.1007651.s009.png]
