# Supplementary material for: The opportunistic pathogen Stenotrophomonas maltophilia utilizes a type IV secretion system for interbacterial killing
Source: PLoS Pathog. 2019 Sep 12;15(9):e1007651. doi: 10.1371/journal.ppat.1007651 (PMC6759196; doi:10.1371/journal.ppat.1007651)
Supplement: S3 Fig — (A) Top 100 homologues of Smlt3025 in protein databases identified using the BLAST algorithm. The first 60 amino acids of the Clustal Omega alignment shows that almost all homologues have an annotated start codon that aligns with Smlt3025 Met47. (B) Alignment of the top 26 homologues of Smlt3025 in the KEGG database using the BLAST algorithm. (DOCX) [file ppat.1007651.s007.docx]

**S3A Fig.**: Alignment of the top 100 homologs of Smlt3025 in the nr protein database identified using the BLAST algorithm. The alignment with reference to the first 60 positions of Smlt3025 shows that almost all of these most similar homologs have an annotated start codon that aligns with Smlt3025 Met_47_. The annotated initiation methionine and other methionines at positions 13, 45, 47 and 50 of the Smlt3025 sequence are shown in bold in the first line.

CAQ46475.1_Smlt3025 **M**PKEYPQYCRQR**M**WSAAWFHEGLRCGSCSVDARLPIVALEPCNG**M**P**M**HV**M**SPSRCLTLAL 60

CCP17132.1 ------------MWSAASLGDGLRSNSCSIDARLPIVAPEPCNGMPMHVMSPGRCLTLAL 48

WP_044570473.1 ----------------------------------------------MHVMSPSRCLTLAL 14

WP_099605537.1 ----------------------------------------------MHVMSPSRCLTLAL 14

WP_099484037.1 ----------------------------------------------MHVMSPSRCLTLAL 14

WP_059034745.1 ----------------------------------------------MHVMSPSRCLTLAL 14

WP_111188249.1 ----------------------------------------------MHVMSPSRCLTLAL 14

WP_111108321.1 ----------------------------------------------MHVMSPSRCLTLAL 14

WP_111687446.1 ----------------------------------------------MHVMPPSRCLTLAL 14

WP_088436602.1 ----------------------------------------------MHVMSPSRCLTLAL 14

WP_032963213.1 ----------------------------------------------MHVMSPSRCLTLAL 14

WP_005413854.1 ----------------------------------------------MHVMSPSRCLTLAL 14

WP_099479415.1 ----------------------------------------------MHVMSPSRCLTLAL 14

WP_049430658.1 ----------------------------------------------MHVMSPSRCLTLAL 14

WP_049449113.1 ----------------------------------------------MHVMSPSRCLTLAL 14

WP_111203532.1 ----------------------------------------------MHVMSPSRCLTLAL 14

WP_049402219.1 ----------------------------------------------MHVMSPSRCLTLAL 14

WP_108323578.1 ----------------------------------------------MHVMSPSRCLTLAL 14

WP_100478353.1 ----------------------------------------------MHVMSPSRCLALAL 14

WP_134709508.1 ----------------------------------------------MHVMSPSRCLALAL 14

WP_099495551.1 ----------------------------------------------MHVMSPSRCLALAL 14

WP_049471385.1 ----------------------------------------------MHVMSPGRCLTLAL 14

WP_088475939.1 ----------------------------------------------MHVMSPGRCLTLAL 14

WP_049422482.1 ----------------------------------------------MHVMSPSRCLTLAL 14

WP_049448527.1 ----------------------------------------------MHVMSPSRCLTLAL 14

WP_074725410.1 ----------------------------------------------MHVMSPGRCLTLAL 14

WP_057494684.1 ----------------------------------------------MHVMSPGRCLTLAL 14

WP_053462717.1 ----------------------------------------------MHVMSPGRCLTLAL 14

WP_049429706.1 ----------------------------------------------MHVMSPSRCLTLAL 14

WP_111201530.1 ----------------------------------------------MHVMSPSRCLTLAL 14

WP_103281792.1 ----------------------------------------------MHVMSPGRCLTLAL 14

WP_099590075.1 ----------------------------------------------MHVMSPSRCLTLAL 14

WP_072168103.1 ----------------------------------------------MHVMSPGRCLTLAL 14

WP_100442312.1 ----------------------------------------------MHVMSPSRCLTLAL 14

WP_107379812.1 ----------------------------------------------MHVMSPSRCLTLAL 14

WP_111111421.1 ----------------------------------------------MHVMSPRRCLALAL 14

WP_032957403.1 ----------------------------------------------MHVMSPSRCLTLAL 14

WP_106549074.1 ----------------------------------------------MHVMSPRRCLALAL 14

WP_126927586.1 ----------------------------------------------MHVMSPSRCLTLAL 14

WP_014037675.1 ----------------------------------------------MHVMSPGRCLTLAL 14

WP_136638220.1 ----------------------------------------------MHVMSPGRCLTLAL 14

WP_012511459.1 ----------------------------------------------MHVMSPGRCLTLAL 14

WP_071227577.1 ----------------------------------------------MHVMSPRRCLPLAL 14

WP_100468887.1 ----------------------------------------------MHVMSPGRCLTLAL 14

WP_106468498.1 ----------------------------------------------MHVMSPGRCLTLAL 14

WP_049398247.1 ----------------------------------------------MHVMSPRRCLPLAL 14

WP_133935035.1 ----------------------------------------------MHVMSPGRCLTLAL 14

WP_100473239.1 ----------------------------------------------MHVMSLGRCLTLAL 14

WP_057500576.1 ----------------------------------------------MHVMSPSRCLTLAL 14

WP_134970662.1 ----------------------------------------------MHVMSPSRCLTLAL 14

TGR56376.1 ----------------------------------------------MHVMTPGRCLTLAL 14

WP_032128614.1 ----------------------------------------------MHVMSPRRCLPLAL 14

SQG11074.1 ------------------------------------------------------------ 0

CRD52651.1 ------------------------------------------------------------ 0

AIL06714.1 ------------------------------------------------------------ 0

CRQ98578.1 ------------------------------------------------------------ 0

WP_053451571.1 ----------------------------------------------MHVMSPGRCLTLAL 14

WP_087922968.1 ----------------------------------------------MHVMSPGRCLTLAL 14

WP_080347500.1 ----------------------------------------------MRVMSPGRCLTLAL 14

VEE51067.1 ------------------------------------------------------------ 0

WP_119006468.1 ----------------------------------------------MHVMSPGRCLTLAL 14

WP_126411110.1 ----------------------------------------------MHVMSPGRCLTLAL 14

WP_041863587.1 ----------------------------------------------MHVMSPGRCLTLAL 14

KUP00661.1 --------------------------------------------MPMHVMSPSRCLTLAL 16

WP_099475956.1 ----------------------------------------------MHVMSPGRCLTLAL 14

WP_065724491.1 ----------------------------------------------MHVMSPSRCLTLAL 14

WP_135967017.1 ----------------------------------------------MLVMSPGRCLTLAL 14

KDE91249.1 ------------------------------------------------------------ 0

WP_043034636.1 ----------------------------------------------MHVMSPSRCLTLAL 14

WP_049396630.1 ----------------------------------------------MHVMSPSRCLTLAL 14

WP_033833181.1 ----------------------------------------------MHVMSPSRCLTLAL 14

WP_062607322.1 ----------------------------------------------MHVMSPSRCLTLAL 14

WP_032965909.1 ----------------------------------------------MHVMSPSRCLTLAL 14

WP_100438704.1 ----------------------------------------------MHVMSPSRCLTLAL 14

WP_014647603.1 ----------------------------------------------MHVMSPGRCLTLAL 14

WP_087803497.1 ----------------------------------------------MHVMSPSRCLTLAL 14

WP_121503602.1 ----------------------------------------------MHVMPPSRCLTLAL 14

WP_099473722.1 ----------------------------------------------MHVMSPSRCLTLAL 14

WP_088429410.1 ----------------------------------------------MHVMSPSRCLTLAL 14

WP_088479772.1 ----------------------------------------------MHVMSPSRCLTLAL 14

WP_111207071.1 ----------------------------------------------MHVMSPGRCLTLAL 14

WP_049458677.1 ----------------------------------------------MHVMSPGRCLTLAL 14

WP_094000917.1 ----------------------------------------------MHVMSPGRCLTLAL 14

HCT28595.1 ----------------------------------------------MHVMSPSRCLTLAL 14

WP_080148498.1 ----------------------------------------------MHPMSSGRCLALAI 14

WP_105164308.1 ----------------------------------------------MHPMSYGRCLALAI 14

WP_053497255.1 ----------------------------------------------MHVMSPGRCLTLAL 14

WP_100442310.1 ----------------------------------------------MHVMSPSRCLMLAL 14

WP_125428345.1 ----------------------------------------------MHVMSPGRCLTLAL 14

WP_053451569.1 ----------------------------------------------MHVMSPGRCLTLAL 14

WP_099475958.1 ----------------------------------------------MHVMSPGRCLTLAL 14

WP_088026391.1 ----------------------------------------------MHVMSPGRCLTLAL 14

RKQ56508.1 -------------------------------------------------MPPSRCLTLAL 11

WP_126411108.1 ----------------------------------------------MHVMSPGRCLTLAL 14

WP_064238803.1 ----------------------------------------------MHVMSHGRCLTLAL 14

WP_134966428.1 ----------------------------------------------MHVMSPGRCLTLAL 14

WP_046431393.1 ----------------------------------------------MHVMSPGRCLTLAL 14

WP_088375515.1 ----------------------------------------------MHVMSPGRCLTLAL 14

WP_032952091.1 ----------------------------------------------MHPMSSGRCLALAI 14

WP_033831447.1 ----------------------------------------------MHPMSSGRCLALAI 14

**S3B Fig**: Alignment of the top 26 homologs of Smlt3025 in the KEGG database using the BLAST algorithm (all alignments have e-values less than 1e^-26^). Most of the homologs have an annotated start codon that aligns with Met_47_ of Smlt3025. The annotated initiation methionine at position 1 and other methionines at positions 13, 45, 47 and 50 of the Smlt3025 sequence are shown in bold in the first line.

sml:Smlt3025 **M**PKEYPQYCRQR**M**WSAAWFHEGLRCGSCSVDARLPIVALEPCNG**M**P**M**HV**M**SP---SRCLT 57

buj:BurJV3_2536 ----------------------------------------------MHVMSP---GRCLT 11

smt:Smal_2473 ----------------------------------------------MHVMSP---GRCLT 11

sten:CCR98_13110 ----------------------------------------------MHVMSP---GRCLT 11

smz:SMD_2664 ----------------------------------------------MHVMSP---GRCLT 11

stem:CLM74_13430 ----------------------------------------------MHPMSY---GRCLA 11

smz:SMD_2666 ------------------------------------------------------------ 0

xfr:BER92_13825 ----------------------------------------------MRMMSS---GRCLA 11

xva:C7V42_06115 ----------------------------------------------MHMMSS---GRCLA 11

srh:BAY15_0594 ----------------------------------------------MSVMSS---GRCLT 11

xve:BJD12_19055 ----------------------------------------------MHMMSS---GRCLA 11

xcp:XCR_1262 ------------------------------------------------------------ 0

srh:BAY15_0595 ----------------------------------------------MSVMSS---GRCLT 11

srh:BAY15_0591 ----------------------------------------------MSVMSS---GRCLS 11

srh:BAY15_0589 ----------------------------------------------MSVMSS---GRCLS 11

smt:Smal_0379 ----------------------------------------------MNLPPP---GRWLA 11

sml:Smlt0501 ----------------------------------------------MSRAPA---CRLAS 11

lcp:LC55x_0313 ------------------------------------------------------------ 0

xtn:FD63_00375 ----------------------------------------------MRSVSRLA--FCLL 12

xhr:XJ27_15260 -----------------------------------------------------------M 1

xga:BI317_01130 -----------------------------------------------------------M 1

xsa:SB85_13075 ----------------------------------------------MRPATRLA--QCLL 12

xcp:XCR_4524 ------------------------------------------------------------ 0

xca:xcc-b100_4398 ----------------------------------------------MRHPLALAGLACMV 14

xcb:XC_4270 ----------------------------------------------MRHPLALAGLACMV 14

xcc:XCC4183 ----------------------------------------------MRHPLALAGLACMV 14

lez:GLE_5040 ------------------------------------------------MTPP---RAPLP 9

sml:Smlt3025 LALAV-AVSAGACARPPSNEHE-RNPAMSGATQTGRT---INGHTYTDAPVDVKLGPNTF 112

buj:BurJV3_2536 LALAV-VVSAGACARPPSTEHE-RNPAMSGATQTGRT---INGHTYTDAPVDVKLGPNTF 66

smt:Smal_2473 LALAV-VVSAGACARPPSTEHE-RNPAMSGATQTGRT---INGHTYTDAPVDVKLGPNTF 66

sten:CCR98_13110 LALAV-VVSAGACARPPSTEHE-RNPAMSGATQTGRT---INGHTYTDAPVDVKLGSNTF 66

smz:SMD_2664 LALAV-AVSAGACARPPSTEHE-RNPAMSGATQTGRT---INGHTYTDAPVDVKLGPNTF 66

stem:CLM74_13430 LAITV-AVSASACARPTSTEHE-RNPAMPGATQTGRT---LNGHTYTDAPVDVKLGPNTF 66

smz:SMD_2666 ---------------------------MSGAPQTGRT---INGHTYTDAPVDVKLGPNTF 30

xfr:BER92_13825 LALAV-AVTGSAYARTPSTEQK-RTSPMSDTVTTGRT---INGHTYSDAPVDVKLGPNTF 66

xva:C7V42_06115 LALAV-AVTGSTCARTPSPEQK-RTSTMSGTVTTGRT---INGHTYTDAPVDVKLGPHIF 66

srh:BAY15_0594 VALAV-ALSASACARTPPTEHK-GTSNMSEATETGRT---LNGHTYTDAPVDVKLGPNTF 66

xve:BJD12_19055 LALAV-AVAGSACARTPSPEQK-RTSPMSGTVTTGRT---INGHTYSDAPVDVKLGPHIF 66

xcp:XCR_1262 ---------------------------MSGTVTTGRT---INGHTYSDAPVDVKLGPNTF 30

srh:BAY15_0595 VALAV-ALSASACVRTPPTEHK-GISNMSEATETGRT---LNGHTYTDAPVDVKLGPNTF 66

srh:BAY15_0591 VALAV-ALSASACAKAPPTEKGNKEPAMSEDTSQSRT---VDGYTYTTAPVVAQLGPHRY 67

srh:BAY15_0589 VALAV-ALSASACAKAPPTEKGNKEPAMSEDTSQSRT---VDGYTYTTAPVVAQLGPHRY 67

smt:Smal_0379 LAIAL-A-SSTACSKAPSKEPEHRETPM-TDTAQSHT---VDGYTYTTAPVEATLGPHRY 65

sml:Smlt0501 FALVA-VLSTTACAKAPSTEKDPNGTPMSETATSPRT---VGGQTYTTQPVEARLGPHRF 67

lcp:LC55x_0313 ---------------------------------MTDA---AVASPYNDKPVLAKLGPHRF 24

xtn:FD63_00375 LTS---VLPMVGCAKPSAADSPAATSTNAQGCARKRHLGPQDPFQTPPPLMEACLGPYKL 69

xhr:XJ27_15260 HGT---G--LLRS---GLQRNANRRQHHDQRCVRQR-GGPQDPGTSPPPVMEACLGPYKL 52

xga:BI317_01130 HGT---G--LLRS---GLQRNANRRQHHDQRCVRQR-GGPQDPGTSPPPVMEACLGPYKL 52

xsa:SB85_13075 LAG---ILPLAGCAQSGSADTPAATSTNAQGCVRKRQLGPQDPFKTPPPLMEACLGPYKL 69

xcp:XCR_4524 --------------------------------------------------MEACLGPYKL 10

xca:xcc-b100_4398 LGF---S--VPGC---SATQTGANTMTNEQGCVRQR-GGPQDPGTSPPPVMEACLGPYKL 65

xcb:XC_4270 LGF---S--VPGC---SATQTGANTMTNEQGCVRQR-GGPQDPGTSPPPVMEACLGPYKL 65

xcc:XCC4183 LGF---S--VPGC---SATQTGANTMTNEQGCVRQR-GGPQDPGTSPPPVMEACLGPYKL 65

lez:GLE_5040 WLLAIACLAAAACGSGAAPPP-------AAEASRPMT---PTDANAGERMVEVHLGPHRF 59

: . **

sml:Smlt3025 RIPANYLDSQIAPWPGEGVTLVIEWPDMKPTAPGARANP-RTNDFRKEIPIRINYVDRVP 171

buj:BurJV3_2536 RIPANYLDSQIAPWPGEGVTLVIEWPDMKPTLPGARANP-RTNDFRKEIAVRINYIDRIP 125

smt:Smal_2473 RIQANYLDSQIAPWPGEGVTLVIEWPDMKPTLPGARANP-RTNDFRKEIPIRINYVDRVP 125

sten:CCR98_13110 RIPANYLDSQIAPWPGEGVTLVIEWPDMKPSAPGARASP-RTNDFRKEIAVRINYIDRIP 125

smz:SMD_2664 RIPANYLDSQIAPWPGEGVTLVIEWPDMKPTAPGARANP-RTNDFRKEIAVAIDYIDRAP 125

stem:CLM74_13430 RIPANYLDSQIAPWPGEGVSLVVEWPDMTPTLPGARANP-RTNDFRKEISVRINYVDRVP 125

smz:SMD_2666 RIPANYLDSQIAPWPGEGVTLVIEWPDMKPTPPGARANP-RTNDFRKEIAVRINYIDRIP 89

xfr:BER92_13825 RIPANYLDSQIAPWPGEGVTLLIEWPEMKPTPPGARVNP-RTNDFRKEISVSIDYIDRAP 125

xva:C7V42_06115 RIPANYLDSQIAPWPGEGVTLVIEWPNMTPTPPGARANP-RTNDFRKEIHASIDYVDRVP 125

srh:BAY15_0594 RIPANYLDSQIAPWPGDGVSLVIEWPDMNPTAPGARVDP-RTNDFRKEIAVSIDYIDTAP 125

xve:BJD12_19055 RIPANYLDSQIAPWPGEGVTLVIEWPDMPPTPPGARANP-RTNDFRKEISVSIDYVDRVP 125

xcp:XCR_1262 RIPANYLDSQIAPWSIEVVTLVIEWPDMTPTPPGARANP-RTNDFRKEIHASMNYVDRVP 89

srh:BAY15_0595 RIPANYLDSQIAPWPGEGVSLVIEWPDMTPTPPGARANP-RTNDFRKEIRILVDHIDRVP 125

srh:BAY15_0591 AFPANFYDDQIGPAIGGGVGLTLMWPELKAAAPGTRGTR-SMADHHRAMSMSVDYIDGVP 126

srh:BAY15_0589 AFPANFYDDQIGPSVGGGIALSFIWPGLQAAAPGDRPTR-SMEDHYRTVTASIDYLDAVP 126

smt:Smal_0379 AFPANLYDDQMGPAVGGGIGLTLLWPDLQAAPPGTRASR-SMSDHHRAISLSLDYIDALP 124

sml:Smlt0501 MFPANLYYNQTGPLADGGVMLTVFWPDFDAAPPGDRPVR-STQDSRRQVLVELRYIDRVP 126

lcp:LC55x_0313 EIPANYFDTQLGPDFQGNMRLIVQWPDLQPLAPGERYFEGGEQRFARNIDITPNYIDRVP 84

xtn:FD63_00375 RIPANYFGDQMGPNFDDSFGLYLEYPTLEPFAPGERSHL-SLDVATRTVNIGYSYLDRVD 128

xhr:XJ27_15260 QIPANYFDDQMGPNFDGSFGLYLEYPELNAFAPGERAHL-KLDVATRTVNIGYRYLDRVD 111

xga:BI317_01130 QIPANYFDDQMGPNFDGSFGLYLEYPELNAFAPGERAHL-KLDVATRTVNIGYRYLDRVD 111

xsa:SB85_13075 RIPANYFGDQMGPNFDDSFGLYLEYPSLQPFAPGERTHL-RLDVSTRTVDIGYHYLDRVD 128

xcp:XCR_4524 HIPANYFDDQMGPNFDGSFGLYLEYPELNAFAPGERAHL-KLDVATRTVNIGYRYLDHVE 69

xca:xcc-b100_4398 QIPANYFDDQMGPNFDGSFGLYLEYPELNAFAPGERAHL-KLDVATRTVNIGYRYLDRVD 124

xcb:XC_4270 QIPANYFDDQMGPNFDGSFGLYLEYPELNAFAPGERAHL-KLDVATRTVNIGYRYLDRVD 124

xcc:XCC4183 QIPANYFDDQMGPNFDGSFGLYLEYPELNAFAPGERAHL-KLDVATRTVNIGYRYLDRVD 124

lez:GLE_5040 RIASNYFDIERGQDAQGFMRLILRWPELAPLPAGTHYLSGGESERARNIDISPDYIDRVP 119

: :* : . . * . :* : * : : : ::*

sml:Smlt3025 VETLLSRLSSNEAITEEGSVERGDPRDRLDQRVAKPQTLGLTPYAIDEAKMVVYA-KKYE 230

buj:BurJV3_2536 IETSLSRLSSNEAITEEGSVEREDPRDRLDQRVAQPETLGLTPYAIDEAKMAVYA-KKYE 184

smt:Smal_2473 VETLLSRLSSNEAVTEEDSVEREDPRDRLDQRVAQPETLGLTPYAIDEAKMVVYA-KKYE 184

sten:CCR98_13110 IETSLSRLSSNEAITEEGSVEREDPRDRLDQRVAQPETLGLTPYAIDEAKMVVYA-KKYE 184

smz:SMD_2664 IETSLERLSSNQASTEEGSVERGDPRDRLDLRIARPDVMGLTPYAIDETRMGIFS-KEYE 184

stem:CLM74_13430 IEGLMERYASNEALTEADSVERGDPRDRLDLRIPLSETMGLTPYAIDEAAMKEFA-KRYE 184

smz:SMD_2666 IETSLSRLSSNEAITEEGSVEREDPRDRLDQRVAQPETLGLTPYAIDEAKMVLYA-KKYE 148

xfr:BER92_13825 IETSLERLSSNEAITEDGSLERRDPRDRLDLRIAQAKTMGLMLYAIDEAKMAGYS-KEYE 184

xva:C7V42_06115 IEALLARYSSNEAITEPDWVERGNPAERLDLRIAQPETLGLTPYAIDEEKMAVYV-KAYE 184

srh:BAY15_0594 IETSLERLSSNTAVTAEGSVERDNPKDRLDLRVAQPETMGLIPYAIDEAKMAAYS-KAYQ 184

xve:BJD12_19055 IEALLERFSSNDGRTEAGSVERGNPVARLDLRIAQPEAFGLTPYAIDEEKMAAYV-KAYQ 184

xcp:XCR_1262 IEGLLTRYSSNEALTEPDSVERGDPVDRLDLRIAQPETLGLTPYAIDEEKMAVYV-KAYE 148

srh:BAY15_0595 IETLLQRQVSNDAITVEGSLERNDPSSRLDLRVAQNEQLGLTPYAINDALMTEYA-RAYE 184

srh:BAY15_0591 ITELLGRMTSTDATSEEGSAYRKDPRRRLDMRTPGAEQFGLTPYAIDEARMVEFA-QAYK 185

srh:BAY15_0589 ASELLPRLSNTEATTEAGSVNRNDPRRRLDLRKAGTERFGLIPYAIDEERMAQFG-NDYQ 185

smt:Smal_0379 IAELLPRKTSTEATTEDGSINRDDPRRRLDLRNAGTPHFGLTPYAIDEARMTRFS-EAYA 183

sml:Smlt0501 IQNYLARRSSNEATSAPGSLERRDPVENLALRVAQPERWGLTPYAIDPSLMAAYA-KDSE 185

lcp:LC55x_0313 LQTSLDRALISNVDSE--QKRREDPTLNPDLRLPGEPVFGLTPYYTDFAKVDAYNLKVYG 142

xtn:FD63_00375 VREALRNQYTP------SSNEQDNPAERLESRIRGATTYGLTPYYADLPKVFAYY-RAKG 181

xhr:XJ27_15260 PDAFLRRQYTP------DSATQDTPEADINTRIKGEEKDGLTPYYANVAAYREHY-LAQG 164

xga:BI317_01130 PDAFLRRQYTP------DSATQDTPEADINTRIKGEEKDGLTPYYANVAAYREHY-LAQG 164

xsa:SB85_13075 VHEALRRRYTG------YSADKDNPAENLESRLEQRSVYGLTPYYADLPKVFAYY-KAKG 181

xcp:XCR_4524 TDAYLQRQYTP------DGAAHDAPEADINTRTKGEEIDGLTPYYANVAAYREHY-LAQG 122

xca:xcc-b100_4398 PDAFLRRQYTP------DGATQDTPEADINTRTKGEEIHGLTPYYANVAAYREHY-LAQG 177

xcb:XC_4270 PDAFLRRQYTP------DGATQDTPEADINTRTKGEEIHGLTPYYANVAAYREHY-LAQG 177

xcc:XCC4183 PDAFLRRQYTP------DGATQDTPEADINTRTKGEEIHGLTPYYANVAAYREHY-LAQG 177

lez:GLE_5040 LQTSLERDLISNTDSE--QERRENPTLNPDLRIHGDPVHGLTPYYTDFAKVDAYYRARYG 177

: . : * * ** * : .

sml:Smlt3025 ARYGK----PPVRNPAYERDWYIARQGDGRISSFIKCDGEEFRRDGVRLEGREVISEPGE 286

buj:BurJV3_2536 ARYGK----PPVRNPAYERDWYIARQADGRISSFIKCDGKEFRRDGVRLEGREVISEPGE 240

smt:Smal_2473 ARYGK----PPVRNPAYERDWYIARQADGRISSFIKCDGKEFRRDGVRLEGSEVISEPGE 240

sten:CCR98_13110 ARYGK----PPVRNPAFERDWYVARDGAGGLTSFIKCDSATFLGDGVRLTQDEVIDLDDP 240

smz:SMD_2664 ARYGK----PPTRNPAYEDDWYIARAPNGSLTTFIKCESTAFRGDGVRLQGDQVISEQGQ 240

stem:CLM74_13430 ARYGK----APVRNPAYERDWYVARQADGRISTFIKCDAEGFRQDGVRLEGDQVISVQGE 240

smz:SMD_2666 ARYGK----PPVRNPAFERDWYVARDGAGGLTSFIKCDSATFLGDGVRLTQDEVIDLDDP 204

xfr:BER92_13825 TRYGK----PPTRNPGYEDDWYVARGPKGNLTTFIKCDSKTFRGDGVRLEGNQVISEDGA 240

xva:C7V42_06115 ARYSK----PPTRNPAFEDDWYVARDSGGNLTTFIKCDSVKFRKDGIRLEGDQVIDEEEP 240

srh:BAY15_0594 TRYGK----PHTRNPGFEDDWYVARDPSGNLTTFIKCDSSTFREDGVQLKGDQVIQEQGA 240

xve:BJD12_19055 AHHGE----PPTRNPAFEDDWYVARDSNGNLTTFIKCDSKKFRADGVQLEGSEVVHEKGA 240

xcp:XCR_1262 AHYGK----PPTRNPAFEDDWYVARESSGKLTTFIKCDSKKYRGDGVRLEGSEVVHEKGA 204

srh:BAY15_0595 AKYGH----PHPRNPAYEDDWYIARDPAGQLSTFIKCDSRKHRPDGFEIKGKELVNTDSR 240

srh:BAY15_0591 KQTGM----PAKRNANVESDWYIARTPGGELATFIKCDKLQEGRDSLTLKGDQVEIDESV 241

srh:BAY15_0589 KQFGE----PVTRNPRKEDEWYIARAANGMLATFIKCDQPEGGNEGLAIQGDRLVPDESV 241

smt:Smal_0379 AQRGT----PPVRTPETDQEWYIARDTQGALATFITCNPPQDGREGLTVQGTTLVDDPDV 239

sml:Smlt0501 AALGR----PYVHNPGMEPDWYVARSMEGQLTTFISCDPADRIPDGLVVQGKTLERAGEG 241

lcp:LC55x_0313 DKTSR----AGDRDSLFNNDWFVARDADGALTTVIKCTSRE-MPDGAEIEGAALRLLGTP 197

xtn:FD63_00375 YSESHSDESTSIFNAASHKDWYVALDDKGQISTIIKCTSKEVTQSGVEYHDGKLVRNKEE 241

xhr:XJ27_15260 LKPS-----NSIMEASYYKDWYVSRDAQGNIDTIIKCTSREVEPSGVEFREGKLVRSKER 219

xga:BI317_01130 LKPS-----NSIMEASYYKDWYVSRDAQGNIDTIIKCTSREVEPSGVEFREGKLVRSKER 219

xsa:SB85_13075 YQAS-----APVFKAEEHDDWYVERDADGEIRTFINCTSHVVAETGVEYRDGKLVRSKDA 236

xcp:XCR_4524 LKPS-----NSIMAASYYKDWYVSRDAQGNIETIIKCTSREVEQSGVEYRDGKLARSKEQ 177

xca:xcc-b100_4398 LKPS-----NSIMEASYYKDWYVSRDAKGNIDTIIKCTSRAVEPSGVEFREGKLVRSKER 232

xcb:XC_4270 LKPS-----NSIMEASYYKDWYVSRDAKGNIDTIIKCTSRAVEPSGVEFREGKLVRSKER 232

xcc:XCC4183 LKPS-----NSIMEASYYKDWYVSRDAKGNIDTIIKCTSRAVEPSGVEFREGKLVRSKER 232

lez:GLE_5040 KHGDT----AAQRHSPFNNDWLVGRDAQGTLTTVIKCTSRE-EPEGARIVEGRLELLDAP 232

. :* : * : :.*.* . :

sml:Smlt3025 VAAGCVHYFVDIDNKLSVSLDYKRAFLKDWKRMEEAVRDVIARTRSK------------ 333

buj:BurJV3_2536 VAAGCVHYFVDIDNKLSVSLDYKRAFLKDWKRMEEAVRDVIARTRSQ------------ 287

smt:Smal_2473 VAAGCVHYFVDIDNKLSVSLDYKRAFLKDWKRMEEVVRDVIARTRSQ------------ 287

sten:CCR98_13110 VAAGCVHYFVDISDSLSISLHYKRAFLKDWKRMEDAVRDVLAAARTR------------ 287

smz:SMD_2664 AAAGCFHYFSDVENKLSISLNYKRAFLADWKRMEDAVRDLLARSRVRQ----------- 288

stem:CLM74_13430 VAAGCVHYFVDGDNKLSVTLDYKRAFLKDWKRMEDAIRDVITRTRSN------------ 287

smz:SMD_2666 VAAGCVHYFVDISDSLSISLHYKRAFLKDWKRMEDAVRDVLAAARTR------------ 251

xfr:BER92_13825 VAAGCFHYFSDIENNLSITLTYKRAFLKDWKRMESAVRHALARTKVQ------------ 287

xva:C7V42_06115 VIAGCTHYLVDVENKLSITLNYHRAFLRDWKRMEKAIRDVLVNTKVG------------ 287

srh:BAY15_0594 VAAGCIHYFTDVENKLSITLDYKRAFLKDWKRMEDAVRDLLARTKAR------------ 287

xve:BJD12_19055 VAASCVHYFSDIENKLSISLNYKRAFLKDWRRMEDAVRNVLARTKIQ------------ 287

xcp:XCR_1262 VAASCVHYFSDIENKLSITLNYKRAFLKDWKRMEDAVKEVLSRTKVR------------ 251

srh:BAY15_0595 TVASCTHYIVDTENSLSISLHYNRVFLKDWKAMEVAVRDVLNRFKVE------------ 287

srh:BAY15_0591 PVSGCTHNFVDAVNDLSVTLSYPRVFLKDWKAIEAAARGVLSAYKVE------------ 288

srh:BAY15_0589 KVSSCTHYFVDSNDNLGVTLFYPRVLLKDWKAMEEATRAALAKYKVR------------ 288

smt:Smal_0379 PVATCTHTFTDPQDGLSVRAIYPRVLLKDWKAVEDATRVLLARYKLR------------ 286

sml:Smlt0501 QIAMCRHSIVDVGDSIAIEMNYARVMLSDWQRLENSVRDLLSRYRVQP----------- 289

lcp:LC55x_0313 KLSMCSHDFTIARYNARINVSYLRVFMQDWKKIEQRIRDLFDQYHINDAR--------- 247

xtn:FD63_00375 ALPECDHFFIVQDLKVLVEIRYVRFALPDWKKVEETARDELRKFMVEKPHS-------- 292

xhr:XJ27_15260 ELPSCAHVFVIPEMKVAVEINYVRVALKDWKKIQDRARSVLKDFMPAPAGT-------- 270

xga:BI317_01130 ELPSCAHVFVIPEMKVAVEINYVRVALKDWKKIQDRARSVLKDFMPAPAGT-------- 270

xsa:SB85_13075 ELPECHHIFALPEISTLVEVRYVRAALPDWKRIEDAARSYLEKFMVTRDRTQNR----- 290

xcp:XCR_4524 ELPTCTHVFVIREMKVAVEIDYVRAALKDWKKIQDRARSALKEFMAAPVGA-------- 228

xca:xcc-b100_4398 ELPSCEHVFVIPEMKVAVEIDYVRVALKDWKKIQDRARSALKDFMPAPTGT-------- 283

xcb:XC_4270 ELPSCEHVFVIPEMKVAVEIDYVRVALKDWKKIQDRARSALKTSCRRPPAPERLPATRR 291

xcc:XCC4183 ELPSCEHVFVIPEMKVAVEIDYVRVALKDWKKIQDRARSALKTSCRRPPAPERLPATRR 291

lez:GLE_5040 MLPSCSHSFLMPRYGANVRVSYQRIFVRDWKRIEQRIREIFDNGYLGDAPAR------- 284

* * : : * * : **: :: : :
