# Supplementary material for: The opportunistic pathogen Stenotrophomonas maltophilia utilizes a type IV secretion system for interbacterial killing
Source: PLoS Pathog. 2019 Sep 12;15(9):e1007651. doi: 10.1371/journal.ppat.1007651 (PMC6759196; doi:10.1371/journal.ppat.1007651)
Supplement: S4 Fig — (DOCX) [file ppat.1007651.s008.docx]

**S4 Fig. Smlt3024 homologs greater than 750 residues in length identified by PSI-BLAST**

Three iterations of PSI-BLAST using the first 308 residues of Smlt3024 gave 815 hits with e-values < e^-6^, the first 402 of which are proteins of less than 600 amino acids, most between 400 and 450 residues in length, similar to that of Smlt3024 (not shown). The PSI-BLAST search also returned 221 hits with lower sequence identity and similarity (e-values between e^-43^ and e^-7^) with proteins larger than 750 amino acids derived from a wide variety of bacterial genera, including *Yersinia, Ralstonia, Pseudomonas, Cupriavidus, Snodgrassella, Xanthomonas, Pseudoxanthomonas, Leisingera, Thalassospira, Nitrosomonas, Halocynthiibacter, Vibrio, Neisseria, Thioalkalivibrio, Stenotrophomonas, Rhizobium, Robbsia, Devosia, Sphingomonas, Paraburkholderia, Sphingomonas* and *Acinetobacter*. This group of 221 proteins (shown below) share the following characteristics: i) all except one align with Smlt3024 via their N-terminal regions (within the first 300 amino acids), ii) all but six have multiple downstream RTX calcium-binding nonapeptide motifs (PF00353) and many also carry hemolysin type calcium binding protein related domains (PF06594). Some have additional C-terminal domains such as peptidase S8, subtilisin-like, pro-protein convertase P, cadherin-like and IgG-like domains.

>TMU18711.1 serine protease [Yersinia sp. KBS0713]

Length=1815

Score = 170 bits (429), Expect = 2e-43, Method: Composition-based stats.

Identities = 48/231 (21%), Positives = 83/231 (36%), Gaps = 31/231 (13%)

Query 4 LTERDL----SVLGSYARDGNRELYWNYLSQLPGADGYGTLALGVVRNDSLPGRVANTYA 59

LT +L +L S+ + +NYL+ D Y LA GVVR DS G +A Y

Sbjct 13 LTATELACAREILESHKESKDPGPMYNYLASK--GDRYAVLANGVVRGDSFAGAMAIYYM 70

Query 60 QDYAKSQQEEGSRFPNAQLTERQWESFGQTLLERDLELRQQWMNERRPDLALNLPGKDVM 119

+ A + +TE + ++ + L Q ++ + ++ K+

Sbjct 71 ERTAAEHNK--------PITEVRLKNIRYDMAGGYLNALQNRLDISTGTIYGDINHKEAG 122

Query 120 LAHDRAFERHELDPNCWTPRVLLQAAEQKSGPAKLEQI-----------------WTNML 162

H+ F+RH L P WT + A ++ S P + + + M

Sbjct 123 QFHEIVFKRHGLPPETWTLEPVFNAIQETSRPTYWQHVLNAAGKPAKELKLSFETYNMMA 182

Query 163 NNDYAGGPRVGNTSVDAISQMGWTKGGQYLTRLSVLEATQALEGRSAVDPN 213

+ + TS S + G L + + + A + S V P

Sbjct 183 QSSMFSPEAIRKTSRRWFSIIDSPSGYWALGKTATNQLFSADDETSIVIPQ 233

>WP_005278009.1 serine protease [Yersinia bercovieri]

EEQ05433.1 serine protease [Yersinia bercovieri ATCC 43970]

PHZ25940.1 serine protease [Yersinia bercovieri]

Length=1815

Score = 169 bits (428), Expect = 3e-43, Method: Composition-based stats.

Identities = 48/231 (21%), Positives = 83/231 (36%), Gaps = 31/231 (13%)

Query 4 LTERDL----SVLGSYARDGNRELYWNYLSQLPGADGYGTLALGVVRNDSLPGRVANTYA 59

LT +L +L S+ + +NYL+ D Y LA GVVR DS G +A Y

Sbjct 13 LTATELACAREILESHKESKDPGPMYNYLASK--GDRYAVLANGVVRGDSFAGAMAIYYM 70

Query 60 QDYAKSQQEEGSRFPNAQLTERQWESFGQTLLERDLELRQQWMNERRPDLALNLPGKDVM 119

+ A + +TE + ++ + L Q ++ + ++ K+

Sbjct 71 ERTAAEHNK--------PITEVRLKNIRYDMAGGYLNALQNRLDISTGTIYGDINHKEAG 122

Query 120 LAHDRAFERHELDPNCWTPRVLLQAAEQKSGPAKLEQI-----------------WTNML 162

H+ F+RH L P WT + A ++ S P + + + M

Sbjct 123 QFHEIVFKRHGLPPETWTLEPVFNAIQETSRPTYWQHVLNAAGKPAKELKLSFETYNMMA 182

Query 163 NNDYAGGPRVGNTSVDAISQMGWTKGGQYLTRLSVLEATQALEGRSAVDPN 213

+ + TS S + G L + + + A + S V P

Sbjct 183 QSSMFSPEAIRKTSRRWFSIIDSPSGYWALGKTATNQLFSADDETSIVIPQ 233

>WP_049599355.1 serine protease [Yersinia bercovieri]

CNH87945.1 putative serine protease [Yersinia bercovieri]

Length=1815

Score = 169 bits (427), Expect = 3e-43, Method: Composition-based stats.

Identities = 47/231 (20%), Positives = 81/231 (35%), Gaps = 31/231 (13%)

Query 4 LTERDL----SVLGSYARDGNRELYWNYLSQLPGADGYGTLALGVVRNDSLPGRVANTYA 59

LT +L +L S+ + +NYL+ D Y LA GV R DS G +A Y

Sbjct 13 LTATELACAREILESHKESKDPGPMYNYLASK--GDRYAVLANGVARGDSFAGAMAIYYM 70

Query 60 QDYAKSQQEEGSRFPNAQLTERQWESFGQTLLERDLELRQQWMNERRPDLALNLPGKDVM 119

+ A + +TE + ++ + L Q ++ + ++ K+

Sbjct 71 ERTAAEHNK--------PITEVRLKNIRYDMAGGYLNALQNRLDISTGTIYGDINHKEAG 122

Query 120 LAHDRAFERHELDPNCWTPRVLLQAAEQKSGPAKLEQI-----------------WTNML 162

H+ F+RH L P WT + A E+ S P + + + M

Sbjct 123 QFHEIVFKRHGLPPETWTLEPVFNAIEETSRPTYWQHVLNAAGKPTEELKLSFETYNMMA 182

Query 163 NNDYAGGPRVGNTSVDAISQMGWTKGGQYLTRLSVLEATQALEGRSAVDPN 213

+ + TS S + G L + + + + S V P

Sbjct 183 QSSMFSPEAIRKTSRRWFSIIDSPSGYWALGKTATNQLFSTDDQISIVIPQ 233

>WP_057647018.1 serine protease [Yersinia enterocolitica]

CFQ29672.1 putative serine protease [Yersinia enterocolitica]

Length=1815

Score = 167 bits (422), Expect = 2e-42, Method: Composition-based stats.

Identities = 50/231 (22%), Positives = 84/231 (36%), Gaps = 31/231 (13%)

Query 4 LTERDL----SVLGSYARDGNRELYWNYLSQLPGADGYGTLALGVVRNDSLPGRVANTYA 59

LT DL +L S+ + + +NYL+ D Y LA GVVR DS G +A Y

Sbjct 13 LTATDLVCAREILESHKQSKDPGPMYNYLASK--GDRYAVLANGVVRGDSFAGAMAIYYM 70

Query 60 QDYAKSQQEEGSRFPNAQLTERQWESFGQTLLERDLELRQQWMNERRPDLALNLPGKDVM 119

+ A + LTE Q + + +E+ Q ++ + ++ K+

Sbjct 71 ERTAADHNK--------PLTEAQIKDIRFDMAGEYIEVLQNRLDISTGTIYGDINHKEAG 122

Query 120 LAHDRAFERHELDPNCWTPRVLLQAAEQKSGPAKLEQI-----------------WTNML 162

H+ F+RH L WT + A ++ S P + + + M

Sbjct 123 QFHEIVFKRHGLPSETWTLEPVFNAIQETSRPTYWQHVLNAAGKPAKELKLSFETYNMMA 182

Query 163 NNDYAGGPRVGNTSVDAISQMGWTKGGQYLTRLSVLEATQALEGRSAVDPN 213

+ + TS S + G L + + + A + S V P

Sbjct 183 QSSMFSPEAIRKTSRRWFSIIDSPSGYWALGKTATNQLFSADDETSIVIPQ 233

>CNK01351.1 putative serine protease [Yersinia enterocolitica]

Length=1814

Score = 166 bits (419), Expect = 4e-42, Method: Composition-based stats.

Identities = 51/267 (19%), Positives = 90/267 (34%), Gaps = 30/267 (11%)

Query 4 LTERDLSVLGSYAR----DGNRELYWNYLSQLPGADGYGTLALGVVRNDSLPGRVANTYA 59

L DL+ + +++L+ D Y LA GVVR DS+ G +A Y

Sbjct 13 LNAADLACARQLLEANKESKDPGPMYDFLASK--GDRYAILANGVVRGDSIAGAMAIHYL 70

Query 60 QDYAKSQQEEGSRFPNAQLTERQWESFGQTLLERDLELRQQWMNERRPDL-ALNLPGKDV 118

+ A S N +T+ + L +Q+ +++ + ++ K

Sbjct 71 ESVAASH--------NQPITDIGLNDIRYDMAHGYLNTQQKRLDDSPTGIIYGDIDHKQA 122

Query 119 MLAHDRAFERHELDPNCWTPRVLLQAAEQKSGPAKLEQIWTNMLNNDYAGGPRVGNTSVD 178

H+ F H L WT + +A +++S P W +L N SVD

Sbjct 123 TQFHNSVFNDHGLPSKAWTLDQVFKAIDERSQPI----YWQWVL-NAAGKPKEELRLSVD 177

Query 179 AISQMGWTKGGQYLTRLSVLEATQALEGRSAVDP----NVIGGNSYYAMYFEADRKWASI 234

+M + + E VD + +S ++ S

Sbjct 178 TYQKMALSAKVA-----PDGIRQSSREWFDRVDSLPGYWALAKSSTSQLFSSDQTTAVSG 232

Query 235 SAGGGHMSLREITDP-SRIAELNDARE 260

+++ P RIA+ + AR

Sbjct 233 QECNFDINISPTPQPVQRIADEDQARR 259

>WP_050336502.1 serine protease [Yersinia enterocolitica]

CRY15757.1 putative serine protease [Yersinia enterocolitica]

Length=1803

Score = 166 bits (419), Expect = 4e-42, Method: Composition-based stats.

Identities = 51/267 (19%), Positives = 90/267 (34%), Gaps = 30/267 (11%)

Query 4 LTERDLSVLGSYAR----DGNRELYWNYLSQLPGADGYGTLALGVVRNDSLPGRVANTYA 59

L DL+ + +++L+ D Y LA GVVR DS+ G +A Y

Sbjct 13 LNAADLACARQLLEANKESKDPGPMYDFLASK--GDRYAILANGVVRGDSIAGAMAIHYL 70

Query 60 QDYAKSQQEEGSRFPNAQLTERQWESFGQTLLERDLELRQQWMNERRPDL-ALNLPGKDV 118

+ A S N +T+ + L +Q+ +++ + ++ K

Sbjct 71 ESVAASH--------NQPITDIGLNDIRYDMAHGYLNTQQKRLDDSPTGIIYGDIDHKQA 122

Query 119 MLAHDRAFERHELDPNCWTPRVLLQAAEQKSGPAKLEQIWTNMLNNDYAGGPRVGNTSVD 178

H+ F H L WT + +A +++S P W +L N SVD

Sbjct 123 TQFHNSVFNDHGLPSKAWTLDQVFKAIDERSQPI----YWQWVL-NAAGKPKEELRLSVD 177

Query 179 AISQMGWTKGGQYLTRLSVLEATQALEGRSAVDP----NVIGGNSYYAMYFEADRKWASI 234

+M + + E VD + +S ++ S

Sbjct 178 TYQKMALSAKVA-----PDGIRQSSREWFDRVDSLPGYWALAKSSTSQLFSSDQTTAVSG 232

Query 235 SAGGGHMSLREITDP-SRIAELNDARE 260

+++ P RIA+ + AR

Sbjct 233 QECNFDINISPTPQPVQRIADEDQARR 259

>PNM08967.1 serine protease [Yersinia enterocolitica]

Length=1803

Score = 166 bits (419), Expect = 5e-42, Method: Composition-based stats.

Identities = 51/267 (19%), Positives = 90/267 (34%), Gaps = 30/267 (11%)

Query 4 LTERDLSVLGSYAR----DGNRELYWNYLSQLPGADGYGTLALGVVRNDSLPGRVANTYA 59

L DL+ + +++L+ D Y LA GVVR DS+ G +A Y

Sbjct 13 LNAADLACARQLLEANKESKDPGPMYDFLASK--GDRYAILANGVVRGDSIAGAMAIHYL 70

Query 60 QDYAKSQQEEGSRFPNAQLTERQWESFGQTLLERDLELRQQWMNERRPDL-ALNLPGKDV 118

+ A S N +T+ + L +Q+ +++ + ++ K

Sbjct 71 ESVAASH--------NQPITDIGLNDIRYDMAHGYLNTQQKRLDDSPTGIIYGDIDHKQA 122

Query 119 MLAHDRAFERHELDPNCWTPRVLLQAAEQKSGPAKLEQIWTNMLNNDYAGGPRVGNTSVD 178

H+ F H L WT + +A +++S P W +L N SVD

Sbjct 123 TQFHNSVFNDHGLPSKAWTLDQVFKAIDERSQPI----YWQWVL-NAAGKPKEELRLSVD 177

Query 179 AISQMGWTKGGQYLTRLSVLEATQALEGRSAVDP----NVIGGNSYYAMYFEADRKWASI 234

+M + + E VD + +S ++ S

Sbjct 178 TYQKMALSAKVA-----PDGIRQSSREWFDRVDSLPGYWALAKSSTSQLFSSDQTTAVSG 232

Query 235 SAGGGHMSLREITDP-SRIAELNDARE 260

+++ P RIA+ + AR

Sbjct 233 QECNFDINISPTPQPVQRIADEDQARR 259

>WP_054872875.1 serine protease [Yersinia bercovieri]

CNF50710.1 putative serine protease [Yersinia bercovieri]

Length=1816

Score = 164 bits (415), Expect = 1e-41, Method: Composition-based stats.

Identities = 48/232 (21%), Positives = 83/232 (36%), Gaps = 32/232 (14%)

Query 4 LTERDL----SVLGSYARDGNRELYWNYLSQLPGADGYGTLALGVVRNDSLPGRVANTYA 59

LT +L +L S+ + +NYL+ D Y LA GVVR DSL G +A Y

Sbjct 13 LTATELACAREILESHKESKDPGPMYNYLASK--GDRYAVLANGVVRGDSLAGAMAIYYM 70

Query 60 QDYAKSQQEEGSRFPNAQLTERQWESFGQTLLERDLELRQQWMNERRPDL-ALNLPGKDV 118

+ A + +TE ++ + L+ + + + ++ K+

Sbjct 71 ERTAADHNK--------PITEVHLKNIRFDMAGGYLDTLDSRLKKSSDGILYGDINHKEA 122

Query 119 MLAHDRAFERHELDPNCWTPRVLLQAAEQKSGPAKLEQI-----------------WTNM 161

H+ F+RH L P WT + A ++ S P + + + M

Sbjct 123 WKFHNEEFKRHGLPPETWTLDPVFNAIQETSRPTYWQHVLNAAGKPTEELKLSFETYNMM 182

Query 162 LNNDYAGGPRVGNTSVDAISQMGWTKGGQYLTRLSVLEATQALEGRSAVDPN 213

+ + TS S + G L + + + A + S V P

Sbjct 183 AQSSMFSPEAIRKTSRRWFSIIDSPSGYWALGKTATNQLFSADDETSIVIPQ 234

>WP_004875327.1 serine protease [Yersinia mollaretii]

EEQ10554.1 serine protease [Yersinia mollaretii ATCC 43969]

Length=1817

Score = 163 bits (413), Expect = 3e-41, Method: Composition-based stats.

Identities = 57/270 (21%), Positives = 105/270 (39%), Gaps = 30/270 (11%)

Query 4 LTERDLS----VLGSYARDGNRELYWNYLSQLPGADGYGTLALGVVRNDSLPGRVANTYA 59

L DL+ +L S+ + +++L D Y TLA GV R +S+ G +A Y

Sbjct 13 LNAADLACARQLLESHQESKDPGPMYDFLISKE--DRYATLANGVARGNSIAGGMAIHYL 70

Query 60 QDYAKSQQEEGSRFPNAQLTERQWESFGQTLLERDLELRQQWMNERRPDLALNLPGKDVM 119

+ A S N +TE Q + L+++Q +++ + ++ K

Sbjct 71 ESVAASH--------NQPITEGQLNDIRFAMARGYLDMQQGRLDKSGDTIYGDINHKQAA 122

Query 120 LAHDRAFERHELDPNCWTPRVLLQAAEQKSGPAKLEQIWTNMLNNDYAGGPRVGNTSVDA 179

L H + FE+ L P WT + +A ++KS P EQ+ N SVD

Sbjct 123 LFHKKVFEKFRLPPEAWTLDPVFKAIDEKSRPVYWEQV-----LNAAGKPVEELKLSVDT 177

Query 180 ISQMGWTKGGQYLTRLSVLEATQALEGRSAVDP----NVIGGNSYYAMYFEADRKWASIS 235

+M L + +++Q E + VD +G ++ ++ D +I

Sbjct 178 YQKMAL---SSKLAPDEIQKSSQ--EWFARVDSPSGYWALGKSTTSQLFTTPDETPIAIP 232

Query 236 AGGGHMSLREITDPSRIAELNDAREVRLER 265

+++ P + D +V+ +

Sbjct 233 QCNIDINITPT--PQATQRITDEDQVQRDV 260

>WP_050142163.1 serine protease [Yersinia enterocolitica]

CNK32175.1 putative serine protease [Yersinia enterocolitica]

Length=1817

Score = 163 bits (412), Expect = 3e-41, Method: Composition-based stats.

Identities = 56/270 (21%), Positives = 105/270 (39%), Gaps = 30/270 (11%)

Query 4 LTERDLS----VLGSYARDGNRELYWNYLSQLPGADGYGTLALGVVRNDSLPGRVANTYA 59

L DL+ +L S+ + +++L D Y TLA GV R +S+ G +A Y

Sbjct 13 LNAADLACARQLLESHQASKDPGPMYDFLISKE--DRYATLANGVARGNSIAGGMAIHYL 70

Query 60 QDYAKSQQEEGSRFPNAQLTERQWESFGQTLLERDLELRQQWMNERRPDLALNLPGKDVM 119

+ A S N +TE Q + L+++Q+ +++ + ++ +

Sbjct 71 ESVAASH--------NQPITEGQLNDIRFAMARGYLDMQQRRLDDSPGTIYGDINHEQAA 122

Query 120 LAHDRAFERHELDPNCWTPRVLLQAAEQKSGPAKLEQIWTNMLNNDYAGGPRVGNTSVDA 179

L H+R F L P WT + +A ++KS P EQ+ N SVD

Sbjct 123 LFHNRIFNEFGLPPKAWTLTPVFKAIDEKSRPVYWEQV-----LNAAGKPVEELKLSVDT 177

Query 180 ISQMGWTKGGQYLTRLSVLEATQALEGRSAVDP----NVIGGNSYYAMYFEADRKWASIS 235

+M L + +++Q E + VD +G ++ ++ D +I

Sbjct 178 YQKMAL---SSKLAPDEIQKSSQ--EWFARVDSPSGYWALGKSATSQLFTTPDETPIAIP 232

Query 236 AGGGHMSLREITDPSRIAELNDAREVRLER 265

+++ P + D +V+ +

Sbjct 233 QCNIDINITPT--PQATQRITDEDQVQRDV 260

>WP_050535946.1 serine protease [Yersinia mollaretii]

CNI95017.1 putative serine protease [Yersinia mollaretii]

CQQ14065.1 putative serine protease [Yersinia mollaretii]

Length=1817

Score = 163 bits (411), Expect = 5e-41, Method: Composition-based stats.

Identities = 55/270 (20%), Positives = 99/270 (37%), Gaps = 30/270 (11%)

Query 4 LTERDLSVLGSYARD----GNRELYWNYLSQLPGADGYGTLALGVVRNDSLPGRVANTYA 59

L DL+ + +++L D Y TLA GV R +S+ G +A Y

Sbjct 13 LNAADLACARQLLESHQVSKDPGPMYDFLISKE--DRYATLANGVARGNSIAGGMAIHYL 70

Query 60 QDYAKSQQEEGSRFPNAQLTERQWESFGQTLLERDLELRQQWMNERRPDLALNLPGKDVM 119

+ A S N +TE Q + L+++Q ++ + ++ K

Sbjct 71 ESVAASH--------NQPITEGQLNDIRFAMAHGYLDMQQIRLDGSVGTIYGDINHKQAA 122

Query 120 LAHDRAFERHELDPNCWTPRVLLQAAEQKSGPAKLEQIWTNMLNNDYAGGPRVGNTSVDA 179

L H FE+ L P WT + +A ++KS P EQ+ N SVD

Sbjct 123 LFHKIVFEKFGLPPEAWTLDPVFKAIDEKSRPVYWEQV-----LNAAGKPVEELKLSVDT 177

Query 180 ISQMGWTKGGQYLTRLSVLEATQALEGRSAVDP----NVIGGNSYYAMYFEADRKWASIS 235

+M L + +++Q E + VD +G ++ ++ D +I

Sbjct 178 YQKMAL---SSKLAPDEIQKSSQ--EWFARVDSPSGYWALGKSATSQLFTTPDETPIAIP 232

Query 236 AGGGHMSLREITDPSRIAELNDAREVRLER 265

+++ P + D +V+ +

Sbjct 233 QCNIDINITPT--PQATQRITDEDQVQRDV 260

>WP_050540422.1 serine protease [Yersinia mollaretii]

PJE89792.1 serine protease [Yersinia mollaretii]

CQD40556.1 putative serine protease [Yersinia mollaretii]

Length=1817

Score = 161 bits (407), Expect = 2e-40, Method: Composition-based stats.

Identities = 54/265 (20%), Positives = 97/265 (37%), Gaps = 28/265 (11%)

Query 4 LTERDLS----VLGSYARDGNRELYWNYLSQLPGADGYGTLALGVVRNDSLPGRVANTYA 59

L DL+ +L S+ + +++L D Y TLA GV R +S+ G +A Y

Sbjct 13 LNAADLACARQLLESHKESKDPGPMYDFLISKE--DRYATLANGVARGNSIAGGMAIHYL 70

Query 60 QDYAKSQQEEGSRFPNAQLTERQWESFGQTLLERDLELRQQWMNERRPDLALNLPGKDVM 119

+ A S N +TE Q + L+++Q ++ + ++ K

Sbjct 71 ESVAASH--------NQPITEGQLNDIRFAMAHGYLDMQQIRLDGSVGTIYGDINHKQAA 122

Query 120 LAHDRAFERHELDPNCWTPRVLLQAAEQKSGPAKLEQIWTNMLNNDYAGGPRVGNTSVDA 179

L H FE+ L P WT + +A ++KS P EQ+ N SVD

Sbjct 123 LFHKIVFEKFGLPPEAWTLDPVFKAIDEKSRPMYWEQV-----LNAAGKPVEELKLSVDI 177

Query 180 ISQMGWTKGGQYLTRLSVLEATQALEGRSAVDP----NVIGGNSYYAMYFEADRKWASIS 235

+M L + +++Q E + VD +G + ++ D

Sbjct 178 YQKMAL---SSKLAPDEIQKSSQ--EWFARVDSPSGYWALGKGATSQLFTTPDETPIDTP 232

Query 236 AGGGHMSLREITDPSRIAELNDARE 260

+++ ++ D +

Sbjct 233 QCNIDINITPTPQATQRITDEDQAQ 257

>WP_050538857.1 serine protease [Yersinia mollaretii]

CQH26655.1 putative serine protease [Yersinia mollaretii]

Length=1817

Score = 161 bits (406), Expect = 2e-40, Method: Composition-based stats.

Identities = 54/265 (20%), Positives = 97/265 (37%), Gaps = 28/265 (11%)

Query 4 LTERDLS----VLGSYARDGNRELYWNYLSQLPGADGYGTLALGVVRNDSLPGRVANTYA 59

L DL+ +L S+ + +++L D Y TLA GV R +S+ G +A Y

Sbjct 13 LNAADLACARQLLESHKESKDPGPMYDFLISKE--DRYATLANGVARGNSIAGGMAIHYL 70

Query 60 QDYAKSQQEEGSRFPNAQLTERQWESFGQTLLERDLELRQQWMNERRPDLALNLPGKDVM 119

+ A S N +TE Q + L+++Q ++ + ++ K

Sbjct 71 ESVAASH--------NQPITEGQLNDIRFAMAHGYLDMQQIRLDGSVGTIYGDINHKQAA 122

Query 120 LAHDRAFERHELDPNCWTPRVLLQAAEQKSGPAKLEQIWTNMLNNDYAGGPRVGNTSVDA 179

L H FE+ L P WT + +A ++KS P EQ+ N SVD

Sbjct 123 LFHKIVFEKFGLPPEAWTLDPVFKAIDEKSRPMYWEQV-----LNAAGKPVEELKLSVDI 177

Query 180 ISQMGWTKGGQYLTRLSVLEATQALEGRSAVDP----NVIGGNSYYAMYFEADRKWASIS 235

+M L + +++Q E + VD +G + ++ D

Sbjct 178 YQKMAL---SSKLAPDEIQKSSQ--EWFARVDSPSGYWALGKGATSQLFTTPDETPIDTP 232

Query 236 AGGGHMSLREITDPSRIAELNDARE 260

+++ ++ D +

Sbjct 233 QCNIDINITPTPQATQRITDEDQAQ 257

>WP_050289612.1 serine protease [Yersinia kristensenii]

CND94226.1 putative serine protease [Yersinia kristensenii]

Length=1813

Score = 161 bits (406), Expect = 2e-40, Method: Composition-based stats.

Identities = 50/267 (19%), Positives = 95/267 (36%), Gaps = 30/267 (11%)

Query 4 LTERDLS----VLGSYARDGNRELYWNYLSQLPGADGYGTLALGVVRNDSLPGRVANTYA 59

L DL+ +L ++ + +++L+ D Y LA GVVR DS+ G +A Y

Sbjct 13 LNAADLACARQLLEAHNESKDPGPMYDFLASK--GDRYAILANGVVRGDSIAGAMAIHYL 70

Query 60 QDYAKSQQEEGSRFPNAQLTERQWESFGQTLLERDLELRQQWMNERRPDL-ALNLPGKDV 118

+ A S N +T+ + L+ +Q +++ + ++ +

Sbjct 71 ESVAASH--------NQPITDIGLNDIRYDMALGYLDTQQSRLDKSPTGIIYGDIGHEQA 122

Query 119 MLAHDRAFERHELDPNCWTPRVLLQAAEQKSGPAKLEQIWTNMLNNDYAGGPRVGNTSVD 178

H+R F H L WT + +A +++S P W +LN + SVD

Sbjct 123 TQFHNRVFNDHGLPSKAWTLDQVFKAIDERSQPI----YWQWVLNAAGKPKEELW-LSVD 177

Query 179 AISQMGWTKGGQYLTRLSVLEATQALEGRSAVDP----NVIGGNSYYAMYFEADRKWASI 234

+M + + E VD + +S ++ +

Sbjct 178 TYQKMALSAKVA-----PDGIRESSREWFDRVDSLSGYWALAKSSTSQLFSSDQTTAVAG 232

Query 235 SAGGGHMSLREITDP-SRIAELNDARE 260

+++ RIA+ + AR

Sbjct 233 QECNIDINITPTPQAVQRIADEDQARR 259

>WP_050941754.1 serine protease [Yersinia enterocolitica]

CRY17340.1 putative serine protease [Yersinia enterocolitica]

Length=1803

Score = 160 bits (404), Expect = 4e-40, Method: Composition-based stats.

Identities = 52/267 (19%), Positives = 93/267 (35%), Gaps = 30/267 (11%)

Query 4 LTERDLSVLGSYAR----DGNRELYWNYLSQLPGADGYGTLALGVVRNDSLPGRVANTYA 59

L DL+ + +++L+ D Y LA GVVR DS+ G +A Y

Sbjct 13 LNAADLACARQLLEANKESKDPGPMYDFLASK--GDRYAILANGVVRGDSIAGAMAIHYL 70

Query 60 QDYAKSQQEEGSRFPNAQLTERQWESFGQTLLERDLELRQQWMNERRPDL-ALNLPGKDV 118

+ A S N +TE + + + L +Q+ +++ + ++ K

Sbjct 71 ESVAASH--------NQPITEMRLKHIRYDMAHGYLNTQQKRLDDSPTGIIYGDIDHKQA 122

Query 119 MLAHDRAFERHELDPNCWTPRVLLQAAEQKSGPAKLEQIWTNMLNNDYAGGPRVGNTSVD 178

H+ +RH L WT + +A +++S P W +L N SVD

Sbjct 123 GQFHNEELKRHGLPSKAWTLDPVFRAIDERSQPI----YWQWVL-NAAGKPKEELRLSVD 177

Query 179 AISQMGWTKGGQYLTRLSVLEATQALEGRSAVDP----NVIGGNSYYAMYFEADRKWASI 234

+M + + E VD + +S ++ S

Sbjct 178 TYQKMALSAKVA-----PDGIRQSSREWFDRVDSLSGYWALAKSSTSQLFSSDQTTAVSG 232

Query 235 SAGGGHMSLREITDP-SRIAELNDARE 260

+++ P RIA+ + AR

Sbjct 233 QECNFDINISPTPQPVQRIADEDQARR 259

>WP_049606695.1 serine protease [Yersinia mollaretii]

CNK03077.1 putative serine protease [Yersinia mollaretii]

Length=1817

Score = 160 bits (404), Expect = 4e-40, Method: Composition-based stats.

Identities = 50/266 (19%), Positives = 100/266 (38%), Gaps = 30/266 (11%)

Query 4 LTERDLS----VLGSYARDGNRELYWNYLSQLPGADGYGTLALGVVRNDSLPGRVANTYA 59

L DL+ +L S+ + +++L D Y TLA GV R +S+ G +A Y

Sbjct 13 LNAADLACARQLLESHQASKDPGPMYDFLISKE--DRYATLANGVARGNSIAGGMAIHYL 70

Query 60 QDYAKSQQEEGSRFPNAQLTERQWESFGQTLLERDLELRQQWMNERRPDLALNLPGKDVM 119

+ A S N +TE Q + L+++Q+ +++ + ++ +

Sbjct 71 ESVAASH--------NQPITEGQLNDIRFAMARGYLDMQQRRLDDSPGTIYGDINHEQAA 122

Query 120 LAHDRAFERHELDPNCWTPRVLLQAAEQKSGPAKLEQIWTNMLNNDYAGGPRVGNTSVDA 179

L H+R F L P WT + +A ++KS P EQ+ G +

Sbjct 123 LFHNRIFNEFGLPPKAWTLTPVFKAIDEKSRPVYWEQV-----------LNAAGKPVEEL 171

Query 180 ISQMGWTKGGQYLTRLSVLEA-TQALEGRSAVDP----NVIGGNSYYAMYFEADRKWASI 234

+ + ++L+ E + E + VD +G ++ ++ D +I

Sbjct 172 KLSVDTYQKMALSSKLAPDEIQKSSREWFARVDSPSGYWALGKSATSQLFTPPDETPIAI 231

Query 235 SAGGGHMSLREITDPSRIAELNDARE 260

+++ ++ D +

Sbjct 232 PQCNIDINITPTPQATQRITDEDQAQ 257

>WP_011815969.1 serine protease [Yersinia enterocolitica]

YP_001005702.1 serine protease [Yersinia enterocolitica subsp. enterocolitica

8081]

AJJ25026.1 proconvertase P-domain protein [Yersinia enterocolitica]

CAL11481.1 putative serine protease [Yersinia enterocolitica subsp. enterocolitica

8081]

Length=1803

Score = 160 bits (404), Expect = 4e-40, Method: Composition-based stats.

Identities = 52/267 (19%), Positives = 93/267 (35%), Gaps = 30/267 (11%)

Query 4 LTERDLSVLGSYAR----DGNRELYWNYLSQLPGADGYGTLALGVVRNDSLPGRVANTYA 59

L DL+ + +++L+ D Y LA GVVR DS+ G +A Y

Sbjct 13 LNAADLACARQLLEANKESKDPGPMYDFLASK--GDRYAILANGVVRGDSIAGAMAIHYL 70

Query 60 QDYAKSQQEEGSRFPNAQLTERQWESFGQTLLERDLELRQQWMNERRPDL-ALNLPGKDV 118

+ A S N +TE + + + L +Q+ +++ + ++ K

Sbjct 71 ESVAASH--------NQPITEMRLKHIRYDMAHGYLNTQQKRLDDSPTGIIYGDIDHKQA 122

Query 119 MLAHDRAFERHELDPNCWTPRVLLQAAEQKSGPAKLEQIWTNMLNNDYAGGPRVGNTSVD 178

H+ +RH L WT + +A +++S P W +L N SVD

Sbjct 123 GQFHNEELKRHGLPSKAWTLDPVFRAIDERSQPI----YWQWVL-NAAGKPKEELRLSVD 177

Query 179 AISQMGWTKGGQYLTRLSVLEATQALEGRSAVDP----NVIGGNSYYAMYFEADRKWASI 234

+M + + E VD + +S ++ S

Sbjct 178 TYQKMALSAKVA-----PDGIRQSSREWFDRVDSLSGYWALAKSSTSQLFSSDQTTAVSG 232

Query 235 SAGGGHMSLREITDP-SRIAELNDARE 260

+++ P RIA+ + AR

Sbjct 233 QECNFDINISPTPQPVQRIADEDQARR 259

>WP_023160798.1 serine protease [Yersinia enterocolitica]

CCV46917.1 putative serine protease [Yersinia enterocolitica (type O:5,27)

str. YE149/02]

CFQ65486.1 putative serine protease [Yersinia enterocolitica]

CNC03673.1 putative serine protease [Yersinia enterocolitica]

CND92659.1 putative serine protease [Yersinia enterocolitica]

CNI33763.1 putative serine protease [Yersinia enterocolitica]

CNJ46589.1 putative serine protease [Yersinia enterocolitica]

CQD42145.1 putative serine protease [Yersinia enterocolitica]

CQD70919.1 putative serine protease [Yersinia enterocolitica]

CQH37979.1 putative serine protease [Yersinia enterocolitica]

CQH83906.1 putative serine protease [Yersinia enterocolitica]

CQH85507.1 putative serine protease [Yersinia enterocolitica]

CQH86555.1 putative serine protease [Yersinia enterocolitica]

CQH98788.1 putative serine protease [Yersinia enterocolitica]

CQQ73367.1 putative serine protease [Yersinia enterocolitica]

CRX96316.1 putative serine protease [Yersinia enterocolitica]

Length=1806

Score = 160 bits (403), Expect = 6e-40, Method: Composition-based stats.

Identities = 52/269 (19%), Positives = 102/269 (38%), Gaps = 33/269 (12%)

Query 4 LTERDLS----VLGSYARDGNRELYWNYLSQLPGADGYGTLALGVVRNDSLPGRVANTYA 59

L DL+ +L S+ N +++L+ D Y LA GV R DS+ G +A Y

Sbjct 13 LNAADLACARQLLESHQESKNPGPMYDFLASK--GDRYAVLANGVARGDSIAGAMAIHYL 70

Query 60 QDYAKSQQEEGSRFPNAQLTERQWESFGQTLLERDLELRQQWMNERR-PDLALNLPGKDV 118

+ A+S + +TE + + + L ++Q ++E + ++ +

Sbjct 71 ESVAESH--------DRPITETRLNNIRYDMARGYLAMQQSRLDENPVGIIYGDINHEQA 122

Query 119 MLAHDRAFERHELDPNCWTPRVLLQAAEQKSGPAKLEQIWTNMLNNDYAGGPRVGNTSVD 178

H+RAF H L WT +L+A ++KS PA EQ+ G +

Sbjct 123 TWFHNRAFRDHRLPSKAWTLDAVLRAIDEKSRPAYWEQV-----------LNAAGKPKEE 171

Query 179 AISQMGWTKGGQYLTRLSVLEATQ-ALEGRSAVDP----NVIGGNSYYAMYFEADRKWAS 233

+ + ++ Q + E VD + +S ++ + S

Sbjct 172 LLLSADTYQKMALSAKVGPDGIQQSSREWFDRVDSLAGYWALVKSSSSQLFSSDEAAALS 231

Query 234 ISAGGGHMSLREITDPSRIAELNDAREVR 262

+ ++++ P + + D +V+

Sbjct 232 AAECNFNINISAT--PQTVERVADEDQVQ 258

>WP_050153043.1 serine protease [Yersinia enterocolitica]

CNI05012.1 putative serine protease [Yersinia enterocolitica]

Length=1806

Score = 160 bits (403), Expect = 6e-40, Method: Composition-based stats.

Identities = 52/269 (19%), Positives = 102/269 (38%), Gaps = 33/269 (12%)

Query 4 LTERDLS----VLGSYARDGNRELYWNYLSQLPGADGYGTLALGVVRNDSLPGRVANTYA 59

L DL+ +L S+ N +++L+ D Y LA GV R DS+ G +A Y

Sbjct 13 LNAADLACARQLLESHQESKNPGPMYDFLASK--GDRYAVLANGVARGDSIAGAMAIHYL 70

Query 60 QDYAKSQQEEGSRFPNAQLTERQWESFGQTLLERDLELRQQWMNERR-PDLALNLPGKDV 118

+ A+S + +TE + + + L ++Q ++E + ++ +

Sbjct 71 ESVAESH--------DRPITETRLNNIRYDMARGYLAMQQSRLDENPVGIIYGDINHEQA 122

Query 119 MLAHDRAFERHELDPNCWTPRVLLQAAEQKSGPAKLEQIWTNMLNNDYAGGPRVGNTSVD 178

H+RAF H L WT +L+A ++KS PA EQ+ G +

Sbjct 123 TWFHNRAFRDHRLPSKAWTLDAVLRAIDEKSRPAYWEQV-----------LNAAGKPKEE 171

Query 179 AISQMGWTKGGQYLTRLSVLEATQ-ALEGRSAVDP----NVIGGNSYYAMYFEADRKWAS 233

+ + ++ Q + E VD + +S ++ + S

Sbjct 172 LLLSADTYQKMALSAKVGPDGIQQSSREWFDRVDSLAGYWALVKSSSSQLFSSDEAAALS 231

Query 234 ISAGGGHMSLREITDPSRIAELNDAREVR 262

+ ++++ P + + D +V+

Sbjct 232 AAECNFNINISAT--PQTVERVADEDQVQ 258

>CQJ20757.1 calcium-dependent protease [Yersinia enterocolitica]

Length=835

Score = 159 bits (401), Expect = 8e-40, Method: Composition-based stats.

Identities = 52/269 (19%), Positives = 102/269 (38%), Gaps = 33/269 (12%)

Query 4 LTERDLS----VLGSYARDGNRELYWNYLSQLPGADGYGTLALGVVRNDSLPGRVANTYA 59

L DL+ +L S+ N +++L+ D Y LA GV R DS+ G +A Y

Sbjct 13 LNAADLACARQLLESHQESKNPGPMYDFLASK--GDRYAVLANGVARGDSIAGAMAIHYL 70

Query 60 QDYAKSQQEEGSRFPNAQLTERQWESFGQTLLERDLELRQQWMNERR-PDLALNLPGKDV 118

+ A+S + +TE + + + L ++Q ++E + ++ +

Sbjct 71 ESVAESH--------DRPITETRLNNIRYDMARGYLAMQQSRLDENPVGIIYGDINHEQA 122

Query 119 MLAHDRAFERHELDPNCWTPRVLLQAAEQKSGPAKLEQIWTNMLNNDYAGGPRVGNTSVD 178

H+RAF H L WT +L+A ++KS PA EQ+ G +

Sbjct 123 TWFHNRAFRDHRLPSKAWTLDAVLRAIDEKSRPAYWEQV-----------LNAAGKPKEE 171

Query 179 AISQMGWTKGGQYLTRLSVLEATQ-ALEGRSAVDP----NVIGGNSYYAMYFEADRKWAS 233

+ + ++ Q + E VD + +S ++ + S

Sbjct 172 LLLSADTYQKMALSAKVGPDGIQQSSREWFDRVDSLAGYWALVKSSSSQLFSSDEAAALS 231

Query 234 ISAGGGHMSLREITDPSRIAELNDAREVR 262

+ ++++ P + + D +V+

Sbjct 232 AAECNFNINISAT--PQTVERVADEDQVQ 258

>WP_049679234.1 serine protease [Yersinia mollaretii]

CNI63132.1 putative serine protease [Yersinia mollaretii]

Length=1817

Score = 159 bits (402), Expect = 8e-40, Method: Composition-based stats.

Identities = 53/265 (20%), Positives = 99/265 (37%), Gaps = 28/265 (11%)

Query 4 LTERDLS----VLGSYARDGNRELYWNYLSQLPGADGYGTLALGVVRNDSLPGRVANTYA 59

L DL+ +L S+ + +++L + D Y TLA GV R +S+ G +A Y

Sbjct 13 LNAADLACARQLLESHQESKDPSPMYDFL--IAKGDRYATLANGVARGNSIAGGMAIHYL 70

Query 60 QDYAKSQQEEGSRFPNAQLTERQWESFGQTLLERDLELRQQWMNERRPDLALNLPGKDVM 119

+ A S N + E Q + L+++Q +++ + ++ K

Sbjct 71 ESVAASH--------NQPINEGQLNDIRFAMAHGYLDMQQDRLDKSGDTIYGDINHKQAA 122

Query 120 LAHDRAFERHELDPNCWTPRVLLQAAEQKSGPAKLEQIWTNMLNNDYAGGPRVGNTSVDA 179

L H+R F L P WT + +A ++KS P EQ+ N SVD

Sbjct 123 LFHNRVFREFVLPPEAWTLDPVFKAIDEKSRPVYWEQV-----LNAAGKPVEELKLSVDT 177

Query 180 ISQMGWTKGGQYLTRLSVLEATQALEGRSAVDP----NVIGGNSYYAMYFEADRKWASIS 235

+M L + +++Q E + VD +G ++ ++ D

Sbjct 178 YQKMAL---SSKLAPDEIQKSSQ--EWFARVDSPSGYWALGKSATSQLFTPPDETPIDTP 232

Query 236 AGGGHMSLREITDPSRIAELNDARE 260

+++ ++ D +

Sbjct 233 QCNIDINITPTPQATQRITDEDQAQ 257

>WP_020283001.1 serine protease [Yersinia enterocolitica]

CCV29237.1 putative serine protease [Yersinia enterocolitica (type O:9)

str. YE212/02]

CCV37716.1 putative serine protease [Yersinia enterocolitica (type O:9)

str. YE56/03]

CNC36618.1 putative serine protease [Yersinia enterocolitica]

CNC42192.1 putative serine protease [Yersinia enterocolitica]

CNC47571.1 putative serine protease [Yersinia enterocolitica]

CNC54956.1 putative serine protease [Yersinia enterocolitica]

CNC71085.1 putative serine protease [Yersinia enterocolitica]

CNC72435.1 putative serine protease [Yersinia enterocolitica]

CND99157.1 putative serine protease [Yersinia enterocolitica]

CNE80325.1 putative serine protease [Yersinia enterocolitica]

CNJ92934.1 putative serine protease [Yersinia enterocolitica]

CQD40937.1 putative serine protease [Yersinia enterocolitica]

CQD65529.1 putative serine protease [Yersinia enterocolitica]

CQH08675.1 putative serine protease [Yersinia enterocolitica]

CQH96202.1 putative serine protease [Yersinia enterocolitica]

CQI08414.1 putative serine protease [Yersinia enterocolitica]

CQJ28759.1 putative serine protease [Yersinia enterocolitica]

CQJ40806.1 putative serine protease [Yersinia enterocolitica]

CQQ91097.1 putative serine protease [Yersinia enterocolitica]

CQQ97907.1 putative serine protease [Yersinia enterocolitica]

CQR02864.1 putative serine protease [Yersinia enterocolitica]

CRX46623.1 putative serine protease [Yersinia enterocolitica]

CRX89836.1 putative serine protease [Yersinia enterocolitica]

Length=1806

Score = 159 bits (401), Expect = 1e-39, Method: Composition-based stats.

Identities = 52/269 (19%), Positives = 102/269 (38%), Gaps = 33/269 (12%)

Query 4 LTERDLS----VLGSYARDGNRELYWNYLSQLPGADGYGTLALGVVRNDSLPGRVANTYA 59

L DL+ +L S+ N +++L+ D Y LA GV R DS+ G +A Y

Sbjct 13 LNAADLACARQLLESHQESKNPGPMYDFLASK--GDRYAVLANGVARGDSVAGAMAIHYL 70

Query 60 QDYAKSQQEEGSRFPNAQLTERQWESFGQTLLERDLELRQQWMNERR-PDLALNLPGKDV 118

+ A+S + +TE + + + L ++Q ++E + ++ +

Sbjct 71 ESVAESH--------DRPITETRLNNIRYDMARGYLAMQQSRLDENPVGIIYGDINHEQA 122

Query 119 MLAHDRAFERHELDPNCWTPRVLLQAAEQKSGPAKLEQIWTNMLNNDYAGGPRVGNTSVD 178

H+RAF H L WT +L+A ++KS PA EQ+ G +

Sbjct 123 TWFHNRAFRDHRLPSKAWTLDAVLRAIDEKSRPAYWEQV-----------LNAAGKPKEE 171

Query 179 AISQMGWTKGGQYLTRLSVLEATQ-ALEGRSAVDP----NVIGGNSYYAMYFEADRKWAS 233

+ + ++ Q + E VD + +S ++ + S

Sbjct 172 LLLSADTYQKMALSAKVGPDGIQQSSREWFDRVDSLAGYWALVKSSSSQLFSSDEAAALS 231

Query 234 ISAGGGHMSLREITDPSRIAELNDAREVR 262

+ ++++ P + + D +V+

Sbjct 232 AAECNFNINISAT--PQTVERVADEDQVQ 258

>CNE41702.1 calcium-dependent protease [Yersinia enterocolitica]

Length=1280

Score = 158 bits (400), Expect = 1e-39, Method: Composition-based stats.

Identities = 52/269 (19%), Positives = 102/269 (38%), Gaps = 33/269 (12%)

Query 4 LTERDLS----VLGSYARDGNRELYWNYLSQLPGADGYGTLALGVVRNDSLPGRVANTYA 59

L DL+ +L S+ N +++L+ D Y LA GV R DS+ G +A Y

Sbjct 13 LNAADLACARQLLESHQESKNPGPMYDFLASK--GDRYAVLANGVARGDSIAGAMAIHYL 70

Query 60 QDYAKSQQEEGSRFPNAQLTERQWESFGQTLLERDLELRQQWMNERR-PDLALNLPGKDV 118

+ A+S + +TE + + + L ++Q ++E + ++ +

Sbjct 71 ESVAESH--------DRPITETRLNNIRYDMARGYLAMQQSRLDENPVGIIYGDINHEQA 122

Query 119 MLAHDRAFERHELDPNCWTPRVLLQAAEQKSGPAKLEQIWTNMLNNDYAGGPRVGNTSVD 178

H+RAF H L WT +L+A ++KS PA EQ+ G +

Sbjct 123 TWFHNRAFRDHRLPSKAWTLDAVLRAIDEKSRPAYWEQV-----------LNAAGKPKEE 171

Query 179 AISQMGWTKGGQYLTRLSVLEATQ-ALEGRSAVDP----NVIGGNSYYAMYFEADRKWAS 233

+ + ++ Q + E VD + +S ++ + S

Sbjct 172 LLLSADTYQKMALSAKVGPDGIQQSSREWFDRVDSLAGYWALVKSSSSQLFSSDEAAALS 231

Query 234 ISAGGGHMSLREITDPSRIAELNDAREVR 262

+ ++++ P + + D +V+

Sbjct 232 AAECNFNINISAT--PQTVERVADEDQVQ 258

>WP_038891128.1 serine protease [Yersinia enterocolitica]

Length=1280

Score = 158 bits (400), Expect = 1e-39, Method: Composition-based stats.

Identities = 52/269 (19%), Positives = 102/269 (38%), Gaps = 33/269 (12%)

Query 4 LTERDLS----VLGSYARDGNRELYWNYLSQLPGADGYGTLALGVVRNDSLPGRVANTYA 59

L DL+ +L S+ N +++L+ D Y LA GV R DS+ G +A Y

Sbjct 13 LNAADLACARQLLESHQESKNPGPMYDFLASK--GDRYAVLANGVARGDSIAGAMAIHYL 70

Query 60 QDYAKSQQEEGSRFPNAQLTERQWESFGQTLLERDLELRQQWMNERR-PDLALNLPGKDV 118

+ A+S + +TE + + + L ++Q ++E + ++ +

Sbjct 71 ESVAESH--------DRPITETRLNNIRYDMARGYLAMQQSRLDENPVGIIYGDINHEQA 122

Query 119 MLAHDRAFERHELDPNCWTPRVLLQAAEQKSGPAKLEQIWTNMLNNDYAGGPRVGNTSVD 178

H+RAF H L WT +L+A ++KS PA EQ+ G +

Sbjct 123 TWFHNRAFRDHRLPSKAWTLDAVLRAIDEKSRPAYWEQV-----------LNAAGKPKEE 171

Query 179 AISQMGWTKGGQYLTRLSVLEATQ-ALEGRSAVDP----NVIGGNSYYAMYFEADRKWAS 233

+ + ++ Q + E VD + +S ++ + S

Sbjct 172 LLLSADTYQKMALSAKVGPDGIQQSSREWFDRVDSLAGYWALVKSSSSQLFSSDEAAALS 231

Query 234 ISAGGGHMSLREITDPSRIAELNDAREVR 262

+ ++++ P + + D +V+

Sbjct 232 AAECNFNINISAT--PQTVERVADEDQVQ 258

>WP_014608845.1 serine protease [Yersinia enterocolitica]

EOR69543.1 calcium-dependent protease [Yersinia enterocolitica subsp. palearctica

YE-149]

EOR80654.1 calcium-dependent protease [Yersinia enterocolitica subsp. palearctica

YE-P1]

EOR81247.1 calcium-dependent protease [Yersinia enterocolitica subsp. palearctica

YE-150]

EOR83778.1 calcium-dependent protease [Yersinia enterocolitica subsp. palearctica

YE-P4]

OAM69911.1 serine protease [Yersinia enterocolitica subsp. palearctica]

CBY27924.1 calcium-dependent protease precursor [Yersinia enterocolitica

subsp. palearctica Y11]

CFQ14913.1 calcium-dependent protease [Yersinia enterocolitica]

CFW64912.1 calcium-dependent protease [Yersinia enterocolitica]

CNB66918.1 calcium-dependent protease [Yersinia enterocolitica]

CND07550.1 calcium-dependent protease [Yersinia enterocolitica]

CNF22941.1 calcium-dependent protease [Yersinia enterocolitica]

CNH50196.1 calcium-dependent protease [Yersinia enterocolitica]

CNH67045.1 calcium-dependent protease [Yersinia enterocolitica]

CNH72879.1 calcium-dependent protease [Yersinia enterocolitica]

CNH75829.1 calcium-dependent protease [Yersinia enterocolitica]

CNI20429.1 calcium-dependent protease [Yersinia enterocolitica]

CNJ59260.1 calcium-dependent protease [Yersinia enterocolitica]

CQD48922.1 calcium-dependent protease [Yersinia enterocolitica]

CQJ20735.1 calcium-dependent protease [Yersinia enterocolitica]

CQJ31860.1 calcium-dependent protease [Yersinia enterocolitica]

CRY22833.1 calcium-dependent protease [Yersinia enterocolitica]

CRY23720.1 calcium-dependent protease [Yersinia enterocolitica]

VEA99978.1 calcium-dependent protease [Yersinia enterocolitica subsp. enterocolitica]

VEF83656.1 calcium-dependent protease [Yersinia enterocolitica subsp. palearctica]

Length=1280

Score = 158 bits (400), Expect = 1e-39, Method: Composition-based stats.

Identities = 52/269 (19%), Positives = 102/269 (38%), Gaps = 33/269 (12%)

Query 4 LTERDLS----VLGSYARDGNRELYWNYLSQLPGADGYGTLALGVVRNDSLPGRVANTYA 59

L DL+ +L S+ N +++L+ D Y LA GV R DS+ G +A Y

Sbjct 13 LNAADLACARQLLESHQESKNPGPMYDFLASK--GDRYAVLANGVARGDSIAGAMAIHYL 70

Query 60 QDYAKSQQEEGSRFPNAQLTERQWESFGQTLLERDLELRQQWMNERR-PDLALNLPGKDV 118

+ A+S + +TE + + + L ++Q ++E + ++ +

Sbjct 71 ESVAESH--------DRPITETRLNNIRYDMARGYLAMQQSRLDENPVGIIYGDINHEQA 122

Query 119 MLAHDRAFERHELDPNCWTPRVLLQAAEQKSGPAKLEQIWTNMLNNDYAGGPRVGNTSVD 178

H+RAF H L WT +L+A ++KS PA EQ+ G +

Sbjct 123 TWFHNRAFRDHRLPSKAWTLDAVLRAIDEKSRPAYWEQV-----------LNAAGKPKEE 171

Query 179 AISQMGWTKGGQYLTRLSVLEATQ-ALEGRSAVDP----NVIGGNSYYAMYFEADRKWAS 233

+ + ++ Q + E VD + +S ++ + S

Sbjct 172 LLLSADTYQKMALSAKVGPDGIQQSSREWFDRVDSLAGYWALVKSSSSQLFSSDEAAALS 231

Query 234 ISAGGGHMSLREITDPSRIAELNDAREVR 262

+ ++++ P + + D +V+

Sbjct 232 AAECNFNINISAT--PQTVERVADEDQVQ 258

>WP_050326492.1 serine protease [Yersinia enterocolitica]

CRX73887.1 calcium-dependent protease [Yersinia enterocolitica]

Length=1280

Score = 158 bits (399), Expect = 2e-39, Method: Composition-based stats.

Identities = 52/269 (19%), Positives = 102/269 (38%), Gaps = 33/269 (12%)

Query 4 LTERDLS----VLGSYARDGNRELYWNYLSQLPGADGYGTLALGVVRNDSLPGRVANTYA 59

L DL+ +L S+ N +++L+ D Y LA GV R DS+ G +A Y

Sbjct 13 LNAADLACARQLLESHQESKNPGPMYDFLASK--GDRYAVLANGVARGDSIAGAMAIHYL 70

Query 60 QDYAKSQQEEGSRFPNAQLTERQWESFGQTLLERDLELRQQWMNERR-PDLALNLPGKDV 118

+ A+S + +TE + + + L ++Q ++E + ++ +

Sbjct 71 ESVAESH--------DRPITETRLNNIRYDMARGYLAMQQSRLDENPVGIIYGDINHEQA 122

Query 119 MLAHDRAFERHELDPNCWTPRVLLQAAEQKSGPAKLEQIWTNMLNNDYAGGPRVGNTSVD 178

H+RAF H L WT +L+A ++KS PA EQ+ G +

Sbjct 123 TWFHNRAFRDHRLPSKAWTLDAVLRAIDEKSRPAYWEQV-----------LNAAGKPKEE 171

Query 179 AISQMGWTKGGQYLTRLSVLEATQ-ALEGRSAVDP----NVIGGNSYYAMYFEADRKWAS 233

+ + ++ Q + E VD + +S ++ + S

Sbjct 172 LLLSADTYQKMALSAKVGPDGIQQSSREWFDRVDSLAGYWALVKSSSSQLFSSDEAAALS 231

Query 234 ISAGGGHMSLREITDPSRIAELNDAREVR 262

+ ++++ P + + D +V+

Sbjct 232 AAECNFNINISAT--PQTVERVADEDQVQ 258

>WP_112067540.1 hypothetical protein [Herbaspirillum rubrisubalbicans]

Length=2397

Score = 158 bits (400), Expect = 2e-39, Method: Composition-based stats.

Identities = 53/291 (18%), Positives = 89/291 (31%), Gaps = 21/291 (7%)

Query 4 LTERDLSVLGSYARDGN--------RELYWNYLSQLPGADGYGTLALGVVRNDSLPGRVA 55

L E D++ +GN + L Y ALGV + +++ G A

Sbjct 7 LNEEDIAAYARKILNGNTNHERISAVIDVYRQLYDQ--GYSYAGWALGVAKGNTVTGTAA 64

Query 56 NTYAQDYAKSQQEEGSRFPNAQLTERQWESFGQTLLERDLELRQQWMNERRPDLALNLPG 115

Y A G+ LT + + + L + + L +L

Sbjct 65 LDYLMGTALM--GLGNDRVCRNLTPTEIDKIRVDMAIETLAQMRMQARDNGGTLRSDLNF 122

Query 116 KDVMLAHDRAFERHELDPNCWTPRVLLQAAEQKSGPAKLEQIWTNMLNNDYAGGPRVGNT 175

V AH+ AF+ L + WT V + + G E++WT + + G

Sbjct 123 MQVRAAHENAFKSSGLSLDNWTLHVPMSLYRKTHGDEATERLWTRIRDTGGD-----GLD 177

Query 176 SVDAISQMGWTKGGQYLTRLSVLEATQALEGRSAVDPNVIGGNSYYAMYFEADRKWASI- 234

+ +Q+ G +AL V P + + + + KW +

Sbjct 178 GLMVSTQLTAEMGRLAFESRDPAIRAEALAWMEKV-PGTLNAAALWRS-TKLIGKWLGVS 235

Query 235 SAGGGHMSLREITDPSRIAELNDAREVRLERLEKRTQFHPDDPYRTITRSP 285

G S I D + +D R + Q D SP

Sbjct 236 VDSIGDFSQPAIADAPQ-QLKSDQRLLEGIVQRLAAQAVTHDNLTPGLDSP 285

>WP_112076531.1 hypothetical protein [Herbaspirillum rubrisubalbicans]

Length=2397

Score = 158 bits (400), Expect = 2e-39, Method: Composition-based stats.

Identities = 53/291 (18%), Positives = 89/291 (31%), Gaps = 21/291 (7%)

Query 4 LTERDLSVLGSYARDGN--------RELYWNYLSQLPGADGYGTLALGVVRNDSLPGRVA 55

L E D++ +GN + L Y ALGV + +++ G A

Sbjct 7 LNEEDIAAYARKILNGNTNHERISAVIDVYRQLYDQ--GYSYAGWALGVAKGNTVTGTAA 64

Query 56 NTYAQDYAKSQQEEGSRFPNAQLTERQWESFGQTLLERDLELRQQWMNERRPDLALNLPG 115

Y A G+ LT + + + L + + L +L

Sbjct 65 LDYLMGTALM--GLGNDRVCRNLTPTEIDKIRVDMAIETLAQMRMQARDNGGTLRSDLNF 122

Query 116 KDVMLAHDRAFERHELDPNCWTPRVLLQAAEQKSGPAKLEQIWTNMLNNDYAGGPRVGNT 175

V AH+ AF+ L + WT V + + G E++WT + + G

Sbjct 123 MQVRAAHENAFKSSGLSLDNWTLHVPMSLYRKTHGDEATERLWTRIRDTGGD-----GLD 177

Query 176 SVDAISQMGWTKGGQYLTRLSVLEATQALEGRSAVDPNVIGGNSYYAMYFEADRKWASI- 234

+ +Q+ G +AL V P + + + + KW +

Sbjct 178 GLMVSTQLTAEMGRLAFESRDPAIRAEALAWMEKV-PGTLNAAALWRS-TKLIGKWLGVS 235

Query 235 SAGGGHMSLREITDPSRIAELNDAREVRLERLEKRTQFHPDDPYRTITRSP 285

G S I D + +D R + Q D SP

Sbjct 236 VDSIGDFSQPAIADAPQ-QLKSDQRLLEGIVQRLAAQAVTHDNLTPGLDSP 285

>CUV21285.1 conserved protein of unknown function, partial [Ralstonia solanacearum]

Length=1051

Score = 156 bits (395), Expect = 7e-39, Method: Composition-based stats.

Identities = 31/194 (16%), Positives = 61/194 (31%), Gaps = 13/194 (7%)

Query 4 LTERDLSVLGSYARDGNRELYWNYLSQLPGADGYGTLALGVVRNDSLPGRVANTYAQDYA 63

+ + D S L + G +++YL Y GV DS+ G A Y A

Sbjct 5 IKKDDFSRLENMLAHGQVREFYSYL--KEHGYAYAGWGRGVAMEDSISGISAIDYLTGSA 62

Query 64 KSQQEEGSRFPNAQLTERQWESFGQTLLERDLELRQQWMNE-----RRPDLALNLPGKDV 118

+T + + Q + + L + + + ++ ++V

Sbjct 63 LM---GMGGEACWNITPDKSDKIKQEMADAYLNTLEAIAEKNLKNTGEYEANRDINAEEV 119

Query 119 MLAHDRAFERHELDPNCWTPRVLLQAAEQKSGPAKLEQIWTNMLNNDYAGGPRVG---NT 175

H + F+ + L WT + + +Q G LE W ++ + G T

Sbjct 120 WDFHKKVFKDNGLGIENWTLDSVFKTIQQTQGDDALEAYWESLRDTQGEGMMATLLNIRT 179

Query 176 SVDAISQMGWTKGG 189

+ +

Sbjct 180 MYNMHESIDSADPA 193

>WP_082657015.1 hypothetical protein [Pseudomonas citronellolis]

Length=2488

Score = 156 bits (394), Expect = 1e-38, Method: Composition-based stats.

Identities = 35/201 (17%), Positives = 65/201 (32%), Gaps = 19/201 (9%)

Query 4 LTERDLSVLGSYARDGNRELYWNYLSQLPGADGYGTLALGVVRNDSLPGRVANTYAQDYA 63

L + DL + + ++Y Y TLA GV R DS+ G A Y ++ A

Sbjct 5 LAQSDLDHARTLLNERGPSAMYDYFESK--GYRYATLANGVARGDSIAGEAAIGYMKETA 62

Query 64 KSQQEEGSRFPNAQLTERQWESFGQTLLERDLELRQQWMNERRPDLALNLPGKDVMLAHD 123

+ ++ +S + + L Q ++ + ++ ++ H

Sbjct 63 ED--------EGRPMSSSDVDSVRRDMANGYLNTLQGQADKNNGVVTRDITHQEAWEFHS 114

Query 124 RAFERHELDPNCWTPRVLLQAAEQKSGPAKLEQIWTNMLNNDYAGGPRVGNTSVDAISQM 183

+ F H L N WT + E+ W ++LN+ + ++ A

Sbjct 115 KVFNEHGLSVNAWTLNIPFSLM----DVEDRERYWQDVLNSAGDPQAEILLSARTA---- 166

Query 184 GWTKGGQYLTRLSVLEATQAL 204

YL L

Sbjct 167 -QFMAYAYLFGDGEQITAARL 186

>WP_050165943.1 serine protease [Yersinia enterocolitica]

CNF58873.1 putative serine protease [Yersinia enterocolitica]

Length=1804

Score = 156 bits (393), Expect = 1e-38, Method: Composition-based stats.

Identities = 50/268 (19%), Positives = 93/268 (35%), Gaps = 31/268 (12%)

Query 4 LTERDLS----VLGSYARDGNRELYWNYLSQLPGADGYGTLALGVVRNDSLPGRVANTYA 59

L DL+ +L ++ + +++L+ D Y LA GVVR DS+ G +A Y

Sbjct 13 LNAADLACARQLLEAHNESKDPGPMYDFLASK--GDRYAILANGVVRGDSIAGAMAIHYL 70

Query 60 QDYAKSQQEEGSRFPNAQLTERQWESFGQTLLERDLELRQQWMNERRPDL-ALNLPGKDV 118

+ A S N +T+ + L +Q +++ + ++ +

Sbjct 71 ESVAASH--------NQPITDIGLNDIRYDMAHGYLNTQQTRLDDSPTGIIYGDIGHEQA 122

Query 119 MLAHDRAFERHELDPNCWTPRVLLQAAEQKSGPAKLEQIWTNMLNNDYAGGPRVGNTSVD 178

H F H L WT + +A ++KS P W +LN + SVD

Sbjct 123 AQFHSSVFGDHGLPAEAWTLDQVFKAIDEKSRP----VYWDLVLNAAGKPKEELW-LSVD 177

Query 179 AISQMGWTKGGQYLTRLSVLEATQALEGRSAVDP----NVIGGNSYYAMYFEADRKW-AS 233

+M + + E VD + +S ++ + S

Sbjct 178 TYQKMALSAKVA-----PDGIRESSREWFDRVDSLSGYWALAKSSTSQLFSSDEEVAPVS 232

Query 234 ISAGGGHMSLREITDP-SRIAELNDARE 260

+++ R+A+ + AR

Sbjct 233 TEMCNIDINISPTPHAVQRVADEDQARR 260

>WP_046694811.1 serine protease [Yersinia enterocolitica]

AKF37574.1 serine protease [Yersinia enterocolitica]

ALG45837.1 serine protease [Yersinia enterocolitica]

CNF01619.1 putative serine protease [Yersinia enterocolitica]

CQH28223.1 putative serine protease [Yersinia enterocolitica]

CQJ08552.1 putative serine protease [Yersinia enterocolitica]

CQJ27626.1 putative serine protease [Yersinia enterocolitica]

CQQ32693.1 putative serine protease [Yersinia enterocolitica]

Length=1804

Score = 156 bits (393), Expect = 1e-38, Method: Composition-based stats.

Identities = 55/268 (21%), Positives = 95/268 (35%), Gaps = 31/268 (12%)

Query 4 LTERDL----SVLGSYARDGNRELYWNYLSQLPGADGYGTLALGVVRNDSLPGRVANTYA 59

L DL +L ++ + +++L+ D Y LA GV + DS+ G +A Y

Sbjct 13 LNAADLACARELLAAHQESKDPSPMYDFLASK--GDRYARLANGVAKGDSIAGAMAIHYL 70

Query 60 QDYAKSQQEEGSRFPNAQLTERQWESFGQTLLERDLELRQQWMNERRPDL-ALNLPGKDV 118

+ A S N +T Q + + L +Q+ ++ + ++ K

Sbjct 71 ESVAASH--------NQPMTASQLNNIRYDMAHGYLNTQQKRLDASPTGIIYGDIDHKQA 122

Query 119 MLAHDRAFERHELDPNCWTPRVLLQAAEQKSGPAKLEQIWTNMLNNDYAGGPRVGNTSVD 178

L H+ F +HEL WT + +A E K PA W +L N SVD

Sbjct 123 ALFHNDVFGKHELPSKAWTLDPVFKAIEPKIRPA----YWQWVL-NAADNPKAELMLSVD 177

Query 179 AISQMGWTKGGQYLTRLSVLEATQALEGRSAVDP----NVIGGNSYYAMYF-EADRKWAS 233

+M + + E VD + +S ++ E + S

Sbjct 178 TYQKMALSVKVA-----PDDIRASSREWFDRVDSLSGYWALAKSSTRQLFSTEEEAALFS 232

Query 234 ISAGGGHMSLREITDP-SRIAELNDARE 260

+++ RIA+ + AR

Sbjct 233 SEICNFDINISPTPQAVQRIADEDQARH 260

>WP_050162668.1 serine protease [Yersinia enterocolitica]

CNL17734.1 putative serine protease [Yersinia enterocolitica]

Length=1804

Score = 155 bits (391), Expect = 2e-38, Method: Composition-based stats.

Identities = 54/268 (20%), Positives = 92/268 (34%), Gaps = 31/268 (12%)

Query 4 LTERDLSVLGSYAR----DGNRELYWNYLSQLPGADGYGTLALGVVRNDSLPGRVANTYA 59

L DL+ + +++L+ D Y LA GV + DS+ G +A Y

Sbjct 13 LNAADLACARQLLEANKESKDPGPMYDFLASK--GDRYARLANGVAKGDSIAGAMAIHYL 70

Query 60 QDYAKSQQEEGSRFPNAQLTERQWESFGQTLLERDLELRQQWMNERRPDL-ALNLPGKDV 118

+ A S N +T Q + + L +Q+ ++ + ++ K

Sbjct 71 ESVAASH--------NQPMTASQLNNIRYDMAHGYLNTQQKRLDASPTGIIYGDIDHKQA 122

Query 119 MLAHDRAFERHELDPNCWTPRVLLQAAEQKSGPAKLEQIWTNMLNNDYAGGPRVGNTSVD 178

L H+ F +HEL WT + +A E K PA W +L N SVD

Sbjct 123 ALFHNDVFGKHELPSKAWTLDPVFKAIEPKIRPA----YWQWVL-NAADNPKAELMLSVD 177

Query 179 AISQMGWTKGGQYLTRLSVLEATQALEGRSAVDP----NVIGGNSYYAMYF-EADRKWAS 233

+M + + E VD + +S ++ E + S

Sbjct 178 TYQKMALSVKVA-----PDDIRASSREWFDRVDSLSGYWALAKSSTRQLFSTEEEAALFS 232

Query 234 ISAGGGHMSLREITDP-SRIAELNDARE 260

+++ RIA+ + AR

Sbjct 233 SEICNFDINISPTPQAVQRIADEDQARH 260

>WP_032912768.1 serine protease [Yersinia enterocolitica]

KGA71778.1 proconvertase P-domain protein [Yersinia enterocolitica]

PNM14056.1 serine protease [Yersinia enterocolitica]

Length=1803

Score = 154 bits (390), Expect = 3e-38, Method: Composition-based stats.

Identities = 50/267 (19%), Positives = 91/267 (34%), Gaps = 30/267 (11%)

Query 4 LTERDLSVLGSYAR----DGNRELYWNYLSQLPGADGYGTLALGVVRNDSLPGRVANTYA 59

L DL+ + +++L+ D Y LA GVVR DS+ G +A Y

Sbjct 13 LNAADLACARQLLEANKESKDPGPMYDFLASK--GDRYAILANGVVRGDSIAGAMAIHYL 70

Query 60 QDYAKSQQEEGSRFPNAQLTERQWESFGQTLLERDLELRQQWMNERRPDL-ALNLPGKDV 118

+ A S N +TE + + + L +Q+ +++ + ++ K

Sbjct 71 ESVAASH--------NQPITEMRLKHIRYDMAHGYLNTQQKRLDDSPTGIIYGDIDHKQA 122

Query 119 MLAHDRAFERHELDPNCWTPRVLLQAAEQKSGPAKLEQIWTNMLNNDYAGGPRVGNTSVD 178

H+ +RH L WT + +A +++S P W +L N S D

Sbjct 123 GQFHNEELKRHGLPSKAWTLDPVFRAIDERSQPI----YWQWVL-NAAGKPKEELRLSAD 177

Query 179 AISQMGWTKGGQYLTRLSVLEATQALEGRSAVDP----NVIGGNSYYAMYFEADRKWASI 234

+M + + E VD + +S ++

Sbjct 178 TYQKMALSAKVA-----PDGIRQSSREWFDRVDSLLGYWALAKSSTSQLFAFDQTTTVLP 232

Query 235 SAGGGHMSLREITDP-SRIAELNDARE 260

+++ P RIA+ + AR

Sbjct 233 QQCNFDINISPTPQPVQRIADEDQARR 259

>WP_005171591.1 serine protease [Yersinia enterocolitica]

AJI85118.1 proconvertase P-domain protein [Yersinia enterocolitica]

EKA27159.1 putative serine protease [Yersinia enterocolitica subsp. enterocolitica

WA-314]

KGA77157.1 proconvertase P-domain protein [Yersinia enterocolitica]

RLY99678.1 serine protease [Yersinia enterocolitica]

CFQ08933.1 putative serine protease [Yersinia enterocolitica]

CNE83342.1 putative serine protease [Yersinia enterocolitica]

CNF61858.1 putative serine protease [Yersinia enterocolitica]

CNI98928.1 putative serine protease [Yersinia enterocolitica]

CNJ70428.1 putative serine protease [Yersinia enterocolitica]

VFS96487.1 putative hemolysin-type calcium-binding region [Yersinia enterocolitica]

Length=1803

Score = 154 bits (390), Expect = 3e-38, Method: Composition-based stats.

Identities = 50/267 (19%), Positives = 91/267 (34%), Gaps = 30/267 (11%)

Query 4 LTERDLSVLGSYAR----DGNRELYWNYLSQLPGADGYGTLALGVVRNDSLPGRVANTYA 59

L DL+ + +++L+ D Y LA GVVR DS+ G +A Y

Sbjct 13 LNAADLACARQLLEANKESKDPGPMYDFLASK--GDRYAILANGVVRGDSIAGAMAIHYL 70

Query 60 QDYAKSQQEEGSRFPNAQLTERQWESFGQTLLERDLELRQQWMNERRPDL-ALNLPGKDV 118

+ A S N +TE + + + L +Q+ +++ + ++ K

Sbjct 71 ESVAASH--------NQPITEMRLKHIRYDMAHGYLNTQQKRLDDSPTGIIYGDIDHKQA 122

Query 119 MLAHDRAFERHELDPNCWTPRVLLQAAEQKSGPAKLEQIWTNMLNNDYAGGPRVGNTSVD 178

H+ +RH L WT + +A +++S P W +L N S D

Sbjct 123 GQFHNEELKRHGLPSKAWTLDPVFRAIDERSQPI----YWQWVL-NAAGKPKEELRLSAD 177

Query 179 AISQMGWTKGGQYLTRLSVLEATQALEGRSAVDP----NVIGGNSYYAMYFEADRKWASI 234

+M + + E VD + +S ++

Sbjct 178 TYQKMALSAKVA-----PDGIRQSSREWFDRVDSLPGYWALAKSSTSQLFAFDQTTTVLP 232

Query 235 SAGGGHMSLREITDP-SRIAELNDARE 260

+++ P RIA+ + AR

Sbjct 233 QQCNFDINISPTPQPVQRIADEDQARR 259

>WP_102990176.1 serine protease [Yersinia enterocolitica]

PNM19254.1 serine protease [Yersinia enterocolitica]

Length=1803

Score = 154 bits (390), Expect = 3e-38, Method: Composition-based stats.

Identities = 50/267 (19%), Positives = 91/267 (34%), Gaps = 30/267 (11%)

Query 4 LTERDLSVLGSYAR----DGNRELYWNYLSQLPGADGYGTLALGVVRNDSLPGRVANTYA 59

L DL+ + +++L+ D Y LA GVVR DS+ G +A Y

Sbjct 13 LNAADLACARQLLEANKESKDPGPMYDFLASK--GDRYAILANGVVRGDSIAGAMAIHYL 70

Query 60 QDYAKSQQEEGSRFPNAQLTERQWESFGQTLLERDLELRQQWMNERRPDL-ALNLPGKDV 118

+ A S N +TE + + + L +Q+ +++ + ++ K

Sbjct 71 ESVAASH--------NQPITEMRLKHIRYDMAHGYLNTQQKRLDDSPTGIIYGDIDHKQA 122

Query 119 MLAHDRAFERHELDPNCWTPRVLLQAAEQKSGPAKLEQIWTNMLNNDYAGGPRVGNTSVD 178

H+ +RH L WT + +A +++S P W +L N S D

Sbjct 123 GQFHNEELKRHGLPSKAWTLDPVFRAIDERSQPI----YWQWVL-NAAGKPKEELRLSAD 177

Query 179 AISQMGWTKGGQYLTRLSVLEATQALEGRSAVDP----NVIGGNSYYAMYFEADRKWASI 234

+M + + E VD + +S ++

Sbjct 178 TYQKMALSAKVA-----PDGIRQSSREWFDRVDSLPGYWALAKSSTSQLFAFDQTTTVLP 232

Query 235 SAGGGHMSLREITDP-SRIAELNDARE 260

+++ P RIA+ + AR

Sbjct 233 QQCNFDINISPTPQPVQRIADEDQARR 259

>WP_050322994.1 serine protease [Yersinia enterocolitica]

CRY02453.1 putative serine protease [Yersinia enterocolitica]

Length=1803

Score = 154 bits (390), Expect = 3e-38, Method: Composition-based stats.

Identities = 50/267 (19%), Positives = 91/267 (34%), Gaps = 30/267 (11%)

Query 4 LTERDLSVLGSYAR----DGNRELYWNYLSQLPGADGYGTLALGVVRNDSLPGRVANTYA 59

L DL+ + +++L+ D Y LA GVVR DS+ G +A Y

Sbjct 13 LNAADLACARQLLEANKESKDPGPMYDFLASK--GDRYAILANGVVRGDSIAGAMAIHYL 70

Query 60 QDYAKSQQEEGSRFPNAQLTERQWESFGQTLLERDLELRQQWMNERRPDL-ALNLPGKDV 118

+ A S N +TE + + + L +Q+ +++ + ++ K

Sbjct 71 ESVAASH--------NQPITEMRLKHIRYDMAHGYLNTQQKRLDDSPTGIIYGDIDHKQA 122

Query 119 MLAHDRAFERHELDPNCWTPRVLLQAAEQKSGPAKLEQIWTNMLNNDYAGGPRVGNTSVD 178

H+ +RH L WT + +A +++S P W +L N S D

Sbjct 123 GQFHNEELKRHGLPSKAWTLDPVFRAIDERSQPI----YWQWVL-NAAGKPKEELRLSAD 177

Query 179 AISQMGWTKGGQYLTRLSVLEATQALEGRSAVDP----NVIGGNSYYAMYFEADRKWASI 234

+M + + E VD + +S ++

Sbjct 178 TYQKMALSAKVA-----PDGIRQSSREWFDRVDSLPGYWALAKSSTSQLFAFDQTTTVLP 232

Query 235 SAGGGHMSLREITDP-SRIAELNDARE 260

+++ P RIA+ + AR

Sbjct 233 QQCNFDINISPTPQPVQRIADEDQARR 259

>WP_050126251.1 serine protease [Yersinia kristensenii]

CNL43029.1 putative serine protease [Yersinia kristensenii]

Length=1822

Score = 154 bits (388), Expect = 6e-38, Method: Composition-based stats.

Identities = 53/313 (17%), Positives = 103/313 (33%), Gaps = 33/313 (11%)

Query 4 LTERDL----SVLGSYARDGNRELYWNYLSQLPGADGYGTLALGVVRNDSLPGRVANTYA 59

L DL +L S+ N +++L + D Y LA GVV +S+ G +A +Y

Sbjct 13 LNTADLKCAREILESHKESKNPGPMYDFL--ITKGDRYAILANGVVNGNSIAGEIAISYL 70

Query 60 QDYAKSQQEEGSRFPNAQLTERQWESFGQTLLERDLELRQQWMNERRPDLALNLPGKDVM 119

+ A N +TE Q + + L++ Q ++ R + ++ K

Sbjct 71 KSVAGGH--------NQPITEVQLINIRYDMALGYLDMLQSRLDSRTGTIYGDINHKQAE 122

Query 120 LAHDRAFERHELDPNCWTPRVLLQAAEQKSGPAKLEQIWTNMLNNDYAGGPRVGNTSVDA 179

H ++ L P WT +L+ + E W + LN + +

Sbjct 123 KFHYIVLNKYGLPPEAWTLNPVLKVMPENF----REGYWQDTLNAAGKPAKEIWLSYYTT 178

Query 180 ISQMGWTKGGQYLTRLSVLEATQALEGRSAV-DPNVIGGNSYY---AMYFEADRKWASIS 235

M L+ ++ + V D + +G + ++ + S

Sbjct 179 -KYM-----FDKLSTSEAVDQPEIRIWLKTVSDIDNLGAVASALGNQLFTSDEAPPVSTQ 232

Query 236 AGGGHMSLREITDPSRIAELNDAREVRLERLEKRTQFHPDDPYRTITRSPLTAAVDDVAD 295

+S+ P + D + R + + + P +++ + T D A

Sbjct 233 QCNIDISITP--SPLATQRITDEDQ---ARRDVANGYLVNKPTHSLSFTDGTLDKTDFAS 287

Query 296 PSQAPTRLADIGP 308

I P

Sbjct 288 VQMGSMASGGIRP 300

>WP_048619760.1 serine protease [Yersinia aleksiciae]

AKP34862.1 serine protease [Yersinia aleksiciae]

CFQ51648.1 putative serine protease [Yersinia aleksiciae]

Length=1822

Score = 154 bits (388), Expect = 6e-38, Method: Composition-based stats.

Identities = 53/313 (17%), Positives = 103/313 (33%), Gaps = 33/313 (11%)

Query 4 LTERDL----SVLGSYARDGNRELYWNYLSQLPGADGYGTLALGVVRNDSLPGRVANTYA 59

L DL +L S+ N +++L + D Y LA GVV +S+ G +A +Y

Sbjct 13 LNTADLKCAREILESHKESKNPGPMYDFL--ITKGDRYAILANGVVNGNSIAGEIAISYL 70

Query 60 QDYAKSQQEEGSRFPNAQLTERQWESFGQTLLERDLELRQQWMNERRPDLALNLPGKDVM 119

+ A N +TE Q + + L++ Q ++ R + ++ K

Sbjct 71 KSVAGGH--------NQPITEVQLINIRYDMALGYLDMLQSRLDSRTGTIYGDINHKQAE 122

Query 120 LAHDRAFERHELDPNCWTPRVLLQAAEQKSGPAKLEQIWTNMLNNDYAGGPRVGNTSVDA 179

H ++ L P WT +L+ + E W + LN + +

Sbjct 123 KFHYIVLNKYGLPPEAWTLNPVLKVMPENF----REGYWQDTLNAAGKPAKEIWLSYYTT 178

Query 180 ISQMGWTKGGQYLTRLSVLEATQALEGRSAV-DPNVIGGNSYY---AMYFEADRKWASIS 235

M L+ ++ + V D + +G + ++ + S

Sbjct 179 -KYM-----FDKLSTSEAVDQPEIRIWLKTVSDIDNLGAVASALGNQLFTSDEAPPVSTQ 232

Query 236 AGGGHMSLREITDPSRIAELNDAREVRLERLEKRTQFHPDDPYRTITRSPLTAAVDDVAD 295

+S+ P + D + R + + + P +++ + T D A

Sbjct 233 QCNIDISITP--SPLATQRITDEDQ---ARRDVANGYLVNKPTHSLSFTDGTLDKTDFAS 287

Query 296 PSQAPTRLADIGP 308

I P

Sbjct 288 VQMGSMASGGIRP 300

>VTP80110.1 putative serine protease [Yersinia enterocolitica subsp. enterocolitica]

Length=1123

Score = 153 bits (387), Expect = 6e-38, Method: Composition-based stats.

Identities = 50/267 (19%), Positives = 91/267 (34%), Gaps = 30/267 (11%)

Query 4 LTERDLSVLGSYAR----DGNRELYWNYLSQLPGADGYGTLALGVVRNDSLPGRVANTYA 59

L DL+ + +++L+ D Y LA GVVR DS+ G +A Y

Sbjct 13 LNAADLACARQLLEANKESKDPGPMYDFLASK--GDRYAILANGVVRGDSIAGAMAIHYL 70

Query 60 QDYAKSQQEEGSRFPNAQLTERQWESFGQTLLERDLELRQQWMNERRPDL-ALNLPGKDV 118

+ A S N +TE + + + L +Q+ +++ + ++ K

Sbjct 71 ESVAASH--------NQPITEMRLKHIRYDMAHGYLNTQQKRLDDSPTGIIYGDIDHKQA 122

Query 119 MLAHDRAFERHELDPNCWTPRVLLQAAEQKSGPAKLEQIWTNMLNNDYAGGPRVGNTSVD 178

H+ +RH L WT + +A +++S P W +L N S D

Sbjct 123 GQFHNEELKRHGLPSKAWTLDPVFRAIDERSQPI----YWQWVL-NAAGKPKEELRLSAD 177

Query 179 AISQMGWTKGGQYLTRLSVLEATQALEGRSAVDP----NVIGGNSYYAMYFEADRKWASI 234

+M + + E VD + +S ++

Sbjct 178 TYQKMALSAKVA-----PDGIRQSSREWFDRVDSLLGYWALAKSSTSQLFAFDQTTTVLP 232

Query 235 SAGGGHMSLREITDP-SRIAELNDARE 260

+++ P RIA+ + AR

Sbjct 233 QQCNFDINISPTPQPVQRIADEDQARR 259

>WP_013650162.1 serine protease [Yersinia enterocolitica]

ADZ43226.1 putative serine protease [Yersinia enterocolitica subsp. palearctica

105.5R(r)]

AJJ28060.1 proconvertase P-domain protein [Yersinia enterocolitica]

ALG79326.1 serine protease [Yersinia enterocolitica]

KGA72435.1 proconvertase P-domain protein [Yersinia enterocolitica]

KGA75451.1 proconvertase P-domain protein [Yersinia enterocolitica]

CNG16113.1 putative serine protease [Yersinia enterocolitica]

CNH00638.1 putative serine protease [Yersinia enterocolitica]

CNH36108.1 putative serine protease [Yersinia enterocolitica]

CRX94112.1 putative serine protease [Yersinia enterocolitica]

CRX95454.1 putative serine protease [Yersinia enterocolitica]

Length=1806

Score = 153 bits (387), Expect = 7e-38, Method: Composition-based stats.

Identities = 51/267 (19%), Positives = 99/267 (37%), Gaps = 33/267 (12%)

Query 4 LTERDLS----VLGSYARDGNRELYWNYLSQLPGADGYGTLALGVVRNDSLPGRVANTYA 59

L DL+ +L S+ N +++L+ D Y +A GVVR DS+ G +A Y

Sbjct 13 LNAADLACARQLLKSHQESKNPGPMYDFLASK--GDRYAVMANGVVRGDSVAGAMAIHYL 70

Query 60 QDYAKSQQEEGSRFPNAQLTERQWESFGQTLLERDLELRQQWMNERR-PDLALNLPGKDV 118

+ A+S + +TE + + + L ++Q +NE + ++ K

Sbjct 71 ESVAESH--------DQPITETRLNNIRYDMARGYLAMQQSRLNENPAGIIYGDIDHKQA 122

Query 119 MLAHDRAFERHELDPNCWTPRVLLQAAEQKSGPAKLEQIWTNMLNNDYAGGPRVGNTSVD 178

H+R F H L WT + +A ++ S PA W +LN G +

Sbjct 123 GSFHNRTFRDHRLPSKAWTLDQVFRAIDKDSRPA----YWQQVLN-------AAGKPKEE 171

Query 179 AISQMGWTKGGQYLTRLSVLEATQ-ALEGRSAVDP----NVIGGNSYYAMYFEADRKWAS 233

+ + ++ Q + E VD + +S ++ + S

Sbjct 172 LLLSADTYQKMALSAKVGPDGIQQSSREWFDRVDSLAGYWALVKSSSSQLFSSDETTALS 231

Query 234 ISAGGGHMSLREITDPSRIAELNDARE 260

+ ++++ P + + D +

Sbjct 232 AAECNFNINISAT--PQTVERVADEDQ 256

>WP_112999018.1 serine protease [Yersinia enterocolitica]

SQA39019.1 putative serine protease [Yersinia enterocolitica]

SUP63545.1 putative serine protease [Yersinia enterocolitica]

Length=1806

Score = 153 bits (387), Expect = 7e-38, Method: Composition-based stats.

Identities = 51/267 (19%), Positives = 99/267 (37%), Gaps = 33/267 (12%)

Query 4 LTERDLS----VLGSYARDGNRELYWNYLSQLPGADGYGTLALGVVRNDSLPGRVANTYA 59

L DL+ +L S+ N +++L+ D Y +A GVVR DS+ G +A Y

Sbjct 13 LNAADLACARQLLKSHQESKNPGPMYDFLASK--GDRYAVMANGVVRGDSVAGAMAIHYL 70

Query 60 QDYAKSQQEEGSRFPNAQLTERQWESFGQTLLERDLELRQQWMNERR-PDLALNLPGKDV 118

+ A+S + +TE + + + L ++Q +NE + ++ K

Sbjct 71 ESVAESH--------DQPITETRLNNIRYDMARGYLAMQQSRLNENPAGIIYGDIDHKQA 122

Query 119 MLAHDRAFERHELDPNCWTPRVLLQAAEQKSGPAKLEQIWTNMLNNDYAGGPRVGNTSVD 178

H+R F H L WT + +A ++ S PA W +LN G +

Sbjct 123 GSFHNRTFRDHRLPSKAWTLDQVFRAIDKDSRPA----YWQQVLN-------AAGKPKEE 171

Query 179 AISQMGWTKGGQYLTRLSVLEATQ-ALEGRSAVDP----NVIGGNSYYAMYFEADRKWAS 233

+ + ++ Q + E VD + +S ++ + S

Sbjct 172 LLLSADTYQKMALSAKVGPDGIQQSSREWFDRVDSLAGYWALVKSSSSQLFSSDETTALS 231

Query 234 ISAGGGHMSLREITDPSRIAELNDARE 260

+ ++++ P + + D +

Sbjct 232 AAECNFNINISAT--PQTVERVADEDQ 256

>WP_049603119.1 serine protease [Yersinia aldovae]

CNK38375.1 putative serine protease [Yersinia aldovae]

Length=1807

Score = 153 bits (386), Expect = 1e-37, Method: Composition-based stats.

Identities = 48/269 (18%), Positives = 98/269 (36%), Gaps = 33/269 (12%)

Query 4 LTERDLS----VLGSYARDGNRELYWNYLSQLPGADGYGTLALGVVRNDSLPGRVANTYA 59

L DL+ +L ++ + + +++L+ D Y LA GV R D + G +A Y

Sbjct 13 LNAADLACARQLLETHKENKDPGPMYDFLASK--GDRYAILANGVTRGDFIAGAMAIHYL 70

Query 60 QDYAKSQQEEGSRFPNAQLTERQWESFGQTLLERDLELRQQWMNERR-PDLALNLPGKDV 118

+ A S N +TE + + + L +Q+ +++ + ++ K

Sbjct 71 ESVAASH--------NQPITEMRLKHIRYDMAHGYLNTQQKRLDDSPAGIIYGDINHKQA 122

Query 119 MLAHDRAFERHELDPNCWTPRVLLQAAEQKSGPAKLEQIWTNMLNNDYAGGPRVGNTSVD 178

H+ +RH L WT + +A ++KS PA EQ+ G + +

Sbjct 123 GQFHNEELKRHGLPSKAWTLDPVFRAIDEKSRPAYWEQV-----------LKAAGKPAEE 171

Query 179 AISQMGWTKGGQYLTRLSVL-EATQALEGRSAVDP----NVIGGNSYYAMYFEADR-KWA 232

+ + +++ T + E VD + + ++ +

Sbjct 172 LKLSVDTYQKMALSAKVAPDGIRTSSREWFDRVDSLPGYWALAKSGTSQLFSSDEEVSPV 231

Query 233 SISAGGGHMSLREITDP-SRIAELNDARE 260

S +++ RIA+ + AR

Sbjct 232 STEMCNIDITINPTPQAVQRIADEDQARR 260

>WP_049688688.1 serine protease [Yersinia aldovae]

CNJ83695.1 putative serine protease [Yersinia aldovae]

Length=1807

Score = 153 bits (386), Expect = 1e-37, Method: Composition-based stats.

Identities = 48/269 (18%), Positives = 98/269 (36%), Gaps = 33/269 (12%)

Query 4 LTERDLS----VLGSYARDGNRELYWNYLSQLPGADGYGTLALGVVRNDSLPGRVANTYA 59

L DL+ +L ++ + + +++L+ D Y LA GV R D + G +A Y

Sbjct 13 LNAADLACARQLLETHKENKDPGPMYDFLASK--GDRYAILANGVTRGDFIAGAMAIHYL 70

Query 60 QDYAKSQQEEGSRFPNAQLTERQWESFGQTLLERDLELRQQWMNERR-PDLALNLPGKDV 118

+ A S N +TE + + + L +Q+ +++ + ++ K

Sbjct 71 ESVAASH--------NQPITEMRLKHIRYDMAHGYLNTQQKRLDDSPAGIIYGDINHKQA 122

Query 119 MLAHDRAFERHELDPNCWTPRVLLQAAEQKSGPAKLEQIWTNMLNNDYAGGPRVGNTSVD 178

H+ +RH L WT + +A ++KS PA EQ+ G + +

Sbjct 123 GQFHNEELKRHGLPSKAWTLDPVFRAIDEKSRPAYWEQV-----------LKAAGKPAEE 171

Query 179 AISQMGWTKGGQYLTRLSVL-EATQALEGRSAVDP----NVIGGNSYYAMYFEADR-KWA 232

+ + +++ T + E VD + + ++ +

Sbjct 172 LKLSVDTYQKMALSAKVAPDGIRTSSREWFDRVDSLPGYWALAKSGTSQLFSSDEEVSPV 231

Query 233 SISAGGGHMSLREITDP-SRIAELNDARE 260

S +++ RIA+ + AR

Sbjct 232 STEMCNIDITINPTPQAVQRIADEDQARR 260

>WP_050113010.1 serine protease [Yersinia kristensenii]

CNH26698.1 putative serine protease [Yersinia kristensenii]

Length=1806

Score = 152 bits (384), Expect = 2e-37, Method: Composition-based stats.

Identities = 54/314 (17%), Positives = 102/314 (32%), Gaps = 33/314 (11%)

Query 4 LTERDLSVLGSYAR----DGNRELYWNYLSQLPGADGYGTLALGVVRNDSLPGRVANTYA 59

L DL+ + +++L+ D Y LA GV + DS+ G +A Y

Sbjct 13 LNAADLACARQLLEANKESKDPSPMYDFLA--TKGDRYARLANGVAKGDSIAGAMAIHYL 70

Query 60 QDYAKSQQEEGSRFPNAQLTERQWESFGQTLLERDLELRQQWMNERRPDL-ALNLPGKDV 118

+ S N +T+ + + L+++Q ++ + ++ +

Sbjct 71 ESVIDSH--------NQPITDSGLNNIRYDMAGGYLDMQQSRLDASHTGIIYGDIDHEQA 122

Query 119 MLAHDRAFERHELDPNCWTPRVLLQAAEQKSGPAKLEQIWTNMLNNDYAGGPRVGNTSVD 178

L H+R F HEL WT + +A E K+ PA W +L N S D

Sbjct 123 ALFHNRVFSDHELPSKAWTLDPVFKAIEPKNRPA----YWQWVL-NAAGKPKEELRLSAD 177

Query 179 AISQMGWTKGGQYLTRLSVLEATQALEGRSAVDP----NVIGGNSYYAMYFEADRKWASI 234

+M + + E VD + +S ++ + A

Sbjct 178 TYQKMALSVKVA-----PEDIRASSREWFDRVDSLSGYWALVKSSTRQLFSTDEEV-AQF 231

Query 235 SAGGGHMSLREITDPSRIAELNDAREVRLERLEKRTQFHPDDPYRTITRSPLTAAVDDVA 294

S+ + + P + + D + + + + + P + + S T D

Sbjct 232 SSEICNFDITITPAPQAVQRIADEDQAQHDV---SNGYLVNKPTHSFSFSDSTLDNTDFT 288

Query 295 DPSQAPTRLADIGP 308

I P

Sbjct 289 SVQMGGLASGGIRP 302

>WP_075337991.1 serine protease [Yersinia enterocolitica]

Length=1804

Score = 152 bits (383), Expect = 2e-37, Method: Composition-based stats.

Identities = 50/268 (19%), Positives = 89/268 (33%), Gaps = 31/268 (12%)

Query 4 LTERDLSVLGSYAR----DGNRELYWNYLSQLPGADGYGTLALGVVRNDSLPGRVANTYA 59

L DL+ + +++L+ D Y LA GV + DS+ G +A Y

Sbjct 13 LNATDLACARQLLEANKESKDPSPMYDFLASK--GDRYARLANGVAKGDSIAGAMAIHYL 70

Query 60 QDYAKSQQEEGSRFPNAQLTERQWESFGQTLLERDLELRQQWMNERRPDL-ALNLPGKDV 118

+ S N +T + L ++Q ++ + ++ +

Sbjct 71 ESVFDSH--------NQPITSIDLNDIRYDMAHGYLIMQQSRLDASPTGIIYGDIDHEQA 122

Query 119 MLAHDRAFERHELDPNCWTPRVLLQAAEQKSGPAKLEQIWTNMLNNDYAGGPRVGNTSVD 178

L H+ F ++EL WT + +A E KS PA W +L N S D

Sbjct 123 ALFHNDVFGKYELPSKAWTLDPVFKAIEPKSRPA----YWQWVL-NAAGKPKEELRLSAD 177

Query 179 AISQMGWTKGGQYLTRLSVLEATQALEGRSAVDP----NVIGGNSYYAMYF-EADRKWAS 233

+M + + E VD + +S ++ E + S

Sbjct 178 TYQKMALSAQVA-----PEDIRASSREWFDRVDSLSGYWALAKSSTRQLFSTEEEAALFS 232

Query 234 ISAGGGHMSLREITDP-SRIAELNDARE 260

+++ RIA+ + AR

Sbjct 233 SEICNFDINISPTPQAVQRIADEDQARH 260

>WP_005193300.1 serine protease [Yersinia intermedia]

EEQ16948.1 serine protease [Yersinia intermedia ATCC 29909]

CRY81016.1 putative serine protease [Yersinia intermedia]

VDZ52904.1 putative serine protease [Yersinia intermedia]

Length=1814

Score = 152 bits (383), Expect = 2e-37, Method: Composition-based stats.

Identities = 40/271 (15%), Positives = 87/271 (32%), Gaps = 30/271 (11%)

Query 4 LTERDLS----VLGSYARDGNRELYWNYLSQLPGADGYGTLALGVVRNDSLPGRVANTYA 59

L DL+ +L ++ + +++L+ D Y LA GV R DS+ G +A

Sbjct 13 LNAADLACARQLLEAHKESKDPGPMYDFLASK--GDRYAILANGVARGDSIAGAMAIHNM 70

Query 60 QDYAKSQQEEGSRFPNAQLTERQWESFGQTLLERDLELRQQWMNERRPDL-ALNLPGKDV 118

+ N +TE + + L+ +Q+ +++ + ++ +

Sbjct 71 ESV--------GGRHNKPVTETDIKHIRYDMAHGYLDTQQKRLDDSPTGIIYGDIGHEQA 122

Query 119 MLAHDRAFERHELDPNCWTPRVLLQAAEQKSGPAKLEQIWTNMLNNDYAGGPRVGNTSVD 178

H+R F H L WT + A + S P W L+

Sbjct 123 AWFHNRVFGDHGLPAEAWTLTEVFNAMTEDSRPI----YWEQTLSTGGRPFE----ELKH 174

Query 179 AISQMGWTKGGQYLTRLSVLEATQALEGRSAVDP----NVIGGNSYYAMYFEADRKWASI 234

+ + ++T + +D + +S ++ + +

Sbjct 175 SFKTYQFMAYSSSFGPDDTQKST--RQWLDRMDSLPGYWALAKSSTSQLFSSDEEVAP-V 231

Query 235 SAGGGHMSLREITDPSRIAELNDAREVRLER 265

S + + P + + D + R +

Sbjct 232 STEMCPIDINITPTPQAVQRIADEDQARRDV 262

>ARU21723.1 transcriptional regulator [Ralstonia solanacearum]

Length=1023

Score = 151 bits (382), Expect = 3e-37, Method: Composition-based stats.

Identities = 31/194 (16%), Positives = 60/194 (31%), Gaps = 13/194 (7%)

Query 4 LTERDLSVLGSYARDGNRELYWNYLSQLPGADGYGTLALGVVRNDSLPGRVANTYAQDYA 63

+ + D S L + G +++YL Y GV DS+ G A Y A

Sbjct 5 IKKDDFSRLENMLAHGQVREFYSYL--KEHGYAYAGWGRGVAMEDSISGISAIDYLTGSA 62

Query 64 KSQQEEGSRFPNAQLTERQWESFGQTLLERDLELRQQWMNE-----RRPDLALNLPGKDV 118

+T + + Q + L + + + ++ ++V

Sbjct 63 LM---GMGGEACWNITPDKSDKIKQETADAYLNTLEAIAEKNLKNTGEYEANRDINAEEV 119

Query 119 MLAHDRAFERHELDPNCWTPRVLLQAAEQKSGPAKLEQIWTNMLNNDYAGGPRVG---NT 175

H + F+ + L WT + + +Q G LE W ++ + G T

Sbjct 120 WDFHKKVFKDNGLGIENWTLDSVFKTIQQTQGDDALETYWESLRDTQGEGMMATLLNIRT 179

Query 176 SVDAISQMGWTKGG 189

+ +

Sbjct 180 MYNMHESIDSADPA 193

>WP_072102548.1 serine protease [Yersinia intermedia]

CRY55840.1 putative serine protease [Yersinia intermedia]

Length=1917

Score = 151 bits (382), Expect = 3e-37, Method: Composition-based stats.

Identities = 48/262 (18%), Positives = 95/262 (36%), Gaps = 25/262 (10%)

Query 4 LTERDLSVLGSYARDG-NRELYWNYLSQLPGADGYGTLALGVVRNDSLPGRVANTYAQDY 62

LT +L+ + L + YL D Y +LA GV R DS+ G A Y ++

Sbjct 13 LTAAELACAKEILDSKKDPALMYEYLISK--GDRYASLANGVARGDSIAGEAAIGYLKNI 70

Query 63 AKSQQEEGSRFPNAQLTERQWESFGQTLLERDLELRQQWMNERRPDLALNLPGKDVMLAH 122

A S N +TE + L L+ + + ++ ++ K+ H

Sbjct 71 AASH--------NQLVTEELINKIRFDMASEYLNLQYSRLEDSTNSISGDINHKEAGKFH 122

Query 123 DRAFERHELDPNCWTPRVLLQAAEQKSGPAKLEQIWTNMLNNDYAGGPRVGNTSVDAISQ 182

+ F++++L P WT +L+ + E W +LN+ + +S

Sbjct 123 NVVFKKYDLPPEAWTLEPVLKVMP----VEQREGYWQRVLNSAGNPSEEIL------LSI 172

Query 183 MGWTKGGQYLTRLSVLEATQALEG-RSAVDPN---VIGGNSYYAMYFEADRKWASISAGG 238

L+ E + + R+ +DP+ V+G ++ +D ++

Sbjct 173 NTVKMMIGELSNSPASEQPKIHQWLRTILDPDNAGVVGRVISSQLFSFSDDIPDAVPQCN 232

Query 239 GHMSLREITDPSRIAELNDARE 260

+++ + D R+

Sbjct 233 IDINITPSPLATEHIADEDQRQ 254

>WP_042568311.1 serine protease [Yersinia intermedia]

AJJ20890.1 proconvertase P-domain protein [Yersinia intermedia]

Length=1814

Score = 151 bits (380), Expect = 7e-37, Method: Composition-based stats.

Identities = 40/271 (15%), Positives = 85/271 (31%), Gaps = 30/271 (11%)

Query 4 LTERDLS----VLGSYARDGNRELYWNYLSQLPGADGYGTLALGVVRNDSLPGRVANTYA 59

L DL+ +L ++ + +++ + D Y LA GV + DS+ G +A

Sbjct 13 LNAADLACARQLLEAHKESKDPSPMYDFFASK--GDRYAILANGVAKGDSIAGAMAIHNM 70

Query 60 QDYAKSQQEEGSRFPNAQLTERQWESFGQTLLERDLELRQQWMNER-RPDLALNLPGKDV 118

+ + LTE + + L+ +Q ++E + ++ +

Sbjct 71 ESVGGRHNKH--------LTETDIKYIRYDMAHGYLDTQQNRLDESLTGIIYGDIGHEQA 122

Query 119 MLAHDRAFERHELDPNCWTPRVLLQAAEQKSGPAKLEQIWTNMLNNDYAGGPRVGNTSVD 178

H+ FE H L P WT + A + S P W L+

Sbjct 123 AQFHNSVFEMHGLPPEAWTLTEVFNAMTEDSRPI----YWEQTLSTGGRPFE----ELKH 174

Query 179 AISQMGWTKGGQYLTRLSVLEATQALEGRSAVDP----NVIGGNSYYAMYFEADRKWASI 234

+ ++T + +D + +S ++ + +

Sbjct 175 SFKTYQLMAYSSSFGPDDTQKST--RQWLDRMDSLPGYWALAKSSTSQLFSSDEEVAP-V 231

Query 235 SAGGGHMSLREITDPSRIAELNDAREVRLER 265

S + + P + + D + R +

Sbjct 232 STEMCPIDINITPTPQAVQRIADEDQARRDV 262

>SMH60995.1 Regulatory P domain of the subtilisin-like proprotein convertase

[Pseudomonas sp. NFIX51]

Length=2199

Score = 150 bits (379), Expect = 8e-37, Method: Composition-based stats.

Identities = 48/319 (15%), Positives = 97/319 (30%), Gaps = 38/319 (12%)

Query 1 MS-GLTERDLSVLGSYARDGNRELYWNYLSQLPGADGYGTLALGVVRNDSLPGRVANTYA 59

M GLT DL + ++YL+ Y LA GV + +S+ G VA +

Sbjct 1 MPQGLTVVDLDYAKALLSSAGPGAMYDYLAV--RGYKYAVLANGVAKGNSIAGEVAINFM 58

Query 60 QDYAKSQQEEGSRFPNAQLTERQWESFGQTLLERDLELRQQWMNERRPDLALNLPGKDVM 119

+ A ++E + + + ++ + ++ ++ ++

Sbjct 59 KTTAAD--------AGHVMSEDDVNRIRRQMASEYVSTLSDKLDA-KGVVSADITYEEAW 109

Query 120 LAHDRAFERHELDPNCWTPRVLLQAAEQKSGPAKLEQIWTNMLNNDYAGGPRVGNTSVDA 179

H + F+ + L + WT + + G W N+L+ G+ +

Sbjct 110 GFHRKVFDNNGLSVDAWTLNSVFEVL----GGNSRAVYWGNVLD-------FAGSPGWEL 158

Query 180 ISQMGWTKGGQYLTRLSVLEATQA-LEGRSAVDP----NVIGGNSYYAMYFEADRKWAS- 233

K R+ E Q + + VD + + +Y KW

Sbjct 159 ALSYETDKLMALAARIGTAETKQIAMNWINRVDSPSGDWTVVTSLTSQVY-----KWVRG 213

Query 234 -ISAGGGHMSLREITDPSRIAELNDAREVRLE---RLEKRTQFHPDDPYRTITRSPLTAA 289

I MS+ + + + ++ E + + F + +I

Sbjct 214 LIVDEAPDMSVPNLINIDINPSPQPQQHIQGESKAQQDVANGFIQNSATNSIFAVGGILN 273

Query 290 VDDVADPSQAPTRLADIGP 308

D A I P

Sbjct 274 KTDFTSTQMASLASGGIRP 292

>WP_054889361.1 peptidase S8 [Pseudomonas sp. NBRC 111118]

Length=1807

Score = 150 bits (379), Expect = 1e-36, Method: Composition-based stats.

Identities = 40/229 (17%), Positives = 78/229 (34%), Gaps = 27/229 (12%)

Query 1 MSGLTERDLSVLGSYARDGNRELYWNYLSQLPGADGYGTLALGVVRNDSLPGRVANTYAQ 60

MSGLT+ D++V ++Y++ D Y LA GV + +S+ G A + +

Sbjct 1 MSGLTKEDIAVARKIFESKGVGDMYDYMAMK--GDRYAVLANGVAKGNSIAGLAAINFMK 58

Query 61 DYAKSQQEEGSRFPNAQLTERQWESFGQTLLERDLELRQQWMNERRPDLALNLPGKDVML 120

++E E + + LEL+++ + + L + +V

Sbjct 59 RTEAD--------AGRPMSEADVEEVRLEMGKAYLELQERRVMDG--TLDGDPNHMEVWD 108

Query 121 AHDRAFERHELDPNCWTPRVLLQAAEQKSGPAKLEQIWTNMLNNDYAGGPRVGNTSVDAI 180

H F+ + WT + + P E W +L+ + G+ +I

Sbjct 109 FHSDVFKSLGRSKDAWTLNSVFELT----DPEYRESYWKKVLDAAGSP----GSEFWLSI 160

Query 181 SQMGWTKGGQYLTRLSVLEATQALEGRSAVD-PNVIGGNSYYAMYFEAD 228

+ + A +S +D P I +Y +

Sbjct 161 QTDSRMANSTVVAPDDM--RGMAKYWKSRIDSPTAIA----SFLYSIGE 203

>WP_083203292.1 serine protease [Pseudomonas sp. 24 E 13]

CRM78955.1 Microbial serine proteinase precursor [Pseudomonas sp. 24 E 13]

Length=2201

Score = 150 bits (379), Expect = 1e-36, Method: Composition-based stats.

Identities = 52/313 (17%), Positives = 93/313 (30%), Gaps = 29/313 (9%)

Query 4 LTERDLSVLGSYARDGNRELYWNYLSQLPGADGYGTLALGVVRNDSLPGRVANTYAQDYA 63

LT DL +++LS Y TLA GV + +SL G A + + A

Sbjct 5 LTAVDLEQAKRILDSSGPSAAYDFLS--GKGYKYATLANGVAKGNSLSGEAAINFMKVTA 62

Query 64 KSQQEEGSRFPNAQLTERQWESFGQTLLERDLELRQQWMNERRPDLALNLPGKDVMLAHD 123

+ L++ + + +E + +N L ++ + H

Sbjct 63 DASGH--------PLSDVDVNRIRKDMANGYIEALRAKLNNASGVLNSDINYDEAWNFHR 114

Query 124 RAFERHELDPNCWTPRVLLQAAEQKSGPAKLEQIWTNMLNNDYAGGPRVGNTSVDAISQM 183

F + L + WT + + E W +L + G+ S + I

Sbjct 115 NVFNTNGLSVDAWTLNSVFSVI----TESTREAYWQTVLES-------AGDVSKELILAA 163

Query 184 GWTKGGQYLTRLSVLEA-TQALEGRSAVDP----NVIGGNSYYAMYFEADRKWASISAGG 238

K T +S + A VD + + + MY +A S

Sbjct 164 NTDKMMSLATTISTDDNKKLAKGWIDRVDSPSGAYTVTKSLFSQMYNMITGGFAEESNIP 223

Query 239 GHMSLREITDPSRIAELNDAREVRLE---RLEKRTQFHPDDPYRTITRSPLTAAVDDVAD 295

S + D + + ++ E R + + + P +I T D

Sbjct 224 PSASTSSLIDINITPDPQPQHNIQGENKARQDVSNGYVVNSPTHSIVFKDDTLNKTDFTS 283

Query 296 PSQAPTRLADIGP 308

I P

Sbjct 284 TQIGSLTTGGIRP 296

>WP_050085098.1 serine protease [Yersinia intermedia]

CNB43454.1 putative serine protease [Yersinia intermedia]

CRE69283.1 putative serine protease [Yersinia intermedia]

Length=1814

Score = 150 bits (378), Expect = 1e-36, Method: Composition-based stats.

Identities = 40/266 (15%), Positives = 84/266 (32%), Gaps = 30/266 (11%)

Query 4 LTERDLS----VLGSYARDGNRELYWNYLSQLPGADGYGTLALGVVRNDSLPGRVANTYA 59

L DL+ +L ++ + +++L+ D Y LA GV DS+ G +A

Sbjct 13 LNAADLACARQLLEAHKESKDPGPMYDFLASK--GDRYAILANGVAMGDSIAGAMAIHNM 70

Query 60 QDYAKSQQEEGSRFPNAQLTERQWESFGQTLLERDLELRQQWMNERRPDL-ALNLPGKDV 118

+ N +TE + + L+ +Q+ +++ + ++ K

Sbjct 71 ESV--------GGRHNKPVTETDIKHIRYGMAHGYLDTQQKRLDDSPTGIIYGDISHKQA 122

Query 119 MLAHDRAFERHELDPNCWTPRVLLQAAEQKSGPAKLEQIWTNMLNNDYAGGPRVGNTSVD 178

H+ FE H L P WT + A + S P W L+

Sbjct 123 AQFHNSVFEMHGLPPEAWTLTEVFNAMTEDSQPI----YWEQTLSTGGRPFE----ELKH 174

Query 179 AISQMGWTKGGQYLTRLSVLEATQALEGRSAVDP----NVIGGNSYYAMYFEADRKWASI 234

+ ++T + +D + +S ++ + +

Sbjct 175 SFKTYQLMAYSSSFGPDDTQKST--RQWLDRMDSLPGYWALAKSSTSQLFSSDEEVAP-V 231

Query 235 SAGGGHMSLREITDPSRIAELNDARE 260

S + + P + + D +

Sbjct 232 STEMCPIDINITPTPQAVQRIADEDQ 257

>PNG84519.1 Microbial serine proteinase precursor [Pseudomonas putida]

Length=1807

Score = 149 bits (377), Expect = 2e-36, Method: Composition-based stats.

Identities = 40/229 (17%), Positives = 78/229 (34%), Gaps = 27/229 (12%)

Query 1 MSGLTERDLSVLGSYARDGNRELYWNYLSQLPGADGYGTLALGVVRNDSLPGRVANTYAQ 60

MSGLT+ D++ S ++Y++ D Y LA GV + +S+ G A + +

Sbjct 1 MSGLTKEDIAAARSIFESKGVGDMYDYMAMK--GDRYAVLANGVAKGNSIAGLAAINFMK 58

Query 61 DYAKSQQEEGSRFPNAQLTERQWESFGQTLLERDLELRQQWMNERRPDLALNLPGKDVML 120

++E E + + LEL+++ + + L + +V

Sbjct 59 RTEAD--------AGRPMSEADVEEVRLEMGKAYLELQERRVMDG--TLDGDPNHMEVWD 108

Query 121 AHDRAFERHELDPNCWTPRVLLQAAEQKSGPAKLEQIWTNMLNNDYAGGPRVGNTSVDAI 180

H F+ + WT + + P E W +L+ + G+ +I

Sbjct 109 FHSDVFKSLGRSKDAWTLNSVFELT----DPEYRESYWKKVLDAAGSP----GSEFWLSI 160

Query 181 SQMGWTKGGQYLTRLSVLEATQALEGRSAVD-PNVIGGNSYYAMYFEAD 228

+ + A +S +D P I +Y +

Sbjct 161 QTDSRMANSTVVAPDDM--RGMAKYWKSRIDSPTAIA----SFLYSIGE 203

>WP_050311379.1 serine protease [Yersinia intermedia]

CNI63126.1 putative serine protease [Yersinia intermedia]

CQD99427.1 putative serine protease [Yersinia intermedia]

Length=1814

Score = 149 bits (377), Expect = 2e-36, Method: Composition-based stats.

Identities = 39/266 (15%), Positives = 83/266 (31%), Gaps = 30/266 (11%)

Query 4 LTERDLS----VLGSYARDGNRELYWNYLSQLPGADGYGTLALGVVRNDSLPGRVANTYA 59

L DL+ +L ++ + +++ + D Y LA GV + DS+ G +A

Sbjct 13 LNAADLACARQLLEAHKESKDPSPMYDFFASK--GDRYAILANGVAKGDSIAGAMAIHNM 70

Query 60 QDYAKSQQEEGSRFPNAQLTERQWESFGQTLLERDLELRQQWMNER-RPDLALNLPGKDV 118

+ + LTE + + L+ +Q ++E + ++ +

Sbjct 71 ESVGGRHNKH--------LTETDIKYIRYDMAHGYLDTQQNRLDESLTGIIYGDIGHEQA 122

Query 119 MLAHDRAFERHELDPNCWTPRVLLQAAEQKSGPAKLEQIWTNMLNNDYAGGPRVGNTSVD 178

H+ FE H L P WT + A + S P W L+

Sbjct 123 AQFHNSVFEMHGLPPEAWTLTEVFNAMTEDSRPI----YWEQTLSTGGRPFE----ELKH 174

Query 179 AISQMGWTKGGQYLTRLSVLEATQALEGRSAVDP----NVIGGNSYYAMYFEADRKWASI 234

+ ++T + +D + +S ++ + +

Sbjct 175 SFKTYQLMAYSSSFGPDDTQKST--RQWLDRMDSLPGYWALAKSSTSQLFSSDEEVAP-V 231

Query 235 SAGGGHMSLREITDPSRIAELNDARE 260

S + + P + + D +

Sbjct 232 STEMCPIDINITPTPQAVQRIADEDQ 257

>WP_070095405.1 peptidase S8 [Pseudomonas sp. NBRC 111139]

Length=1807

Score = 149 bits (377), Expect = 2e-36, Method: Composition-based stats.

Identities = 40/229 (17%), Positives = 78/229 (34%), Gaps = 27/229 (12%)

Query 1 MSGLTERDLSVLGSYARDGNRELYWNYLSQLPGADGYGTLALGVVRNDSLPGRVANTYAQ 60

MSGLT+ D++V ++Y++ D Y LA GV + +S+ G A + +

Sbjct 1 MSGLTKEDIAVARKIFESKGVGDMYDYMAVK--GDRYAVLANGVAKGNSIAGLAAINFMK 58

Query 61 DYAKSQQEEGSRFPNAQLTERQWESFGQTLLERDLELRQQWMNERRPDLALNLPGKDVML 120

++E E + + LEL+++ + + L + +V

Sbjct 59 RTEAD--------AGRPMSEADVEEVRLEMGKAYLELQERRVMDG--TLDGDPNHMEVWD 108

Query 121 AHDRAFERHELDPNCWTPRVLLQAAEQKSGPAKLEQIWTNMLNNDYAGGPRVGNTSVDAI 180

H F+ + WT + + P E W +L+ + G+ +I

Sbjct 109 FHSDVFKSLGRSKDAWTLNSVFELT----DPEYRESYWKKVLDAAGSP----GSEFWLSI 160

Query 181 SQMGWTKGGQYLTRLSVLEATQALEGRSAVD-PNVIGGNSYYAMYFEAD 228

+ + A +S +D P I +Y +

Sbjct 161 QTDSRMANSTVVAPDDM--RGMAKYWKSRIDSPTAIA----SFLYSIGE 203

>CNJ21697.1 putative serine protease [Yersinia intermedia]

Length=1465

Score = 149 bits (376), Expect = 2e-36, Method: Composition-based stats.

Identities = 40/271 (15%), Positives = 85/271 (31%), Gaps = 30/271 (11%)

Query 4 LTERDLS----VLGSYARDGNRELYWNYLSQLPGADGYGTLALGVVRNDSLPGRVANTYA 59

L DL+ +L ++ + +++ + D Y LA GV + DS+ G +A

Sbjct 13 LNAADLACARQLLEAHKESKDPSPMYDFFASK--GDRYAILANGVAKGDSIAGAMAIHNM 70

Query 60 QDYAKSQQEEGSRFPNAQLTERQWESFGQTLLERDLELRQQWMNER-RPDLALNLPGKDV 118

+ + LTE + + L+ +Q ++E + ++ +

Sbjct 71 ESVGGRHNKH--------LTETDIKYIRYDMAHGYLDTQQNRLDESLTGIIYGDIGHEQA 122

Query 119 MLAHDRAFERHELDPNCWTPRVLLQAAEQKSGPAKLEQIWTNMLNNDYAGGPRVGNTSVD 178

H+ FE H L P WT + A + S P W L+

Sbjct 123 AQFHNSVFEMHGLPPEAWTLTEVFNAMTEDSRPI----YWEQTLSTGGRPFE----ELKH 174

Query 179 AISQMGWTKGGQYLTRLSVLEATQALEGRSAVDP----NVIGGNSYYAMYFEADRKWASI 234

+ ++T + +D + +S ++ + +

Sbjct 175 SFKTYQLMAYSSSFGPDDTQKST--RQWLDRMDSLPGYWALAKSSTSQLFSSDEEVAP-V 231

Query 235 SAGGGHMSLREITDPSRIAELNDAREVRLER 265

S + + P + + D + R +

Sbjct 232 STEMCPIDINITPTPQAVQRIADEDQARRDV 262

>WP_052196249.1 peptidase S8 [Pseudomonas putida]

Length=1807

Score = 149 bits (377), Expect = 2e-36, Method: Composition-based stats.

Identities = 40/229 (17%), Positives = 78/229 (34%), Gaps = 27/229 (12%)

Query 1 MSGLTERDLSVLGSYARDGNRELYWNYLSQLPGADGYGTLALGVVRNDSLPGRVANTYAQ 60

MSGLT+ D++ S ++Y++ D Y LA GV + +S+ G A + +

Sbjct 1 MSGLTKEDIAAARSIFESKGVGDMYDYMAMK--GDRYAVLANGVAKGNSIAGLAAINFMK 58

Query 61 DYAKSQQEEGSRFPNAQLTERQWESFGQTLLERDLELRQQWMNERRPDLALNLPGKDVML 120

++E E + + LEL+++ + + L + +V

Sbjct 59 RTEAD--------AGRPMSEADVEEVRLEMGKAYLELQERRVMDG--TLDGDPNHMEVWD 108

Query 121 AHDRAFERHELDPNCWTPRVLLQAAEQKSGPAKLEQIWTNMLNNDYAGGPRVGNTSVDAI 180

H F+ + WT + + P E W +L+ + G+ +I

Sbjct 109 FHSDVFKSLGRSKDAWTLNSVFELT----DPEYRESYWKKVLDAAGSP----GSEFWLSI 160

Query 181 SQMGWTKGGQYLTRLSVLEATQALEGRSAVD-PNVIGGNSYYAMYFEAD 228

+ + A +S +D P I +Y +

Sbjct 161 QTDSRMANSTVVAPDDM--RGMAKYWKSRIDSPTAIA----SFLYSIGE 203

>WP_050296718.1 MULTISPECIES: serine protease [Yersinia]

ARB84452.1 serine protease [Yersinia sp. FDAARGOS_228]

AVL34228.1 serine protease [Yersinia intermedia]

CNC67837.1 putative serine protease [Yersinia intermedia]

Length=1814

Score = 149 bits (377), Expect = 2e-36, Method: Composition-based stats.

Identities = 39/266 (15%), Positives = 83/266 (31%), Gaps = 30/266 (11%)

Query 4 LTERDLS----VLGSYARDGNRELYWNYLSQLPGADGYGTLALGVVRNDSLPGRVANTYA 59

L DL+ +L ++ + +++ + D Y LA GV + DS+ G +A

Sbjct 13 LNAADLACARQLLEAHKESKDPSPMYDFFASK--GDRYAILANGVAKGDSIAGAMAIHNM 70

Query 60 QDYAKSQQEEGSRFPNAQLTERQWESFGQTLLERDLELRQQWMNER-RPDLALNLPGKDV 118

+ + LTE + + L+ +Q ++E + ++ +

Sbjct 71 ESVGGRHNKH--------LTETDIKYIRYDMAHGYLDTQQNRLDESLTGIIYGDIGHEQA 122

Query 119 MLAHDRAFERHELDPNCWTPRVLLQAAEQKSGPAKLEQIWTNMLNNDYAGGPRVGNTSVD 178

H+ FE H L P WT + A + S P W L+

Sbjct 123 AQFHNSVFEMHGLPPEAWTLTEVFNAMTEDSRPI----YWEQTLSTGGRPFE----ELKH 174

Query 179 AISQMGWTKGGQYLTRLSVLEATQALEGRSAVDP----NVIGGNSYYAMYFEADRKWASI 234

+ ++T + +D + +S ++ + +

Sbjct 175 SFKTYQLMAYSSSFGPDDTQKST--RQWLDRMDSLPGYWALAKSSTSQLFSSDEEVAP-V 231

Query 235 SAGGGHMSLREITDPSRIAELNDARE 260

S + + P + + D +

Sbjct 232 STEMCPIDINITPTPQAVQRIADEDQ 257

>WP_084254674.1 tandem-95 repeat protein [Cupriavidus oxalaticus]

SPC10803.1 RTX toxin exported protein [Cupriavidus oxalaticus]

Length=2456

Score = 149 bits (377), Expect = 2e-36, Method: Composition-based stats.

Identities = 39/174 (22%), Positives = 67/174 (39%), Gaps = 8/174 (5%)

Query 4 LTERDLSVLGSYARDGNRELYWNYLSQLPGADGYGTLALGVVRNDSLPGRVANTYAQDYA 63

++ + L+ + G Y Y A GV +++ G A TY Q++A

Sbjct 5 ISAQSLNEIRFQLNSGKLTPSQVYSQFEQYGYKYAGWAGGVADANTIAGASALTYMQNFA 64

Query 64 KSQQEEGSRFPNAQLTERQWESFGQTLLERDLELRQQWMNERRPDLALNLPGKDVMLAHD 123

K + LT Q E + + L+ Q NE + ++ ++V H

Sbjct 65 KELGK--------PLTGVQIERIKLDMAKGYLDALYQQTNEGSLPVTRDINSREVWNFHS 116

Query 124 RAFERHELDPNCWTPRVLLQAAEQKSGPAKLEQIWTNMLNNDYAGGPRVGNTSV 177

+ FE + L P WT + E+ GPA++EQ W + + A G +

Sbjct 117 KVFEDNGLRPGAWTLDTPFRVIEKMGGPAQVEQYWNMLRDTGGAYGDALSANIY 170

>WP_064492578.1 peptidase S8 [Pseudomonas putida]

OAS05222.1 peptidase S8 [Pseudomonas putida]

Length=1807

Score = 149 bits (376), Expect = 3e-36, Method: Composition-based stats.

Identities = 41/230 (18%), Positives = 81/230 (35%), Gaps = 29/230 (13%)

Query 1 MSGLTERDLSVLGSYARDGNRELYWNYLSQLPGADGYGTLALGVVRNDSLPGRVANTYAQ 60

MSGLT+ D++V ++Y++ D Y LA GV + +S+ G A + +

Sbjct 1 MSGLTKEDIAVARKIFESKGVGDMYDYMAMK--GDRYAVLANGVAKGNSIAGLAAINFMK 58

Query 61 DYAKSQQEEGSRFPNAQLTERQWESFGQTLLERDLELRQQWMNERRPDLALNLPGKDVML 120

++E E + + LEL+++ + + + K+V

Sbjct 59 RTEAD--------AGRPMSEADVEEVRLEMGKAYLELQERRVM--GGKVDGDPNHKEVWA 108

Query 121 AHDRAFERHELDPNCWTPRVLLQAAEQKSGPAKLEQIWTNMLNNDYAGGPRVGNTSVDAI 180

H + F+ + WT + + P E W +L+ G+ +

Sbjct 109 FHSKVFKNLGRSKDAWTLNSVFELTH----PEHRESYWEKVLD-------AAGSPGSEFW 157

Query 181 SQMGWTKGGQYLTRLSVLEA-TQALEGRSAVD-PNVIGGNSYYAMYFEAD 228

+ Y T ++ + A +S +D P I +Y +

Sbjct 158 LSIQTDSRMAYSTVVAPDDMRGMAKYWKSRIDSPTAIA----SFLYSIGE 203

>WP_087816309.1 serine protease [Yersinia intermedia]

OVZ84769.1 serine protease [Yersinia intermedia]

Length=1814

Score = 149 bits (376), Expect = 3e-36, Method: Composition-based stats.

Identities = 39/266 (15%), Positives = 83/266 (31%), Gaps = 30/266 (11%)

Query 4 LTERDLS----VLGSYARDGNRELYWNYLSQLPGADGYGTLALGVVRNDSLPGRVANTYA 59

L DL+ +L ++ + +++L+ D Y LA GV DS+ G +A

Sbjct 13 LNAADLACARQLLEAHKESKDPGPMYDFLASK--GDRYAILANGVAMGDSIAGAMAIHNM 70

Query 60 QDYAKSQQEEGSRFPNAQLTERQWESFGQTLLERDLELRQQWMNERRPDL-ALNLPGKDV 118

+ N +TE + + L+ +Q+ +++ + ++ K

Sbjct 71 ESV--------GGRHNKPVTETDIKHIRYGMAHGYLDTQQKRLDDSPTGIIYGDISHKQA 122

Query 119 MLAHDRAFERHELDPNCWTPRVLLQAAEQKSGPAKLEQIWTNMLNNDYAGGPRVGNTSVD 178

H+ FE H L WT + A + S P W L+

Sbjct 123 AQFHNSVFEMHGLPAEAWTLTEVFNAMTEDSRPI----YWEQTLSTGGRPFE----ELKH 174

Query 179 AISQMGWTKGGQYLTRLSVLEATQALEGRSAVDP----NVIGGNSYYAMYFEADRKWASI 234

+ ++T + +D + +S ++ + +

Sbjct 175 SFKTYQLMAYSSSFGPDDTQKST--RQWLDRMDSLPGYWALAKSSTSQLFSSDEEVAP-V 231

Query 235 SAGGGHMSLREITDPSRIAELNDARE 260

S + + P + + D +

Sbjct 232 STEMCPIDINITPTPQAVQRIADEDQ 257

>WP_049614359.1 serine protease [Yersinia pekkanenii]

CNI23072.1 putative serine protease [Yersinia pekkanenii]

CRY67357.1 putative serine protease [Yersinia pekkanenii]

Length=1816

Score = 149 bits (375), Expect = 3e-36, Method: Composition-based stats.

Identities = 41/266 (15%), Positives = 92/266 (35%), Gaps = 30/266 (11%)

Query 4 LTERDLS----VLGSYARDGNRELYWNYLSQLPGADGYGTLALGVVRNDSLPGRVANTYA 59

L DL+ +L ++ + +++L+ D Y LA GVV+ +S+ GR+A Y

Sbjct 13 LNAADLACARQLLEAHKESKDPGPMYDFLASK--GDRYAILANGVVKENSIAGRIAINYM 70

Query 60 QDYAKSQQEEGSRFPNAQLTERQWESFGQTLLERDLELRQQWMNERRPDL-ALNLPGKDV 118

+D S + +TE + G + + +Q +++ + ++ +

Sbjct 71 RDVGVSHNK--------PVTETDIKHIGYDMARGYFDTQQDRLDKSPTGIIYGDIDHEQA 122

Query 119 MLAHDRAFERHELDPNCWTPRVLLQAAEQKSGPAKLEQIWTNMLNNDYAGGPRVGNTSVD 178

H+ F+ H L P WT + + + W + LN+ +

Sbjct 123 AQFHNSVFKTHGLPPEAWTLYPVYKVLPKNQHGG----HWQSTLNSAGNSADEIL----- 173

Query 179 AISQMGWTKGGQYLTRLSVLEATQALEGRSAV-DPNVIGGNS---YYAMYFEADRKWASI 234

+S L+ + + V D + +G + ++ + +

Sbjct 174 -LSFNSVKIMVDALSTSPASDQPMIHNWLNIVMDADNLGAAAGAIGSQLFSSDEEVAP-V 231

Query 235 SAGGGHMSLREITDPSRIAELNDARE 260

S + + P + + D +

Sbjct 232 STEMCPIDINITPTPQAVQRIADEDQ 257

>WP_049634978.1 serine protease [Yersinia aldovae]

CNI10567.1 putative serine protease [Yersinia aldovae]

Length=1806

Score = 148 bits (374), Expect = 5e-36, Method: Composition-based stats.

Identities = 46/314 (15%), Positives = 92/314 (29%), Gaps = 33/314 (11%)

Query 4 LTERDLSVLGSYAR----DGNRELYWNYLSQLPGADGYGTLALGVVRNDSLPGRVANTYA 59

L DL+ + +++L+ D Y LA GV R DS+ G +A

Sbjct 13 LNAADLAYARQLLEANKESKDPGPMYDFLASK--GDRYAILANGVARGDSIAGGMAIHNM 70

Query 60 QDYAKSQQEEGSRFPNAQLTERQWESFGQTLLERDLELRQQWMNERRPDL-ALNLPGKDV 118

+ N L+E ++ G + L +Q ++ + ++ K

Sbjct 71 ERV--------GGRHNKPLSETHIKNIGYDMAGGYLNTQQSRLDNSPTGIIYGDIDHKQA 122

Query 119 MLAHDRAFERHELDPNCWTPRVLLQAAEQKSGPAKLEQIWTNMLNNDYAGGPRVGNTSVD 178

H+ FE + L P WT + A + S P W L+

Sbjct 123 AQFHNSVFEMYGLPPEAWTLTEVFNAMTKDSRPI----YWEQTLSTCGRPFE----ELKH 174

Query 179 AISQMGWTKGGQYLTRLSVLEATQALEGRSAVDP----NVIGGNSYYAMYFEADRKWASI 234

+ +++ +D + +S ++ + +

Sbjct 175 SFKTYQLMAYSSSFGPDDTQKSS--RAWLDRMDSLPGYWALAKSSTSQLFSSDEGV-VPV 231

Query 235 SAGGGHMSLREITDPSRIAELNDAREVRLERLEKRTQFHPDDPYRTITRSPLTAAVDDVA 294

S ++ + P + + D + R + + + P + + S T D

Sbjct 232 STEMCNIDITINPTPQAVQRIADEDQ---ARRDVTNGYLVNKPTHSFSFSDSTLNNTDFT 288

Query 295 DPSQAPTRLADIGP 308

I P

Sbjct 289 SVQMGSMASGGIRP 302

>WP_082632033.1 serine protease [Pseudomonas orientalis]

SDU23666.1 Regulatory P domain of the subtilisin-like proprotein convertase

[Pseudomonas orientalis]

Length=2201

Score = 148 bits (373), Expect = 5e-36, Method: Composition-based stats.

Identities = 52/313 (17%), Positives = 94/313 (30%), Gaps = 29/313 (9%)

Query 4 LTERDLSVLGSYARDGNRELYWNYLSQLPGADGYGTLALGVVRNDSLPGRVANTYAQDYA 63

LT DL +++LS Y TLA GV + +SL G A + + A

Sbjct 5 LTAVDLEQAKRILDSSGPSAAYDFLS--GKGYKYATLANGVAKGNSLSGEAAINFMKVTA 62

Query 64 KSQQEEGSRFPNAQLTERQWESFGQTLLERDLELRQQWMNERRPDLALNLPGKDVMLAHD 123

+ L++ + + +E + +N L ++ + H

Sbjct 63 DASGH--------PLSDVDVNRIRKDMANGYIEALRAKLNNASGVLNSDINYDEAWNFHR 114

Query 124 RAFERHELDPNCWTPRVLLQAAEQKSGPAKLEQIWTNMLNNDYAGGPRVGNTSVDAISQM 183

F + L + WT + + E W +L + G+ S + I

Sbjct 115 NVFNTNGLSVDAWTLNSVFSVI----TESTREAYWQTVLES-------AGDVSKELILAA 163

Query 184 GWTKGGQYLTRLSVLEA-TQALEGRSAVDP----NVIGGNSYYAMYFEADRKWASISAGG 238

K T +S + A VD + + + MY +A S

Sbjct 164 NTDKMMSLATTISTDDNKKLAKGWIDRVDSPSGAYTVTKSLFSQMYNMITGGFAEESNIP 223

Query 239 GHMSLREITDPSRIAELNDAREVRLE---RLEKRTQFHPDDPYRTITRSPLTAAVDDVAD 295

S + D + + + ++ E R + + + P +I T D

Sbjct 224 PSASTSGLIDINITPDPQPQQNIQGENKARQDVSNGYVVNSPTHSIVFKDDTLNKTDFTS 283

Query 296 PSQAPTRLADIGP 308

I P

Sbjct 284 TQIGSLTTGGIRP 296

>WP_084562436.1 calcium-binding protein [Snodgrassella alvi]

ORF02741.1 hypothetical protein BGH97_04470 [Snodgrassella alvi]

ORF08598.1 hypothetical protein BGH99_05020 [Snodgrassella alvi]

ORF10400.1 hypothetical protein BGI00_09475 [Snodgrassella alvi]

ORF14914.1 hypothetical protein BGI02_04300 [Snodgrassella alvi]

ORF19420.1 hypothetical protein BGI05_08030 [Snodgrassella alvi]

ORF23878.1 hypothetical protein BGI06_07875 [Snodgrassella alvi]

Length=1585

Score = 148 bits (372), Expect = 7e-36, Method: Composition-based stats.

Identities = 37/213 (17%), Positives = 73/213 (34%), Gaps = 23/213 (11%)

Query 4 LTERDLSVLGSYARDGNRELYWN-YLSQLPGADGYGTLALGVVRNDSLPGRVANTYAQDY 62

LT+ L+ + GN Y Y A+GV DS+ G A + Q

Sbjct 7 LTKEQLNSWNQKIKTGNLSAIGEVYQILQEKGYNYAAWAIGVATGDSITGNGALEFMQTV 66

Query 63 AKSQQEEGSRFPNAQLTERQWESFGQTLLERDLELRQQWMNERRPDLALNLPGKDVMLAH 122

AK LT+ + +S + + L + Q + ++ +++ H

Sbjct 67 AKDH--------KQILTQARIDSVRRDMAIGYLAMLQNKLEHGHGG--EDITYEEMYEFH 116

Query 123 DRAFERHELDPNCWTPRVLLQAAEQKS-----------GPAKLEQIWTNMLNNDYAGGPR 171

F ++ LD + WT + + + G +E +W +

Sbjct 117 VNVFNKNGLDISYWTLYTPMSIIQTNASGNGRNGNMIEGSKVVESMWEQIRATKGEVVNG 176

Query 172 VGNTSVDAISQM-GWTKGGQYLTRLSVLEATQA 203

+ S++ M KG Y+ +++ ++Q

Sbjct 177 GASVSLELYQIMQDAKKGYIYVDKITGDVSSQT 209

>WP_087784791.1 serine protease [Yersinia intermedia]

OVZ74465.1 serine protease [Yersinia intermedia]

Length=1814

Score = 148 bits (372), Expect = 7e-36, Method: Composition-based stats.

Identities = 43/271 (16%), Positives = 94/271 (35%), Gaps = 30/271 (11%)

Query 4 LTERDLS----VLGSYARDGNRELYWNYLSQLPGADGYGTLALGVVRNDSLPGRVANTYA 59

L DL+ +L + + +++L+ D Y LA GVV+ +S+ GR+A Y

Sbjct 13 LNTADLACAKQLLEANKESKDPGPMYDFLASK--GDRYAILANGVVKENSIAGRIAINYM 70

Query 60 QDYAKSQQEEGSRFPNAQLTERQWESFGQTLLERDLELRQQWMNER-RPDLALNLPGKDV 118

+D S + LTE + + + +Q+ +++ + ++ K

Sbjct 71 RDVGVSHNKH--------LTETDIKHIRYDMARGYFDTQQKRLDDSLTGIIYGDIDHKQA 122

Query 119 MLAHDRAFERHELDPNCWTPRVLLQAAEQKSGPAKLEQIWTNMLNNDYAGGPRVGNTSVD 178

H+ F++H L P WT + + + W + LN+ +

Sbjct 123 AQFHNSVFKKHGLPPEAWTLYPVYKVLPKNQHGG----HWQSTLNSAGNSADEIL----- 173

Query 179 AISQMGWTKGGQYLTRLSVLEATQALEGRSAV-DPNVIGGNSYY---AMYFEADRKWASI 234

+S L+ + + V D + +G + ++ + +

Sbjct 174 -LSFNTVKIMVDALSTSPASDQPMIHNWLNIVMDADNLGAAAGAIGNQLFSSDEEVAP-V 231

Query 235 SAGGGHMSLREITDPSRIAELNDAREVRLER 265

S + + P + + D + R +

Sbjct 232 STEMCPIDINITPTPQAVQRIADEDQARRDV 262

>WP_005966781.1 calcium-binding protein [endosymbiont of Riftia pachyptila]

EGV49774.1 hemolysin-type calcium binding protein [endosymbiont of Riftia

pachyptila (vent Ph05)]

Length=1018

Score = 146 bits (367), Expect = 3e-35, Method: Composition-based stats.

Identities = 27/172 (16%), Positives = 57/172 (33%), Gaps = 4/172 (2%)

Query 3 GLTERDLSVLGSYARDGNRELYWN-YLSQLPGADGYGTLALGVVRNDSLPGRVANTYAQD 61

+T + L G + Y Y A GV D++ G+ A Y +

Sbjct 4 TITRQQLVDYRRRIESGGVKAVRQVYAELYGKGYNYAGWAAGVANGDTVAGQAALNYLKG 63

Query 62 YAKSQQEEGSRFPNAQLTERQWESFGQTLLERDLELRQQWMNERRPDLALNLPGKDVMLA 121

A L+ + ++ + E ++ ++ + ++ + M

Sbjct 64 TALM---GMGGEECINLSLAEIDNIRVDMAEGYIKALISNADQNGGWIDRDVRYNETMKF 120

Query 122 HDRAFERHELDPNCWTPRVLLQAAEQKSGPAKLEQIWTNMLNNDYAGGPRVG 173

H AFE++ L WT ++ ++ +E +W + + G V

Sbjct 121 HQEAFEKNNLSLENWTLYTPMELIRKEYDDQAVEDLWAQIRDTGGDGPDAVL 172

>POG07288.1 peptidase S8 [Pseudomonas putida]

Length=1807

Score = 146 bits (367), Expect = 4e-35, Method: Composition-based stats.

Identities = 41/230 (18%), Positives = 80/230 (35%), Gaps = 29/230 (13%)

Query 1 MSGLTERDLSVLGSYARDGNRELYWNYLSQLPGADGYGTLALGVVRNDSLPGRVANTYAQ 60

MS LT+ D++V ++Y++ D Y LA GV + +S+ G A + +

Sbjct 1 MSVLTKEDIAVARKIFESKGVGDMYDYMAMK--GDRYAVLANGVAKGNSIAGLAAINFMK 58

Query 61 DYAKSQQEEGSRFPNAQLTERQWESFGQTLLERDLELRQQWMNERRPDLALNLPGKDVML 120

++E E + + LEL+++ + + + K+V

Sbjct 59 RTEAD--------AGRPMSEADVEEVRLEMGKAYLELQERRVM--GGKVDGDPNHKEVWA 108

Query 121 AHDRAFERHELDPNCWTPRVLLQAAEQKSGPAKLEQIWTNMLNNDYAGGPRVGNTSVDAI 180

H R F+ + WT + + P E W +L+ G+ +

Sbjct 109 FHSRVFKNLGRSKDAWTLNSVFELTH----PEHRESYWEKVLD-------AAGSPGSEFW 157

Query 181 SQMGWTKGGQYLTRLSVLEA-TQALEGRSAVD-PNVIGGNSYYAMYFEAD 228

+ Y T ++ + A +S +D P I +Y +

Sbjct 158 LSIQTDSRMAYSTVVAPDDMRGMAKYWKSRIDSPTAIA----SFLYSIGE 203

>WP_080996553.1 hypothetical protein [Pseudomonas corrugata]

SDU89544.1 Regulatory P domain of the subtilisin-like proprotein convertase

[Pseudomonas corrugata]

Length=2331

Score = 145 bits (366), Expect = 4e-35, Method: Composition-based stats.

Identities = 27/177 (15%), Positives = 54/177 (31%), Gaps = 14/177 (8%)

Query 4 LTERDLSVLGSYARDGNRELYWNYLSQLPGADGYGTLALGVVRNDSLPGRVANTYAQDYA 63

L + DL S ++YL+ Y LA GV + +SL G A Y ++ A

Sbjct 5 LKQADLDYAQSLLGSNGPAAMYDYLA--ARGYKYAELANGVAKGNSLAGEAAIGYMKETA 62

Query 64 KSQQEEGSRFPNAQLTERQWESFGQTLLERDLELRQQWMNERRPDLALNLPGKDVMLAHD 123

Q + E + ++ + + + ++ + + + + H

Sbjct 63 SDQG--------RPIAEAEVDAIRRDMAQNYIDSLKFQAKNSGGLVTREISHDEAWEFHK 114

Query 124 RAFERHELDPNCWTPRVLLQAAEQKSGPAKLEQIWTNMLNNDYAGGPRVGNTSVDAI 180

F +H L + WT W + L + + +

Sbjct 115 DVFLKHGLTVDAWTLNTPFLMM----SEDMRAAFWQDTLAAAGDPLAEAKLSYLTMM 167

>WP_084554026.1 calcium-binding protein [Snodgrassella alvi]

ORF24223.1 hypothetical protein BGI07_08335 [Snodgrassella alvi]

ORF33081.1 hypothetical protein BGI11_09585 [Snodgrassella alvi]

ORF34266.1 hypothetical protein BGI10_00690 [Snodgrassella alvi]

ORF37593.1 hypothetical protein BGI13_08060 [Snodgrassella alvi]

ORF40515.1 hypothetical protein BGI14_04725 [Snodgrassella alvi]

ORF43187.1 hypothetical protein BGI15_05160 [Snodgrassella alvi]

Length=1585

Score = 145 bits (365), Expect = 5e-35, Method: Composition-based stats.

Identities = 38/213 (18%), Positives = 74/213 (35%), Gaps = 23/213 (11%)

Query 4 LTERDLSVLGSYARDGNRELYWN-YLSQLPGADGYGTLALGVVRNDSLPGRVANTYAQDY 62

LT L+ + GN Y + Y A+GV DS+ G A + Q

Sbjct 7 LTAEQLNSWNQKIKTGNLSAIGEVYQTLQEKGYNYAAWAIGVATGDSITGNGALEFMQTV 66

Query 63 AKSQQEEGSRFPNAQLTERQWESFGQTLLERDLELRQQWMNERRPDLALNLPGKDVMLAH 122

AK LT+ + +S + + L Q+ + E ++ +++ H

Sbjct 67 AKDH--------KQILTQARIDSVRRDMALGYLAKLQRKLKEGHGG--EDITYEEMYEFH 116

Query 123 DRAFERHELDPNCWTPRVLLQAAEQKS-----------GPAKLEQIWTNMLNNDYAGGPR 171

F ++ LD + WT + + + G +E +W +

Sbjct 117 VNVFNKNGLDISYWTLYTPMSIIQTNASGSGRNGNMIEGSKVVESMWEQIRATKGEVVHG 176

Query 172 VGNTSVDAISQM-GWTKGGQYLTRLSVLEATQA 203

+ S++ M KG Y+ +++ ++Q

Sbjct 177 GASVSLELYQIMQDAKKGYIYVDKITGDVSSQT 209

>WP_050288624.1 serine protease [Yersinia intermedia]

CNI45747.1 putative serine protease [Yersinia intermedia]

Length=1814

Score = 145 bits (365), Expect = 6e-35, Method: Composition-based stats.

Identities = 41/271 (15%), Positives = 89/271 (33%), Gaps = 30/271 (11%)

Query 4 LTERDLSVLGSYAR----DGNRELYWNYLSQLPGADGYGTLALGVVRNDSLPGRVANTYA 59

L DL+ + +++L+ D Y LA GVV+ +S+ GR+A Y

Sbjct 13 LNAADLAYAKQLLEANKESKDPGPMYDFLASK--GDRYAILANGVVKENSIAGRIAINYM 70

Query 60 QDYAKSQQEEGSRFPNAQLTERQWESFGQTLLERDLELRQQWMNERRPDL-ALNLPGKDV 118

+D S + LTE + + + +Q +++ + ++ +

Sbjct 71 RDVGVSHNKH--------LTETDIKHIRYDMALGYFDTQQDRLDKSPTGIIYGDIGHEQA 122

Query 119 MLAHDRAFERHELDPNCWTPRVLLQAAEQKSGPAKLEQIWTNMLNNDYAGGPRVGNTSVD 178

H+ F+ H L P WT + + + W + LN+ +

Sbjct 123 AQFHNSVFKMHGLPPEAWTLYPVYKVLPKNQHGG----HWQSTLNSAGNSADEIL----- 173

Query 179 AISQMGWTKGGQYLTRLSVLEATQALEGRSAV-DPNVIGGNSYY---AMYFEADRKWASI 234

+S L+ + + V D + +G + ++ + +

Sbjct 174 -LSFNTVKIMVDALSTSPASDQPMIHNWLNIVMDADNLGAAAGAIGNQLFSSDEEVAP-V 231

Query 235 SAGGGHMSLREITDPSRIAELNDAREVRLER 265

S + + P + + D + R +

Sbjct 232 STEMCPIDINITPTPQAVQRIADEDQARRDV 262

>WP_100125262.1 hypothetical protein [Snodgrassella alvi]

PIT34659.1 hypothetical protein BHC50_02705 [Snodgrassella alvi]

PIT35852.1 hypothetical protein BHC42_03515 [Snodgrassella alvi]

Length=2377

Score = 144 bits (363), Expect = 1e-34, Method: Composition-based stats.

Identities = 36/198 (18%), Positives = 64/198 (32%), Gaps = 22/198 (11%)

Query 4 LTERDLSVLGSYARDGNRELYWN-YLSQLPGADGYGTLALGVVRNDSLPGRVANTYAQDY 62

LT+ L+ + GN Y Y A+GV DS+ G A Y Q

Sbjct 7 LTKEQLNSWNQKIKTGNLSAIGEVYQILKKKGYDYAAWAIGVATGDSITGNGALEYMQAV 66

Query 63 AKSQQEEGSRFPNAQLTERQWESFGQTLLERDLELRQQWMNERRPDLALNLPGKDVMLAH 122

AK LT+ + +S + + L + Q + + ++ ++ H

Sbjct 67 AKGH--------KQILTQARIDSVRRDMALGYLAMLQNKLEKGHGG--EDITYAEMYEFH 116

Query 123 DRAFERHELDPNCWTPRVLLQAAEQKS-----------GPAKLEQIWTNMLNNDYAGGPR 171

F + LD + WT + + + G +E +W +

Sbjct 117 VNVFNENGLDISYWTLYTPMSIIQTNASGAGRNGNMIKGDKVVESMWEQIRATKGEVAHG 176

Query 172 VGNTSVDAISQMGWTKGG 189

+ S++ M K G

Sbjct 177 GTSVSLELYRIMQDAKKG 194

>WP_042546422.1 serine protease [Yersinia aldovae]

AJJ63209.1 proconvertase P-domain protein [Yersinia aldovae 670-83]

Length=1806

Score = 144 bits (362), Expect = 1e-34, Method: Composition-based stats.

Identities = 42/268 (16%), Positives = 93/268 (35%), Gaps = 31/268 (12%)

Query 4 LTERDLS----VLGSYARDGNRELYWNYLSQLPGADGYGTLALGVVRNDSLPGRVANTYA 59

L DL+ +L ++ + + +++L+ D Y LA VV+ +S+ GR+A Y

Sbjct 13 LNAADLACARQLLETHKENKDPGPMYDFLASK--GDRYAILANSVVKENSIAGRIAINYM 70

Query 60 QDYAKSQQEEGSRFPNAQLTERQWESFGQTLLERDLELRQQWMNER-RPDLALNLPGKDV 118

+D S + LTE + + + +Q +++ + ++ +

Sbjct 71 RDVGVSHNK--------PLTETDIKHIRYDMARGYFDTQQSRLDDSLTGIIYGDIGHEQA 122

Query 119 MLAHDRAFERHELDPNCWTPRVLLQAAEQKSGPAKLEQIWTNMLNNDYAGGPRVGNTSVD 178

H+ F++H L WT + + + W + LN+ +

Sbjct 123 AQFHNSVFKKHGLPAEAWTLYPVYKVLPKNQHGG----HWQSTLNSAGNSADEIL----- 173

Query 179 AISQMGWTKGGQYLTRLSVLEATQALEGRSAV-DPNVIGGNSYY---AMYFEADR-KWAS 233

+S L+ + + V D + +G + ++ + S

Sbjct 174 -LSFNTVKIMVDALSTSPASDQPMIHNWLNIVMDADNLGAAAGAIGNQLFSSDEEVSPVS 232

Query 234 ISAGGGHMSLREITDP-SRIAELNDARE 260

+++ RIA+ + AR

Sbjct 233 TEMCNIDITINPTPQAVQRIADEDQARR 260

>WP_050074755.1 serine protease [Yersinia intermedia]

CNG72078.1 putative serine protease [Yersinia intermedia]

Length=1814

Score = 143 bits (361), Expect = 2e-34, Method: Composition-based stats.

Identities = 43/271 (16%), Positives = 90/271 (33%), Gaps = 30/271 (11%)

Query 4 LTERDLSVLGSYAR----DGNRELYWNYLSQLPGADGYGTLALGVVRNDSLPGRVANTYA 59

L DL+ + +++L+ D Y LA GVV+ +S+ GR+A Y

Sbjct 13 LNAADLAYAKQLLEANKESKDPGPMYDFLASK--GDRYAILANGVVKENSIAGRIAINYM 70

Query 60 QDYAKSQQEEGSRFPNAQLTERQWESFGQTLLERDLELRQQWMNERRPDL-ALNLPGKDV 118

+D S + LTE + + + +Q +++ + ++ +

Sbjct 71 RDVGVSHNKH--------LTETDIKHIRYDMALGYFDTQQDRLDKSPTGIIYGDIGHEQA 122

Query 119 MLAHDRAFERHELDPNCWTPRVLLQAAEQKSGPAKLEQIWTNMLNNDYAGGPRVGNTSVD 178

H+ FE H L P WT + + + W + LN+ +

Sbjct 123 AQFHNSVFEMHGLPPEAWTLYPVYKVLPKNQHGG----HWQSTLNSAGNSADEIL----- 173

Query 179 AISQMGWTKGGQYLTRLSVLEATQALEGRSAV-DPNVIGGNSYY---AMYFEADRKWASI 234

+S L+ S + + V D + +G + ++ + +

Sbjct 174 -LSFNTVKIMVDALSTSSASDQPMIHNWLNIVMDVDNLGAAAGAIGNQLFSSDEEVAP-V 231

Query 235 SAGGGHMSLREITDPSRIAELNDAREVRLER 265

S + + P + + D + R +

Sbjct 232 STEMCPIDINITPTPQAVQRIADEDQARRDV 262

>WP_049596152.1 serine protease [Yersinia aldovae]

CNL27137.1 putative serine protease [Yersinia aldovae]

Length=1807

Score = 143 bits (361), Expect = 2e-34, Method: Composition-based stats.

Identities = 44/271 (16%), Positives = 95/271 (35%), Gaps = 30/271 (11%)

Query 4 LTERDLS----VLGSYARDGNRELYWNYLSQLPGADGYGTLALGVVRNDSLPGRVANTYA 59

L DL+ +L ++ + + +++L+ D Y LA GVV+ +S+ GR+A Y

Sbjct 13 LNAADLACARQLLETHKENKDPGPMYDFLASK--GDRYAILANGVVKENSIAGRIAINYM 70

Query 60 QDYAKSQQEEGSRFPNAQLTERQWESFGQTLLERDLELRQQWMNERRPDL-ALNLPGKDV 118

+D S + LTE + + + +Q +++ + ++ +

Sbjct 71 RDVGVSHNKH--------LTETDIKHIRYDMALGYFDTQQDRLDKSPTGIIYGDIGHEQA 122

Query 119 MLAHDRAFERHELDPNCWTPRVLLQAAEQKSGPAKLEQIWTNMLNNDYAGGPRVGNTSVD 178

H+ FE H L P WT + + + W + LN+ +

Sbjct 123 AQFHNSVFEMHGLPPEAWTLYPVYKVLPKNQHGG----HWQSTLNSAGNSADEIL----- 173

Query 179 AISQMGWTKGGQYLTRLSVLEATQALEGRSAV-DPNVIGGNSYY---AMYFEADRKWASI 234

+S L+ S + + V D + +G + ++ + +

Sbjct 174 -LSFNTVKIMVDALSTSSASDQPMIHNWLNIVMDVDNLGAAAGAIGNQLFSSDEEVAP-V 231

Query 235 SAGGGHMSLREITDPSRIAELNDAREVRLER 265

S + + P + + D + R +

Sbjct 232 STEMCPIDINITPTPQAVQRIADEDQARRDV 262

>WP_081951580.1 hypothetical protein [Pseudomonas cremoricolorata]

Length=1820

Score = 143 bits (361), Expect = 2e-34, Method: Composition-based stats.

Identities = 51/281 (18%), Positives = 93/281 (33%), Gaps = 24/281 (9%)

Query 1 MSGLTERDLSVLGSYARDGNRELYWNYLSQLPGADGYGTLALGVVRNDSLPGRVANTYAQ 60

M LT D+ + ++++L Y LA GV + D G A +

Sbjct 1 MMKLTPEDIIHAQKLLSEQGPSSFYDFLELK--GSRYAKLANGVAKEDGYAGNAAVGFMN 58

Query 61 DYAKSQQEEGSRFPNAQLTERQWESFGQTLLERDLELRQQWMNERRPDLALNLPGKDVML 120

+ E + + + + E L ++ +NE L ++ +

Sbjct 59 RVYAEKHGE-------PMPGFKRDEVKLLMAESALVAIRKNVNEG--LLNGDVSLDQAID 109

Query 121 AHDRAFERHELDPNCWTPRVLLQAAEQKSGPAKLEQIWTNMLNNDYAGGPRVGNTSVDAI 180

H+RAF + L P WT +L+ + A E + A+

Sbjct 110 FHNRAFTQAGLAPEYWTLDAVLKVIPKSEHQAYWESTLKQAGDFTGEMIIAGSTFKEMAM 169

Query 181 --SQMGWTKGGQYLTRLSVLEATQALEGRSAVDPNVIGGNSYYAM-----YFEADRKWAS 233

S + ++ R++ E AL+G VIG ++ + YF + +

Sbjct 170 HASTAPDGEVSGWMGRMASPEVASALQGPLLKHAQVIGKDAAEQLIGLEPYFRKEEPMIN 229

Query 234 I-SAGGGHMSLREITDPSRI-----AELNDAREVRLERLEK 268

I + S RE T+ A ++RLE +

Sbjct 230 IQTDLLPKDSTREETEAVDSVRNGNAVFEPTHQIRLENGQL 270

>CTQ34811.1 Cyclolysin [Jannaschia rubra]

SFG67579.1 Ca2+-binding protein, RTX toxin-related [Jannaschia rubra]

Length=1734

Score = 143 bits (360), Expect = 2e-34, Method: Composition-based stats.

Identities = 55/255 (22%), Positives = 85/255 (33%), Gaps = 35/255 (14%)

Query 1 MSGLTERDLSVLGSYARDGNRELYWNYLSQLPGADGYGTLALGVVRNDSLPGRVANTYAQ 60

M+ +T+ LS L +A NR Y+ YL + YG LAL VV DS GR+AN +

Sbjct 1 MANITQNQLSTLSGFAASANRTGYYQYLDSIGVGYEYGQLALDVVTGDSRSGRLANLFLA 60

Query 61 DYAKSQQEEGSRFPNAQLTERQWESFGQTLLERDLELRQQWMNERRPDLALNLPGKDVML 120

+ A+++ L + L+ DL RQ A L +

Sbjct 61 ETAQARY-------GVTLQPSDAQQISVALMRADLAARQAAFQGGD---AGALDYAAIRD 110

Query 121 AHDRAFERHELDPNCWTPRVLLQAAEQK-------------SGPAKLEQIWTNMLNNDYA 167

H + F L + WT V L+ A Q SG A +W +L+

Sbjct 111 YHGQVFTSFGLGVDAWTATVPLRLAAQYPQLLVNPAGGVFTSGAAAEAHMWNLLLDAGGV 170

Query 168 GGPRVGNTSVDAISQMGWTKGGQYLTRLSVLEATQALEGRSAVDPNVIGGNSYYAMYFEA 227

+ A T ++ ++ DP I S Y

Sbjct 171 T--FGLKNAPQAFLPNAQTAIHDWVAFVANATG----------DPTAIAWASEVTGYTID 218

Query 228 DRKWASISAGGGHMS 242

+ + + G +

Sbjct 219 ELAYLMGNIGSPYPP 233

>MBT83646.1 hypothetical protein [Sutterellaceae bacterium]

Length=1158

Score = 143 bits (360), Expect = 3e-34, Method: Composition-based stats.

Identities = 47/254 (19%), Positives = 83/254 (33%), Gaps = 18/254 (7%)

Query 4 LTERDLSVLGSYARDGN-RELYWNYLSQLPGADGYGTLALGVVRNDSLPGRVANTYAQDY 62

L+ L+ + G E+ Y GY A GV +++ G A Y

Sbjct 7 LSSAQLNSYLTRINTGGVPEVIKVYQELQDKGYGYAGWAKGVATAETVTGIAAVDYLTGT 66

Query 63 AKSQQEEGSRFPNAQLTERQWESFGQTLLERDLELRQQWMN--ERRPDLALNLPGKDVML 120

A L + Q ++ + + + N ++ ++ +D

Sbjct 67 AL---IGLGGQECRNLGQVQVDAIRVGMADGYVRALLTKANQANNNGFVSSDVNFRDTQA 123

Query 121 AHDRAFERHELDPNCWTPRVLLQAAEQKSGPAKLEQIWTNMLNNDYAGGPRVGNTSVDAI 180

H FE++ L N WT ++ ++ Q G A +E W + + G + +

Sbjct 124 FHRDVFEQNGLTNNNWTLQIPMELIRQTEGDAAVEAYWIRLRDTGGDGPDAI-------L 176

Query 181 SQMGWTKGGQYLTR-LSVLEATQALEGRSAVDPNVIGGNSYYAMYFEADRKWASISAGGG 239

M L A QA E S V P G ++ A+ AD + +

Sbjct 177 ESMWLYTKIGKLANSNDPAIAAQANEWISLV-P---GTANFDALIRAADDLYTVLVGENP 232

Query 240 HMSLREITDPSRIA 253

+ L P +A

Sbjct 233 WLYLHPTIGPILLA 246

>WP_122741956.1 calcium-binding protein [Pseudomonas sp. 286]

Length=1397

Score = 143 bits (360), Expect = 3e-34, Method: Composition-based stats.

Identities = 52/303 (17%), Positives = 94/303 (31%), Gaps = 52/303 (17%)

Query 4 LTERDLSVLGSYARDGNRELYWNYLSQLPGADGYGTLALGVVRNDSLPGRVANTYAQDYA 63

+ L V + + YL++ Y LA GV +SL G A +Y + A

Sbjct 5 IARDQLDVARTVLAGQGVSQMYGYLAEN--GFNYARLADGVALGNSLSGEAALSYMRSVA 62

Query 64 KSQQEEGSRFPNAQLTERQWESFGQTLLERDLELRQQWMNERRPDLALNLPGKDVMLAHD 123

KS+ + LTE + L++ + + + ++V+ H

Sbjct 63 KSRGKT--------LTESDVNKIRLDMARGYLDVLDSIASGNDGSVDREINSEEVLQFHT 114

Query 124 RAFERHELDPNCWTPRVLLQAAEQKSGPAKLEQIWTNMLNNDY---AGGPRVGNTSVDAI 180

+ FE + L WT + + S ++E W +L + T

Sbjct 115 QVFEANGLPIQAWTLDSIFKIINDSS---EIEAYWDMVLESAGHADKELELAFETMGLMY 171

Query 181 SQMG----WTKGGQYLTRLSVLEA-----TQALEGRSAVDPNVIGGNSYYAMYFEADRKW 231

+ +L + ++ + R ++D +V YF

Sbjct 172 HSYDTGNSVLESSTWLGHMISFDSVWSTLSHVFAPRESIDFDV-------YAYF------ 218

Query 232 ASISAGGGHMSLREITDPSRIAELNDAREVRLERLEKRTQF-HPDDPYRTITRSPLTAAV 290

S R++ DP + D V + F H D RT S +

Sbjct 219 ---------TSARQVIDPLVLD--LDGDGVETVSADVGIMFDHDGDGIRTG--SGWVLSD 265

Query 291 DDV 293

D +

Sbjct 266 DGL 268

>SEM26499.1 Regulatory P domain of the subtilisin-like proprotein convertase

[Pseudomonas sp. NFACC41-3]

Length=2190

Score = 142 bits (358), Expect = 6e-34, Method: Composition-based stats.

Identities = 42/310 (14%), Positives = 92/310 (30%), Gaps = 37/310 (12%)

Query 9 LSVLGSYARDGNRELYWNYLSQLPGADGYGTLALGVVRNDSLPGRVANTYAQDYAKSQQE 68

+ + ++YL+ Y LA GV + +S+ G VA + + A

Sbjct 1 MDYAKALLSSAGPGAMYDYLAV--RGYKYAVLANGVAKGNSIAGEVAINFMKTTAAD--- 55

Query 69 EGSRFPNAQLTERQWESFGQTLLERDLELRQQWMNERRPDLALNLPGKDVMLAHDRAFER 128

++E + + + ++ + ++ ++ ++ H + F+

Sbjct 56 -----AGHVMSEDDVNRIRRQMASEYVSTLSDKLDA-KGVVSADITYEEAWGFHRKVFDN 109

Query 129 HELDPNCWTPRVLLQAAEQKSGPAKLEQIWTNMLNNDYAGGPRVGNTSVDAISQMGWTKG 188

+ L + WT + + G W N+L+ G+ + K

Sbjct 110 NGLSVDAWTLNSVFEVL----GGNSRAVYWGNVLD-------FAGSPGWELALSYETDKL 158

Query 189 GQYLTRLSVLEATQA-LEGRSAVDP----NVIGGNSYYAMYFEADRKWAS--ISAGGGHM 241

R+ E Q + + VD + + +Y KW I M

Sbjct 159 MALAARIGTAETKQIAMNWINRVDSPSGDWTVVTSLTSQVY-----KWVRGLIVDEAPDM 213

Query 242 SLREITDPSRIAELNDAREVRLE---RLEKRTQFHPDDPYRTITRSPLTAAVDDVADPSQ 298

S+ + + + ++ E + + F + +I D

Sbjct 214 SVPNLINIDINPSPQPQQHIQGESKAQQDVANGFIQNSATNSIFAVGGILNKTDFTSTQM 273

Query 299 APTRLADIGP 308

A I P

Sbjct 274 ASLASGGIRP 283

>WP_120266707.1 serine protease [Pseudomonas sp. TMW 2.1634]

Length=1890

Score = 141 bits (356), Expect = 9e-34, Method: Composition-based stats.

Identities = 33/206 (16%), Positives = 59/206 (29%), Gaps = 15/206 (7%)

Query 4 LTERDLSVLGSYARDGNRELYWNYLSQLPGADGYGTLALGVVRNDSLPGRVANTYAQDYA 63

L+ DL ++YLS Y LA GV + SL G A + + A

Sbjct 5 LSLHDLERAKGLLETVGPAAAYDYLSAK--GYRYAVLANGVAKGGSLSGDAAINFMRLTA 62

Query 64 KSQQEEGSRFPNAQLTERQWESFGQTLLERDLELRQQWMNERRPDLALNLPGKDVMLAHD 123

L++ + + ++ + ++ R L ++ + H

Sbjct 63 SD--------SGRTLSDVDVNRIRKDMAIAYIDALKDKLDFRGGALIEDVNYSEAWEFHR 114

Query 124 RAFERHELDPNCWTPRVLLQAAEQKSGPAKLEQIWTNMLNNDYAGGPRVGNTSVDAISQM 183

R F H L WT +L E W +L + P N ++ M

Sbjct 115 RVFNDHGLTVEAWTLHSVLSVI----TENTREAYWKKVLESAG-DLPGELNLAIQTGQLM 169

Query 184 GWTKGGQYLTRLSVLEATQALEGRSA 209

+ + +

Sbjct 170 ALASAISTPANRELAKRWVGRVDSPS 195

>WP_095003778.1 serine protease [Pseudomonas fragi]

PAA32098.1 serine protease [Pseudomonas fragi]

Length=1890

Score = 141 bits (356), Expect = 9e-34, Method: Composition-based stats.

Identities = 33/206 (16%), Positives = 59/206 (29%), Gaps = 15/206 (7%)

Query 4 LTERDLSVLGSYARDGNRELYWNYLSQLPGADGYGTLALGVVRNDSLPGRVANTYAQDYA 63

L+ DL ++YLS Y LA GV + SL G A + + A

Sbjct 5 LSLHDLERAKGLLETVGPAAAYDYLSAK--GYRYAVLANGVAKGGSLSGDAAINFMRLTA 62

Query 64 KSQQEEGSRFPNAQLTERQWESFGQTLLERDLELRQQWMNERRPDLALNLPGKDVMLAHD 123

L++ + + ++ + ++ R L ++ + H

Sbjct 63 SD--------SGRTLSDVDVNRIRKDMAIAYIDALKDKLDFRGGALIEDVNYSEAWEFHR 114

Query 124 RAFERHELDPNCWTPRVLLQAAEQKSGPAKLEQIWTNMLNNDYAGGPRVGNTSVDAISQM 183

R F H L WT +L E W +L + P N ++ M

Sbjct 115 RVFNDHGLTVEAWTLHSVLSVI----TENTREAYWKKVLESAG-DLPGELNLAIQTGQLM 169

Query 184 GWTKGGQYLTRLSVLEATQALEGRSA 209

+ + +

Sbjct 170 ALASAISTPANRELAKRWVGRVDSPS 195

>WP_011617781.1 tandem-95 repeat protein [Cupriavidus necator]

CAJ97095.1 RTX toxin exported protein [Cupriavidus necator H16]

Length=2426

Score = 141 bits (356), Expect = 1e-33, Method: Composition-based stats.

Identities = 35/171 (20%), Positives = 61/171 (36%), Gaps = 9/171 (5%)

Query 1 MSG-LTERDLSVLGSYARDGNRELYWNYLSQLPGADGYGTLALGVVRNDSLPGRVANTYA 59

M ++ R L + G Y Y A GV S+ G A TY

Sbjct 1 MPNPISARSLKDIRFQLNAGTLTPSQVYSRLEGYGYKYSGWAGGVADATSIAGASALTYM 60

Query 60 QDYAKSQQEEGSRFPNAQLTERQWESFGQTLLERDLELRQQWMNERRPDLALNLPGKDVM 119

+ AK LT Q E + + L+ Q + + ++ ++V

Sbjct 61 HNTAKDLG--------RPLTGAQVEKIKLDMAKGYLDALYQQTIDGSLPVTRDIDSREVW 112

Query 120 LAHDRAFERHELDPNCWTPRVLLQAAEQKSGPAKLEQIWTNMLNNDYAGGP 170

H + F+ + L P+ WT + E+ GPA++E+ W+ + +

Sbjct 113 NFHRQVFKDNGLPPSAWTLDTPFRLMEKMGGPAQVERFWSMLRDTGGGYSD 163

>THG74799.1 hypothetical protein E5198_18800, partial [Pseudomonas sp. A-1]

Length=802

Score = 141 bits (355), Expect = 1e-33, Method: Composition-based stats.

Identities = 45/233 (19%), Positives = 79/233 (34%), Gaps = 24/233 (10%)

Query 5 TERDLSVLGSYARDGNRELYWN-YLSQLPGADGYGTLALGVVRNDSLPGRVANTYAQDYA 63

T L+ L + G Y + Y A GV D++ G+ A +Y Q A

Sbjct 6 TSTQLNNLQQSVQSGGVGAAAQAYGNLYAQGYNYAGWAGGVATGDTISGQAALSYLQGTA 65

Query 64 KSQQEEGSRFPNAQLTERQWESFGQTLLERDLELRQQWMNERRPDLALNLPGKDVMLAHD 123

LT++Q + + + LE ++ E L +L ++ HD

Sbjct 66 MM---GMGGDQCRNLTQQQIDKIRTDMANQTLEKYKEIARENGGILDRDLTYQETKDIHD 122

Query 124 RAFERHELDPNCWTPRVLLQAAEQKSGPAKLEQIWTNMLNNDYAGGPRVGNTSVDAISQM 183

+ F + L + WT ++ +K G +E +W + + G ++

Sbjct 123 KVFRENSLSLDNWTLNTPMELIREKYGDQAVENLWKQIRDTGGDGADAAMINTI------ 176

Query 184 GWTKGGQYLTRLSVLEATQALEGRSAVDPNVIGGNSYYAMYFEADRKWASISA 236

LT + + +Q E R D S+ + E W IS

Sbjct 177 -------LLTFMKGAQDSQNPETRQKAD-------SWLDQFDELSEWWDVISD 215

>MAG80794.1 hypothetical protein [Sutterellaceae bacterium]

Length=1158

Score = 141 bits (354), Expect = 2e-33, Method: Composition-based stats.

Identities = 47/254 (19%), Positives = 82/254 (32%), Gaps = 18/254 (7%)

Query 4 LTERDLSVLGSYARDGN-RELYWNYLSQLPGADGYGTLALGVVRNDSLPGRVANTYAQDY 62

L+ L+ + G E+ Y GY A GV +++ G A Y

Sbjct 7 LSSAQLNSYLTRINTGGVPEVIKVYQELQDKGYGYAGWAKGVATAETVTGIAAVDYLTGT 66

Query 63 AKSQQEEGSRFPNAQLTERQWESFGQTLLERDLELRQQWMN--ERRPDLALNLPGKDVML 120

A L + Q ++ + + + N ++ + +D

Sbjct 67 AL---IGLGGQECRNLGQVQVDAIRVGMADGYVRALLTKANQXNNNGXVSSXVNFRDTQA 123

Query 121 AHDRAFERHELDPNCWTPRVLLQAAEQKSGPAKLEQIWTNMLNNDYAGGPRVGNTSVDAI 180

H FE++ L N WT ++ ++ Q G A +E W + + G + +

Sbjct 124 FHRDVFEQNGLTNNNWTLQIPMELIRQTEGDAAVEAYWIRLRDTGGDGPDAI-------L 176

Query 181 SQMGWTKGGQYLTR-LSVLEATQALEGRSAVDPNVIGGNSYYAMYFEADRKWASISAGGG 239

M L A QA E S V P G ++ A+ AD + +

Sbjct 177 ESMWLYTKIGKLANSNDPAIAAQANEWISLV-P---GTANFDALIRAADDLYTVLVGENP 232

Query 240 HMSLREITDPSRIA 253

+ L P +A

Sbjct 233 WLYLHPTIGPILLA 246

>WP_038044393.1 calcium-binding protein [Thioalkalivibrio sp. ALJ3]

Length=1514

Score = 140 bits (353), Expect = 2e-33, Method: Composition-based stats.

Identities = 40/224 (18%), Positives = 78/224 (35%), Gaps = 24/224 (11%)

Query 2 SGLTERDLSVLGSYARDGNRELYWNYLSQLPGADGYGTLALGVVRNDSLPGRVANTYAQD 61

+ LT+ DL S +G ++YL + D Y LA GVV D++ G+VA Y Q

Sbjct 8 TPLTQSDLDYARSLQAEGRLTDMYDYL--VSFGDRYSMLAKGVVEGDTMSGQVALRYMQA 65

Query 62 YAKSQQEEGSRFPNAQLTERQWESFGQTLLERDLELRQQWMNERRP-DLALNLPGKDVML 120

A+ + + + L+ Q+ +++ + + +

Sbjct 66 AAE--------EEGVPHEDADVDRIRNDMAIGYLDALQRIIDDSDQAVVDREITAVEARD 117

Query 121 AHDRAFERHELDPNCWTPRVLLQAAEQKSGPAKLEQIWTNMLNNDYAGGPRVGNTSVDAI 180

H AFE+++L + WT + + G + W L+ R + + +

Sbjct 118 FHRHAFEQNDLGIDAWTLYIPFELM----GDEGAQAYWEGTLDAAG-DSAREFALAKETL 172

Query 181 SQMGWTKGGQYLTRLSVLEATQALEGRSAV--------DPNVIG 216

+M + + ++ D +VIG

Sbjct 173 GKMVSMHQALEFAKGFATNREELDAATESIETWSGRLADLDVIG 216

>PYE75958.1 hemolysin type calcium-binding protein [Xylophilus ampelinus]

Length=1210

Score = 140 bits (353), Expect = 3e-33, Method: Composition-based stats.

Identities = 44/249 (18%), Positives = 80/249 (32%), Gaps = 11/249 (4%)

Query 1 MSGLTERDLSVLGSYARDGNRELYWN-YLSQLPGADGYGTLALGVVRNDSLPGRVANTYA 59

M+ LT L + G + Y Y ALGV +S+ G+ A Y

Sbjct 1 MNNLTVSQLISYRGAIKSGGMDAARTVYADLNSQGYHYAGWALGVATGNSITGQSALDYL 60

Query 60 QDYAKSQQEEGSRFPNAQLTERQWESFGQTLLERDLELRQQWMNERRPDLALNLPGKDVM 119

A LT Q ++ + + L+ ++ K+

Sbjct 61 SGTAM---IGLGGDACRNLTPAQVDNIRMDMALGYINKLIGIAKNENGILSRDVKFKETA 117

Query 120 LAHDRAFERHELDPNCWTPRVLLQAAEQKSGPAKLEQIWTNMLNNDYAGGPRVGNTSVDA 179

H AFE++ L + WT V ++ ++ G +E W + ++ + G ++

Sbjct 118 AFHKEAFEKNHLTLDNWTLNVPMEIIRRQRGDQAVEDAWARIRDSGGS-----GIDALMV 172

Query 180 ISQMGWTKGGQYLTRLSVLEATQALEGRSAVDPNVIGGNSYYAMYFEADRKWASISAGGG 239

+ + T G ++ + L A + V P V W+ G

Sbjct 173 SAGLANTVGHASVSPDAALR-KMAQDWIDQV-PGVANWAQMGRFANAVSNAWSGTLGDWG 230

Query 240 HMSLREITD 248

R + D

Sbjct 231 RTMARTVND 239

>WP_018868108.1 MULTISPECIES: calcium-binding protein [Thioalkalivibrio]

Length=1506

Score = 140 bits (352), Expect = 3e-33, Method: Composition-based stats.

Identities = 44/270 (16%), Positives = 89/270 (33%), Gaps = 35/270 (13%)

Query 2 SGLTERDLSVLGSYARDGNRELYWNYLSQLPGADGYGTLALGVVRNDSLPGRVANTYAQD 61

+ +T+ DL S A G +++L+ D Y LA GVV D+L G A + ++

Sbjct 8 TPITQGDLDHARSLADQGRLADMYDHLAS--FGDRYSILAKGVVEGDTLSGVAAIEFMKN 65

Query 62 YAKSQQEEGSRFPNAQLTERQWESFGQTLLERDLELRQQWMNE---RRPDLALNLPGKDV 118

A+++ LTE ++ + + L+ Q ++ + + ++

Sbjct 66 TAEAKG--------IDLTEADIDAIREDMATGYLDTLQGILDNPDNATGTINREIDAREA 117

Query 119 MLAHDRAFERHELDPNCWTPRVLLQAAEQKSGPAKLEQIWTNMLNNDYAGGPRVGNTSV- 177

HDR F L + WT + G ++ W L+ G

Sbjct 118 QRFHDRVFVDAGLGEDAWTLNTPYEI----FGEQAAQEHWEKTLDAAGNLGKEFVLAGGL 173

Query 178 -------------DAISQMGWTKGGQYLTRLSVLEATQALEGRSAVDPNVIGGNSYYAMY 224

+ QM ++ R + + A + D + + M

Sbjct 174 HQWMAFTSSFVEDEQKEQMRSWS-SRFDNRGAAWKLALAFGEQGITDSRDWLTDFFEEMG 232

Query 225 FEA---DRKWASISAGGGHMSLREITDPSR 251

+ + W + G +R+ + +

Sbjct 233 GPSGALESPWNYWTGGEFIDDIRDFFNRGQ 262

>WP_025330317.1 hypothetical protein [Snodgrassella alvi]

ORF03560.1 hypothetical protein BGH96_06005 [Snodgrassella alvi]

PIT46434.1 hypothetical protein BHC45_00975 [Snodgrassella alvi]

Length=1608

Score = 140 bits (352), Expect = 3e-33, Method: Composition-based stats.

Identities = 31/182 (17%), Positives = 63/182 (35%), Gaps = 22/182 (12%)

Query 4 LTERDLSVLGSYARDGNRELYWN-YLSQLPGADGYGTLALGVVRNDSLPGRVANTYAQDY 62

LT++++ + G Y + Y A GV D+L G A + Q

Sbjct 20 LTKKEIDTWSEQIKTGELSAIGEVYQALAKKGYDYAQWAFGVATADTLTGNGALQFMQAV 79

Query 63 AKSQQEEGSRFPNAQLTERQWESFGQTLLERDLELRQQWMNERRPDLALNLPGKDVMLAH 122

AK +L + + +S + + L + Q+ +NE + ++ +++ H

Sbjct 80 AKDH--------KQKLPQARVDSVRRDMALGYLAMLQKKLNEGKGG--EDITYDEMLDFH 129

Query 123 DRAFERHELDPNCWTPRVLLQAAEQK-----------SGPAKLEQIWTNMLNNDYAGGPR 171

+ F+ ++LD WT + + G +E +W +M

Sbjct 130 AKVFKDNKLDIGYWTLYTPMSIIQNHASATGSDGQVIGGKQVVENMWQHMRATKGTKLKG 189

Query 172 VG 173

Sbjct 190 SW 191

>WP_060484107.1 peptidase S8 [Pseudomonas sp. NBRC 111123]

Length=1785

Score = 140 bits (352), Expect = 3e-33, Method: Composition-based stats.

Identities = 39/212 (18%), Positives = 65/212 (31%), Gaps = 22/212 (10%)

Query 1 MSGLTERDLSVLGSYARDGNRELYWNYLSQLPGADGYGTLALGVVRNDSLPGRVANTYAQ 60

MSGL D+ ++YL+ D Y LA GV + +S+ G VA + +

Sbjct 1 MSGLLMEDVRRARELYESQGVGEMYDYLASK--GDRYAVLANGVAKGNSVAGVVAVDFMK 58

Query 61 DYAKSQQEEGSRFPNAQLTERQWESFGQTLLERDLELRQQWMNERRPDLALNLPGKDVML 120

R + + E + E L+L + + E + + K+V

Sbjct 59 --------RTEREAGRPMQDADVEMVRLRMAEAYLDLLDKKVKEGTIGV--EINHKEVWS 108

Query 121 AHDRAFERHELDPNCWTPRVLLQAAEQKSGPAKLEQIWTNMLNNDYAGGPRVGNTSVDAI 180

H FE E + WT + P E W +L+ +

Sbjct 109 FHAEVFEGLERSRDAWTLNTVFDLKP----PKARESYWKMVLDAAGNPVDEGL------L 158

Query 181 SQMGWTKGGQYLTRLSVLEATQALEGRSAVDP 212

+ + QA S +D

Sbjct 159 AVRTDMQVSFSTAMAPAHLRAQARSWLSRIDS 190

>AHN28085.1 Alkaline phosphatase [Snodgrassella alvi wkB2]

Length=1595

Score = 140 bits (352), Expect = 4e-33, Method: Composition-based stats.

Identities = 31/182 (17%), Positives = 63/182 (35%), Gaps = 22/182 (12%)

Query 4 LTERDLSVLGSYARDGNRELYWN-YLSQLPGADGYGTLALGVVRNDSLPGRVANTYAQDY 62

LT++++ + G Y + Y A GV D+L G A + Q

Sbjct 7 LTKKEIDTWSEQIKTGELSAIGEVYQALAKKGYDYAQWAFGVATADTLTGNGALQFMQAV 66

Query 63 AKSQQEEGSRFPNAQLTERQWESFGQTLLERDLELRQQWMNERRPDLALNLPGKDVMLAH 122

AK +L + + +S + + L + Q+ +NE + ++ +++ H

Sbjct 67 AKDH--------KQKLPQARVDSVRRDMALGYLAMLQKKLNEGKGG--EDITYDEMLDFH 116

Query 123 DRAFERHELDPNCWTPRVLLQAAEQK-----------SGPAKLEQIWTNMLNNDYAGGPR 171

+ F+ ++LD WT + + G +E +W +M

Sbjct 117 AKVFKDNKLDIGYWTLYTPMSIIQNHASATGSDGQVIGGKQVVENMWQHMRATKGTKLKG 176

Query 172 VG 173

Sbjct 177 SW 178

>WP_100156361.1 calcium-binding protein [Snodgrassella alvi]

PIT41053.1 hypothetical protein BHC43_02050 [Snodgrassella alvi]

Length=1613

Score = 139 bits (351), Expect = 4e-33, Method: Composition-based stats.

Identities = 33/192 (17%), Positives = 69/192 (36%), Gaps = 22/192 (11%)

Query 4 LTERDLSVLGSYARDGNRELYWN-YLSQLPGADGYGTLALGVVRNDSLPGRVANTYAQDY 62

LT+ L + GN Y Y A+GV DS+ G A + Q

Sbjct 7 LTKEQLKTWNQKIKTGNLSTIGEIYQMLREKGYDYAGWAIGVATGDSITGNGALEFMQAV 66

Query 63 AKSQQEEGSRFPNAQLTERQWESFGQTLLERDLELRQQWMNERRPDLALNLPGKDVMLAH 122

A+ + +T+ + +S + + L++ + +++ + ++ +++ H

Sbjct 67 ARDK--------KQVITQARIDSVRRDMALGYLDILLKKLDDGKEGA--DIGYEEMFNFH 116

Query 123 DRAFERHELDPNCWTPRVLLQAAEQK-----------SGPAKLEQIWTNMLNNDYAGGPR 171

+ FE++ LD N WT + + G +E +W N+

Sbjct 117 NVVFEKNNLDINYWTLYAPMSIIQNNASITRTDGSIIQGAQVVENMWENIRKTKGEVVSG 176

Query 172 VGNTSVDAISQM 183

S++ + M

Sbjct 177 GSIVSLELYNIM 188

>WP_100149956.1 hypothetical protein [Snodgrassella alvi]

PIT52820.1 hypothetical protein BHC48_01775 [Snodgrassella alvi]

Length=1609

Score = 139 bits (351), Expect = 5e-33, Method: Composition-based stats.

Identities = 31/186 (17%), Positives = 65/186 (35%), Gaps = 22/186 (12%)

Query 4 LTERDLSVLGSYARDGNRELYWN-YLSQLPGADGYGTLALGVVRNDSLPGRVANTYAQDY 62

LT++++ + G Y + Y ALGV D++ G A + Q

Sbjct 7 LTKKEIDAWSQQIKSGKLSAIGEVYQALAQKGYDYAHWALGVATADTITGNGALQFMQAV 66

Query 63 AKSQQEEGSRFPNAQLTERQWESFGQTLLERDLELRQQWMNERRPDLALNLPGKDVMLAH 122

AK +L + + +S + + L + Q + + + ++ K+++ H

Sbjct 67 AKDH--------KQKLPQARVDSVRRDMALGYLAMLQDKLAKGKGG--EDITYKEMLEFH 116

Query 123 DRAFERHELDPNCWTPRVLLQAAEQK-----------SGPAKLEQIWTNMLNNDYAGGPR 171

+ F ++LD WT + + G +E +W +M G

Sbjct 117 VKVFNDNKLDIGYWTLYTPMSIIQNHASATGSNGQVIGGKQVVENMWQHMRATQGTGLTG 176

Query 172 VGNTSV 177

+ +

Sbjct 177 SWLSLM 182

>PCL21424.1 hypothetical protein CPT77_02030, partial [Snodgrassella alvi]

Length=2029

Score = 139 bits (350), Expect = 7e-33, Method: Composition-based stats.

Identities = 32/176 (18%), Positives = 66/176 (38%), Gaps = 22/176 (13%)

Query 4 LTERDLSVLGSYARDGNRELYWN-YLSQLPGADGYGTLALGVVRNDSLPGRVANTYAQDY 62

LT+ L+ + GN Y + Y A+GV DS+ G A + Q

Sbjct 7 LTKEQLNSWYQKIKTGNLSAIGEVYQTLQEKGYDYAAWAIGVATGDSITGNGALEFMQTV 66

Query 63 AKSQQEEGSRFPNAQLTERQWESFGQTLLERDLELRQQWMNERRPDLALNLPGKDVMLAH 122

AK LT+ + +S + + L + Q+ +N+ + ++ +++ + H

Sbjct 67 AKDH--------KQILTQARIDSVRRDMALGYLAMLQKKLNDGQGG--EDITYQEMFIFH 116

Query 123 DRAFERHELDPNCWTPRVLLQAAEQK-----------SGPAKLEQIWTNMLNNDYA 167

+ F+++ LD + WT + + G +E +W ++

Sbjct 117 EEVFKKNNLDLSYWTLYTPMLIIQNNASVSNNNGYTIDGKQIVENMWQDIRATKGE 172

>WP_100152328.1 calcium-binding protein [Snodgrassella alvi]

PIT38355.1 hypothetical protein BHC54_07340 [Snodgrassella alvi]

Length=1613

Score = 139 bits (349), Expect = 7e-33, Method: Composition-based stats.

Identities = 33/192 (17%), Positives = 69/192 (36%), Gaps = 22/192 (11%)

Query 4 LTERDLSVLGSYARDGNRELYWN-YLSQLPGADGYGTLALGVVRNDSLPGRVANTYAQDY 62

LT+ L + GN Y Y A+GV DS+ G A + Q

Sbjct 7 LTKEQLKTWNQKIKTGNLSTIGEIYQMLREKGYDYAGWAIGVATGDSITGNGALEFMQAV 66

Query 63 AKSQQEEGSRFPNAQLTERQWESFGQTLLERDLELRQQWMNERRPDLALNLPGKDVMLAH 122

A+ + +T+ + +S + + L++ + +++ + ++ +++ H

Sbjct 67 ARDK--------KQVITQARIDSVRRDMALGYLDILLKKLDDGKEGA--DIGYEEMFNFH 116

Query 123 DRAFERHELDPNCWTPRVLLQAAEQK-----------SGPAKLEQIWTNMLNNDYAGGPR 171

+ FE++ LD N WT + + G +E +W N+

Sbjct 117 NVVFEKNNLDINYWTLYAPMSIIQNNASITRTDGSIIQGAQVVENMWENIRKTKGEVVSG 176

Query 172 VGNTSVDAISQM 183

S++ + M

Sbjct 177 GSIVSLELYNIM 188

>WP_100153513.1 hypothetical protein [Snodgrassella alvi]

PIT47347.1 hypothetical protein BHC51_05310 [Snodgrassella alvi]

Length=1609

Score = 139 bits (349), Expect = 7e-33, Method: Composition-based stats.

Identities = 31/182 (17%), Positives = 60/182 (33%), Gaps = 22/182 (12%)

Query 4 LTERDLSVLGSYARDGNRELYWN-YLSQLPGADGYGTLALGVVRNDSLPGRVANTYAQDY 62

LT+ L + G Y + Y A GV D++ G A + Q

Sbjct 20 LTKEQLKAWSEQIKTGELSAIGEVYQALAKKGYDYAHWAFGVATADTITGNGALQFMQAV 79

Query 63 AKSQQEEGSRFPNAQLTERQWESFGQTLLERDLELRQQWMNERRPDLALNLPGKDVMLAH 122

AK +LT+ + +S + + L + Q + + ++ +++ H

Sbjct 80 AKDH--------KQKLTQARVDSVRRDMALGYLAMLQDKLENGKGG--EDITYYEMLEFH 129

Query 123 DRAFERHELDPNCWTPRVLLQAAEQK-----------SGPAKLEQIWTNMLNNDYAGGPR 171

+ F+ ++LD WT + + G +E +W +M G

Sbjct 130 IKVFKDNKLDIGYWTLYTPMSIIQNHASATGSNGQVIGGKQVVENMWQHMRATQGTGVTG 189

Query 172 VG 173

Sbjct 190 SW 191

>WP_100120845.1 hypothetical protein [Snodgrassella alvi]

PIT09585.1 hypothetical protein BGI31_04590 [Snodgrassella alvi]

Length=1609

Score = 139 bits (349), Expect = 8e-33, Method: Composition-based stats.

Identities = 29/186 (16%), Positives = 65/186 (35%), Gaps = 22/186 (12%)

Query 4 LTERDLSVLGSYARDGNRELYWN-YLSQLPGADGYGTLALGVVRNDSLPGRVANTYAQDY 62

LT++++ + G Y + Y ALGV D++ G A + Q

Sbjct 7 LTKKEIDAWSEQIKSGKLSAIGEVYQALAQKGYDYAHWALGVATADTITGNGALQFMQAV 66

Query 63 AKSQQEEGSRFPNAQLTERQWESFGQTLLERDLELRQQWMNERRPDLALNLPGKDVMLAH 122

AK +L + + +S + + L + Q+ + + ++ ++++ H

Sbjct 67 AKDH--------KQKLPQARVDSVRRDMALGYLAMLQKKLENGKGG--EDITYEEMLKFH 116

Query 123 DRAFERHELDPNCWTPRVLLQAAEQK-----------SGPAKLEQIWTNMLNNDYAGGPR 171

+ F ++L+ WT + + G +E +W +M G

Sbjct 117 VKVFNDNKLNIGYWTLYTPMSIIQNHASATGSNGQVIGGKQVVENMWQHMRATQGTGLTG 176

Query 172 VGNTSV 177

+ +

Sbjct 177 SWLSLM 182

>WP_029240182.1 calcium-binding protein [Ralstonia solanacearum]

Length=1543

Score = 138 bits (348), Expect = 1e-32, Method: Composition-based stats.

Identities = 28/176 (16%), Positives = 53/176 (30%), Gaps = 13/176 (7%)

Query 22 ELYWNYLSQLPGADGYGTLALGVVRNDSLPGRVANTYAQDYAKSQQEEGSRFPNAQLTER 81

++ YL Y GV R +S+ G A Y A +++

Sbjct 2 SEFYFYL--QDRGYSYAGWGGGVARENSIAGISAIDYLTGSALM---GMGGEACWNISQW 56

Query 82 QWESFGQTLLERDLELRQQWMNE-----RRPDLALNLPGKDVMLAHDRAFERHELDPNCW 136

+ + Q + E L E + ++ ++V H F+ + L W

Sbjct 57 KSDKIKQEMAEAYLNRLDTIAQENKRLTGNYEAGRDIQAQEVWDFHKEVFKNNGLGIENW 116

Query 137 TPRVLLQAAEQKSGPAKLEQIWTNMLNNDYAGGPRVG---NTSVDAISQMGWTKGG 189

T + + +Q G LE W ++ + G T + +

Sbjct 117 TLDSVFKIIQQTQGEDALETYWESLRDTQGEGMMATLLNIRTMYNMHESIDSADPA 172

>WP_100139132.1 hypothetical protein [Snodgrassella alvi]

PIT47825.1 hypothetical protein BHC46_05955 [Snodgrassella alvi]

Length=1609

Score = 138 bits (348), Expect = 1e-32, Method: Composition-based stats.

Identities = 30/186 (16%), Positives = 63/186 (34%), Gaps = 22/186 (12%)

Query 4 LTERDLSVLGSYARDGNRELYWN-YLSQLPGADGYGTLALGVVRNDSLPGRVANTYAQDY 62

LT+ L + G Y + Y ALGV D++ G A + Q

Sbjct 7 LTKEQLKAWSEQIKTGKLSAIGEVYQALAQKGYDYAHWALGVATADTITGNGALQFMQAV 66

Query 63 AKSQQEEGSRFPNAQLTERQWESFGQTLLERDLELRQQWMNERRPDLALNLPGKDVMLAH 122

AK+ +L + + +S + + L + Q+ + + ++ ++++ H

Sbjct 67 AKNH--------KQKLPQARVDSVRRDMALGYLAMLQKKLENGKGG--EDITYEEMLDFH 116

Query 123 DRAFERHELDPNCWTPRVLLQAAEQK-----------SGPAKLEQIWTNMLNNDYAGGPR 171

F ++L+ WT + + G +E +W +M G

Sbjct 117 VTVFNDNKLNIGYWTLYTPMSIIQNHASATGSNGQVIGGKQVVENMWQHMRATQGTGLTG 176

Query 172 VGNTSV 177

+ +

Sbjct 177 SWLSLM 182

>AIS17384.1 hypothetical protein LT40_08225 [Pseudomonas rhizosphaerae]

Length=1378

Score = 138 bits (347), Expect = 1e-32, Method: Composition-based stats.

Identities = 38/222 (17%), Positives = 66/222 (30%), Gaps = 25/222 (11%)

Query 6 ERDLSVLGSYARDGNRELYWNYLSQLPGADGYGTLALGVVRNDSLPGRVANTYAQDYAKS 65

+L V + YL+ Y LA GV +SL G A +Y + AKS

Sbjct 7 RAELDVASKILVTEGVSKMYGYLADK--GFNYARLANGVALGNSLSGEAAISYMKSVAKS 64

Query 66 QQEEGSRFPNAQLTERQWESFGQTLLERDLELRQQWMNERRPDLALNLPGKDVMLAHDRA 125

+ LTE + + L + ++ K+ H

Sbjct 65 HGK--------VLTEADVDKVRWDMAHGYLNTLSSLAVGDDASVGRDVTAKEASRFHTNV 116

Query 126 FERHELDPNCWTPRVLLQAAEQKSGPAKLEQIWTNMLNNDY---AGGPRVGNTSVDAISQ 182

FE + L WT + + S ++E W +L + T

Sbjct 117 FEANGLPIQAWTLDSIFKIINDSS---EIEAYWEKVLESAGHADKELELAFETMGLMYHS 173

Query 183 MG----WTKGGQYLTRLSVLEA-----TQALEGRSAVDPNVI 215

+ +L + ++ T R ++D +V

Sbjct 174 YDTGSNVLESSTWLGHMISFDSVWSTLTHVFAPRESIDFDVY 215

>WP_064047592.1 hypothetical protein [Ralstonia solanacearum]

OAI72924.1 hypothetical protein RSP781_00760 [Ralstonia solanacearum]

Length=1546

Score = 138 bits (347), Expect = 2e-32, Method: Composition-based stats.

Identities = 28/176 (16%), Positives = 53/176 (30%), Gaps = 13/176 (7%)

Query 22 ELYWNYLSQLPGADGYGTLALGVVRNDSLPGRVANTYAQDYAKSQQEEGSRFPNAQLTER 81

++ YL Y GV R +S+ G A Y A +++

Sbjct 2 SEFYFYL--QDRGYSYAGWGGGVARENSIAGISAIDYLTGSALM---GMGGEACWNISQW 56

Query 82 QWESFGQTLLERDLELRQQWMNE-----RRPDLALNLPGKDVMLAHDRAFERHELDPNCW 136

+ + Q + E L E + ++ ++V H F+ + L W

Sbjct 57 KSDKIKQEMAEAYLNRLDTIAQENKRLTGNYEAGRDIQAQEVWDFHKEVFKNNGLGIENW 116

Query 137 TPRVLLQAAEQKSGPAKLEQIWTNMLNNDYAGGPRVG---NTSVDAISQMGWTKGG 189

T + + +Q G LE W ++ + G T + +

Sbjct 117 TLDSVFKIIQQTQGDDALEAYWESLRDTQGEGPMATLLNIRTMYNMHESIDSADPA 172

>AGH82632.1 hypothetical protein F504_110 [Ralstonia solanacearum FQY_4]

Length=1769

Score = 138 bits (346), Expect = 2e-32, Method: Composition-based stats.

Identities = 28/176 (16%), Positives = 53/176 (30%), Gaps = 13/176 (7%)

Query 22 ELYWNYLSQLPGADGYGTLALGVVRNDSLPGRVANTYAQDYAKSQQEEGSRFPNAQLTER 81

++ YL Y GV R +S+ G A Y A +++

Sbjct 2 SEFYFYL--QDRGYSYAGWGGGVARENSIAGISAIDYLTGSALM---GMGGEACWNISQW 56

Query 82 QWESFGQTLLERDLELRQQWMNE-----RRPDLALNLPGKDVMLAHDRAFERHELDPNCW 136

+ + Q + E L E + ++ ++V H F+ + L W

Sbjct 57 KSDKIKQEMAEAYLNRLDTIAQENKRLTGNYEAGRDIQAQEVWDFHKEVFKNNGLGIENW 116

Query 137 TPRVLLQAAEQKSGPAKLEQIWTNMLNNDYAGGPRVG---NTSVDAISQMGWTKGG 189

T + + +Q G LE W ++ + G T + +

Sbjct 117 TLDSVFKIIQQTQGDDALEAYWESLRDTQGEGPMATLLNIRTMYNMHESIDSADPA 172

>WP_064820771.1 calcium-binding protein [Ralstonia solanacearum]

Length=1769

Score = 138 bits (346), Expect = 2e-32, Method: Composition-based stats.

Identities = 28/176 (16%), Positives = 53/176 (30%), Gaps = 13/176 (7%)

Query 22 ELYWNYLSQLPGADGYGTLALGVVRNDSLPGRVANTYAQDYAKSQQEEGSRFPNAQLTER 81

++ YL Y GV R +S+ G A Y A +++

Sbjct 2 SEFYFYL--QDRGYSYAGWGGGVARENSIAGISAIDYLTGSALM---GMGGEACWNISQW 56

Query 82 QWESFGQTLLERDLELRQQWMNE-----RRPDLALNLPGKDVMLAHDRAFERHELDPNCW 136

+ + Q + E L E + ++ ++V H F+ + L W

Sbjct 57 KSDKIKQEMAEAYLNRLDTIAQENKRLTGNYEAGRDIQAQEVWDFHKEVFKNNGLGIENW 116

Query 137 TPRVLLQAAEQKSGPAKLEQIWTNMLNNDYAGGPRVG---NTSVDAISQMGWTKGG 189

T + + +Q G LE W ++ + G T + +

Sbjct 117 TLDSVFKIIQQTQGDDALEAYWESLRDTQGEGPMATLLNIRTMYNMHESIDSADPA 172

>WP_054572161.1 peptidase S8 [Pseudomonas putida]

KPM67885.1 peptidase S8 [Pseudomonas putida]

Length=1807

Score = 138 bits (346), Expect = 2e-32, Method: Composition-based stats.

Identities = 31/166 (19%), Positives = 61/166 (37%), Gaps = 16/166 (10%)

Query 1 MSGLTERDLSVLGSYARDGNRELYWNYLSQLPGADGYGTLALGVVRNDSLPGRVANTYAQ 60

MSGLTE D+ ++Y++ D Y LA GV + +S+ G A + +

Sbjct 1 MSGLTEEDVKNARKIFEFQGVGEMYDYMAMK--GDRYAVLANGVAKGNSIAGLAAIDFMR 58

Query 61 DYAKSQQEEGSRFPNAQLTERQWESFGQTLLERDLELRQQWMNERRPDLALNLPGKDVML 120

++ E + + L+L+++ + E + +L +V

Sbjct 59 RTEAD--------AGRPMSAEDVEKVRLEMGKAYLDLQERRVKEG--TIGGDLGHLEVWK 108

Query 121 AHDRAFERHELDPNCWTPRVLLQAAEQKSGPAKLEQIWTNMLNNDY 166

H FE+ + WT + + E+ W ++L +

Sbjct 109 FHSDVFEKLGRSKDAWTLNSVFELMRS----DAREKYWQDVLGSAG 150

>PPC94133.1 hypothetical protein CTY35_10805, partial [Methylotenera sp.]

Length=784

Score = 137 bits (344), Expect = 3e-32, Method: Composition-based stats.

Identities = 32/166 (19%), Positives = 62/166 (37%), Gaps = 15/166 (9%)

Query 2 SGLTERDLSVLGSYAR-DGNRELYWNYLSQLPGADGYGTLALGVVRNDSLPGRVANTYAQ 60

+ LT DL+ S G ++Y+ + Y LA G+V + G A + +

Sbjct 5 NTLTTNDLAYAKSLLDTQGGVSAAYSYM--IINGYTYAQLANGLVSDGDFEGSFALRFME 62

Query 61 DYAKSQQEEGSRFPNAQLTERQWESFGQTLLERDLELRQQWMNERRPDLALNLPGKDVML 120

A+SQ LT+ + ++ + + L+ Q E + ++ +

Sbjct 63 AQAESQG--------NPLTQLEIDAIKRDMAFAYLDALQSRAMENGGYIDSDVTVIEAEN 114

Query 121 AHDRAFERHELDPNCWTPRVLLQAAEQKSGPAKLEQIWTNMLNNDY 166

H++ F + L P WT + ++ E +W +L N

Sbjct 115 FHNQVFTDNGLSPEAWTLYEPFRGM----SESEKELVWKEILENTG 156

>CUV47316.1 conserved protein of unknown function, partial [Ralstonia solanacearum]

Length=1056

Score = 137 bits (345), Expect = 3e-32, Method: Composition-based stats.

Identities = 32/194 (16%), Positives = 61/194 (31%), Gaps = 13/194 (7%)

Query 4 LTERDLSVLGSYARDGNRELYWNYLSQLPGADGYGTLALGVVRNDSLPGRVANTYAQDYA 63

+ L L G ++NYL A GV R DS+ G A Y A

Sbjct 5 INREKLDHLEGLLSKGMISEFYNYLKNEGYAYA--GWGGGVAREDSIAGISAIDYLTGSA 62

Query 64 KSQQEEGSRFPNAQLTERQWESFGQTLLERDLELR-----QQWMNERRPDLALNLPGKDV 118

++ + + + + E L+ + ++ ++ K+V

Sbjct 63 LM---GMGGEACRNISPDKSDLIKKGMAEAYLQALNTIAKENKTLTGNDEVNRDINAKEV 119

Query 119 MLAHDRAFERHELDPNCWTPRVLLQAAEQKSGPAKLEQIWTNMLNNDYAGGPRVG---NT 175

+ H + F+ + L WT + + +Q G LE W ++ + G T

Sbjct 120 LDFHQKVFKENGLGIENWTLDSVFKIIQQTQGDDALEAYWESLRDTQGEGMMATLLNIRT 179

Query 176 SVDAISQMGWTKGG 189

+ +

Sbjct 180 MYNMHESIDSADPA 193

>WP_037490581.1 hypothetical protein [Snodgrassella alvi]

KDN15454.1 Alkaline phosphatase [Snodgrassella alvi]

PIT07145.1 hypothetical protein BGI29_09545 [Snodgrassella alvi]

PIT25944.1 hypothetical protein BGI38_08965 [Snodgrassella alvi]

PIT27718.1 hypothetical protein BGI39_08045 [Snodgrassella alvi]

PIT31290.1 hypothetical protein BGI40_10150 [Snodgrassella alvi]

Length=1609

Score = 137 bits (345), Expect = 3e-32, Method: Composition-based stats.

Identities = 30/182 (16%), Positives = 63/182 (35%), Gaps = 22/182 (12%)

Query 4 LTERDLSVLGSYARDGNRELYWN-YLSQLPGADGYGTLALGVVRNDSLPGRVANTYAQDY 62

LT++++ + G Y + Y ALGV D++ G A + Q

Sbjct 7 LTKKEIDAWSEQIKSGKLSAIGEVYQALAQKGYDYAHWALGVATADTITGNGALQFMQAV 66

Query 63 AKSQQEEGSRFPNAQLTERQWESFGQTLLERDLELRQQWMNERRPDLALNLPGKDVMLAH 122

AK +LT+ + +S + + L + Q+ + + ++ ++++ H

Sbjct 67 AKDH--------KQKLTQARVDSVRRDMALGYLAMLQKKLENSKGG--EDITYEEMLKFH 116

Query 123 DRAFERHELDPNCWTPRVLLQAAEQK-----------SGPAKLEQIWTNMLNNDYAGGPR 171

F ++L+ WT + + G +E +W +M G

Sbjct 117 VTVFNNNKLNIGYWTLYTPMSIIQNHASATGSNGQVIGGKQVVENMWQHMRATQGTGLTG 176

Query 172 VG 173

Sbjct 177 SW 178

>WP_079227773.1 peptidase S8 [Pseudomonas putida]

Length=1785

Score = 137 bits (344), Expect = 4e-32, Method: Composition-based stats.

Identities = 45/270 (17%), Positives = 76/270 (28%), Gaps = 32/270 (12%)

Query 1 MSGLTERDLSVLGSYARDGNRELYWNYLSQLPGADGYGTLALGVVRNDSLPGRVANTYAQ 60

MSGL + D+ ++YL D Y LA GV R +S+ G A + +

Sbjct 1 MSGLLQEDVRRAREIFESEGIGAMYDYLESK--GDRYAILANGVARGNSIAGIAAIDFMK 58

Query 61 DYAKSQQEEGSRFPNAQLTERQWESFGQTLLERDLELRQQWMNERRPDLALNLPGKDVML 120

+ + E E + E L + + + + L + +V

Sbjct 59 RTEQG--------AGRPMQEEDVEQVRFKMAEAYLGVLAEKVKAG--TIDLEINHLEVWG 108

Query 121 AHDRAFERHELDPNCWTPRVLLQAAEQKSGPAKLEQIWTNMLNNDYAGGPRVG------- 173

H FE + WT + E W +L + +

Sbjct 109 FHSNVFEDMGRSKDAWTLNTVFNLLH----EDARETYWKEVLGAAGSLPSELWLSLRTDA 164

Query 174 --NTSVDAISQMGWTKGGQYLTRLSVL-EATQALEGRSAVDPNVIGGNSYYAMYFEADRK 230

S + + + R+ L G V N I + D

Sbjct 165 SVAFSSAMAPEELRVQAESWKARIDSPGGVASVLLGLGTVTANGI--QGVISDLINTDGP 222

Query 231 WASISAGGGHMSLREITDPSR-IAELNDAR 259

S + + TD R + +D R

Sbjct 223 APSQPI---QIEITPTTDSRRDLQGQDDIR 249

>WP_038034741.1 hypothetical protein [Thioalkalivibrio sp. AKL7]

Length=1501

Score = 137 bits (344), Expect = 4e-32, Method: Composition-based stats.

Identities = 45/271 (17%), Positives = 89/271 (33%), Gaps = 41/271 (15%)

Query 2 SGLTERDLSVLGSYARDGNRELYWNYLSQLPGADGYGTLALGVVRNDSLPGRVANTYAQD 61

+ +T+ +L S G ++YLS D Y LA GVV +L G+ A T+ +

Sbjct 8 TPITQDELDHARSLYAGGQLAEMYDYLSS--FGDRYSILANGVVEGGTLSGQAALTFMEK 65

Query 62 YAKSQQEEGSRFPNAQLTERQWESFGQTLLERDLELRQQWMNERRPDL-ALNLPGKDVML 120

A+ + + + + L Q ++ + + +

Sbjct 66 AAED--------EGVVFGGYELDLIRSDMADEYLITLQGILDSSDQGIIEREINPSEAHA 117

Query 121 AHDRAFERHELDPNCWTPRVLLQAAEQKSGPAKLEQIWTNMLNN---------------D 165

H + FER+ L + WT + + E +W +L +

Sbjct 118 FHSKVFERNGLGADAWTLHIPFSLM----SEDQREGMWQAVLESTDHFGREAALTVALGA 173

Query 166 YAGGPRVGNTSVDAISQMGWTKG---GQYLTRLSVLEATQALEGRSAVDPNVI---GGNS 219

+ R+G + D + W + + L++ Q LE + + GG +

Sbjct 174 WMVNARIGTDAADVENINDWWGRFLEAEVIAVLALTARDQGLEESRTWGSDFLEEMGGPA 233

Query 220 YYAMYFEADRKWASISAGGGHMSLREITDPS 250

+ W + GG +R+ +

Sbjct 234 GIW-----ESAWDYWTGGGFIGDIRDFFNRG 259

>WP_110414045.1 hypothetical protein [Snodgrassella alvi]

PXY97133.1 hypothetical protein DKK71_03530 [Snodgrassella alvi]

Length=1608

Score = 137 bits (344), Expect = 4e-32, Method: Composition-based stats.

Identities = 28/182 (15%), Positives = 59/182 (32%), Gaps = 22/182 (12%)

Query 4 LTERDLSVLGSYARDGNRELYWN-YLSQLPGADGYGTLALGVVRNDSLPGRVANTYAQDY 62

LT++++ + G Y + Y A GV D+L G A + Q

Sbjct 20 LTKKEIDAWSEQIKTGELSAIGEVYQALAQKGYDYAQWAFGVATADTLTGNGALQFMQAV 79

Query 63 AKSQQEEGSRFPNAQLTERQWESFGQTLLERDLELRQQWMNERRPDLALNLPGKDVMLAH 122

AK +L + + +S + + L + ++ + + ++ +++ H

Sbjct 80 AKDH--------KQKLPQARVDSVRRDMALGYLAMLKEKLENGKGGK--DITYYEMLEFH 129

Query 123 DRAFERHELDPNCWTPRVLLQAAEQK-----------SGPAKLEQIWTNMLNNDYAGGPR 171

F ++LD WT + + G +E +W +M

Sbjct 130 VEVFNNNKLDIGYWTLYTPMSIIQNHASATGSNGQEIDGEQVVENMWQHMRATKGTKLTG 189

Query 172 VG 173

Sbjct 190 SW 191

>WP_084563889.1 hypothetical protein [Snodgrassella alvi]

ORF41041.1 hypothetical protein BGI12_00995 [Snodgrassella alvi]

Length=1608

Score = 136 bits (343), Expect = 5e-32, Method: Composition-based stats.

Identities = 28/182 (15%), Positives = 59/182 (32%), Gaps = 22/182 (12%)

Query 4 LTERDLSVLGSYARDGNRELYWN-YLSQLPGADGYGTLALGVVRNDSLPGRVANTYAQDY 62

LT++++ + G Y + Y A GV D+L G A + Q

Sbjct 20 LTKKEIDAWSEQIKTGELSAIGEVYQALAQKGYDYAQWAFGVATADTLTGNGALQFMQAV 79

Query 63 AKSQQEEGSRFPNAQLTERQWESFGQTLLERDLELRQQWMNERRPDLALNLPGKDVMLAH 122

AK +L + + +S + + L + ++ + + ++ +++ H

Sbjct 80 AKDH--------KQKLPQARVDSVRRDMALGYLAMLKEKLENGKGGK--DITYYEMLEFH 129

Query 123 DRAFERHELDPNCWTPRVLLQAAEQK-----------SGPAKLEQIWTNMLNNDYAGGPR 171

F ++LD WT + + G +E +W +M

Sbjct 130 VEVFNNNKLDIGYWTLYTPMSIIQNHASATSSNGQEIDGEQVVENMWQHMRATKGTKLTG 189

Query 172 VG 173

Sbjct 190 SW 191

>WP_100149648.1 hypothetical protein [Snodgrassella alvi]

PIT19843.1 hypothetical protein BGI36_10225 [Snodgrassella alvi]

Length=1609

Score = 136 bits (342), Expect = 6e-32, Method: Composition-based stats.

Identities = 31/186 (17%), Positives = 63/186 (34%), Gaps = 22/186 (12%)

Query 4 LTERDLSVLGSYARDGNRELYWN-YLSQLPGADGYGTLALGVVRNDSLPGRVANTYAQDY 62

LT+ L + G Y + Y ALGV D++ G A + Q

Sbjct 7 LTKEQLRAWSEQIKTGKLSAIGEVYQALAKKGYDYAQWALGVATADTITGNGALQFMQAV 66

Query 63 AKSQQEEGSRFPNAQLTERQWESFGQTLLERDLELRQQWMNERRPDLALNLPGKDVMLAH 122

AK +L + + +S + + L + Q + + + ++ K+++ H

Sbjct 67 AKDH--------KQKLPQARVDSVRRDMALGYLAMLQDKLAKGKGG--EDITYKEMLKFH 116

Query 123 DRAFERHELDPNCWTPRVLLQAAEQK-----------SGPAKLEQIWTNMLNNDYAGGPR 171

+ F ++L+ WT + + G +E +W +M G

Sbjct 117 VKVFNDNKLNIGYWTLYTPMSIIQNHASATGSNGQEIGGELVVENMWQHMRATQGTGLTG 176

Query 172 VGNTSV 177

+ +

Sbjct 177 SWLSLM 182

>WP_037406346.1 hypothetical protein [Snodgrassella alvi]

KDN12548.1 Alkaline phosphatase [Snodgrassella alvi]

Length=1609

Score = 136 bits (341), Expect = 9e-32, Method: Composition-based stats.

Identities = 31/182 (17%), Positives = 58/182 (32%), Gaps = 22/182 (12%)

Query 4 LTERDLSVLGSYARDGNRELYWN-YLSQLPGADGYGTLALGVVRNDSLPGRVANTYAQDY 62

LT+ L + G Y + Y ALGV D++ G A + Q

Sbjct 7 LTKEQLRAWSEQIKTGKLSAIGEVYQALAQKGYDYAHWALGVATADTITGNGALQFMQAV 66

Query 63 AKSQQEEGSRFPNAQLTERQWESFGQTLLERDLELRQQWMNERRPDLALNLPGKDVMLAH 122

AK +L + + +S + + L + Q + + ++ +++ H

Sbjct 67 AKDH--------KQKLPQARVDSVRRDMALGYLAMLQDKLANGKGG--EDITYYEMLDFH 116

Query 123 DRAFERHELDPNCWTPRVLLQAAEQK-----------SGPAKLEQIWTNMLNNDYAGGPR 171

F ++LD WT + + G +E +W +M G

Sbjct 117 VTVFNNNKLDIGYWTLYTPMSIIQNHASATGSNGQVIGGKQVVENMWQHMRATQGTGLTG 176

Query 172 VG 173

Sbjct 177 SW 178

>WP_100101807.1 hypothetical protein [Snodgrassella alvi]

PIT22485.1 hypothetical protein BGI35_04470 [Snodgrassella alvi]

Length=1622

Score = 136 bits (341), Expect = 9e-32, Method: Composition-based stats.

Identities = 31/186 (17%), Positives = 64/186 (34%), Gaps = 22/186 (12%)

Query 4 LTERDLSVLGSYARDGNRELYWN-YLSQLPGADGYGTLALGVVRNDSLPGRVANTYAQDY 62

LT+ L + G Y + Y ALGV D++ G A + Q

Sbjct 20 LTKEQLRAWSEQIKTGKLSAIGEVYQALAQKGYDYAHWALGVATADTITGNGALQFMQAV 79

Query 63 AKSQQEEGSRFPNAQLTERQWESFGQTLLERDLELRQQWMNERRPDLALNLPGKDVMLAH 122

AK +L + + +S + + L + Q +N+ + ++ ++++ H

Sbjct 80 AKDH--------KQKLPQARVDSVRRDMALGYLAMLQTKLNKGKGG--EDITYEEMLEFH 129

Query 123 DRAFERHELDPNCWTPRVLLQAAEQK-----------SGPAKLEQIWTNMLNNDYAGGPR 171

+ F ++L+ WT + + G +E +W +M G

Sbjct 130 VKVFNDNKLNIGYWTLYTPMSIIQNHASATGSNGQEIGGDLVVENMWQHMRATQGTGLTG 189

Query 172 VGNTSV 177

+ +

Sbjct 190 SWLSLM 195

>WP_027916848.1 MULTISPECIES: peptidase S8 [Pseudomonas]

Length=1785

Score = 135 bits (339), Expect = 2e-31, Method: Composition-based stats.

Identities = 36/225 (16%), Positives = 65/225 (29%), Gaps = 26/225 (12%)

Query 1 MSGLTERDLSVLGSYARDGNRELYWNYLSQLPGADGYGTLALGVVRNDSLPGRVANTYAQ 60

MSGL + D+ ++YL+ D Y LA GV + +S+ G A + +

Sbjct 1 MSGLLQEDVRRAREIFESERTGAMYDYLASK--GDRYAVLANGVAKGNSIAGIAAIDFMK 58

Query 61 DYAKSQQEEGSRFPNAQLTERQWESFGQTLLERDLELRQQWMNERRPDLALNLPGKDVML 120

+ + + E E + E L + + + + L + +

Sbjct 59 RTEQG--------ADRPMQEEDVEQVRFKMAEAYLGVLDEKVKAG--SIGLEINHLEAWG 108

Query 121 AHDRAFERHELDPNCWTPRVLLQAAEQKSGPAKLEQIWTNMLNNDYAGGPRVG------- 173

H FE + WT + E W +L + +

Sbjct 109 FHSNVFEDMGRSKDAWTLNTVFNLLH----EDARETYWKEVLGAAGSLPSELWLSLRTDA 164

Query 174 --NTSVDAISQMGWTKGGQYLTRLSVL-EATQALEGRSAVDPNVI 215

S + + + R+ L G V N I

Sbjct 165 SVAFSSAMAPEELRVQAESWKARIDSPGGVASVLLGLGTVTANGI 209

>WP_131390282.1 hypothetical protein [Acinetobacter sp. ANC 3791]

TCB81919.1 hypothetical protein E0H90_14705 [Acinetobacter sp. ANC 3791]

Length=3366

Score = 135 bits (339), Expect = 2e-31, Method: Composition-based stats.

Identities = 41/237 (17%), Positives = 76/237 (32%), Gaps = 13/237 (5%)

Query 1 MS---GLTERDLSVLGSYARDGNRELY-WNYLSQLPGADGYGTLALGVVRNDSLPGRVAN 56

MS LT +S L Y GY A GV DS+ G A

Sbjct 1 MSVTQALTTEKISALKKLVDTDGINSVPGIYTELNNMGFGYAGWAYGVSTGDSVTGMGAL 60

Query 57 TYAQDYAKSQQEEGSRFPNAQLTERQWESFGQTLLERDLELRQQWMNERRPDLALNLPGK 116

+ ++ A+ +++ + +L+ L+ + + ++

Sbjct 61 DFMENTAEK--------AGVTISDEKVTQIRVGMLKGYLDALMLEAESKGGSVNKDIDFL 112

Query 117 DVMLAHDRAFERHELDPNCWTPRVLLQAAEQKSGPAKLEQIWTNMLNNDYAGGPRVGNTS 176

+ H F+ L + WT + G K+ QIW M + G + ++

Sbjct 113 QIKAFHAGVFKGVGLSIDYWTLNTPMNIIFNLFGLNKVNQIWEKMTETNGTGPDALLSSL 172

Query 177 VDAISQMGWTKGGQYLTRLSVLEATQALEGRSAVDPNVIGGNSYYAMYFEADRKWAS 233

M + G YL + ++ L NV+ +Y + ++W S

Sbjct 173 DLVNIVMEASYGKIYLDASGNIISSVKLATHPERMNNVV-KTLDTGIYVKDAQEWLS 228

>WP_100102283.1 calcium-binding protein [Snodgrassella alvi]

PIT20764.1 hypothetical protein BGI35_07935 [Snodgrassella alvi]

Length=1694

Score = 134 bits (338), Expect = 3e-31, Method: Composition-based stats.

Identities = 32/221 (14%), Positives = 73/221 (33%), Gaps = 22/221 (10%)

Query 5 TERDLSVLGSYARDGNRELYWN-YLSQLPGADGYGTLALGVVRNDSLPGRVANTYAQDYA 63

+ ++ +G+ YL Y A GV D++ G A + Q A

Sbjct 8 SAAEIRAWRKQIENGDLSDVGAVYLQLAKRGYHYAKWAYGVASADTITGNGALEFMQAVA 67

Query 64 KSQQEEGSRFPNAQLTERQWESFGQTLLERDLELRQQWMNERRPDLALNLPGKDVMLAHD 123

LT + + + L++ ++ ++ ++ K++ H

Sbjct 68 NEHNH--------ILTADETNKIRRYMALGYLDMLEEKA--GSGSVSQDISYKEMREFHI 117

Query 124 RAFERHELDPNCWTPRVLLQAAEQKS-----------GPAKLEQIWTNMLNNDYAGGPRV 172

+ F+ + +D N WT ++ E+ + G +EQ+W M

Sbjct 118 KVFKDNNVDINYWTLYEPMRIIERYASGKLADGKVVTGEDVVEQVWAEMWATKGTNVDSW 177

Query 173 GNTSVDAISQMGWTKGGQYLTRLSVLEATQALEGRSAVDPN 213

+++ G Y+ + + + ++ G D +

Sbjct 178 FSSNELFQIMNDAQNGYIYVDKKTGVPISKFSMGLQLSDAD 218

>WP_060507474.1 peptidase S8 [Pseudomonas sp. NBRC 111124]

Length=1785

Score = 134 bits (338), Expect = 3e-31, Method: Composition-based stats.

Identities = 36/212 (17%), Positives = 70/212 (33%), Gaps = 22/212 (10%)

Query 1 MSGLTERDLSVLGSYARDGNRELYWNYLSQLPGADGYGTLALGVVRNDSLPGRVANTYAQ 60

M+GL D+ ++YL+ D Y LA GV + +S+ G VA + +

Sbjct 1 MTGLLIEDVRRARELFESQGVSDMYDYLASK--GDRYAVLANGVAKGNSVAGVVAIDFMK 58

Query 61 DYAKSQQEEGSRFPNAQLTERQWESFGQTLLERDLELRQQWMNERRPDLALNLPGKDVML 120

+ + + E+ + E L L + + E + + ++V

Sbjct 59 RTERD--------AGRPMQDADVETVRFKMAEAYLGLLDKKVREGTIGV--EINHEEVWG 108

Query 121 AHDRAFERHELDPNCWTPRVLLQAAEQKSGPAKLEQIWTNMLNNDYAGGPRVGNTSVDAI 180

H F+ + WT + + +K + W +L+ G + +

Sbjct 109 FHTEVFQGLGRSKDAWTLNTIFELKHEK----ARDGYWKMVLDAAGKPGSEL---ELAVS 161

Query 181 SQMGWTKGGQYLTRLSVLEATQALEGRSAVDP 212

+ M + V QA S +D

Sbjct 162 TDM---QVSYATAMAPVDLRAQAKSWLSRIDS 190

>WP_085614738.1 MULTISPECIES: peptidase S8 [Pseudomonas]

Length=1807

Score = 134 bits (338), Expect = 3e-31, Method: Composition-based stats.

Identities = 35/212 (17%), Positives = 69/212 (33%), Gaps = 22/212 (10%)

Query 1 MSGLTERDLSVLGSYARDGNRELYWNYLSQLPGADGYGTLALGVVRNDSLPGRVANTYAQ 60

M+ L E D+ ++YL+ D Y LA GV + +S+ G A + +

Sbjct 1 MAALVEGDVRRAREIFESQGPGGMYDYLA--GRGDKYALLANGVAKGNSIAGLAALEFMK 58

Query 61 DYAKSQQEEGSRFPNAQLTERQWESFGQTLLERDLELRQQWMNERRPDLALNLPGKDVML 120

+ + ER + + + L L + + + L+ K+V

Sbjct 59 RTERD--------AGRVMDERDVQLVRVEMAKAYLGLLEDKVKSDSIGVTLD--HKEVWG 108

Query 121 AHDRAFERHELDPNCWTPRVLLQAAEQKSGPAKLEQIWTNMLNNDYAGGPRVGNTSVDAI 180

H+ FE+ + WT + + P+ E W ++L++ + V + I

Sbjct 109 FHNEVFEKLGRSRDAWTLNSVFELMV----PSDRETYWRSVLDSAGSPAGEVWLS----I 160

Query 181 SQMGWTKGGQYLTRLSVLEATQALEGRSAVDP 212

+ A +D

Sbjct 161 ETDRKLSTASAIA--PQHLRPMAKYWLERIDS 190

>CUV31962.1 conserved protein of unknown function, partial [Ralstonia solanacearum]

Length=1086

Score = 134 bits (336), Expect = 4e-31, Method: Composition-based stats.

Identities = 33/198 (17%), Positives = 63/198 (32%), Gaps = 14/198 (7%)

Query 1 MS-GLTERDLSVLGSYARDGNRELYWNYLSQLPGADGYGTLALGVVRNDSLPGRVANTYA 59

M + DLS L G ++ YL + GV R DS+ G A Y

Sbjct 1 MPQTIKRDDLSHLKDLLHMGLVSDFYLYLQKQGYGYA--GWGGGVAREDSIAGISAVDYL 58

Query 60 QDYAKSQQEEGSRFPNAQLTERQWESFGQTLLERDLELRQQWMNE-----RRPDLALNLP 114

A L+ + + + + + L ++ E ++ ++

Sbjct 59 TGSALM---GMGGQACWDLSTDKSKIIKKEMAQAYLGSLEKIAGENKRLTGHDEVNRDIR 115

Query 115 GKDVMLAHDRAFERHELDPNCWTPRVLLQAAEQKSGPAKLEQIWTNMLNNDYAGGPRVG- 173

++V H F+++ L WT + + +Q G LE W ++ + G

Sbjct 116 AQEVWDFHREVFQKNGLGIENWTLDSVFKIIQQTQGEDALETYWESLRDTQGEGMMATLL 175

Query 174 --NTSVDAISQMGWTKGG 189

T + +

Sbjct 176 NIRTMYNMHESIDSADPA 193

>WP_110414750.1 hypothetical protein [Snodgrassella alvi]

PXY95776.1 hypothetical protein DKK71_10800 [Snodgrassella alvi]

Length=1696

Score = 134 bits (336), Expect = 5e-31, Method: Composition-based stats.

Identities = 35/219 (16%), Positives = 72/219 (33%), Gaps = 22/219 (10%)

Query 7 RDLSVLGSYARDGNRELYWN-YLSQLPGADGYGTLALGVVRNDSLPGRVANTYAQDYAKS 65

+ +G+ YL Y A GV D++ G A + Q A

Sbjct 10 NQIRAWRKQIENGDLSDAGEVYLQLAKRGYYYAKWAYGVESADTITGNGALEFMQAMATE 69

Query 66 QQEEGSRFPNAQLTERQWESFGQTLLERDLELRQQWMNERRPDLALNLPGKDVMLAHDRA 125

+Q LT + + + L+ ++ N ++ ++ K++ H

Sbjct 70 KQ--------QILTFDKTNKIRKEMALAYLDTLEK--NAGAGSISQDISYKEMREFHISV 119

Query 126 FERHELDPNCWTPRVLLQAAEQKS-----------GPAKLEQIWTNMLNNDYAGGPRVGN 174

F+ + +D N WT ++ EQ + G +EQ+W M +

Sbjct 120 FKNNGIDINYWTLYEPMRIIEQYASGKMSDGREIKGEQVVEQVWEAMWETKGTNVDSWFS 179

Query 175 TSVDAISQMGWTKGGQYLTRLSVLEATQALEGRSAVDPN 213

++ G Y+ + + + ++ G D +

Sbjct 180 SNELFQIMNDVQSGYIYIDKKTGVPISKFSMGLQLSDAD 218

>CUV53102.1 conserved protein of unknown function, partial [Ralstonia solanacearum]

Length=1048

Score = 133 bits (334), Expect = 6e-31, Method: Composition-based stats.

Identities = 33/198 (17%), Positives = 63/198 (32%), Gaps = 14/198 (7%)

Query 1 MS-GLTERDLSVLGSYARDGNRELYWNYLSQLPGADGYGTLALGVVRNDSLPGRVANTYA 59

M + DLS L G ++ YL + GV R DS+ G A Y

Sbjct 1 MPQTIKRDDLSHLKDLLHMGLVSDFYLYLQKQGYGYA--GWGGGVAREDSIAGISAVDYL 58

Query 60 QDYAKSQQEEGSRFPNAQLTERQWESFGQTLLERDLELRQQWMNE-----RRPDLALNLP 114

A L+ + + + + + L ++ E ++ ++

Sbjct 59 TGSALM---GMGGQACWDLSTDKSKIIKKEMAQAYLGSLEKIAGENKRLTGHDEVNRDIR 115

Query 115 GKDVMLAHDRAFERHELDPNCWTPRVLLQAAEQKSGPAKLEQIWTNMLNNDYAGGPRVG- 173

++V H F+++ L WT + + +Q G LE W ++ + G

Sbjct 116 AQEVWDFHREVFQKNGLGIENWTLDSVFKIIQQTQGDDALEAYWESLRDTQGEGMMATLL 175

Query 174 --NTSVDAISQMGWTKGG 189

T + +

Sbjct 176 NIRTMYNMHESIDSADPA 193

>GAB03280.1 hypothetical protein ACT4_059_00030 [Acinetobacter sp. NBRC 100985]

Length=2820

Score = 133 bits (335), Expect = 6e-31, Method: Composition-based stats.

Identities = 31/200 (16%), Positives = 62/200 (31%), Gaps = 12/200 (6%)

Query 1 MS---GLTERDLSVLGSYARDGNRELY-WNYLSQLPGADGYGTLALGVVRNDSLPGRVAN 56

MS LT + ++ Y + GY A GV DS+ G A

Sbjct 1 MSTNQNLTTAQIEQYQRLVQEQGINAVPGIYAALNNMGFGYAGWAYGVSTGDSVTGMGAL 60

Query 57 TYAQDYAKSQQEEGSRFPNAQLTERQWESFGQTLLERDLELRQQWMNERRPDLALNLPGK 116

+ Q A ++ + + +L L + + + +

Sbjct 61 DFMQAMASHLGT--------IISPEKVDQIRVGMLNGYLSALLEKAEANGGFVNEEIDFE 112

Query 117 DVMLAHDRAFERHELDPNCWTPRVLLQAAEQKSGPAKLEQIWTNMLNNDYAGGPRVGNTS 176

+ H + F +EL + WT ++ + G ++Q W + + G + ++

Sbjct 113 IIRDFHIQVFNNNELSIDYWTLETPMKLIGEIFGSDVVDQTWLELTETNGTGLDALFSSL 172

Query 177 VDAISQMGWTKGGQYLTRLS 196

++G YL

Sbjct 173 GLVTIVEAISQGIIYLDENG 192

>WP_100124260.1 hypothetical protein [Snodgrassella alvi]

PIT59008.1 hypothetical protein BHC57_10540 [Snodgrassella alvi]

Length=1689

Score = 133 bits (333), Expect = 1e-30, Method: Composition-based stats.

Identities = 33/197 (17%), Positives = 61/197 (31%), Gaps = 23/197 (12%)

Query 5 TERDLSVLGSYARDGNRELYWN-YLSQLPGADGYGTLALGVVRNDSLPGRVANTYAQDYA 63

+ ++ +G+ YL Y A GV D+ G A + Q A

Sbjct 8 SAAEIRAWRKQIENGDLNDVGEVYLQLAKRGYHYAKWAYGVASADTFTGNGALEFMQAVA 67

Query 64 KSQQEEGSRFPNAQLTERQWESFGQTLLERDLELRQQWMNERRPDLALNLPGKDVMLAHD 123

K LT+ + + + LE+ ++ ++ ++ + + H

Sbjct 68 KEHHH--------ILTDDETNKIRRGMALGYLEMLEKKA--GSGSVSQDITYQQMKEFHI 117

Query 124 RAFERHELDPNCWTPRVLLQAAEQKS-----------GPAKLEQIWTNMLNNDYAGGPRV 172

F+ + +D N WT + EQ + G +EQ+W M

Sbjct 118 EVFKDNHVDINYWTLYQPMAIIEQYASGKMADGKVVTGEDVVEQMWAGMWKTKGTNFD-S 176

Query 173 GNTSVDAISQMGWTKGG 189

S M + G

Sbjct 177 WFGSNKLFLIMDDAQAG 193

>WP_100117127.1 calcium-binding protein [Snodgrassella alvi]

PIT61551.1 hypothetical protein BHC56_07840 [Snodgrassella alvi]

PIT62938.1 hypothetical protein BHC47_11720 [Snodgrassella alvi]

Length=1690

Score = 133 bits (333), Expect = 1e-30, Method: Composition-based stats.

Identities = 29/183 (16%), Positives = 59/183 (32%), Gaps = 26/183 (14%)

Query 5 TERDLSVLGSYARDGNRE---LYWNYLSQLPGADGYGTLALGVVRNDSLPGRVANTYAQD 61

++ ++ +G+ + L+ Y A GV DS+ G A + Q

Sbjct 8 SKSEIEAWRKQIENGDLSDVGAVYQQLA--KRGYHYAKWAYGVASADSITGNGALEFMQA 65

Query 62 YAKSQQEEGSRFPNAQLTERQWESFGQTLLERDLELRQQWMNERRPDLALNLPGKDVMLA 121

A LT + + + L++ + ++ ++ K++

Sbjct 66 VANEHNH--------ILTADETNKIRRGMALGYLDMLAKKA--GSGSVSQDISYKEMREF 115

Query 122 HDRAFERHELDPNCWTPRVLLQAAEQKS-----------GPAKLEQIWTNMLNNDYAGGP 170

H F+ + +D N WT ++ EQ + G +EQ+W M

Sbjct 116 HIEVFKNNNVDINYWTLYEPMRIIEQYASGKLIGGREIKGEQVVEQVWEAMWTTKGTNVD 175

Query 171 RVG 173

Sbjct 176 SWL 178

>WP_075046543.1 peptidase S8 [Pseudomonas putida]

APO84660.1 peptidase S8 [Pseudomonas putida]

Length=1807

Score = 132 bits (332), Expect = 1e-30, Method: Composition-based stats.

Identities = 35/212 (17%), Positives = 68/212 (32%), Gaps = 22/212 (10%)

Query 1 MSGLTERDLSVLGSYARDGNRELYWNYLSQLPGADGYGTLALGVVRNDSLPGRVANTYAQ 60

M L E D+ ++YL+ D Y LA GV + +S+ G A + +

Sbjct 1 MFALVEGDVRRAREIFESQGPGGMYDYLA--GRGDKYALLANGVAKGNSIAGLAALEFMK 58

Query 61 DYAKSQQEEGSRFPNAQLTERQWESFGQTLLERDLELRQQWMNERRPDLALNLPGKDVML 120

+ + ER + + + L L + + + L+ K+V

Sbjct 59 RTERD--------AGRVMDERDVQLVRVEMAKAYLGLLEDKVKSDSIGVTLD--HKEVWG 108

Query 121 AHDRAFERHELDPNCWTPRVLLQAAEQKSGPAKLEQIWTNMLNNDYAGGPRVGNTSVDAI 180

H+ FE+ + WT + + P+ E W ++L++ + V + I

Sbjct 109 FHNEVFEKLGRSRDAWTLNSVFELMV----PSDRETYWRSVLDSAGSPAGEVWLS----I 160

Query 181 SQMGWTKGGQYLTRLSVLEATQALEGRSAVDP 212

+ A +D

Sbjct 161 ETDRKLSTASAIA--PQHLRPMAKYWLERIDS 190

>WP_128450905.1 calcium-binding protein [Yersinia sp. 2105 StPb PI]

RXA98421.1 calcium-binding protein [Yersinia sp. 2105 StPb PI]

Length=860

Score = 131 bits (330), Expect = 2e-30, Method: Composition-based stats.

Identities = 55/271 (20%), Positives = 96/271 (35%), Gaps = 31/271 (11%)

Query 4 LTERDL----SVLGSYARDGNRELYWNYLSQLPGADGYGTLALGVVRNDSLPGRVANTYA 59

L DL +L ++ + +++L+ D Y LA GVV+ +SL G+VA +

Sbjct 13 LNTADLARAREILEAHKESKDPSPMYDFLA--AYGDCYANLANGVVKENSLAGKVAINHL 70

Query 60 QDYAKSQQEEGSRFPNAQLTERQWESFGQTLLERDLELRQQWMNERRPDLALNLPGKDVM 119

A + + LTE Q + L L + + + N+ K

Sbjct 71 VSVATNYGK--------PLTEVQLKDLRFDLANEYLNILDNRLKASNGTIYGNIDHKQAS 122

Query 120 LAHDRAFERHELDPNCWTPRVLLQAAEQKSGPAKLEQIWTNMLNNDYAGGPRVGNTSVDA 179

L H R F ++EL WT + + + W +LN +

Sbjct 123 LLHKRVFNQYELPDKAWTLDAVFKTL----DEKEHVSYWGQVLNAAGNPAEELK------ 172

Query 180 ISQMGWTKGGQYLTRLSVLEATQALEGRSAVD--PN--VIGGNSYYAMYF-EADRKWASI 234

+S + + + Y T S T A E + VD PN I + ++ + S

Sbjct 173 LSFITYKEMVYYSTFASEDIKTIAREWFTTVDSPPNYGTITKGTINQLFNPPTEDALVSP 232

Query 235 SAGGGHMSLREITDPSRIAELNDAREVRLER 265

+S+ P + + D +VR +

Sbjct 233 PQCNTDISITP--SPQAVQRVIDEDQVRRDV 261

>WP_100126054.1 hypothetical protein [Snodgrassella alvi]

PIT30846.1 hypothetical protein BHC50_10215 [Snodgrassella alvi]

PIT32137.1 hypothetical protein BHC42_09700 [Snodgrassella alvi]

Length=1689

Score = 132 bits (331), Expect = 2e-30, Method: Composition-based stats.

Identities = 29/171 (17%), Positives = 59/171 (35%), Gaps = 26/171 (15%)

Query 5 TERDLSVLGSYARDGNRE---LYWNYLSQLPGADGYGTLALGVVRNDSLPGRVANTYAQD 61

++ ++ +G+ + L+ Y A GV DS+ G A + Q

Sbjct 8 SKSEIEAWRKQIENGDLSDVGAVYQQLA--KRGYHYAKWAYGVASADSITGNGALEFMQA 65

Query 62 YAKSQQEEGSRFPNAQLTERQWESFGQTLLERDLELRQQWMNERRPDLALNLPGKDVMLA 121

A LT + + + L++ + ++ ++ K++

Sbjct 66 VANEHNH--------ILTADETNKIRRGMALGYLDMLAKKA--GSGSVSQDISYKEMREF 115

Query 122 HDRAFERHELDPNCWTPRVLLQAAEQKS-----------GPAKLEQIWTNM 161

H F+ + +D N WT ++ EQ + G +EQ+W M

Sbjct 116 HIEVFKNNNVDINYWTLYEPMRIIEQYASGKLIGGREIKGEQVVEQVWEAM 166

>WP_084585768.1 hypothetical protein [Snodgrassella alvi]

ORF31752.1 hypothetical protein BGI09_05055 [Snodgrassella alvi]

Length=1689

Score = 131 bits (330), Expect = 3e-30, Method: Composition-based stats.

Identities = 32/224 (14%), Positives = 69/224 (31%), Gaps = 28/224 (13%)

Query 5 TERDLSVLGSYARDGNRE---LYWNYLSQLPGADGYGTLALGVVRNDSLPGRVANTYAQD 61

+ ++ +G+ + L+ Y A GV DS+ G A + Q

Sbjct 8 SRSEIQAWRKQIENGDLSDVGAVYQQLA--KRGYHYAKWAYGVASADSITGNGALEFMQA 65

Query 62 YAKSQQEEGSRFPNAQLTERQWESFGQTLLERDLELRQQWMNERRPDLALNLPGKDVMLA 121

A LT + + + L++ ++ + ++ + +

Sbjct 66 VANEHNH--------ILTADETNKIRRGMALGYLDMLEKKA--GSGSVNQDITYQQMKEF 115

Query 122 HDRAFERHELDPNCWTPRVLLQAAEQKS-----------GPAKLEQIWTNMLNNDYAGGP 170

H F+ + +D N WT ++ EQ + G +EQ+W M

Sbjct 116 HIEVFKNNNVDINYWTLYEPMRIIEQYASGKLIGGREIKGEQVVEQVWEAMWATKGTNVD 175

Query 171 RVGNTSVDAISQMGWTKGGQYLTRLSVL--EATQALEGRSAVDP 212

+ + G Y+ +L+ + ++ P

Sbjct 176 SWLMSLGLSGIMSDAKNGYIYINKLTGTPESSAAITLKAASTGP 219

>WP_100120493.1 calcium-binding protein [Snodgrassella alvi]

PIT11363.1 hypothetical protein BGI31_03410 [Snodgrassella alvi]

Length=1694

Score = 131 bits (330), Expect = 3e-30, Method: Composition-based stats.

Identities = 33/198 (17%), Positives = 66/198 (33%), Gaps = 23/198 (12%)

Query 4 LTERDLSVLGSYARDGNRELYWN-YLSQLPGADGYGTLALGVVRNDSLPGRVANTYAQDY 62

+T+ ++ + +G+ YL Y A GV DS+ G A + Q

Sbjct 7 ITKDEIRAWRNQILNGDLSDVGEVYLQLAKRGYHYAKWAYGVASADSITGNGALEFMQAV 66

Query 63 AKSQQEEGSRFPNAQLTERQWESFGQTLLERDLELRQQWMNERRPDLALNLPGKDVMLAH 122

A+ LT + + + L++ ++ ++ ++ + + H

Sbjct 67 AEEHNH--------ILTADETNKIRRYMALGYLDMLEKKA--GSGSVSQDITYQQMKEFH 116

Query 123 DRAFERHELDPNCWTPRVLLQAAEQKS-----------GPAKLEQIWTNMLNNDYAGGPR 171

F+ +++D N WT + EQ + G +EQ+W M

Sbjct 117 IEVFKINDVDINYWTLYQPMAIIEQYASGKLADGREIKGEQVVEQVWAGMWATKGTNVD- 175

Query 172 VGNTSVDAISQMGWTKGG 189

S + M + G

Sbjct 176 SWFGSNELFQIMDDVQNG 193

>WP_084548571.1 calcium-binding protein [Snodgrassella alvi]

ORF05318.1 hypothetical protein BGH98_10855 [Snodgrassella alvi]

ORF10713.1 hypothetical protein BGI01_10405 [Snodgrassella alvi]

ORF17088.1 hypothetical protein BGI03_08305 [Snodgrassella alvi]

ORF17662.1 hypothetical protein BGI04_09865 [Snodgrassella alvi]

Length=1690

Score = 131 bits (330), Expect = 3e-30, Method: Composition-based stats.

Identities = 31/206 (15%), Positives = 65/206 (32%), Gaps = 26/206 (13%)

Query 5 TERDLSVLGSYARDGNRE---LYWNYLSQLPGADGYGTLALGVVRNDSLPGRVANTYAQD 61

+ ++ +G+ + L+ Y A GV DS+ G A + Q

Sbjct 8 SRSEIQAWRKQIENGDLSDVGAVYQQLA--KRGYHYAKWAYGVASADSITGNGALEFMQA 65

Query 62 YAKSQQEEGSRFPNAQLTERQWESFGQTLLERDLELRQQWMNERRPDLALNLPGKDVMLA 121

A LT + + + L++ ++ + ++ + +

Sbjct 66 VANEHNH--------ILTADETNKIRRGMALGYLDMLEKKA--GSGSVNQDITYQQMKEF 115

Query 122 HDRAFERHELDPNCWTPRVLLQAAEQKS-----------GPAKLEQIWTNMLNNDYAGGP 170

H F+ + +D N WT ++ EQ + G +EQ+W M

Sbjct 116 HIEVFKNNNVDINYWTLYEPMRIIEQYASGKLIGGREIKGEQVVEQVWEAMWTTKGTNVD 175

Query 171 RVGNTSVDAISQMGWTKGGQYLTRLS 196

+ + G Y+ +L+

Sbjct 176 SWLMSLGLSGIMSDAKNGYIYINKLT 201

>SBW80776.1 hypothetical protein PVE_R1G2892 [Pseudomonas veronii 1YdBTEX2]

Length=752

Score = 131 bits (329), Expect = 3e-30, Method: Composition-based stats.

Identities = 25/178 (14%), Positives = 60/178 (34%), Gaps = 16/178 (9%)

Query 24 YWNYLSQLPGADGYGTLALGVVRNDSLPGRVANTYAQDYAKSQQEEGSRFPNAQLTERQW 83

++YL+ Y LA GV + D+ G A ++ + A + A +++

Sbjct 1 MYDYLAAK--GYQYAKLANGVAKGDTFAGATAISFMKMTASNIG--------APVSDADV 50

Query 84 ESFGQTLLERDLELRQQWMN-ERRPDLALNLPGKDVMLAHDRAFERHELDPNCWTPRVLL 142

+ L + ++ ++ ++ H + FE + L + WT +L

Sbjct 51 ARILNQMARGYLSALDSKLETNGGSPISSDVTYQEAWGFHTKVFEDNGLSKDAWTLNSVL 110

Query 143 QAAEQKSGPAKLEQIWTNMLNNDYAGGPRVGNTSVDAISQMGWTKGGQYLTRLSVLEA 200

E W N+L++ + +++ + M + ++

Sbjct 111 SNI----TENTRESYWQNVLDSAG-DLKKELLLAINTVQLMSLATVAGSGANKELAQS 163

>WP_078474277.1 hypothetical protein [Snodgrassella alvi]

OOX81392.1 hypothetical protein BGH94_00795 [Snodgrassella alvi]

ORF02259.1 hypothetical protein BGH95_05205 [Snodgrassella alvi]

Length=2332

Score = 131 bits (329), Expect = 4e-30, Method: Composition-based stats.

Identities = 33/226 (15%), Positives = 73/226 (32%), Gaps = 28/226 (12%)

Query 3 GLTERDLSVLGSYARDGNRE---LYWNYLSQLPGADGYGTLALGVVRNDSLPGRVANTYA 59

++ ++ + +G+ + L+ Y A GV +DS+ G A +

Sbjct 6 NVSLSEIQAWRNQIENGDLSDVGAVYQQLA--KRGYHYAKWAYGVASSDSITGNGALEFM 63

Query 60 QDYAKSQQEEGSRFPNAQLTERQWESFGQTLLERDLELRQQWMNERRPDLALNLPGKDVM 119

Q A LT + + + L++ + ++ ++ K++

Sbjct 64 QAVANEHNH--------ILTTDETNKIRRGMALGYLDILAKKA--GSDSVSQDISYKEMR 113

Query 120 LAHDRAFERHELDPNCWTPRVLLQAAEQKS-----------GPAKLEQIWTNMLNNDYAG 168

H F+ + +D N WT ++ EQ + G +E++W M

Sbjct 114 EFHIEVFKNNNVDINYWTLYEPMRIIEQYASGKPIGGHEIKGEQVVEEVWEAMWATKGTN 173

Query 169 GPRVGNTSVDAISQMGWTKGGQYLTRLSVL--EATQALEGRSAVDP 212

+ + G Y+ +L+ ++ DP

Sbjct 174 VDSWLMSLGLSGIMSDAQNGYIYINKLTGTPESIASITLKAASRDP 219

>WP_100153498.1 hypothetical protein [Snodgrassella alvi]

PIT47598.1 hypothetical protein BHC51_05145 [Snodgrassella alvi]

Length=1741

Score = 131 bits (329), Expect = 4e-30, Method: Composition-based stats.

Identities = 33/212 (16%), Positives = 71/212 (33%), Gaps = 22/212 (10%)

Query 5 TERDLSVLGSYARDGNRELYWN-YLSQLPGADGYGTLALGVVRNDSLPGRVANTYAQDYA 63

+ ++ +G+ Y Y A GV D++ G A + Q A

Sbjct 8 SAAEIRAWREQILNGDLSDVGEVYQRLAKRGYHYAQWAYGVASADTITGNGALEFMQAVA 67

Query 64 KSQQEEGSRFPNAQLTERQWESFGQTLLERDLELRQQWMNERRPDLALNLPGKDVMLAHD 123

K LT+ + + + LE+ ++ ++ ++ + + H

Sbjct 68 KEHHH--------ILTDDETNKIRRHMALGYLEMLEKKA--GSGSVSQDITYQQMKEFHI 117

Query 124 RAFERHELDPNCWTPRVLLQAAEQKS-----------GPAKLEQIWTNMLNNDYAGGPRV 172

+ F+++ +D N WT ++ E+ + G +E++W M +

Sbjct 118 KVFDKNNVDINYWTLYEPMRLIERYTSGKLIGGREIKGEQVVEEVWEAMWATEGTSVSSW 177

Query 173 GNTSVDAISQMGWTKGGQYLTRLSVLEATQAL 204

+ A G Y+ +L+ T A

Sbjct 178 AMSFGLAEIMRDAHNGYIYIDKLTGKPHTSAK 209

>WP_050096931.1 serine protease [Yersinia frederiksenii]

OVZ98944.1 serine protease [Yersinia frederiksenii]

CNH81887.1 putative serine protease [Yersinia frederiksenii]

CNI67855.1 putative serine protease [Yersinia frederiksenii]

Length=1807

Score = 131 bits (328), Expect = 5e-30, Method: Composition-based stats.

Identities = 55/271 (20%), Positives = 96/271 (35%), Gaps = 31/271 (11%)

Query 4 LTERDL----SVLGSYARDGNRELYWNYLSQLPGADGYGTLALGVVRNDSLPGRVANTYA 59

L DL +L ++ + +++L+ D Y LA GVV+ +SL G+VA +

Sbjct 13 LNTADLARAREILEAHKESKDPSPMYDFLA--AYGDCYANLANGVVKENSLAGKVAINHL 70

Query 60 QDYAKSQQEEGSRFPNAQLTERQWESFGQTLLERDLELRQQWMNERRPDLALNLPGKDVM 119

A + + LTE Q + L L + + + N+ K

Sbjct 71 VSVATNYGK--------PLTEVQLKDLRFDLANEYLNILDNRLKASNGTIYGNIDHKQAS 122

Query 120 LAHDRAFERHELDPNCWTPRVLLQAAEQKSGPAKLEQIWTNMLNNDYAGGPRVGNTSVDA 179

L H R F ++EL WT + + + W +LN +

Sbjct 123 LLHKRVFNQYELPDKAWTLDAVFKTL----DEKEHVSYWGQVLNAAGNPAEELK------ 172

Query 180 ISQMGWTKGGQYLTRLSVLEATQALEGRSAVD--PN--VIGGNSYYAMYF-EADRKWASI 234

+S + + + Y T S T A E + VD PN I + ++ + S

Sbjct 173 LSFITYKEMVYYSTFASEDIKTIAREWFTTVDSPPNYGTITKGTINQLFNPPTEDALVSP 232

Query 235 SAGGGHMSLREITDPSRIAELNDAREVRLER 265

+S+ P + + D +VR +

Sbjct 233 PQCNTDISITP--SPQAVQRVIDEDQVRRDV 261

>WP_100138955.1 hypothetical protein [Snodgrassella alvi]

PIT48793.1 hypothetical protein BHC46_03580 [Snodgrassella alvi]

Length=1536

Score = 131 bits (328), Expect = 5e-30, Method: Composition-based stats.

Identities = 31/187 (17%), Positives = 67/187 (36%), Gaps = 26/187 (14%)

Query 4 LTERDLSVLGSYARDGNRE---LYWNYLSQLPGADGYGTLALGVVRNDSLPGRVANTYAQ 60

+++ + +G+ + L++ Y + A GV D++ G A + Q

Sbjct 7 ISKSQIKAWREQIENGDLSDVGAVYQQLAE--RGYHYASWAYGVESADTITGHGALEFMQ 64

Query 61 DYAKSQQEEGSRFPNAQLTERQWESFGQTLLERDLELRQQWMNERRPDLALNLPGKDVML 120

A +Q LT+ Q + + L++ +Q + ++ ++ + +

Sbjct 65 AVATEKQH--------ILTQDQTNKIRRDMAVAYLKMLEQKASSG--SVSQDVTYQQMQE 114

Query 121 AHDRAFERHELDPNCWTPRVLLQAAEQK-----------SGPAKLEQIWTNMLNNDYAGG 169

H + FE + LD N WT ++ E+ SG +E +W M

Sbjct 115 FHIKVFENNNLDINYWTLYEPMRLIERYASAKLPNGQVLSGENIVELLWERMWKTGGTVI 174

Query 170 PRVGNTS 176

+ +

Sbjct 175 NGLSGSY 181

>WP_084554548.1 calcium-binding protein [Snodgrassella alvi]

ORF25441.1 hypothetical protein BGI07_05325 [Snodgrassella alvi]

ORF29937.1 hypothetical protein BGI10_09615 [Snodgrassella alvi]

ORF32875.1 hypothetical protein BGI11_10090 [Snodgrassella alvi]

ORF36748.1 hypothetical protein BGI13_09795 [Snodgrassella alvi]

ORF38480.1 hypothetical protein BGI14_09115 [Snodgrassella alvi]

ORF42423.1 hypothetical protein BGI15_08535 [Snodgrassella alvi]

Length=1689

Score = 131 bits (328), Expect = 6e-30, Method: Composition-based stats.

Identities = 28/183 (15%), Positives = 57/183 (31%), Gaps = 26/183 (14%)

Query 5 TERDLSVLGSYARDGNRE---LYWNYLSQLPGADGYGTLALGVVRNDSLPGRVANTYAQD 61

+ ++ +G+ + L+ Y A GV DS+ G A + Q

Sbjct 8 SRSEIQAWRKQIENGDLSDVGAVYQQLA--KRGYHYAKWAYGVASADSITGNGALEFMQA 65

Query 62 YAKSQQEEGSRFPNAQLTERQWESFGQTLLERDLELRQQWMNERRPDLALNLPGKDVMLA 121

A LT + + + L++ ++ + ++ + +

Sbjct 66 VANEHNH--------ILTADETNKIRRGMALGYLDMLEKKA--GSGSVNQDITYQQMKEF 115

Query 122 HDRAFERHELDPNCWTPRVLLQAAEQKS-----------GPAKLEQIWTNMLNNDYAGGP 170

H F+ + +D N WT ++ EQ + G +EQ+W M

Sbjct 116 HIEVFKNNNVDINYWTLYEPMRIIEQYASGKLIGGREIKGEQVVEQVWEAMWATKGTNVD 175

Query 171 RVG 173

Sbjct 176 SWL 178

>WP_083674417.1 calcium-binding protein [Burkholderia sp. GAS332]

SIO54672.1 Ca2+-binding protein, RTX toxin-related [Burkholderia sp. GAS332]

Length=1315

Score = 130 bits (326), Expect = 8e-30, Method: Composition-based stats.

Identities = 40/223 (18%), Positives = 74/223 (33%), Gaps = 20/223 (9%)

Query 4 LTERDLSVLGSYARDGNRELYWNYLSQLPGADGYGTLALGVVRNDSLPGRVANTYAQDYA 63

++ L L GN ++YLS Y LA GV ++S+ G A +

Sbjct 10 ISSVTLQNLKDNLASGNAAAVYDYLSSQ--GYPYARLAQGVNLDNSVSGTTAIEFL---- 63

Query 64 KSQQEEGSRFPNAQLTERQWESFGQTLLERDLELRQQWMNERRPDLA-LNLPGKDVMLAH 122

+ + + + L+ L++ + + L +L + H

Sbjct 64 ----GLSGENVGRPIGDAELGAIKAGLVNAYLDVLLKQAKDSPDGLTTRDLNSDEAWEIH 119

Query 123 DRAFERHELDPNCWTPRVLLQAAEQKSGPAKLEQIWTNMLNNDYAGGPRVGNTSVDAISQ 182

+ F+ H+L P+ WT A E +W +L +D + G+T

Sbjct 120 NEVFKSHDLSPDVWTLNTPFSVLN----EAARESLWQKILESDGSVL---GDTKAALDLV 172

Query 183 MGWTKG-GQYLTRLSVLEATQALEGRSAVD-PNVIGGNSYYAM 223

M T+ + Q S ++ P+ I A+

Sbjct 173 MAMTQANIDAIKTGDAATIEQIGNWASRIESPSTIAPAIGTAL 215

>WP_100150336.1 hypothetical protein [Snodgrassella alvi]

PIT51249.1 hypothetical protein BHC48_04330 [Snodgrassella alvi]

Length=1695

Score = 130 bits (326), Expect = 8e-30, Method: Composition-based stats.

Identities = 31/197 (16%), Positives = 64/197 (32%), Gaps = 23/197 (12%)

Query 5 TERDLSVLGSYARDGNRELYWN-YLSQLPGADGYGTLALGVVRNDSLPGRVANTYAQDYA 63

++ ++ + +G+ YL Y A GV DS+ G A + Q A

Sbjct 8 SKSEIQAWRNRIENGDLSDVGEVYLQLAKRGYHYAKWAYGVASADSITGNGALEFMQAVA 67

Query 64 KSQQEEGSRFPNAQLTERQWESFGQTLLERDLELRQQWMNERRPDLALNLPGKDVMLAHD 123

+ LT + + + L++ ++ ++ ++ + + H

Sbjct 68 EEHNH--------ILTTDETNKIRRYMALGYLDMLEKKA--GSGSVSQDITYQQMKEFHI 117

Query 124 RAFERHELDPNCWTPRVLLQAAEQKS-----------GPAKLEQIWTNMLNNDYAGGPRV 172

F+ + +D + WT + EQ + G +EQ+W M

Sbjct 118 EVFKANNVDIDYWTLYQPMAIIEQYASGKLADGREIKGEQVVEQVWAGMWATKGTNVD-S 176

Query 173 GNTSVDAISQMGWTKGG 189

S + M + G

Sbjct 177 WFGSNELFQIMDDVQNG 193

>WP_081921142.1 calcium-binding protein [Rhizobium sp. CF097]

Length=895

Score = 129 bits (324), Expect = 2e-29, Method: Composition-based stats.

Identities = 43/167 (26%), Positives = 66/167 (40%), Gaps = 17/167 (10%)

Query 4 LTERDLSVLGSYARDGNRELYWNYLSQLPGADGYGTLALGVVRNDSLPGRVANTYAQDYA 63

L E D L YA G+R Y++YLS+ D Y LA VV+N G VAN +A + A

Sbjct 10 LNEADFQKLEEYADAGDRISYYDYLSEK--GDSYARLANSVVQNSLFAGAVANAFASNVA 67

Query 64 KSQQEEGSRFPNAQLTERQWESFGQTLLERDLELRQQWMNERRPDLALNLPGKDVMLAHD 123

+ L+ + L++ DLE RQ + ++ H

Sbjct 68 QE-------LAGRTLSNDELAQISLALMKADLEARQLMAESGGV-----IGRVEITNYHV 115

Query 124 RAFERHELDPNCWTPRVLLQAAEQKSGPAKLEQIWTNMLNNDYAGGP 170

F +L P+ WT L AE + +W ++L+ +

Sbjct 116 AVFADFDLPPDVWTAYRPLFLAET---EQAADALWASLLDPAFYELS 159

>WP_112846424.1 hypothetical protein [Rhizobiales bacterium]

Length=1139

Score = 129 bits (323), Expect = 2e-29, Method: Composition-based stats.

Identities = 49/213 (23%), Positives = 77/213 (36%), Gaps = 22/213 (10%)

Query 1 MS-GLTERDLSVLGSYARDGNRELYWNYLSQLPGADGYGTLALGVVRNDSLPGRVANTYA 59

M+ L D +VL ++ G+R Y+ L+Q YG LALGVV ND+L G AN +

Sbjct 1 MAKALAADDFAVLEAHVAAGDRVAYYTQLAQW--GYDYGRLALGVVNNDTLAGATANIFF 58

Query 60 QDYAKSQQEEGSRFPNAQLTERQWESFGQTLLERDLELRQQWMNERRPDLALNLPGKDVM 119

D A +Q + + L++ D RQ L V

Sbjct 59 LDAAATQAQG------VTIDPDTLAQISIELMQADFAARQ---ESPLVAAGQELTYDIVQ 109

Query 120 LAHDRAFERHELDPNCWTPRVLLQAAEQKSGPAKLEQIWTNMLNNDYAGGPRV------- 172

H F R+ + + WTP + L + P + + +W M+ +

Sbjct 110 GFHQSVFARYGVSSDAWTPSLALDFLQT---PEEKQALWAEMMASSAVLSYLAVIASVFD 166

Query 173 GNTSVDAISQMGWTKGGQYLTRLSVLEATQALE 205

G T ++ +G L LS +

Sbjct 167 GTTDEAILNYIGKLSAAGGLAGLSPSNEFGNFD 199

>WP_115096921.1 hypothetical protein [Rhizobiales bacterium]

Length=1139

Score = 128 bits (322), Expect = 3e-29, Method: Composition-based stats.

Identities = 49/213 (23%), Positives = 77/213 (36%), Gaps = 22/213 (10%)

Query 1 MS-GLTERDLSVLGSYARDGNRELYWNYLSQLPGADGYGTLALGVVRNDSLPGRVANTYA 59

M+ L D +VL ++ G+R Y+ L+Q YG LALGVV ND+L G AN +

Sbjct 1 MAKALAADDFAVLEAHVAAGDRVAYYTQLAQW--GYDYGRLALGVVNNDTLAGATANIFF 58

Query 60 QDYAKSQQEEGSRFPNAQLTERQWESFGQTLLERDLELRQQWMNERRPDLALNLPGKDVM 119

D A +Q + + L++ D RQ L V

Sbjct 59 LDAAATQAQG------VTIDPDTLAQISIELMQADFAARQ---ESPLVAAGQELTYDIVQ 109

Query 120 LAHDRAFERHELDPNCWTPRVLLQAAEQKSGPAKLEQIWTNMLNNDYAGGPRV------- 172

H F R+ + + WTP + L + P + + +W M+ +

Sbjct 110 GFHQSVFARYGVSSDAWTPSLALDFLQT---PEEKQALWAEMMASSAVLSYLAVIASVFD 166

Query 173 GNTSVDAISQMGWTKGGQYLTRLSVLEATQALE 205

G T ++ +G L LS +

Sbjct 167 GTTDEAILNYIGKLSAAGGLAGLSPSNEFGNFD 199

>WP_084551678.1 calcium-binding protein [Snodgrassella alvi]

ORF27769.1 hypothetical protein BGI08_07580 [Snodgrassella alvi]

Length=1689

Score = 128 bits (322), Expect = 3e-29, Method: Composition-based stats.

Identities = 35/224 (16%), Positives = 74/224 (33%), Gaps = 28/224 (13%)

Query 5 TERDLSVLGSYARDGNRE---LYWNYLSQLPGADGYGTLALGVVRNDSLPGRVANTYAQD 61

++ ++ +G+ + L+ Y A GV DS+ G A + Q

Sbjct 8 SKSEIQAWRKQIENGDLSDVGAVYQQLA--KRGYHYAKWAYGVASADSITGNGALEFMQS 65

Query 62 YAKSQQEEGSRFPNAQLTERQWESFGQTLLERDLELRQQWMNERRPDLALNLPGKDVMLA 121

AK + LT + + + LE+ ++ ++ ++ + +

Sbjct 66 VAKERNH--------ILTTDETNKIRRGMALGYLEMLEKKA--GSGSVSQDITYQQMKEF 115

Query 122 HDRAFERHELDPNCWTPRVLLQAAEQKS-----------GPAKLEQIWTNMLNNDYAGGP 170

H + FE + +D N WT ++ EQ + G +EQ+W M

Sbjct 116 HLKVFETNNVDINYWTLYEPMRIIEQYASGKLIGGREIKGEQVVEQVWEAMWATKGTNVD 175

Query 171 RVGNTSVDAISQMGWTKGGQYLTRLSVL--EATQALEGRSAVDP 212

+ + G Y+ +L+ + ++ P

Sbjct 176 SWLMSLGLSGIMSDAKNGYIYINKLTGTPESSAAITLKAASTGP 219

>WP_100091115.1 hypothetical protein [Snodgrassella alvi]

PIT13233.1 hypothetical protein BGI33_11240 [Snodgrassella alvi]

PIT18749.1 hypothetical protein BGI34_04215 [Snodgrassella alvi]

Length=1692

Score = 128 bits (320), Expect = 6e-29, Method: Composition-based stats.

Identities = 29/204 (14%), Positives = 65/204 (32%), Gaps = 22/204 (11%)

Query 5 TERDLSVLGSYARDGNRELYWN-YLSQLPGADGYGTLALGVVRNDSLPGRVANTYAQDYA 63

+ ++ +G+ Y Y A GV D++ G A + Q A

Sbjct 8 SAAEIKAWREQILNGDLSDVGEVYQQLAKRGYHYAKWAYGVASADTITGNGALEFMQAVA 67

Query 64 KSQQEEGSRFPNAQLTERQWESFGQTLLERDLELRQQWMNERRPDLALNLPGKDVMLAHD 123

+ LT + + + L + ++ ++ ++ + + H

Sbjct 68 EEHHH--------VLTTDETNKIRRGMALGYLAMLEKKA--GSGSVSQDITYQQMKEFHI 117

Query 124 RAFERHELDPNCWTPRVLLQAAEQKS-----------GPAKLEQIWTNMLNNDYAGGPRV 172

+ F+ + +D N WT ++ EQ + G +E+ W M +

Sbjct 118 KVFKDNNVDINYWTLYEPMRIIEQYASGKLIGGREIKGEQVVEREWEAMWATEGTNVDSW 177

Query 173 GNTSVDAISQMGWTKGGQYLTRLS 196

+ + G Y+ +L+

Sbjct 178 TMSFGLSEIMRDAHNGYIYIDKLT 201

>WP_100154929.1 hypothetical protein [Snodgrassella alvi]

PIT56305.1 hypothetical protein BHC44_01200 [Snodgrassella alvi]

Length=1697

Score = 127 bits (319), Expect = 7e-29, Method: Composition-based stats.

Identities = 32/191 (17%), Positives = 62/191 (32%), Gaps = 23/191 (12%)

Query 5 TERDLSVLGSYARDGNRELYWN-YLSQLPGADGYGTLALGVVRNDSLPGRVANTYAQDYA 63

+ ++ +G+ Y Y A GV D+L G A + Q A

Sbjct 8 SAAEIKAWREQILNGDLSDVGEVYQQLAKRGYHYAKWAYGVASADTLTGNGALEFMQAVA 67

Query 64 KSQQEEGSRFPNAQLTERQWESFGQTLLERDLELRQQWMNERRPDLALNLPGKDVMLAHD 123

K LT+ + + + L++ + ++ ++ K++ H

Sbjct 68 KEHHH--------ILTDDETNKIRRYMALGYLDMLAKKA--GSGSVSQDISYKEMEEFHI 117

Query 124 RAFERHELDPNCWTPRVLLQAAEQKS-----------GPAKLEQIWTNMLNNDYAGGPRV 172

F+ + +D N WT ++ E+ + G +E +W M G

Sbjct 118 EVFKNNNVDINYWTLYEPMRMIERYAKAKLPNGKVLTGEDIVELMWERMWKTCG-TGISG 176

Query 173 GNTSVDAISQM 183

S + S M

Sbjct 177 SVASYELFSIM 187

>WP_033725382.1 peptidase S8 [Pseudomonas putida]

Length=1803

Score = 127 bits (319), Expect = 9e-29, Method: Composition-based stats.

Identities = 35/240 (15%), Positives = 70/240 (29%), Gaps = 37/240 (15%)

Query 1 MSGLTERDLSVLGSYARDGNRELYWNYLSQLPGADGYGTLALGVVRNDSLPGRVANTYAQ 60

M L+ D+ + ++ L++ D Y LA GV R +S+ G A Y +

Sbjct 1 MMKLSHSDIELASELLTKEGPSAMYDKLAEK--GDKYAVLANGVARGNSIAGVAAINYMK 58

Query 61 DYAKSQQEEGSRFPNAQLTERQWESFGQTLLERDLELRQQWMNERRPDLALNLPGKDVML 120

A + + E+ + + L + + + +P +

Sbjct 59 GVAA--------EAGMPMRDEDVEAIRFNMAKEYLNALKAHAVDGF--VLGPIPQTAIAN 108

Query 121 AHDRAFERHELDPNCWTPRVLLQAAEQKSGPAKLEQIWTNMLNNDYAGGPRVGNTSVDAI 180

H + FE + WT + + W +L++

Sbjct 109 FHKQVFEGSGYPESAWTLYPIFLGLP----EESKQIYWGLVLSSAGDPAKE--------- 155

Query 181 SQMGWTKGGQYLTRLSVLEATQALEGRSAVDPNVIGGNSYYAMYFEADRKWASISAGGGH 240

G+ +S + E VIG + + EA W +++

Sbjct 156 ----LELAGRTHAFMSQKSVLGSKE--------VIGAARKWFHHIEAPTGWFDLASTTFD 203

>WP_121085876.1 calcium-binding protein [Robbsia sp. DHC34]

RKP56661.1 calcium-binding protein [Robbsia sp. DHC34]

Length=1517

Score = 127 bits (318), Expect = 1e-28, Method: Composition-based stats.

Identities = 28/187 (15%), Positives = 51/187 (27%), Gaps = 5/187 (3%)

Query 1 MSGLTER-DLSVLGSYARDGNRELY-WNYLSQLPGADGYGTLALGVVRNDSLPGRVANTY 58

M L + L + G Y Y A GV ++ G A +

Sbjct 1 MPALIRKATLEEYRRRVQVGGLAEIERVYAELDAQGYHYAGWAKGVETGSTMTGTSALGF 60

Query 59 AQDYAKSQQEEGSRFPNAQLTERQWESFGQTLLERDLELRQQWMNERRPDLALNLPGKDV 118

+ + + + +R L L+ L V

Sbjct 61 LTETT---WRGWGMPQCEPMPPERVNKIRVDMADRYLHTLISIAEHNGNVLSGKLDFTQV 117

Query 119 MLAHDRAFERHELDPNCWTPRVLLQAAEQKSGPAKLEQIWTNMLNNDYAGGPRVGNTSVD 178

H FE + L WT + + + G +E+ W + + G + S+

Sbjct 118 REFHKEVFELNGLGIENWTLEIPMALIRKTEGDVAVEKTWARLRDTSGDGLDALAECSLL 177

Query 179 AISQMGW 185

A+ +

Sbjct 178 ALRILNL 184

>WP_100138417.1 calcium-binding protein [Snodgrassella alvi]

PIT52975.1 hypothetical protein BHC49_11970 [Snodgrassella alvi]

Length=1698

Score = 127 bits (318), Expect = 1e-28, Method: Composition-based stats.

Identities = 32/191 (17%), Positives = 62/191 (32%), Gaps = 23/191 (12%)

Query 5 TERDLSVLGSYARDGNRELYWN-YLSQLPGADGYGTLALGVVRNDSLPGRVANTYAQDYA 63

+ ++ +G+ Y Y A GV D+L G A + Q A

Sbjct 8 SAAEIKAWREQILNGDLSDVGEVYQQLAKRGYHYAKWAYGVASADTLTGNGALEFMQAVA 67

Query 64 KSQQEEGSRFPNAQLTERQWESFGQTLLERDLELRQQWMNERRPDLALNLPGKDVMLAHD 123

K LT+ + + + L++ + ++ ++ K++ H

Sbjct 68 KEHHH--------ILTDDETNKIRRYMALGYLDMLAKKA--GSGSVSQDISYKEMEEFHI 117

Query 124 RAFERHELDPNCWTPRVLLQAAEQKS-----------GPAKLEQIWTNMLNNDYAGGPRV 172

F+ + +D N WT ++ E+ + G +E +W M G

Sbjct 118 EVFKNNNVDINYWTLYEPMRMIERYAKAKLPNGKVLTGEDIVELMWERMWKTCG-TGISG 176

Query 173 GNTSVDAISQM 183

S + S M

Sbjct 177 SVASYELFSIM 187

>RKR47885.1 YD repeat-containing protein [Paraburkholderia sediminicola]

Length=847

Score = 126 bits (316), Expect = 2e-28, Method: Composition-based stats.

Identities = 44/222 (20%), Positives = 75/222 (34%), Gaps = 23/222 (10%)

Query 1 MSG-LTERDLSVLGSYARDGNRELYWNYLSQLPGADGYGTLALGVVRNDSLPGRVANTYA 59

M +T+ L L + ++G+ + YL Y LALGV N+S+ G A +

Sbjct 1 MPNQITQTQLDSLQNMVKNGDISGAYKYL--QQNGYQYAGLALGVALNNSVSGSAAIGFL 58

Query 60 QDYAKSQQEEGSRFPNAQLTERQWESFGQTLLERDLELRQQWMNERRPDLALNLPGKDVM 119

+ A LT+ Q L L Q + + ++ +

Sbjct 59 KTSA--------GLNGVNLTDDQLTQIKSALASAYLSFLQVEIKSQ-GYANQDIDWQSAW 109

Query 120 LAHDRAFERHELDPNCWTPRVLLQAAEQKSGPAKLEQIWTNMLNNDYAGGPRVGNTSVDA 179

H + F+ L + WT PA +++WT++LN+ ++ ++

Sbjct 110 DIHKQVFKNAGLPNDVWTLSTPFSVL----DPATSQKLWTDLLNDQNGTFVGTTSSILEL 165

Query 180 ISQMGWTKGGQYLTRLSVLEATQALEGRS--AVDPNVIGGNS 219

M Y Q S D NVI G +

Sbjct 166 GKDMED----AYFHTTDPAVKEQIGNWFSKITTD-NVIEGEA 202

>WP_133748256.1 hypothetical protein [Paraburkholderia sediminicola]

RKD39672.1 hypothetical protein DFJ54_9006 [Paraburkholderia sediminicola]

TDO81041.1 hypothetical protein DFJ48_02778 [Paraburkholderia sediminicola]

TDR03961.1 hypothetical protein DFH25_10063 [Paraburkholderia sediminicola]

Length=906

Score = 126 bits (316), Expect = 2e-28, Method: Composition-based stats.

Identities = 44/222 (20%), Positives = 75/222 (34%), Gaps = 23/222 (10%)

Query 1 MSG-LTERDLSVLGSYARDGNRELYWNYLSQLPGADGYGTLALGVVRNDSLPGRVANTYA 59

M +T+ L L + ++G+ + YL Y LALGV N+S+ G A +

Sbjct 1 MPNQITQTQLDSLQNMVKNGDISGAYKYL--QQNGYQYAGLALGVALNNSVSGSAAIGFL 58

Query 60 QDYAKSQQEEGSRFPNAQLTERQWESFGQTLLERDLELRQQWMNERRPDLALNLPGKDVM 119

+ A LT+ Q L L Q + + ++ +

Sbjct 59 KTSA--------GLNGVNLTDDQLAQIKSALASAYLSFLQVEIKSQ-GYANQDIDWQSAW 109

Query 120 LAHDRAFERHELDPNCWTPRVLLQAAEQKSGPAKLEQIWTNMLNNDYAGGPRVGNTSVDA 179

H + F+ L + WT PA +++WT++LN+ ++ ++

Sbjct 110 DIHKQVFKNAGLPNDVWTLSTPFSVL----DPATSQKLWTDLLNDQNGTFVGTTSSILEL 165

Query 180 ISQMGWTKGGQYLTRLSVLEATQALEGRS--AVDPNVIGGNS 219

M Y Q S D NVI G +

Sbjct 166 GKDMED----AYFHTTDPAVKEQIGNWFSKITTD-NVIEGEA 202

>WP_121109794.1 hypothetical protein [Paraburkholderia sediminicola]

RKR42457.1 hemolysin type calcium-binding protein [Paraburkholderia sediminicola]

Length=1046

Score = 126 bits (316), Expect = 2e-28, Method: Composition-based stats.

Identities = 38/227 (17%), Positives = 75/227 (33%), Gaps = 21/227 (9%)

Query 1 MSG-LTERDLSVLGSYARDGNRELYWNYLSQLPGADGYGTLALGVVRNDSLPGRVANTYA 59

M ++ L L GN +N+L Y LA GV + ++ G A +

Sbjct 1 MPNKISSSVLYNLQGNLASGNAAAVYNFLRSQ--GYRYAGLAQGVNLDSTVSGATAIGFL 58

Query 60 QDYAKSQQEEGSRFPNAQLTERQWESFGQTLLERDLELRQQWMNERRPDLA-LNLPGKDV 118

+ + + + + L++ +++ + +A +L ++

Sbjct 59 --------GLSGQVAGRPIDDTELIAIKTELVKAYVDVLIKQAENSPDGMASRDLNSQNA 110

Query 119 MLAHDRAFERHELDPNCWTPRVLLQAAEQKSGPAKLEQIWTNMLNNDYAGGPRVGNTSVD 178

H+ F H+L P+ WT A E +W +L +D + G+T

Sbjct 111 WDIHNDVFRLHDLSPDVWTLNTPFSVLN----EAARESLWQKILESDGSVL---GDTKAA 163

Query 179 AISQMGWTKG-GQYLTRLSVLEATQALEGRSAVD-PNVIGGNSYYAM 223

M T+ + Q S ++ P+ I A+

Sbjct 164 LNLVMAMTQANIDAIKTGDAATIEQIGNWASRIESPSTIAPAIGTAL 210

>WP_131265154.1 hemolysin-type calcium-binding protein [Acinetobacter sp. ANC

4910]

TCB33795.1 hemolysin-type calcium-binding protein [Acinetobacter sp. ANC

4910]

Length=2715

Score = 126 bits (316), Expect = 2e-28, Method: Composition-based stats.

Identities = 31/182 (17%), Positives = 61/182 (34%), Gaps = 13/182 (7%)

Query 3 GLTERDLSVLGSYARDGNRELYWN-YLSQLPGADGYGTLALGVVRNDSLPGRVANTYAQD 61

LT ++ + + YL+ Y A GV +L G+ A + Q

Sbjct 6 TLTLAEIIQYQVLLDEQGLQAIEAIYLALQEKGFAYAGWAYGVASGSTLTGQAALNFMQI 65

Query 62 YAKSQQEEGSRFPNAQLTERQWESFGQTLLERDLELRQQWMNERRPDLALNLPGKDVMLA 121

A +E + + ++ L + ++ +

Sbjct 66 SANE-----------NFSEEKINNIRLGMIREYLNSLNIQASNNGNYTNQDISFEVTESY 114

Query 122 HDRAFERHELDPNCWTPRVLLQAAEQKSGPAKLEQIWTNMLNNDYAGGPRVGNTSVDAIS 181

H+ F+ + L N WT + + G +KLE++W+ + + TS+D +S

Sbjct 115 HELVFKENNLSLNNWTLETPMFIIKAHLGKSKLEEVWSVIRETEGDYAD-ALLTSLDLVS 173

Query 182 QM 183

M

Sbjct 174 LM 175

>WP_096766289.1 hypothetical protein [Snodgrassella alvi]

PCL20096.1 hypothetical protein CPT77_09520 [Snodgrassella alvi]

Length=1699

Score = 125 bits (314), Expect = 3e-28, Method: Composition-based stats.

Identities = 27/176 (15%), Positives = 58/176 (33%), Gaps = 26/176 (15%)

Query 5 TERDLSVLGSYARDGNRE---LYWNYLSQLPGADGYGTLALGVVRNDSLPGRVANTYAQD 61

++ ++ +G+ + L+ Y A GV +++ G A Y Q

Sbjct 8 SKSEIEAWRKQIENGDLSDVGAVYQQLA--KRGYHYAEWAYGVASANTITGNGALEYMQT 65

Query 62 YAKSQQEEGSRFPNAQLTERQWESFGQTLLERDLELRQQWMNERRPDLALNLPGKDVMLA 121

A N LT + + + LE+ ++ + ++ + +

Sbjct 66 VANK--------NNHTLTTDEINKIRRGMALGYLEMLEKKA--GSGSVNQDITYQQMKEF 115

Query 122 HDRAFERHELDPNCWTPRVLLQAAEQKS-----------GPAKLEQIWTNMLNNDY 166

H + F+ + +D N WT ++ E + G E +W +M

Sbjct 116 HIKVFKDNNVDINYWTLYEPMRLIELYASAKLPNGQALTGEDIAELMWEHMWKTGG 171

>WP_100121666.1 calcium-binding protein [Snodgrassella alvi]

PIT28906.1 hypothetical protein BGI39_03940 [Snodgrassella alvi]

Length=1695

Score = 125 bits (314), Expect = 4e-28, Method: Composition-based stats.

Identities = 31/186 (17%), Positives = 63/186 (34%), Gaps = 26/186 (14%)

Query 5 TERDLSVLGSYARDGNRE---LYWNYLSQLPGADGYGTLALGVVRNDSLPGRVANTYAQD 61

+ ++ +G+ + L++ Y + A GV D++ G A + Q

Sbjct 8 SRNEIQAWRKQIENGDLSDVGAVYQQLAE--RGYHYASWAYGVESADTITGHGALEFMQA 65

Query 62 YAKSQQEEGSRFPNAQLTERQWESFGQTLLERDLELRQQWMNERRPDLALNLPGKDVMLA 121

A +Q LT+ Q + + L ++ N ++ ++ K++

Sbjct 66 VATEKQH--------ILTQDQTNKIRKEMAMAYLSKLEE--NAGAGSISQDISYKEMREF 115

Query 122 HDRAFERHELDPNCWTPRVLLQAAEQK-----------SGPAKLEQIWTNMLNNDYAGGP 170

H F + LD N WT ++ E+ SG +E +W M

Sbjct 116 HISVFNNNNLDINYWTLYEPMRLIERYASAKLPNGQVLSGENIVELLWERMWKTGGTVIN 175

Query 171 RVGNTS 176

+ +

Sbjct 176 GLSGSY 181

>WP_037490193.1 calcium-binding protein [Snodgrassella alvi]

KDN15714.1 Alkaline phosphatase [Snodgrassella alvi]

PIT11925.1 hypothetical protein BGI29_02435 [Snodgrassella alvi]

PIT30033.1 hypothetical protein BGI38_02795 [Snodgrassella alvi]

PIT33408.1 hypothetical protein BGI40_06925 [Snodgrassella alvi]

Length=1695

Score = 125 bits (314), Expect = 4e-28, Method: Composition-based stats.

Identities = 31/186 (17%), Positives = 63/186 (34%), Gaps = 26/186 (14%)

Query 5 TERDLSVLGSYARDGNRE---LYWNYLSQLPGADGYGTLALGVVRNDSLPGRVANTYAQD 61

+ ++ +G+ + L++ Y + A GV D++ G A + Q

Sbjct 8 SRNEIQAWRKQIENGDLSDVGAVYQQLAE--RGYHYASWAYGVESADTITGHGALEFMQA 65

Query 62 YAKSQQEEGSRFPNAQLTERQWESFGQTLLERDLELRQQWMNERRPDLALNLPGKDVMLA 121

A +Q LT+ Q + + L ++ N ++ ++ K++

Sbjct 66 VATEKQH--------ILTQDQTNKIRKEMAMAYLSKLEE--NAGAGSISQDISYKEMREF 115

Query 122 HDRAFERHELDPNCWTPRVLLQAAEQK-----------SGPAKLEQIWTNMLNNDYAGGP 170

H F + LD N WT ++ E+ SG +E +W M

Sbjct 116 HISVFNNNNLDINYWTLYEPMRLIERYASAKLPNGQVLSGENIVELLWERMWKTGGTVIN 175

Query 171 RVGNTS 176

+ +

Sbjct 176 GLSGSY 181

>RKT21632.1 hypothetical protein B0G69_5028 [Paraburkholderia sp. RAU2J]

Length=968

Score = 125 bits (313), Expect = 4e-28, Method: Composition-based stats.

Identities = 44/222 (20%), Positives = 71/222 (32%), Gaps = 22/222 (10%)

Query 1 MSG-LTERDLSVLGSYARDGNRELYWNYLSQLPGADGYGTLALGVVRNDSLPGRVANTYA 59

M L++ + L A +GN + YL Y LALGVV ++ G A +

Sbjct 1 MPNSLSKDQIGSLIQMANNGNLVGVYQYL--QQNGYQYAGLALGVVTGSTVSGAAALGFL 58

Query 60 QDYAKSQQEEGSRFPNAQLTERQWESFGQTLLERDLELRQQWMNERRPDLALNLPGKDVM 119

A+ LT Q L + L L ++ +D

Sbjct 59 NTSAQ--------INGVNLTSTQLTQIKTELTKDYLNLLLNQAVNNGGTTNTDITWQDAW 110

Query 120 LAHDRAFERHELDPNCWTPRVLLQAAEQKSGPAKLEQIWTNMLNNDYAGGPRVGNTSVDA 179

H++ F + L + WT PA +Q+W ++L + + ++

Sbjct 111 AIHNQTFYNNGLPNDVWTLNTPFSVL----DPATSQQLWNDLLKDQNGSFISTTGSILEL 166

Query 180 ISQMGWTKGGQYLTRLSVLEATQALEGRS--AVDPNVIGGNS 219

M Y + Q S D NVI G +

Sbjct 167 GKDME----TAYFNATNPSVKAQIGNWFSKITTD-NVIEGEA 203

>KES13134.1 RTX toxin or related Ca2+-binding protein [Snodgrassella alvi

SCGC AB-598-P14]

Length=1695

Score = 125 bits (313), Expect = 4e-28, Method: Composition-based stats.

Identities = 27/176 (15%), Positives = 57/176 (32%), Gaps = 26/176 (15%)

Query 5 TERDLSVLGSYARDGNRE---LYWNYLSQLPGADGYGTLALGVVRNDSLPGRVANTYAQD 61

+ ++ +G+ + L+ Y A GV +++ G A Y Q

Sbjct 8 SAAEIRAWRKQIENGDLSDVGAVYQQLA--KRGYHYAEWAYGVASANTITGNGALEYMQT 65

Query 62 YAKSQQEEGSRFPNAQLTERQWESFGQTLLERDLELRQQWMNERRPDLALNLPGKDVMLA 121

A N LT + + + LE+ ++ + ++ + +

Sbjct 66 VANK--------NNHTLTTDEINKIRRGMALGYLEMLEKKA--GSGSVNQDITYQQMKEF 115

Query 122 HDRAFERHELDPNCWTPRVLLQAAEQKS-----------GPAKLEQIWTNMLNNDY 166

H + F+ + +D N WT ++ E + G E +W +M

Sbjct 116 HIKVFKDNNVDINYWTLYEPMRLIELYASAKLPNGQALTGEDIAELMWEHMWKTGG 171

>WP_100149320.1 calcium-binding protein [Snodgrassella alvi]

PIT21682.1 hypothetical protein BGI36_06050 [Snodgrassella alvi]

Length=1694

Score = 125 bits (313), Expect = 4e-28, Method: Composition-based stats.

Identities = 31/186 (17%), Positives = 63/186 (34%), Gaps = 26/186 (14%)

Query 5 TERDLSVLGSYARDGNRE---LYWNYLSQLPGADGYGTLALGVVRNDSLPGRVANTYAQD 61

+ ++ +G+ + L++ Y + A GV D++ G A + Q

Sbjct 8 SRNEIQAWRKQIENGDLSDVGAVYQQLAE--RGYHYASWAYGVESADTITGHGALEFMQA 65

Query 62 YAKSQQEEGSRFPNAQLTERQWESFGQTLLERDLELRQQWMNERRPDLALNLPGKDVMLA 121

A +Q LT+ Q + + L ++ N ++ ++ K++

Sbjct 66 VATEKQH--------ILTQDQTNKIRKEMAMAYLSKLEE--NAGAGSISQDISYKEMREF 115

Query 122 HDRAFERHELDPNCWTPRVLLQAAEQK-----------SGPAKLEQIWTNMLNNDYAGGP 170

H F + LD N WT ++ E+ SG +E +W M

Sbjct 116 HISVFNNNNLDINYWTLYEPMRLIERYASAKLPNGQVLSGENIVELLWERMWKTGGTVIN 175

Query 171 RVGNTS 176

+ +

Sbjct 176 GLSGSY 181

>WP_084565220.1 hypothetical protein [Snodgrassella alvi]

ORF36512.1 hypothetical protein BGI12_07085 [Snodgrassella alvi]

Length=1699

Score = 124 bits (312), Expect = 7e-28, Method: Composition-based stats.

Identities = 27/176 (15%), Positives = 58/176 (33%), Gaps = 26/176 (15%)

Query 5 TERDLSVLGSYARDGNRE---LYWNYLSQLPGADGYGTLALGVVRNDSLPGRVANTYAQD 61

++ ++ +G+ + L+ Y A GV +++ G A Y Q

Sbjct 8 SKSEIQAWRKQIENGDLSDVGAVYQQLA--KRGYHYAEWAYGVASANTITGNGALEYMQT 65

Query 62 YAKSQQEEGSRFPNAQLTERQWESFGQTLLERDLELRQQWMNERRPDLALNLPGKDVMLA 121

A N LT + + + LE+ ++ + ++ + +

Sbjct 66 VANK--------NNHTLTTDEINKIRRGMALGYLEMLEKKA--GSGSVNQDITYQQMKEF 115

Query 122 HDRAFERHELDPNCWTPRVLLQAAEQKS-----------GPAKLEQIWTNMLNNDY 166

H + F+ + +D N WT ++ E + G E +W +M

Sbjct 116 HIKVFKDNNVDINYWTLYEPMRLIELYASAQLPNGQALTGEDIAELMWEHMWKTGG 171

>WP_084624593.1 hypothetical protein [Xanthomonas cassavae]

Length=1704

Score = 124 bits (312), Expect = 7e-28, Method: Composition-based stats.

Identities = 29/198 (15%), Positives = 59/198 (30%), Gaps = 18/198 (9%)

Query 3 GLTERDLSVLGSYARDGNRELYWNYLSQLPGADGYGTLALGVVRNDSLPGRVANTYAQDY 62

+ DL+ + G + + Y++ Y LA GV R + G A + ++

Sbjct 4 TMNLEDLNQARALLDAGRIDDMYAYIA--RFGHRYSRLASGVARGNMFSGLSALEFLEET 61

Query 63 AKSQQEEGSRFPNAQLTERQWESFGQTLLERDLELRQQWMNERRPDLALNLPGKDVMLAH 122

AKS A + + + L + + + H

Sbjct 62 AKS--------AGASFDPAGIQEVRKQMAGAFLTQLFTIAQNENGIVEREITADEAWNFH 113

Query 123 DRAFERHELDPNCWTPRVLLQAAEQKSGPAKLEQIWTNMLNNDYA----GGPRVGNTSVD 178

+R F L + WT + + ++W +L+++ +

Sbjct 114 NRVFTSLGLPTDSWTMNTPFELL----DEDQRNELWDVLLDSNGRWPFDSASGIRLALTV 169

Query 179 AISQMGWTKGGQYLTRLS 196

+ G K + RL

Sbjct 170 WQNSDGSGKAASWFDRLG 187

>WP_130660614.1 hypothetical protein [Rhizobium leguminosarum]

TAU35283.1 hypothetical protein ELI43_37095 [Rhizobium leguminosarum]

TBC53819.1 hypothetical protein ELH27_36740 [Rhizobium leguminosarum]

TBE60625.1 hypothetical protein ELH03_27530 [Rhizobium leguminosarum]

Length=989

Score = 124 bits (310), Expect = 1e-27, Method: Composition-based stats.

Identities = 47/209 (22%), Positives = 81/209 (39%), Gaps = 21/209 (10%)

Query 3 GLTERDLSVLGSYARDGNRELYWNYLSQLPGADGYGTLALGVVRNDSLPGRVANTYAQDY 62

LT++DL++LG+YA G+R Y+ L + YG LA+GV ND+L G AN + +

Sbjct 4 PLTDQDLALLGTYADAGDRIAYYTQL--VAWGYNYGALAMGVADNDTLSGATANIFFLNS 61

Query 63 AKSQQEEGSRFPNAQLTERQWESFGQTLLERDLELRQQWMNERRPDLALNLPGKDVMLAH 122

A +++ Q + L++ D R ++ +L V H

Sbjct 62 ASDLG--------VTISDDQLATISIRLMQADFAARTTAAHDHPG---QDLTYDVVQGYH 110

Query 123 DRAFERHELDPNCWTPRVLLQAAEQKSGPAKLEQIWTNMLNNDYAGGPRVGN----TSVD 178

F + + WTP + L + P + + +W +L G D

Sbjct 111 ADVFAGFNVPADAWTPNIALTYLQT---PEQKQALWDELLTASPVGSFAAVLQSLFGEGD 167

Query 179 AISQMGWTKGGQ-YLTRLSVLEATQALEG 206

+ Q Q Y+ L + A+ +

Sbjct 168 PLEQFASDPVIQSYIANLEIAGASATVSP 196

>RKS45304.1 LOW QUALITY PROTEIN: subtilisin-like proprotein convertase family

protein [Pseudomonas plecoglossicida]

Length=1805

Score = 123 bits (309), Expect = 1e-27, Method: Composition-based stats.

Identities = 36/221 (16%), Positives = 68/221 (31%), Gaps = 17/221 (8%)

Query 1 MSGLTERDLSVLGSYARDGNRELYWNYLSQLPGADGYGTLALGVVRNDSLPGRVANTYAQ 60

M + D+ RD + ++ L++ D Y LA GV R DSL G A Y +

Sbjct 1 MIRIELSDVDEARQALRDKGPDAMYDVLAKK--GDKYAVLANGVARGDSLSGVAAINYMK 58

Query 61 DYAKSQQEEGSRFPNAQLTERQWESFGQTLLERDLELRQQWMNERRPDLALNLPGKDVML 120

A + E + + + +E+ + +++ + + K+V

Sbjct 59 SVAAGVG--------RPMQEIDVDKVRVEMANKYMEVLLKKISKGP--IVGQVSQKEVAA 108

Query 121 AHDRAFERHELDPNCWTPRVLLQAAEQKSGPAKLEQIWTNMLNNDYAGGPRVGNTSVDAI 180

H FE E WT + E W ++L++ S

Sbjct 109 FHKETFESLEYPERAWTLSPVFDVIA----VENREDYWRHVLSSAGNLTDEF-TLSFHTH 163

Query 181 SQMGWTKGGQYLTRLSVLEATQALEGRSAVDPNVIGGNSYY 221

M + + + P++I +

Sbjct 164 RMMAMASVVSSAENRLIAKEWISRVESPTGVPDLISAAAAS 204

>WP_010955504.1 serine protease [Pseudomonas putida]

NP_747027.1 subtilase family serine protease [Pseudomonas putida KT2440]

AAN70491.1 Serine protease, subtilase family [Pseudomonas putida KT2440]

SKC14032.1 Regulatory P domain of the subtilisin-like proprotein convertase

[Pseudomonas putida]

VEE43673.1 serine protease [Pseudomonas putida]

VTQ32217.1 serine protease [Pseudomonas putida]

Length=1805

Score = 123 bits (309), Expect = 2e-27, Method: Composition-based stats.

Identities = 36/221 (16%), Positives = 68/221 (31%), Gaps = 17/221 (8%)

Query 1 MSGLTERDLSVLGSYARDGNRELYWNYLSQLPGADGYGTLALGVVRNDSLPGRVANTYAQ 60

M + D+ RD + ++ L++ D Y LA GV R DSL G A Y +

Sbjct 1 MIRIELSDVDEARQALRDKGPDAMYDVLAKK--GDKYAVLANGVARGDSLSGVAAINYMK 58

Query 61 DYAKSQQEEGSRFPNAQLTERQWESFGQTLLERDLELRQQWMNERRPDLALNLPGKDVML 120

A + E + + + +E+ + +++ + + K+V

Sbjct 59 SVAAGVG--------RPMQEIDVDKVRVEMANKYMEVLLKKISKGP--IVGQVSQKEVAA 108

Query 121 AHDRAFERHELDPNCWTPRVLLQAAEQKSGPAKLEQIWTNMLNNDYAGGPRVGNTSVDAI 180

H FE E WT + E W ++L++ S

Sbjct 109 FHKETFESLEYPERAWTLSPVFDVIA----VENREDYWRHVLSSAGNLTDEF-TLSFHTH 163

Query 181 SQMGWTKGGQYLTRLSVLEATQALEGRSAVDPNVIGGNSYY 221

M + + + P++I +

Sbjct 164 RMMAMASVVSSAENRLIAKEWISRVESPTGVPDLISAAAAS 204

>WP_080604824.1 peptidase S8 [Pseudomonas putida]

Length=1777

Score = 123 bits (308), Expect = 2e-27, Method: Composition-based stats.

Identities = 52/292 (18%), Positives = 92/292 (32%), Gaps = 31/292 (11%)

Query 24 YWNYLSQLPGADGYGTLALGVVRNDSLPGRVANTYAQDYAKSQQEEGSRFPNAQLTERQW 83

+ YL D Y LA GVV S+ G A++Y A QL++

Sbjct 1 MYEYLGSK--GDRYSKLAAGVVELKSISGIAASSYMHSVA--------GREGRQLSKADV 50

Query 84 ESFGQTLLERDLELRQQWMNERRPDLALNLPGKDVMLAHDRAFERHELDPNCWTPRVLLQ 143

ES +T+ + L+ + + + + H F+ H L WT +L+

Sbjct 51 ESIMRTMAKHYLDDTGLRLKDG--VVDREIDHLAAAAFHSETFKGHGLPDEAWTLYPVLE 108

Query 144 AAEQKSGPAKLEQIWTNMLNNDYAGGPRVGNTSVDAISQMGWTKGGQYLTR--LSVLEAT 201

++ A E++ + G V T + + + + + L R + ++

Sbjct 109 VLTKQGRAAYWEKVLDAAGDPVKEGLLTVDTTMMMSSASAMAPEPQRQLAREFIDTFDSP 168

Query 202 QALEGRSAVDPNVIGGNSYYAMYFEADRKW-----ASISAGGGHMSLREITDPSRIAELN 256

L + +FE+ W + L+ T P+ E+

Sbjct 169 SGLMSMVGT---------ASSKWFESLIDWLPSFNVETPPVEADLQLQINTTPAPRQEIL 219

Query 257 DAREVRLERLEKRTQFHPDDPYRTITRSPLTAAVDDVADPSQAPTRLADIGP 308

D V R + F + +I S T D I P

Sbjct 220 DEDRV---RDDVSNGFIQNRATHSIAFSDGTLDKTDFTSAQMGSIATGGIRP 268

>WP_123137714.1 hypothetical protein [Xanthomonas sp. CFBP 7698]

Length=2801

Score = 122 bits (306), Expect = 5e-27, Method: Composition-based stats.

Identities = 27/197 (14%), Positives = 59/197 (30%), Gaps = 18/197 (9%)

Query 4 LTERDLSVLGSYARDGNRELYWNYLSQLPGADGYGTLALGVVRNDSLPGRVANTYAQDYA 63

+ +L + G E + Y++ Y LA GV R + G A + ++ A

Sbjct 7 MNADNLVQARTLLEAGRIEEMYAYIA--AFGHRYSRLASGVARGNMFSGLSALEFLEETA 64

Query 64 KSQQEEGSRFPNAQLTERQWESFGQTLLERDLELRQQWMNERRPDLALNLPGKDVMLAHD 123

+ A + + + + E L + + ++ H+

Sbjct 65 SA--------AGAPVNSAGIQEIRRRMAEAFLGQLDIIARGNGGSVVREVTAREAWEFHN 116

Query 124 RAFERHELDPNCWTPRVLLQAAEQKSGPAKLEQIWTNMLNNDYA----GGPRVGNTSVDA 179

+ F L + WT + + ++W +L ++ +

Sbjct 117 QVFRGLNLPTDAWTMNTPFELL----DEDQRNELWDVLLESNGRWPFDSASGIRLALTVW 172

Query 180 ISQMGWTKGGQYLTRLS 196

+ G K + RL

Sbjct 173 QNSDGSGKAASWFDRLG 189

>WP_118888099.1 calcium-binding protein [Ralstonia solanacearum]

AXV71125.1 hypothetical protein CJO74_16820 [Ralstonia solanacearum]

AXV97637.1 hypothetical protein CJO80_18085 [Ralstonia solanacearum]

AXW02807.1 hypothetical protein CJO81_17940 [Ralstonia solanacearum]

AXW30296.1 hypothetical protein CJO87_17940 [Ralstonia solanacearum]

Length=1546

Score = 122 bits (305), Expect = 6e-27, Method: Composition-based stats.

Identities = 29/171 (17%), Positives = 56/171 (33%), Gaps = 11/171 (6%)

Query 27 YLSQLPGADGYGTLALGVVRNDSLPGRVANTYAQDYAKSQQEEGSRFPNAQLTERQWESF 86

YL GY GV R DS+ G A Y A L+ + +

Sbjct 5 YLYLQKQGYGYAGWGGGVAREDSIAGISAVDYLTGSALM---GMGGQACWDLSTDKSKII 61

Query 87 GQTLLERDLELRQQWMNE-----RRPDLALNLPGKDVMLAHDRAFERHELDPNCWTPRVL 141

+ + + L+ ++ E ++ ++ ++V H F+++ L WT +

Sbjct 62 KKEMAQAYLDSLEKIAGENKRLTGHDEVNRDIRAQEVWDFHREVFQKNGLGIENWTLDSV 121

Query 142 LQAAEQKSGPAKLEQIWTNMLNNDYAGGPRVG---NTSVDAISQMGWTKGG 189

+ +Q G LE W ++ + G T + +

Sbjct 122 FKIIQQTQGEDALETYWESLRDTQGEGMMATLLNIRTMYNMHESIDSADPA 172

>WP_010199167.1 HlyJ hemolysin-like protein [Psychrobacter sp. PAMC 21119]

Length=2118

Score = 120 bits (301), Expect = 2e-26, Method: Composition-based stats.

Identities = 34/215 (16%), Positives = 63/215 (29%), Gaps = 15/215 (7%)

Query 5 TERDLSVLGSYARDGNRELYWN-YLSQLPGADGYGTLALGVVRNDSLPGRVANTYAQDYA 63

T + L + + G + Y Y A GV N + G+ AN + +

Sbjct 15 TTQQLESMRNAVEAGGVDAAVAAYTELQDNGYTYAGWAKGVATNQTATGQAANGFMDNT- 73

Query 64 KSQQEEGSRFPNAQLTERQWESFGQTLLERDLELRQQWMNERRPDLALNLPGKDVMLAHD 123

+ Q + L + E + ++ D H+

Sbjct 74 ----------SAQNFSAEQVNQIKTDMAVNYLNTLIENSGETGGIITTDVDFADTRDFHE 123

Query 124 RAFERHELDPNCWTPRVLLQAAEQKSGP---AKLEQIWTNMLNNDYAGGPRVGNTSVDAI 180

+ FE H L + WT + + + GP E +W + + + G + + +

Sbjct 124 KTFEAHGLTLDNWTLKTPMDIVGEAFGPYGAQVQEAVWEQIRDTEGEGFDALTASMMLTA 183

Query 181 SQMGWTKGGQYLTRLSVLEATQALEGRSAVDPNVI 215

K Y A + L P V+

Sbjct 184 LVASEAKPDVYDLLTGDTRALEWLLATGQSLPEVV 218

>WP_085982926.1 peptidase S8 [Pseudomonas putida]

Length=1805

Score = 120 bits (301), Expect = 2e-26, Method: Composition-based stats.

Identities = 46/269 (17%), Positives = 83/269 (31%), Gaps = 22/269 (8%)

Query 1 MSGLTERDLSVLGSYARDGNRELYWNYLSQLPGADGYGTLALGVVRNDSLPGRVANTYAQ 60

M L D+ ++ L+ D Y LA GV R DS+ G A Y +

Sbjct 1 MIRLEVADIEEASVALESKGASAMYDLLAAK--GDKYAVLANGVARGDSVAGVAAINYMK 58

Query 61 DYAKSQQEEGSRFPNAQLTERQWESFGQTLLERDLELRQQWMNERRPDLALNLPGKDVML 120

A +++ ++ + L + + + + + +V

Sbjct 59 MVAAD--------AGRPMSDSDVDAIRVAMARGYLRILADKVADGP--IVEQVRQSEVSA 108

Query 121 AHDRAFERHELDPNCWTPRVLLQAAEQKSGPAKLEQIWTNMLNNDYAGGPRVGNTSVDAI 180

H F WT + + S E W +L++ G S+

Sbjct 109 FHAETFTSLGYPEKAWTLHPVFKVMADAS----KEDYWRIVLSSSGDLIDE-GLLSLRTH 163

Query 181 SQMGWTKG-GQYLTRLSVLEATQALEGRSAVDPNVI-GGNSYYAMYFEADRKWASISAGG 238

M RL E +E SAV P++I + EA +S

Sbjct 164 RMMAMASVIIDAENRLLAKEWISRVESPSAV-PDLINATANAVFNSIEASLSALMVSEPI 222

Query 239 GHMSLREITDPSR--IAELNDAREVRLER 265

+ +L+ P+ +L ++R +

Sbjct 223 PNETLKIEITPATESRRDLQGQDDIRNDV 251

>WP_111374657.1 MULTISPECIES: calcium-binding protein [Ralstonia]

AZU58152.1 hypothetical protein CFM90_15495 [Ralstonia solanacearum]

RAA11025.1 calcium-binding protein [Ralstonia pseudosolanacearum]

Length=1770

Score = 120 bits (301), Expect = 2e-26, Method: Composition-based stats.

Identities = 29/171 (17%), Positives = 56/171 (33%), Gaps = 11/171 (6%)

Query 27 YLSQLPGADGYGTLALGVVRNDSLPGRVANTYAQDYAKSQQEEGSRFPNAQLTERQWESF 86

YL GY GV R DS+ G A Y A L+ + +

Sbjct 5 YLYLQKQGYGYAGWGGGVAREDSIAGISAVDYLTGSALM---GMGGQACWDLSTDKSKII 61

Query 87 GQTLLERDLELRQQWMNE-----RRPDLALNLPGKDVMLAHDRAFERHELDPNCWTPRVL 141

+ + + L+ ++ E ++ ++ ++V H F+++ L WT +

Sbjct 62 KKEMAQAYLDSLEKIAGENKRLTGHDEVNRDIRAQEVWDFHREVFQKNGLGIENWTLDSV 121

Query 142 LQAAEQKSGPAKLEQIWTNMLNNDYAGGPRVG---NTSVDAISQMGWTKGG 189

+ +Q G LE W ++ + G T + +

Sbjct 122 FKTIQQTQGDDALEAYWESLRDTQGEGMMATLLNIRTMYNMHESIDSADPA 172

>WP_053450200.1 hypothetical protein [Stenotrophomonas maltophilia]

ALA83092.1 hypothetical protein VN11_14070 [Stenotrophomonas maltophilia]

Length=2880

Score = 120 bits (301), Expect = 2e-26, Method: Composition-based stats.

Identities = 28/202 (14%), Positives = 64/202 (32%), Gaps = 20/202 (10%)

Query 3 GLTERDLSVLGSYARDGNRELYWNYLSQLPGADGYGTLALGVVRNDSLPGRVANTYAQDY 62

+T ++ + G E + +++ Y LA GV + G A + ++

Sbjct 4 TMTAEQRAMARAMLDSGKIEEVYQFIA--GFGHRYARLASGVAVGNMFSGLTALEFLEET 61

Query 63 AKSQQEEGSRFPNAQLTERQWESFGQTLLERDLELRQQWMNERRPDLALNLPGKDVMLAH 122

AK + + + ++ + L++ + D+ + + H

Sbjct 62 AKDHG--------VPVDDAKIQAVRAGMARGLLDML-DQIANAAGDVVREITADEAWTFH 112

Query 123 DRAFERHELDPNCWTPRVLLQAAEQKSGPAKLEQIWTNMLNNDYAGGPRVGNTSVDAI-- 180

+R F L + WT + + IW +ML+++ +

Sbjct 113 NRIFTELNLPVDSWTLNTPFKLL----DEQERAAIWDSMLDSNGRLPWDTKAGIELLVEV 168

Query 181 ---SQMGWTKGGQYLTRLSVLE 199

++ G + RL E

Sbjct 169 VKNAEHDPQGAGTWFLRLGANE 190

>KDN13418.1 Alkaline phosphatase [Snodgrassella alvi]

Length=1666

Score = 120 bits (300), Expect = 2e-26, Method: Composition-based stats.

Identities = 30/164 (18%), Positives = 57/164 (35%), Gaps = 23/164 (14%)

Query 24 YWNYLSQLPGADGYGTLALGVVRNDSLPGRVANTYAQDYAKSQQEEGSRFPNAQLTERQW 83

+ L++ Y + A GV D++ G A + Q A +Q LT+ Q

Sbjct 1 MYQQLAE--RGYHYASWAYGVESADTITGHGALEFMQAVATEKQH--------ILTQDQT 50

Query 84 ESFGQTLLERDLELRQQWMNERRPDLALNLPGKDVMLAHDRAFERHELDPNCWTPRVLLQ 143

+ + L ++ N ++ ++ K++ H F + LD N WT ++

Sbjct 51 NKIRKEMAMAYLSKLEE--NAGAGSISQDISYKEMREFHISVFNNNNLDINYWTLYEPMR 108

Query 144 AAEQK-----------SGPAKLEQIWTNMLNNDYAGGPRVGNTS 176

E+ SG +E +W M + +

Sbjct 109 LIERYASAKLPNGQVLSGENIVELLWERMWKTGGTVINGLSGSY 152

>WP_112312869.1 peptidase S8 [Pseudomonas sp. URMO17WK12:I7]

RAS25118.1 subtilisin-like proprotein convertase family protein [Pseudomonas

sp. URMO17WK12:I7]

SMF04956.1 Regulatory P domain of the subtilisin-like proprotein convertase

[Pseudomonas sp. URMO17WK12:I5]

Length=1762

Score = 116 bits (289), Expect = 6e-25, Method: Composition-based stats.

Identities = 31/202 (15%), Positives = 58/202 (29%), Gaps = 26/202 (13%)

Query 24 YWNYLSQLPGADGYGTLALGVVRNDSLPGRVANTYAQDYAKSQQEEGSRFPNAQLTERQW 83

++YL+ D Y LA GV + +S+ G A + + + + + E

Sbjct 1 MYDYLASK--GDRYAVLANGVAKGNSIAGIAAIDFMKRTEQG--------ADRPMQEEDV 50

Query 84 ESFGQTLLERDLELRQQWMNERRPDLALNLPGKDVMLAHDRAFERHELDPNCWTPRVLLQ 143

E + E L + + + + L + + H FE + WT +

Sbjct 51 EQVRFKMAEAYLGVLDEKVKAG--SIGLEINHLEAWGFHSNVFEDMGRSKDAWTLNTVFN 108

Query 144 AAEQKSGPAKLEQIWTNMLNNDYAGGPRVG---------NTSVDAISQMGWTKGGQYLTR 194

E W +L + + S + + + R

Sbjct 109 LLH----EDARETYWKEVLGAAGSLPSELWLSLRTDASVAFSSAMAPEELRVQAESWKAR 164

Query 195 LSVL-EATQALEGRSAVDPNVI 215

+ L G V N I

Sbjct 165 IDSPGGVASVLLGLGTVTANGI 186

>WP_116564525.1 hypothetical protein [Paraburkholderia sp. OV555]

PVX84067.1 hypothetical protein C7513_11292 [Paraburkholderia sp. OV555]

Length=1228

Score = 116 bits (289), Expect = 7e-25, Method: Composition-based stats.

Identities = 34/174 (20%), Positives = 63/174 (36%), Gaps = 16/174 (9%)

Query 1 MSG-LTERDLSVLGSYARDGNRELYWNYLSQLPGADGYGTLALGVVRNDSLPGRVANTYA 59

M L+ L GN ++ Y+++ Y TLA GVV + G A +

Sbjct 1 MPNTLSTAQLLKAKQMISAGNLLGFYQYMAEQ--GYKYATLAEGVVSASAPNGLAALGFM 58

Query 60 QDYAKSQQEEGSRFPNAQLTERQWESFGQTLLERDLELRQQWMNERRPDLALNLPGKDVM 119

Q A +Q L++ Q + + + + +L G+ ++

Sbjct 59 QASAINQGH--------PLSDAQINKIKIDMANGWADALIGVASTN-GKVNADLTGQAIL 109

Query 120 LAHDRAFERHELDPNCWTPRVLLQAAEQKSGPAKLEQIWTNMLNNDYAGGPRVG 173

H+ F ++ LDP+ WT Q GP + ++ ++LN +

Sbjct 110 TMHNDVFLKNNLDPSTWTLHAPAQIL----GPTQFNALFQDLLNVQGNTALEIA 159

>WP_136914919.1 peptidase S8 [Pseudomonas putida]

QCI12766.1 peptidase S8 [Pseudomonas putida]

Length=1796

Score = 114 bits (284), Expect = 3e-24, Method: Composition-based stats.

Identities = 42/285 (15%), Positives = 79/285 (28%), Gaps = 18/285 (6%)

Query 24 YWNYLSQLPGADGYGTLALGVVRNDSLPGRVANTYAQDYAKSQQEEGSRFPNAQLTERQW 83

++ L++ D Y LA GVVR D++ G A + + A + E Q

Sbjct 1 MYDLLARK--GDRYAVLANGVVRGDTIAGVAAINFMKSVAAD--------AGTPMGETQV 50

Query 84 ESFGQTLLERDLELRQQWMNERRPDLALNLPGKDVMLAHDRAFERHELDPNCWTPRVLLQ 143

++ + L + + LP D+ H F + + WT +

Sbjct 51 DAVRLGMAHAYLSALNANVVAG--VVKGPLPQTDIARFHREVFVGNGYPESAWTLHSVFM 108

Query 144 AAEQKSGPAKLEQIWTNMLNNDYAGGPRVGNTSVDAISQMGWTKGGQYLTRLSVLEATQA 203

A Q S EQ+ ++ + + S + + R +

Sbjct 109 AMPQASHQTYWEQVLSSAGDLRKEVELSARTHYLMGQSSVMGPSTVRAAARDWIRRVESP 168

Query 204 LEGRSAVDPNVIGGNSYYAMYFEADRKWASISAGGGHMSLREITDPSRIAELNDAREVRL 263

R +G +++ R + S +I D +

Sbjct 169 SGWRE------LGEVGMNSLFKPILRDMPVPAESSSPSSPIKIDITPTTESRRDLQGQDD 222

Query 264 ERLEKRTQFHPDDPYRTITRSPLTAAVDDVADPSQAPTRLADIGP 308

R + + +I + T D I P

Sbjct 223 IRNDVSAGYVTRRLTHSIAFTDKTLDNTDFTSAQMGSLATGGIRP 267

>WP_062579277.1 calcium-binding protein [Rhizobium sp. Leaf391]

KQT01638.1 hypothetical protein ASG42_26890 [Rhizobium sp. Leaf391]

Length=1394

Score = 113 bits (283), Expect = 5e-24, Method: Composition-based stats.

Identities = 39/167 (23%), Positives = 58/167 (35%), Gaps = 19/167 (11%)

Query 4 LTERDLSVLGSYARDGNRELYWNYLSQLPGADGYGTLALGVVRNDSLPGRVANTYAQDYA 63

L D + L +A G+R Y++YL D Y LA VV+N G VAN +A + A

Sbjct 2 LDSYDFAKLQQHADAGDRISYYDYL--YEKGDAYARLANSVVQNSLFAGAVANAFASNVA 59

Query 64 KSQQEEGSRFPNAQLTERQWESFGQTLLERDLELRQQWMNERRPDLALNLPGKDVMLAHD 123

+ L+ L+ DL R + ++ H

Sbjct 60 LEK-------AGVTLSNEDLAQISLNLMNADLNARL-------GAAGGVIGRVEITNYHI 105

Query 124 RAFERHELDPNCWTPRVLLQAAEQKSGPAKLEQIWTNMLNNDYAGGP 170

F L P+ WT L AE +W ++LN +

Sbjct 106 ATFASFGLPPDVWTAYRPLLLAET---EDAANTLWESLLNPAFYELS 149

>WP_121106204.1 hypothetical protein [Paraburkholderia sediminicola]

Length=832

Score = 113 bits (281), Expect = 7e-24, Method: Composition-based stats.

Identities = 40/207 (19%), Positives = 68/207 (33%), Gaps = 22/207 (11%)

Query 15 YARDGNRELYWNYLSQLPGADGYGTLALGVVRNDSLPGRVANTYAQDYAKSQQEEGSRFP 74

++G+ + YL Y LALGV N+S+ G A + + A

Sbjct 1 MVKNGDISGAYKYL--QQNGYQYAGLALGVALNNSVSGSAAIGFLKTSA--------GLN 50

Query 75 NAQLTERQWESFGQTLLERDLELRQQWMNERRPDLALNLPGKDVMLAHDRAFERHELDPN 134

LT+ Q L L Q + + ++ + H + F+ L +

Sbjct 51 GVNLTDDQLTQIKSALASAYLSFLQVEIKSQ-GYANQDIDWQSAWDIHKQVFKNAGLPND 109

Query 135 CWTPRVLLQAAEQKSGPAKLEQIWTNMLNNDYAGGPRVGNTSVDAISQMGWTKGGQYLTR 194

WT PA +++WT++LN+ ++ ++ M Y

Sbjct 110 VWTLSTPFSVL----DPATSQKLWTDLLNDQNGTFVGTTSSILELGKDMED----AYFHT 161

Query 195 LSVLEATQALEGRS--AVDPNVIGGNS 219

Q S D NVI G +

Sbjct 162 TDPAVKEQIGNWFSKITTD-NVIEGEA 187

>WP_120307676.1 hypothetical protein [Paraburkholderia sediminicola]

Length=891

Score = 113 bits (281), Expect = 7e-24, Method: Composition-based stats.

Identities = 40/207 (19%), Positives = 68/207 (33%), Gaps = 22/207 (11%)

Query 15 YARDGNRELYWNYLSQLPGADGYGTLALGVVRNDSLPGRVANTYAQDYAKSQQEEGSRFP 74

++G+ + YL Y LALGV N+S+ G A + + A

Sbjct 1 MVKNGDISGAYKYL--QQNGYQYAGLALGVALNNSVSGSAAIGFLKTSA--------GLN 50

Query 75 NAQLTERQWESFGQTLLERDLELRQQWMNERRPDLALNLPGKDVMLAHDRAFERHELDPN 134

LT+ Q L L Q + + ++ + H + F+ L +

Sbjct 51 GVNLTDDQLAQIKSALASAYLSFLQVEIKSQ-GYANQDIDWQSAWDIHKQVFKNAGLPND 109

Query 135 CWTPRVLLQAAEQKSGPAKLEQIWTNMLNNDYAGGPRVGNTSVDAISQMGWTKGGQYLTR 194

WT PA +++WT++LN+ ++ ++ M Y

Sbjct 110 VWTLSTPFSVL----DPATSQKLWTDLLNDQNGTFVGTTSSILELGKDMED----AYFHT 161

Query 195 LSVLEATQALEGRS--AVDPNVIGGNS 219

Q S D NVI G +

Sbjct 162 TDPAVKEQIGNWFSKITTD-NVIEGEA 187

>WP_061373341.1 hypothetical protein [Pseudomonas stutzeri]

KXO83810.1 hypothetical protein AYK87_06580 [Pseudomonas stutzeri]

Length=3483

Score = 112 bits (280), Expect = 1e-23, Method: Composition-based stats.

Identities = 32/159 (20%), Positives = 56/159 (35%), Gaps = 15/159 (9%)

Query 4 LTERDLSVLGSYARDGNRELYWNYLSQLPGADGYGTLALGVVRNDSLPGRVANTYAQDYA 63

LT+ DL L + + G + +++ +S Y +LALG+V +S G +A Y +D A

Sbjct 2 LTQEDLVRLEALLQIGQIQEFYSQISLA--GFQYPSLALGIVVGNSKSGEIALGYMKDIA 59

Query 64 KSQQEEGSRFPNAQLTERQWESFGQTLLERDLELRQQWMNERRPDLALNLPGKDVMLAHD 123

+ + + L+ E + + + + H+

Sbjct 60 T--------ESGVYVDDALVSEIKLAMARGYLDALSVIAAED-GVVRREITWSEALAFHN 110

Query 124 RAFERHELDPNCWTPRVLLQAAEQKSGPAKLEQIWTNML 162

R F L WT V GP + W +L

Sbjct 111 RVFSEMGLPEESWTLWVPFNVL----GPESAQHSWEQIL 145

>WP_130530154.1 MULTISPECIES: hypothetical protein [Pseudoxanthomonas]

TAA09723.1 hypothetical protein EA659_09045 [Pseudoxanthomonas sp. NML171107]

TAA22899.1 hypothetical protein EA658_04880 [Pseudoxanthomonas sp. NML170316]

TAH73310.1 hypothetical protein EA657_06415 [Pseudoxanthomonas sp. NML160639]

Length=2090

Score = 112 bits (279), Expect = 1e-23, Method: Composition-based stats.

Identities = 37/201 (18%), Positives = 62/201 (31%), Gaps = 24/201 (12%)

Query 7 RDLSVLGSYARDGNRELYWNYLSQLPGADGYGTLALGVVRNDSLPGRVANTYAQDYAKSQ 66

DLS + ++YL Y LALG+V S+ G+ A + + A

Sbjct 14 ADLSHARYLLENQGPAAMYSYL--KDYGHPYSELALGLVNESSVSGQAAISNLLEVAAEN 71

Query 67 QEEGSRFPNAQLTERQWESFGQTLLERDLELRQQWMNERRPDLALNLPGKDVMLAHDRAF 126

+ ++ + LE + + ++ H R F

Sbjct 72 NVV-----------VDLNAVKYSMADAYLETLARKAERND---YSEIGAQEAWDFHSRVF 117

Query 127 -ERHELDPNCWTPRVLLQAAEQKSGPAKLEQIWTNMLNNDYAGGPRVGNTSVDAISQMGW 185

E+ EL + WT V + A+ + IW L N R S+ +

Sbjct 118 EEQFELPSSAWTMDVPFEVM----TEAERDSIWERTL-NAAGSPDREIALSLQIAAY--V 170

Query 186 TKGGQYLTRLSVLEATQALEG 206

+ L +S A A

Sbjct 171 SDKAGLLDGMSPDSAFAADMA 191

>WP_085987407.1 hypothetical protein [Pseudomonas monteilii]

Length=1819

Score = 111 bits (278), Expect = 2e-23, Method: Composition-based stats.

Identities = 48/286 (17%), Positives = 89/286 (31%), Gaps = 36/286 (13%)

Query 1 MSGLTERDLSVLGSYARDGNRELYWNYLSQLPGADGYGTLALGVVRNDSLPGRVANTYAQ 60

MS +T +D + D ++ +L Y TLA GVV + + GR A T+ +

Sbjct 1 MS-ITRQDFETAKALLDDQGPADFYAFLESK--GSRYATLAKGVVSYEGIAGRAAVTFMK 57

Query 61 DYAKSQQEEGSRFPNAQLTERQWESFGQTLLERDLELRQQWMNERRPDLALNLPGKDVML 120

+ Q E ++ E +++ + L+L + + +

Sbjct 58 ERYAEQYAE-------EMPHVILERVRRSMAKSTLDL----FEKSIGQDKFEINAGQAIA 106

Query 121 AHDRAFERHELDPNCWTPRVLLQAAEQKSGPAKLEQIWTNMLNNDYAGGPRVGNTSVDAI 180

H+R F LD + WT +A ++ E +WT L + +

Sbjct 107 VHERVFSAAGLDISYWTLDTFFKATP----VSEHEVLWTRALQLAGDFTGEMLLSGYVLS 162

Query 181 S------QMGWTKGGQYLTRLSVLEATQALEGRSAVDPNVIGGNSY-----YAMYFEADR 229

++ R++ QAL + +S A Y D

Sbjct 163 ELAAHSPSNPDADVSGWIRRVASPRMIQALSDPILDQATTLARDSLQELLNTATYVVPDP 222

Query 230 KWASISAGGGHM--SLREITDPSRI-----AELNDAREVRLERLEK 268

+ A + T+ + A ++R ER

Sbjct 223 ELALFIKNNPLPKNRTQGETEAADSVRNGYAVSEATHQIRFERGTL 268

>TBV73961.1 hypothetical protein EYC45_11055, partial [Pseudoxanthomonas

sp. NML171590]

Length=1405

Score = 111 bits (277), Expect = 2e-23, Method: Composition-based stats.

Identities = 37/201 (18%), Positives = 62/201 (31%), Gaps = 24/201 (12%)

Query 7 RDLSVLGSYARDGNRELYWNYLSQLPGADGYGTLALGVVRNDSLPGRVANTYAQDYAKSQ 66

DLS + ++YL Y LALG+V S+ G+ A + + A

Sbjct 14 ADLSHARYLLENQGPAAMYSYL--KDYGHPYSELALGLVNESSVSGQAAISNLLEVAAEN 71

Query 67 QEEGSRFPNAQLTERQWESFGQTLLERDLELRQQWMNERRPDLALNLPGKDVMLAHDRAF 126

+ ++ + LE + + ++ H R F

Sbjct 72 NVV-----------VDLNAVKYSMADAYLETLARKAERND---YSEIGAQEAWDFHSRVF 117

Query 127 -ERHELDPNCWTPRVLLQAAEQKSGPAKLEQIWTNMLNNDYAGGPRVGNTSVDAISQMGW 185

E+ EL + WT V + A+ + IW L N R S+ +

Sbjct 118 EEQFELPSSAWTMDVPFEVM----TEAERDSIWERTL-NAAGNPDREIALSLQIAAY--V 170

Query 186 TKGGQYLTRLSVLEATQALEG 206

+ L +S A A

Sbjct 171 SDKAGLLDGMSPDSAFAADMA 191

>WP_116264928.1 calcium-binding protein [Pseudomonas sp. OV081]

REF34564.1 Ca2+-binding RTX toxin-like protein [Pseudomonas sp. OV081]

Length=1509

Score = 111 bits (277), Expect = 2e-23, Method: Composition-based stats.

Identities = 43/215 (20%), Positives = 78/215 (36%), Gaps = 24/215 (11%)

Query 1 MS--GLTERDLSVLGSYARDGNRELYWNYLSQLPGADGYGTLALGVVRNDSLPGRVANTY 58

MS L + L+V + E ++ YLS + Y TLALGV +SL + Y

Sbjct 1 MSEINLHKDKLAVAENELYTKGPEAFYTYLSDM--GFHYATLALGVASENSLSAS-SIGY 57

Query 59 AQDYAKSQQEEGSRFPNAQLTERQWESFGQTLLERDLELRQQW-MNERRPDLALNLPGKD 117

++ A N LT+ + + L+ ++ + + + +

Sbjct 58 LKEAALGI--------NRTLTDDEIAEIKFGMANGYLKALKKISADNHNGPVTREINFDE 109

Query 118 VMLAHDRAFERHELDPNCWTPRVLLQAAEQKSGPAKLEQIWTNMLN---NDYAGGPRVGN 174

L H + FE L + W +A + + + W + LN N N

Sbjct 110 AELFHRQLFESKSLPSDSWIA----KATYELLTIEQRKIYWQDCLNSAGNTIEEVSFSAN 165

Query 175 TSVDAISQ---MGWTKGGQYLTRLSVLEATQALEG 206

T + ++Q + +L R+ L T+ + G

Sbjct 166 TYLYMLNQKNINHDLQSVTWLERMVSLPVTKVVTG 200

>WP_135705203.1 hypothetical protein [Cupriavidus oxalaticus]

QBY53138.1 hypothetical protein E0W60_18675 [Cupriavidus oxalaticus]

Length=835

Score = 110 bits (275), Expect = 4e-23, Method: Composition-based stats.

Identities = 28/133 (21%), Positives = 50/133 (38%), Gaps = 8/133 (6%)

Query 73 FPNAQLTERQWESFGQTLLERDLELRQQWMNERRPDLALNLPGKDVMLAHDRAFERHELD 132

L E Q + L+ + ++ ++ +V H + FE++ L

Sbjct 7 ELGKPLDEAQVNQIKFGMARGYLDTLYEQTKGGTQPVSRDINSNEVWGFHSKVFEKNGLP 66

Query 133 PNCWTPRVLLQAAEQKSGPAKLEQIWTNMLNNDYAGGPRVGNTSVDAISQMGWTKGGQYL 192

P WT + E+ GPAK+EQ W + + G DA++ +T G Y

Sbjct 67 PEAWTLDAPFRVMEKMGGPAKVEQFWNMLRD--------TGGAYGDALAANLYTLGVMYG 118

Query 193 TRLSVLEATQALE 205

S + +A+

Sbjct 119 ASASPDPSIRAMA 131

>WP_081765235.1 hypothetical protein [Robbsia andropogonis]

Length=2568

Score = 109 bits (272), Expect = 1e-22, Method: Composition-based stats.

Identities = 29/190 (15%), Positives = 60/190 (32%), Gaps = 19/190 (10%)

Query 2 SGLTERDLSVLGSYARDGNRELYWNYLSQLPGADGYGTLALGVVRNDSLPGRVANTYAQD 61

+ LT +L L + + N + + +L Y LA G+ S G+ A Y D

Sbjct 7 TPLTVEELDQLAAQVNEKNPAVLYQFLE--NNGYNYAYLAAGLTSGSSFSGQSAVRYLVD 64

Query 62 YAKSQQEEGSRFPNAQLTERQWESFGQTLLERDLELRQQWMNERRPDLALNLPGKDVMLA 121

++ + + ES + + + + ++ + ++ ++ ++ A

Sbjct 65 --------EGAKLGYEIDQAKIESIEKEMFTQYIIALEKTAVDG--VVSRDINASEMYDA 114

Query 122 HDRAFERHELDPNCWTPRVLLQAAEQKSGPAKLEQIW---TNMLNNDYAGGPRVGNTSVD 178

H+ FE L + WT G L+ W + L+N

Sbjct 115 HEIGFENQGLPISLWTLATPYTIL----GKEALQLWWSDAQSALSNSVESMYYNSKLLYQ 170

Query 179 AISQMGWTKG 188

Sbjct 171 VAEASYSLDA 180

>WP_136227885.1 tandem-95 repeat protein [Cupriavidus necator]

QCC04871.1 tandem-95 repeat protein [Cupriavidus necator H16]

Length=2367

Score = 108 bits (270), Expect = 2e-22, Method: Composition-based stats.

Identities = 19/96 (20%), Positives = 40/96 (42%), Gaps = 0/96 (0%)

Query 75 NAQLTERQWESFGQTLLERDLELRQQWMNERRPDLALNLPGKDVMLAHDRAFERHELDPN 134

LT Q E + + L+ Q + + ++ ++V H + F+ + L P+

Sbjct 9 GRPLTGAQVEKIKLDMAKGYLDALYQQTIDGSLPVTRDIDSREVWNFHRQVFKDNGLPPS 68

Query 135 CWTPRVLLQAAEQKSGPAKLEQIWTNMLNNDYAGGP 170

WT + E+ GPA++E+ W+ + +

Sbjct 69 AWTLDTPFRLMEKMGGPAQVERFWSMLRDTGGGYSD 104

>WP_083294658.1 hypothetical protein [Burkholderia plantarii]

Length=2813

Score = 107 bits (268), Expect = 4e-22, Method: Composition-based stats.

Identities = 33/201 (16%), Positives = 58/201 (29%), Gaps = 12/201 (6%)

Query 1 MSG-LTERDLSVLGSYARDGNRELYWNYLSQLPGADGYGTLALGVVRNDSLPGRVANTYA 59

M+ + L L G+ ++ Y++ L Y LA G+V G A Y

Sbjct 1 MATSMNYAQLEQLRGMISAGDLAGFYGYMANL--GYNYAYLAGGLVTGGGFSGAAAINYM 58

Query 60 QDYAKSQQEEGSRFPNAQLTERQWESFGQTLLERDLELRQQWMNERRPDLALNLPGKDVM 119

A +E Q + + L+ +Q + + +L +D +

Sbjct 59 LSVA--------HKDGVAFSEAQIPDLEKNMAFGWLDALEQVATKN-GVVDADLGYEDTL 109

Query 120 LAHDRAFERHELDPNCWTPRVLLQAAEQKSGPAKLEQIWTNMLNNDYAGGPRVGNTSVDA 179

H F++ L WT + + + N A V A

Sbjct 110 NFHVAVFKQFGLGKEAWTLTTPGEILGIPYMEGNFASLLVELGANAGADPIVSEGKLVAA 169

Query 180 ISQMGWTKGGQYLTRLSVLEA 200

+ G L + A

Sbjct 170 MQGAGADGQDFSLEDMVAANA 190

>CNC54841.1 putative serine protease [Yersinia intermedia]

CNG60366.1 putative serine protease [Yersinia intermedia]

Length=1752

Score = 107 bits (268), Expect = 4e-22, Method: Composition-based stats.

Identities = 25/199 (13%), Positives = 59/199 (30%), Gaps = 16/199 (8%)

Query 67 QEEGSRFPNAQLTERQWESFGQTLLERDLELRQQWMNERRPDL-ALNLPGKDVMLAHDRA 125

+ N +TE + + L+ +Q+ +++ + ++ K H+

Sbjct 8 WKVWGGRHNKPVTETDIKHIRYGMAHGYLDTQQKRLDDSPTGIIYGDISHKQAAQFHNSV 67

Query 126 FERHELDPNCWTPRVLLQAAEQKSGPAKLEQIWTNMLNNDYAGGPRVGNTSVDAISQMGW 185

FE H L P WT + A + S P W L+ + +

Sbjct 68 FEMHGLPPEAWTLTEVFNAMTEDSQPI----YWEQTLSTGGRPFE----ELKHSFKTYQF 119

Query 186 TKGGQYLTRLSVLEATQALEGRSAVDP----NVIGGNSYYAMYFEADRKWASISAGGGHM 241

++T + +D + +S ++ + +S +

Sbjct 120 MAYSSSFGPDDTQKST--RQWLDRMDSLPGYWALAKSSTSQLFSSDEEVAP-VSTEMCPI 176

Query 242 SLREITDPSRIAELNDARE 260

+ P + + D +

Sbjct 177 DINITPTPQAVQRIADEDQ 195

>ANH34724.1 hypothetical protein A3768_3609 [Ralstonia solanacearum]

Length=1727

Score = 105 bits (262), Expect = 2e-21, Method: Composition-based stats.

Identities = 17/130 (13%), Positives = 38/130 (29%), Gaps = 8/130 (6%)

Query 68 EEGSRFPNAQLTERQWESFGQTLLERDLELRQQWMNE-----RRPDLALNLPGKDVMLAH 122

+++ + + Q + E L E + ++ ++V H

Sbjct 1 MGMGGEACWNISQWKSDKIKQEMAEAYLNRLDTIAQENKRLTGNYEAGRDIQAQEVWDFH 60

Query 123 DRAFERHELDPNCWTPRVLLQAAEQKSGPAKLEQIWTNMLNNDYAGGPRVG---NTSVDA 179

F+ + L WT + + +Q G LE W ++ + G T +

Sbjct 61 KEVFKNNGLGIENWTLDSVFKIIQQTQGDDALEAYWESLRDTQGEGPMATLLNIRTMYNM 120

Query 180 ISQMGWTKGG 189

+

Sbjct 121 HESIDSADPA 130

>MBM63543.1 hypothetical protein [Acidobacteria bacterium]

Length=1058

Score = 104 bits (260), Expect = 4e-21, Method: Composition-based stats.

Identities = 48/209 (23%), Positives = 72/209 (34%), Gaps = 30/209 (14%)

Query 1 MSG-LTERDLSVLGSYARD-----GNRELYWNYLSQLPGADGYGTLALGVVRNDSLPGRV 54

MS L ++ + L + G R Y+ L+ YG LA GVV N+ GR+

Sbjct 1 MSAFLNQQRIDQLQTIVDQLDTDPGARADYYQQLADW--GFQYGNLAKGVVTNERFAGRI 58

Query 55 ANTYAQDYAKSQQEEGSRFPNAQLTERQWESFGQTLLERDLELRQQWM-----NERRPDL 109

AN + NA++T Q Q L+E+DL RQ + +

Sbjct 59 ANAFID-------------ANAEITSEQSLGISQGLIEQDLAARQTLLLEAIDDNGPDAT 105

Query 110 ALNLPGKDVMLAHDRAFER-HELDPNCWTPRVLLQAAEQKSGPAKLEQ-IWTNMLNNDYA 167

+LPG + H F L P WT A E +W LN +

Sbjct 106 IGDLPGGAIRDYHVVVFNGVAGLPPEAWTAYTPTVLARDGYLVGWTEAEVWDAFLNEEAY 165

Query 168 GGPRVGNTSVDAISQMGWT--KGGQYLTR 194

+ +D + + + YL

Sbjct 166 PEEDNLSDFLDDSAIIATMLQQYAAYLVN 194

>WP_082511674.1 hypothetical protein [Devosia sp. Leaf420]

Length=766

Score = 104 bits (258), Expect = 6e-21, Method: Composition-based stats.

Identities = 44/196 (22%), Positives = 70/196 (36%), Gaps = 25/196 (13%)

Query 4 LTERDLSVLGSYARDGNRELYWNYLSQLPGADGYGTLALGVVRNDSLPGRVANTYAQDYA 63

L+ DL +L S+A G+R Y++ LS YG LALGVV ++L GR AN +

Sbjct 18 LSSSDLDLLKSHADAGDRVAYYSQLS--AWGYAYGALALGVVTQETLSGRTANRFF---- 71

Query 64 KSQQEEGSRFPNAQLTERQWESFGQTLLERDLELRQQWMNERRPDLALNLPGKDVMLAHD 123

+ + L+ DLE R+ ++ + H

Sbjct 72 --------INQSGVFEPDDLATVSLALMNFDLEARRDANGN-------DIGYHAISEYHR 116

Query 124 RAFERHELDPNCWTPRVLLQAAEQKSGPAKLEQIWTNMLNNDYAGGPRVGNTSVDAISQM 183

AFE + + WTP L + +W ++L + + +

Sbjct 117 EAFELVGANIDAWTPTRALN----ELDEEDRGALWISLLTSGELSSGASLLQPLPWLELN 172

Query 184 GWTKGGQYLTRLSVLE 199

G Q L RL+

Sbjct 173 PNETGAQALERLAQYA 188

>PIE08497.1 hypothetical protein CSA74_02040 [Rhodobacterales bacterium]

Length=879

Score = 96.8 bits (239), Expect = 2e-18, Method: Composition-based stats.

Identities = 37/171 (22%), Positives = 60/171 (35%), Gaps = 23/171 (13%)

Query 9 LSVLGSYARDGNRELYWNYLSQLPGADGYGTLALGVVRNDSLPGRVANTYAQDYAKSQQE 68

+S+L ++ G+R Y+ + YG LALGVV N+S+ G AN +

Sbjct 1 MSILKAHVEAGDRIAYYT--ALDSFGVAYGGLALGVVLNNSISGASANGFLVAQGLDGVA 58

Query 69 EGSRFPNAQLTERQWESFGQTLLERDLELRQQWMNERRPDLALNLPGKDVMLAHDRAFER 128

G L+E D + R+ +L G + H R FE+

Sbjct 59 ----------NTDLLARIGTELMEADYDARKAANGA-------DLEGTVISNYHQRVFEK 101

Query 129 -HELDPNCWTPRVLLQAAEQKSGPAKLEQIWTNMLNNDYAGGPRVGNTSVD 178

P WTP + L + P W ++ + + +D

Sbjct 102 LAGAGPEAWTPYIYLDTFDS---PEAFNAAWEELVGAGNVETWELISDRLD 149

>WP_049842140.1 calcium-binding protein, partial [Ralstonia solanacearum]

Length=854

Score = 96.4 bits (238), Expect = 3e-18, Method: Composition-based stats.

Identities = 16/108 (15%), Positives = 37/108 (34%), Gaps = 8/108 (7%)

Query 90 LLERDLELRQQWMNERRP-----DLALNLPGKDVMLAHDRAFERHELDPNCWTPRVLLQA 144

+ + L+ + ++ ++ K+V H++ FE++ L WT + +

Sbjct 1 MAQAYLDALESIAERNYQLTNKYEVDRDISAKEVWNFHEKVFEKNGLGIENWTLDSVFKT 60

Query 145 AEQKSGPAKLEQIWTNMLNNDYAGGPRVG---NTSVDAISQMGWTKGG 189

+Q G LE W ++ + G T + +

Sbjct 61 IQQTQGDDALEAYWESLRDTQGEGMMATLLNIRTMYNMHESIDSADPA 108

>WP_049832774.1 calcium-binding protein, partial [Ralstonia solanacearum]

Length=866

Score = 96.4 bits (238), Expect = 3e-18, Method: Composition-based stats.

Identities = 16/108 (15%), Positives = 37/108 (34%), Gaps = 8/108 (7%)

Query 90 LLERDLELRQQWMNERRP-----DLALNLPGKDVMLAHDRAFERHELDPNCWTPRVLLQA 144

+ + L+ + ++ ++ K+V H++ FE++ L WT + +

Sbjct 1 MAQAYLDALESIAERNYQLTNKYEVDRDISAKEVWNFHEKVFEKNGLGIENWTLDSVFKT 60

Query 145 AEQKSGPAKLEQIWTNMLNNDYAGGPRVG---NTSVDAISQMGWTKGG 189

+Q G LE W ++ + G T + +

Sbjct 61 IQQTQGDDALEAYWESLRDTQGEGMMATLLNIRTMYNMHESIDSADPA 108

>CAD13632.1 putative hemolysin-type calcium-binding protein [Ralstonia solanacearum

GMI1000]

Length=960

Score = 96.1 bits (237), Expect = 3e-18, Method: Composition-based stats.

Identities = 17/108 (16%), Positives = 37/108 (34%), Gaps = 8/108 (7%)

Query 90 LLERDLELRQQWMNERR-----PDLALNLPGKDVMLAHDRAFERHELDPNCWTPRVLLQA 144

+ L+ ++ E R ++ ++ K+V H F+++ L WT + +

Sbjct 1 MARAYLDTLEKIAEENRKLTGNDEVNRDIQAKEVWDFHREVFQKNGLGVENWTLDSVFKT 60

Query 145 AEQKSGPAKLEQIWTNMLNNDYAGGPRVG---NTSVDAISQMGWTKGG 189

+Q G LE W ++ + G T + +

Sbjct 61 IQQTQGDDALEAYWESLRDTQGEGMMATLLNIRTMYNMHESIDSADPA 108

>WP_038962153.1 calcium-binding protein, partial [Ralstonia solanacearum]

Length=884

Score = 96.1 bits (237), Expect = 4e-18, Method: Composition-based stats.

Identities = 15/108 (14%), Positives = 37/108 (34%), Gaps = 8/108 (7%)

Query 90 LLERDLELRQQWMNE-----RRPDLALNLPGKDVMLAHDRAFERHELDPNCWTPRVLLQA 144

+ + L+ ++ E ++ ++ ++V H F+++ L WT + +

Sbjct 1 MAQAYLDSLEKIAGENKRLTGHDEVNRDIRAQEVWDFHREVFQKNGLGIENWTLDSVFKI 60

Query 145 AEQKSGPAKLEQIWTNMLNNDYAGGPRVG---NTSVDAISQMGWTKGG 189

+Q G LE W ++ + G T + +

Sbjct 61 IQQTQGEDALETYWESLRDTQGEGMMATLLNIRTMYNMHESIDSADPA 108

>WP_071011979.1 calcium-binding protein, partial [Ralstonia solanacearum]

OHV01570.1 hemolysin, partial [Ralstonia solanacearum]

Length=934

Score = 95.7 bits (236), Expect = 5e-18, Method: Composition-based stats.

Identities = 15/108 (14%), Positives = 37/108 (34%), Gaps = 8/108 (7%)

Query 90 LLERDLELRQQWMNE-----RRPDLALNLPGKDVMLAHDRAFERHELDPNCWTPRVLLQA 144

+ + L+ ++ E ++ ++ ++V H F+++ L WT + +

Sbjct 1 MAQAYLDSLEKIAGENKRLTGHDEVNRDIRAQEVWDFHREVFQKNGLGIENWTLDSVFKI 60

Query 145 AEQKSGPAKLEQIWTNMLNNDYAGGPRVG---NTSVDAISQMGWTKGG 189

+Q G LE W ++ + G T + +

Sbjct 61 IQQTQGEDALETYWESLRDTQGEGMMATLLNIRTMYNMHESIDSADPA 108

>WP_071624011.1 calcium-binding protein, partial [Ralstonia solanacearum]

OIT10551.1 hemolysin, partial [Ralstonia solanacearum]

Length=883

Score = 95.3 bits (235), Expect = 6e-18, Method: Composition-based stats.

Identities = 14/108 (13%), Positives = 34/108 (31%), Gaps = 8/108 (7%)

Query 90 LLERDLELRQQWMNE-----RRPDLALNLPGKDVMLAHDRAFERHELDPNCWTPRVLLQA 144

+ + L + + + ++ ++V H + F+ + L WT + +

Sbjct 1 MADAYLNTLEAIAEKNLKNTGEYEANRDINAEEVWDFHKKVFKDNGLGIENWTLDSVFKT 60

Query 145 AEQKSGPAKLEQIWTNMLNNDYAGGPRVG---NTSVDAISQMGWTKGG 189

+Q G LE W ++ + G T + +

Sbjct 61 IQQTQGDDALEAYWESLRDTQGEGMMATLLNIRTMYNMHESIDSADPA 108

>WP_081357709.1 hypothetical protein [Ralstonia solanacearum]

Length=1546

Score = 95.3 bits (235), Expect = 9e-18, Method: Composition-based stats.

Identities = 16/108 (15%), Positives = 37/108 (34%), Gaps = 8/108 (7%)

Query 90 LLERDLELRQQWMNERRP-----DLALNLPGKDVMLAHDRAFERHELDPNCWTPRVLLQA 144

+ + L+ + ++ ++ K+V H++ FE++ L WT + +

Sbjct 1 MAQAYLDALESIAERNYQLTNKYEVDRDISAKEVWNFHEKVFEKNGLGIENWTLDSVFKT 60

Query 145 AEQKSGPAKLEQIWTNMLNNDYAGGPRVG---NTSVDAISQMGWTKGG 189

+Q G LE W ++ + G T + +

Sbjct 61 IQQTQGDDALEAYWESLRDTQGEGPMATLLNIRTMYNMHESIDSADPA 108

>AFV98791.1 alkaline phosphatase [Candidatus Snodgrassella sp. TA7_36335]

Length=1532

Score = 94.5 bits (233), Expect = 2e-17, Method: Composition-based stats.

Identities = 18/129 (14%), Positives = 42/129 (33%), Gaps = 21/129 (16%)

Query 56 NTYAQDYAKSQQEEGSRFPNAQLTERQWESFGQTLLERDLELRQQWMNERRPDLALNLPG 115

+ Q AK +L + + +S + + L + Q +N+ + ++

Sbjct 1 MQFMQAVAKDH--------KQKLPQARVDSVRRDMALGYLAMLQTKLNKGKGG--EDITY 50

Query 116 KDVMLAHDRAFERHELDPNCWTPRVLLQAAEQK-----------SGPAKLEQIWTNMLNN 164

++++ H F ++LD WT + + G +E +W +M

Sbjct 51 EEMLEFHVEVFNNNKLDIGYWTLYTPMSIIQNHASATGSNGQEIGGEQVVENMWQHMQAT 110

Query 165 DYAGGPRVG 173

Sbjct 111 KGTKLKGSW 119

>WP_118872512.1 calcium-binding protein [Ralstonia solanacearum]

AXW40115.1 hypothetical protein CJO89_17090 [Ralstonia solanacearum]

AXW72904.1 hypothetical protein CJO96_16445 [Ralstonia solanacearum]

Length=1929

Score = 94.1 bits (232), Expect = 2e-17, Method: Composition-based stats.

Identities = 14/108 (13%), Positives = 34/108 (31%), Gaps = 8/108 (7%)

Query 90 LLERDLELRQQWMNE-----RRPDLALNLPGKDVMLAHDRAFERHELDPNCWTPRVLLQA 144

+ + L + + + ++ ++V H + F+ + L WT + +

Sbjct 1 MADAYLNTLEAIAEKNLKNTGEYEANRDINAEEVWDFHKKVFKDNGLGIENWTLDSVFKT 60

Query 145 AEQKSGPAKLEQIWTNMLNNDYAGGPRVG---NTSVDAISQMGWTKGG 189

+Q G LE W ++ + G T + +

Sbjct 61 IQQTQGDDALEAYWESLRDTQGEGMMATLLNIRTMYNMHESIDSADPA 108

>AKZ27857.1 hypothetical protein ACH51_00510 [Ralstonia solanacearum]

Length=1929

Score = 94.1 bits (232), Expect = 2e-17, Method: Composition-based stats.

Identities = 14/108 (13%), Positives = 34/108 (31%), Gaps = 8/108 (7%)

Query 90 LLERDLELRQQWMNE-----RRPDLALNLPGKDVMLAHDRAFERHELDPNCWTPRVLLQA 144

+ + L + + + ++ ++V H + F+ + L WT + +

Sbjct 1 MADAYLNTLEAIAEKNLKNTGEYEANRDINAEEVWDFHKKVFKDNGLGIENWTLDSVFKT 60

Query 145 AEQKSGPAKLEQIWTNMLNNDYAGGPRVG---NTSVDAISQMGWTKGG 189

+Q G LE W ++ + G T + +

Sbjct 61 IQQTQGDDALEAYWESLRDTQGEGMMATLLNIRTMYNMHESIDSADPA 108

>OAK93227.1 hypothetical protein AB851_00555, partial [Ralstonia solanacearum]

Length=930

Score = 91.4 bits (225), Expect = 1e-16, Method: Composition-based stats.

Identities = 16/108 (15%), Positives = 32/108 (30%), Gaps = 8/108 (7%)

Query 90 LLERDLELRQQWMNE-----RRPDLALNLPGKDVMLAHDRAFERHELDPNCWTPRVLLQA 144

+ E L E + ++ ++V H F+ + L WT + +

Sbjct 1 MAEAYLNRLDTIAQENKRLTGNYEAGRDIQAQEVWDFHKEVFKNNGLGIENWTLDSVFKI 60

Query 145 AEQKSGPAKLEQIWTNMLNNDYAGGPRVG---NTSVDAISQMGWTKGG 189

+Q G LE W ++ + G T + +

Sbjct 61 IQQTQGDDALEAYWESLRDTQGEGMMATLLNIRTMYNMHESIDSADPA 108

>WP_003272661.1 calcium-binding protein [Ralstonia solanacearum]

OYQ13208.1 hemolysin [Ralstonia solanacearum]

CCF98887.1 Putative hemolysin-type calcium-binding protein (modular protein)

[Ralstonia solanacearum K60-1]

Length=1258

Score = 90.7 bits (223), Expect = 3e-16, Method: Composition-based stats.

Identities = 16/108 (15%), Positives = 32/108 (30%), Gaps = 8/108 (7%)

Query 90 LLERDLELRQQWMNE-----RRPDLALNLPGKDVMLAHDRAFERHELDPNCWTPRVLLQA 144

+ E L E + ++ ++V H F+ + L WT + +

Sbjct 1 MAEAYLNRLDTIAQENKRLTGNYEAGRDIRAQEVWDFHKEVFKNNGLGIENWTLDSVFKI 60

Query 145 AEQKSGPAKLEQIWTNMLNNDYAGGPRVGNT---SVDAISQMGWTKGG 189

+Q G LE W ++ + G + + T

Sbjct 61 IQQTQGDDALEAYWESLRDTKGEGVTATLQNILTMYNMHGAIDSTDPA 108

>WP_086005414.1 calcium-binding protein [Ralstonia solanacearum]

Length=1705

Score = 90.3 bits (222), Expect = 4e-16, Method: Composition-based stats.

Identities = 16/108 (15%), Positives = 32/108 (30%), Gaps = 8/108 (7%)

Query 90 LLERDLELRQQWMNE-----RRPDLALNLPGKDVMLAHDRAFERHELDPNCWTPRVLLQA 144

+ E L E + ++ ++V H F+ + L WT + +

Sbjct 1 MAEAYLNRLDTIAQENKRLTGNYEAGRDIQAQEVWDFHKEVFKNNGLGIENWTLDSVFKI 60

Query 145 AEQKSGPAKLEQIWTNMLNNDYAGGPRVG---NTSVDAISQMGWTKGG 189

+Q G LE W ++ + G T + +

Sbjct 61 IQQTQGDDALEAYWESLRDTQGEGPMATLLNIRTMYNMHESIDSADPA 108

>PPR16777.1 hypothetical protein CFH43_00789, partial [Proteobacteria bacterium]

Length=1020

Score = 87.6 bits (215), Expect = 3e-15, Method: Composition-based stats.

Identities = 31/189 (16%), Positives = 61/189 (32%), Gaps = 22/189 (12%)

Query 33 GADGYGTLALGVVRNDSLPGRVANTYAQDYAKSQQEEGSRFPNAQLTERQWESFGQTLLE 92

D YG L + + G AN + + A + E W++ L +

Sbjct 1 YEDPYGRLGKEISEDKGWQGEFANDFLESGASDSGKNFKA------GEDDWKALNLALAQ 54

Query 93 RDLELRQQWMNERRPDLALNLPGKDVMLAHDRAFERHELDPNCWTPRVLLQAAEQKSGPA 152

R L+ + N + N + V H+ +E EL + W P +L ++ A

Sbjct 55 RYLDAYKANQNTDGTYDSPNW--EQVQDFHNEEYEDAELSADDWFPNKMLD---DQASDA 109

Query 153 KLEQIWTNMLNNDYAG---------GPRVGNTSVDAISQMGWT--KGGQYLTRLSVLEAT 201

+ + +W + L N+ + +D + +L ++

Sbjct 110 ERDAMWQDFLVNESISDLMEDALDVLDAAIPSDMDVFNYYDAIKKDPEGFLAQIDASNEF 169

Query 202 QALEGRSAV 210

G + V

Sbjct 170 AKNTGAAIV 178

>PCJ99994.1 hypothetical protein COA45_04050 [Zetaproteobacteria bacterium]

Length=1756

Score = 86.0 bits (211), Expect = 1e-14, Method: Composition-based stats.

Identities = 35/165 (21%), Positives = 63/165 (38%), Gaps = 17/165 (10%)

Query 1 MSGLTERDLSVLGSYARDG--NRELYWNYLSQLPGADG------YGTLALGVVRNDSLPG 52

M + + S A G + +Y+ ++ A G Y LA VVR+D++ G

Sbjct 9 MENIADITYEAFVSLADQGYNSAAVYYQAMALAAEASGNDSVEDYANLAESVVRDDNVNG 68

Query 53 RVANTYAQDYAKSQQEEGSRFPNAQLTERQWESFG--QTLLERDLELRQQWMNERRPDLA 110

++AN Y + A++ E + L++ DL+ RQ + +

Sbjct 69 QLANNYTEAVAEANNVNFDDSTA----EGRANRLRMQYELMQNDLKERQDNILDGG---T 121

Query 111 LNLPGKDVMLAHDRAFERHELDPNCWTPRVLLQAAEQKSGPAKLE 155

L +D H+ A +R L P ++ + L + A E

Sbjct 122 GELNYEDTNNIHEEALDRIGLPPEAFSLYIPLSQTAEHDPAAAQE 166

>WP_126585206.1 calcium-binding protein [Pseudomonas veronii]

RTY70755.1 calcium-binding protein [Pseudomonas veronii]

Length=1912

Score = 85.3 bits (209), Expect = 2e-14, Method: Composition-based stats.

Identities = 13/119 (11%), Positives = 36/119 (30%), Gaps = 6/119 (5%)

Query 83 WESFGQTLLERDLELRQQWMN-ERRPDLALNLPGKDVMLAHDRAFERHELDPNCWTPRVL 141

+ L + ++ ++ ++ H + FE + L + WT +

Sbjct 1 MARILNQMARGYLSALDSKLETNGGSPISSDVTHREAWGFHTKVFEDNGLSKDAWTLNSV 60

Query 142 LQAAEQKSGPAKLEQIWTNMLNNDYAGGPRVGNTSVDAISQMGWTKGGQYLTRLSVLEA 200

L E W N+L++ + +++ + M + ++

Sbjct 61 LSNI----TENTRESYWQNVLDSAG-DIKKELLLAINTVQLMSLATVAGSGANKELAQS 114

>PZQ34321.1 hypothetical protein DI562_00325 [Stenotrophomonas acidaminiphila]

Length=1449

Score = 85.3 bits (209), Expect = 2e-14, Method: Composition-based stats.

Identities = 26/121 (21%), Positives = 40/121 (33%), Gaps = 5/121 (4%)

Query 13 GSYARDGNRELYWNYLSQLPGADGYGTLALGVVRNDSLPGRVANTYAQDYAKSQQEEGSR 72

+YA GN LY+ + G D Y A V N + NT + + +E +

Sbjct 1306 QAYADAGNWSLYYQ--AAAAGGDKYAARAFEVASNIGFLSNITNTRLSNSILERTKEKTC 1363

Query 73 FPNAQLTERQWESFGQTLLERDLELRQQWMNERRPDLALNLPGKDVMLAHDRAFERHELD 132

E++ E+ L + + L DV HDR F +

Sbjct 1364 EAARADMEKKMEAIRVGLARAHANALKGATRDNPRMLDR---VTDVGGFHDRVFAENGAG 1420

Query 133 P 133

P

Sbjct 1421 P 1421

>WP_027237585.1 calcium-binding protein [Leisingera caerulea]

Length=1763

Score = 83.7 bits (205), Expect = 6e-14, Method: Composition-based stats.

Identities = 41/223 (18%), Positives = 75/223 (34%), Gaps = 18/223 (8%)

Query 1 MSGLTERDLSVLGSYARDGNRELYWNYLSQLPGADG------YGTLALGVVRNDSLPGRV 54

++ LT D L E+Y+ L G Y +A GVV+N + G++

Sbjct 12 LANLTFADFKAL-HLQNQNRAEVYYTELRIQGQNSGITSIENYANIAFGVVQNSTTNGQM 70

Query 55 ANTYAQDYAKSQQEEGSRFPNAQLTERQWESFGQTLLERDLELRQQWMNERRPDLALNLP 114

AN + +A+ P ++ + TL++ DL R +N D L+

Sbjct 71 ANYFTAAFAEVATPVVDFSPG---SDARLL-MQYTLMQEDLAARLNNINNLGGDGELDFS 126

Query 115 GKDVMLAHDRAFERHELDPNCWTPRVLLQAAEQKSGPAKLEQIWTNMLNNDY----AGGP 170

+ H+ A + L P + L + PAK + + +++ G

Sbjct 127 VTNT--IHENALDAIGLGPEAFALYTPLS-ILAEHDPAKAQNYFEAAIDSTGFLDTVGQG 183

Query 171 RVGNTSVDAISQMGWTKGGQYLTRLSVLEATQALEGRSAVDPN 213

+ D S + + + A +D N

Sbjct 184 LLLGFGADISSAQSLSDILKDYGDQAEWLAASLEAMEDWIDAN 226

>QCG67373.1 hypothetical protein E4167_23705 [Pseudomonas veronii]

Length=1905

Score = 83.3 bits (204), Expect = 8e-14, Method: Composition-based stats.

Identities = 13/112 (12%), Positives = 36/112 (32%), Gaps = 6/112 (5%)

Query 90 LLERDLELRQQWMN-ERRPDLALNLPGKDVMLAHDRAFERHELDPNCWTPRVLLQAAEQK 148

+ L + ++ ++ ++ H + FE + L + WT +L

Sbjct 1 MARGYLSALDSKLETNGGSPISSDVTHQEAWGFHTKVFEDNGLSKDAWTLNSVLSNI--- 57

Query 149 SGPAKLEQIWTNMLNNDYAGGPRVGNTSVDAISQMGWTKGGQYLTRLSVLEA 200

E W N+L++ + +++ + M + ++

Sbjct 58 -TENTRESYWQNVLDSAG-DIKKELLLAINTVQLMSLATVAGSGANKELAQS 107

>WP_084584981.1 calcium-binding protein [Snodgrassella alvi]

ORF34260.1 hypothetical protein BGI09_01200 [Snodgrassella alvi]

Length=1499

Score = 79.9 bits (195), Expect = 1e-12, Method: Composition-based stats.

Identities = 18/126 (14%), Positives = 45/126 (36%), Gaps = 14/126 (11%)

Query 90 LLERDLELRQQWMNERRPDLALNLPGKDVMLAHDRAFERHELDPNCWTPRVLLQAAEQKS 149

+ L + Q+ + E ++ +++ H F ++ LD + WT + + +

Sbjct 1 MALGYLAMLQRKLKEGHGG--EDITYEEMYEFHVNVFNKNGLDISYWTLYTPMSIIQTNA 58

Query 150 -----------GPAKLEQIWTNMLNNDYAGGPRVGNTSVDAISQM-GWTKGGQYLTRLSV 197

G +E +W + + S++ M KG Y+ +++

Sbjct 59 SGSGRNGNMIEGSKVVESMWEQICATKGEVVNGGASVSLELYQIMQDAKKGYIYVDKITG 118

Query 198 LEATQA 203

++Q

Sbjct 119 DVSSQT 124

>WP_090633030.1 tandem-95 repeat protein [Nitrosomonas marina]

SEN38768.1 type I secretion C-terminal target domain (VC_A0849 subclass)

[Nitrosomonas marina]

Length=1865

Score = 79.9 bits (195), Expect = 1e-12, Method: Composition-based stats.

Identities = 29/173 (17%), Positives = 63/173 (36%), Gaps = 17/173 (10%)

Query 1 MSGLTERDLSVLGSYARDGNRELYWNYL--SQLPGA----DGYGTLALGVVRNDSLPGRV 54

++ L+ D L ++Y+ L + Y LA VV + ++ G +

Sbjct 12 LANLSFEDFKAL-HIQSQNRADVYYKALKLAAQDAGLVDVVNYADLARNVVNDSNINGVL 70

Query 55 ANTYAQDYAKSQQEEGSRFPNAQLTERQWESFGQTLLERDLELRQQWMNERRPDLALNLP 114

AN + ++ A + + S +++L L++ D+ R L

Sbjct 71 ANNHTENIANNLGIDFSAGADSRL------RTQYELMKADVLQRVNNKLAGG---NGELN 121

Query 115 GKDVMLAHDRAFERHELDPNCWTPRVLLQAAEQKSGPAKLEQIWTNMLNNDYA 167

++ + H A + L P ++ L K P + E+++ +L +

Sbjct 122 FQETIDIHTSALSKSNLPPEAFSLYAPLS-ILAKHNPDRAEKLFKAVLPGEGF 173

>MAB33466.1 hypothetical protein [Thalassospira sp.]

Length=3409

Score = 79.1 bits (193), Expect = 2e-12, Method: Composition-based stats.

Identities = 30/160 (19%), Positives = 60/160 (38%), Gaps = 22/160 (14%)

Query 1 MSGLTERDLSVLGSYARDGNRELYWNYLSQLPGADGYGTLALGVVRNDSLPGRVANTYAQ 60

M+ L+E+ ++ L + A G+ + +S+ GYG LAL V G AN

Sbjct 1 MTQLSEQQINSLRTLADTGDIGGVYKQMSE--FGVGYGELALDVYSGQGFGGIYANNLLT 58

Query 61 DYAKSQQEEGSRFPNAQLTERQWESFGQTLLERDLELRQQWMNERRPDLALNLPGKDVML 120

G + +++ ++++F + L E ++ + + LP ++

Sbjct 59 --------LGDSLNLSLMSDVEFDAFRKKLAEAAIDTVESFGTY--------LPAAEIDR 102

Query 121 AHDRAFERHELDPNCWTPRVLLQAAEQKSGPAKLEQIWTN 160

H+ F +++L W G LE +

Sbjct 103 FHEEIFAKYQLP---WFSYGGAN-YGDLFGQEALENYYNT 138

>WP_038601691.1 hypothetical protein [Rickettsiales bacterium Ac37b]

AIL64519.1 hypothetical protein NOVO_00545 [Rickettsiales bacterium Ac37b]

Length=958

Score = 76.4 bits (186), Expect = 2e-11, Method: Composition-based stats.

Identities = 29/147 (20%), Positives = 49/147 (33%), Gaps = 17/147 (12%)

Query 3 GLTERDLSVLGSYARDGNRELYWNYLSQLPGADGYGTLALGVVRNDSLPGRVANTYAQDY 62

L+ ++++ A Y+NYL+ YG LA VV N +AN + +

Sbjct 29 PLSSQEVA-----ASTKIVNEYYNYLANS--GIAYGGLARDVVNNHGSFAGLANNHLEHR 81

Query 63 AKSQQEEGSRFPNAQLTERQWESFGQTLLERDLELRQQWMNERRPDLALNLPGKDVMLAH 122

A + + + N + + L D LR + + + H

Sbjct 82 ALQEGKSIEQIANIK------NNIRVALAYNDANLRSDNKES----INGLIGYNKIANYH 131

Query 123 DRAFERHELDPNCWTPRVLLQAAEQKS 149

AFE+ L W + A S

Sbjct 132 YDAFEKQGLSKYAWGGTFFEEFAGSGS 158

>WP_093316205.1 type VI secretion system tip protein VgrG [Sphingomonas jatrophae]

SFS09806.1 type VI secretion system secreted protein VgrG [Sphingomonas

jatrophae]

Length=771

Score = 75.6 bits (184), Expect = 2e-11, Method: Composition-based stats.

Identities = 31/146 (21%), Positives = 53/146 (36%), Gaps = 19/146 (13%)

Query 13 GSYARDGNRELYWNYLSQLPGADGYGTLALGVVRNDSLPGRVANTYAQDYAKSQQEEGSR 72

++ARDGNR +W S+L D A +V + S G AN ++ +S+ +

Sbjct 643 RAFARDGNRRAFW--ASRLARGDKMARTATEIVDSSSARGYTANMRLRNGLRSRDLDPFN 700

Query 73 FPNAQLTERQWESFGQTLLERDLELRQQWMNERRPDLALNLPGKDVMLAHDRAFERHELD 132

+ + E G+ L+ + D L + H + F H L

Sbjct 701 NY-----DAEVEQVGRELMREHVA---------GTDRFGTLTPPQIADYHHKVFRDHGLP 746

Query 133 PNCWTPRVLLQAAEQKSGPAKLEQIW 158

P + + +S EQ+W

Sbjct 747 PTMFGGTM---ITGNRSEADWYEQLW 769

>WP_107794511.1 tandem-95 repeat protein [Nitrosomonas aestuarii]

PTN12512.1 putative secreted protein (type I secretion substrate) [Nitrosomonas

aestuarii]

Length=2035

Score = 73.3 bits (178), Expect = 2e-10, Method: Composition-based stats.

Identities = 38/229 (17%), Positives = 76/229 (33%), Gaps = 37/229 (16%)

Query 2 SGLTERD----LSVL--GSYARDGNRELYWNYLSQLPGADGYGTLALGVVRNDSLPGRVA 55

GL++ + L++L + ++ +++YL + YG L LGV N++ G+ A

Sbjct 1 MGLSQTERTEALAILGDQTKSKTERSRDFYSYL--NDKGEDYGRLGLGVTENNTWQGKWA 58

Query 56 NTYAQDYAKSQQEEGSRFPNAQLTERQWESFGQTLLERDLELRQQWMNERRPDLALNLPG 115

N +A+ A++ + ++W L ER + Q +++ +

Sbjct 59 NGFAESAAQNNGGGF------EHGSQKWVDTNFRLAERHM---QTYIDNQGETPGR---- 105

Query 116 KDVMLAHDRAFERHELDPNCWTPRVLLQAAEQKSGPAKLEQIWTNMLNND-----YAGGP 170

D+ H+ + L+ N W P +L + G +W + + ND

Sbjct 106 SDIQQYHNAEYLFQGLNVNDWLPNKMLNESSDPDG------LWVDWMRNDNPADVMQDAV 159

Query 171 RVGNTSVDA-----ISQMGWTKGGQYLTRLSVLEATQALEGRSAVDPNV 214

+ + DPNV

Sbjct 160 LTAKAGGAIIIPPFFMVQAISDPQGLPEAMEFARNLHGGMTNVMSDPNV 208

>WP_062628421.1 hypothetical protein [Halocynthiibacter arcticus]

AML53805.1 hypothetical protein RC74_21365 [Halocynthiibacter arcticus]

Length=1261

Score = 66.8 bits (161), Expect = 3e-08, Method: Composition-based stats.

Identities = 41/210 (20%), Positives = 75/210 (36%), Gaps = 30/210 (14%)

Query 44 VVRNDSLPGRVANTYAQDYAKSQQEEGSRFPNAQLTERQWESFGQTLLERDLELRQQWMN 103

+V ND++ G AN++ A ++Q+ +T S G L+E DL+LR +

Sbjct 1 MVLNDTIAGAGANSFFLTTATTEQKT--------ITGNDLASVGLALMEEDLKLR--ILR 50

Query 104 ERRPDLALNLPGKDVMLAHDRAFER-HELDPNCWTPRVLLQAAEQKSGPAKLEQIWTNML 162

+L G D H F R + WTP + L + + + W +

Sbjct 51 NGA-----DLSGDDYERYHREVFGRVAGVSAEAWTPTIFLDSF---GDAIERQDAWVEL- 101

Query 163 NNDYAGGPRVGNTSVDAISQMGWTKGGQYLTRLSVLEATQALEGRSAVDPNV---IGGNS 219

AG + + ++ +M T +YL + A+ IG

Sbjct 102 ----AGSGPIASYNLIVARRMDVTN--EYLDNTTTPLGGIVFSDLDAMTKETMLAIGIEE 155

Query 220 YYAMYFEADRKWA-SISAGGGHMSLREITD 248

+A +F + + G S + ++

Sbjct 156 EFADWFIQYSVYLDRLIEAGFPESFVDSSN 185

>WP_080927886.1 hypothetical protein [Vibrio nigripulchritudo]

Length=2452

Score = 64.9 bits (156), Expect = 1e-07, Method: Composition-based stats.

Identities = 27/163 (17%), Positives = 49/163 (30%), Gaps = 31/163 (19%)

Query 3 GLTERDLSVLGSYARDGNRELYWNYLSQLPGADGYGTLALGVVRNDS------LPGRVAN 56

G+TE DG + +W S+L D L + + G N

Sbjct 2307 GVTEEQ----RQLVADGKIQEFWE--SRLAVGDPVARAGLASLNPEGGVVDYLFGGTSIN 2360

Query 57 TYAQDYAKSQQEEGSRFPNAQLTERQWESFGQTLLERDLELRQQWMNERRPDLALNLPGK 116

+ Q +A + + L + ++ + R + L

Sbjct 2361 SRLQAFANVYNDGVLN----------IDQVRVDLATAHI----KFTDGDRLGVRGLLNPG 2406

Query 117 DVMLAHDRAFERHELDPNCWTPRVLLQAAEQKSGPAKLEQ-IW 158

+ H + F+RH L P + A G A + + +W

Sbjct 2407 QIAEYHHQVFDRHGLPPTTFGGTPFTGAV----GEAWVTRPVW 2445

>WP_082391774.1 hypothetical protein [Neisseria sp. 83E34]

Length=1326

Score = 64.1 bits (154), Expect = 2e-07, Method: Composition-based stats.

Identities = 12/58 (21%), Positives = 25/58 (43%), Gaps = 0/58 (0%)

Query 118 VMLAHDRAFERHELDPNCWTPRVLLQAAEQKSGPAKLEQIWTNMLNNDYAGGPRVGNT 175

+ H+ F+ H L+ N WT + + +G E+ W ++ + G + N+

Sbjct 1 MRDFHESVFKDHGLNINNWTLEYPMSLVGKYAGKKVQEKYWQDLSKTEGDGVDGLWNS 58
